# Enantioselective organocatalytic Michael reactions using chiral (*R*,*R*)-1,2-diphenylethylenediamine-derived thioureas†

**DOI:** 10.1039/d0ra03550e

**Published:** 2020-08-27

**Authors:** Jae Ho Shim, Min Ji Lee, Min Ho Lee, Byeong-Seon Kim, Deok-Chan Ha

**Affiliations:** Laboratory of Organic Synthesis, Department of Chemistry, Research Institute for Natural Sciences, Korea University Seoul 136-713 Korea dechha@korea.ac.kr; Department of Chemistry Education, Research Institute of Natural Science, Gyeongsang National University Jinju 52828 Korea bkim@gnu.ac.kr

## Abstract

Although the Michael addition is a very well-known and widely applied reaction, cost-effective, metal-free, and readily prepared organic catalysts remain rare. A chiral, bifunctional, (*R*,*R*)-1,2-diphenylethylenediamine-derived thiourea organic catalyst was developed and applied to asymmetric Michael additions of nitroalkenes under neutral conditions. Generally, fluorine-substituted thiourea catalysts exhibited high chemical yields and enantioselectivities under neutral conditions. The mild reactions were tolerant of many functional groups and afforded good-to-excellent yields, as well as high diastereo- and enantioselectivities for the Michael adducts. The utility of the transformation was demonstrated by the synthesis of a bioactive compound, (*R*)-Phenibut.

## Abstract 2

Although the Michael addition is a very well-known and widely applied reaction, cost-effective, metal-free, and readily prepared organic catalysts remain rare.

## Graphical abstract

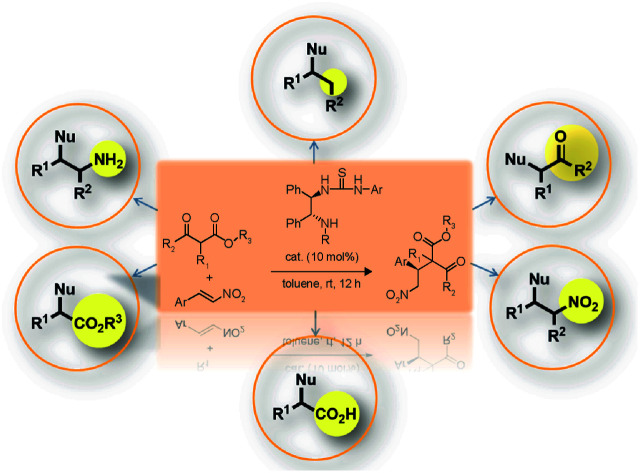

## Introduction

Numerous studies on metal-free stereoselective organic catalysts have been reported over the past century.^1^ Although stereoselective metal-catalyzed reactions generally provide more reliable results than those catalyzed by organic compounds, metal catalysts are disadvantageous in several respects. They are typically expensive, which leads to higher production costs.^2^ Moreover, the metal waste remaining after completion of the reaction can contaminate both the product and/or the environment.^3^ To overcome these drawbacks, stereoselective syntheses using metal-free organic catalysts are becoming increasingly important. With this in mind, our group has been motivated to examine organic catalysts from a variety of perspectives, and we previously reported the application of a thiourea catalyst derived from (*R*,*R*)-1,2-diphenylethane-1,2-diamine (DPEN)^4^ in several reactions.

**Fig. 1:**
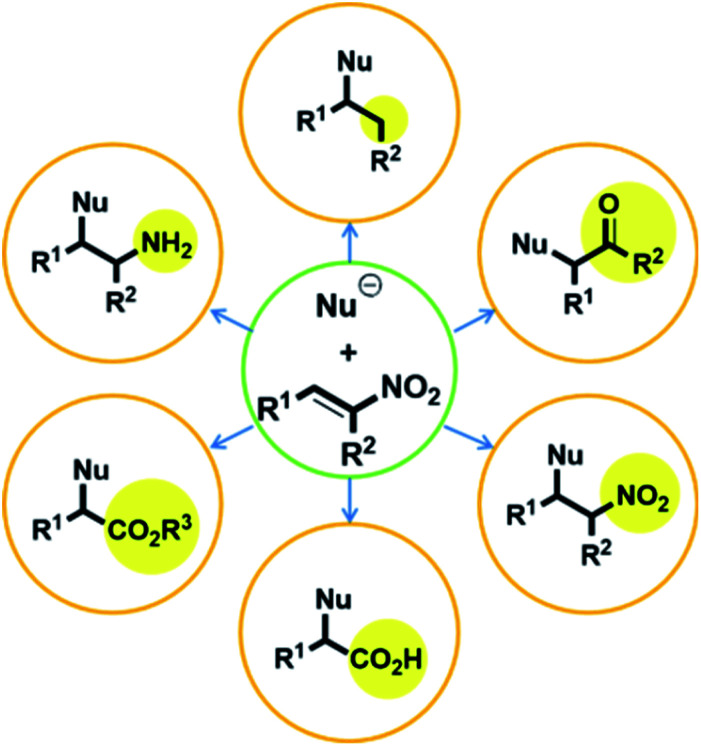
Transformations of nitro compounds.

Compounds with adjacent quaternary and tertiary stereo-centers can be prepared by Michael additions to electron-deficient olefins, as exemplified by the reactions of trisubstituted carbon nucleophiles with nitroalkenes; these products can serve as building blocks for the syntheses of complex natural materials. In this regard, the Michael reaction is one of the most important C–C bond-forming reactions. However, despite its high synthetic potential, the number of highly stereoselective Michael-addition-based synthetic methods is limited.^6,7^ Nitro-group-containing Michael acceptors are very attractive due to their highly electron-deficient properties and because the nitro group is easily converted into other functional groups, including ketones, esters, amine, and carboxylic acids (Fig. 1).^5^

The Takemoto group reported high enantioselectivity in a Michael reaction involving a malonate and nitroalkene using an (*R*,*R*)-1,2-cyclohexyldiamine-thiourea-based catalyst.^8^ In another study, they obtained high enantio- and diastereoselectivities using a thiourea-derived catalyst in a Michael reaction involving a β-ketoester and nitroalkene.^9^ Cavallo and co-workers recently explored the use of hexafluorobenzene (C_6_F_6_) as the solvent in a study into the asymmetric Michael reaction of a β-ketoester and nitroalkene.^10^ Conventionally, hydrogen-bond-forming noncovalent catalysts afford good stereoselectivities in nonpolar solvents such as toluene. On the other hand, hexa-fluorobenzene (*ε* = 2.05) stabilizes the enol form of the β-keto ester through π-stacking, which may lead to good reactivity and stereoselectivity.

Herein, we report on the catalytic activities of new chiral organocatalysts based on (*R*,*R*)-DPEN-derived thioureas^11^ in the asymmetric Michael addition reactions of nitroalkenes.

## Results and discussion

The Michael addition reaction of *trans*-β-nitrostyrene 3a with 2-carboxyethylcyclopentanone 2a was first screened with catalyst 1a under neutral conditions. This catalyst is a highly basic *N*-monosubstituted thiourea that lacks an alkyl group (R^1^ = H) on the amine of the DPEN unit (Table 1). Catalyst effectiveness was initially investigated at room temperature in CH_2_Cl_2_, affording the desired product with 29% ee (Table 1, entry 1). This result suggests that the catalyst can impart stereoselectivity to some extent even without an alkyl group (R^1^) on the terminal amine. Subsequent reactions were performed with 3-pentyl-substituted thiourea catalysts 1b–1h.

**Table 1:** Catalyst and temperature optimization

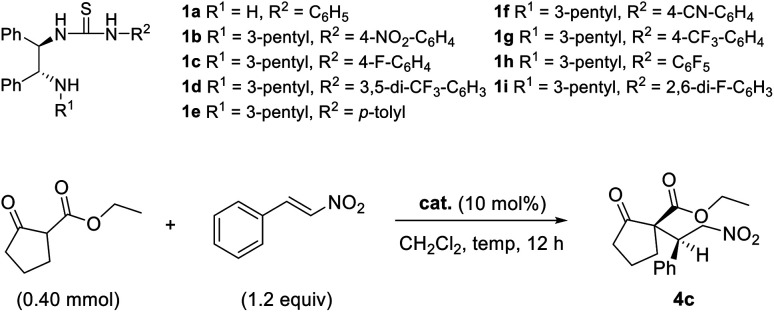					
Entry	Cat.	Temp (°C)	Yield^a^ (%)	dr^b^	ee^b^ (%)
1	1a	Ambient	98	87 : 13	29
2	1b	Ambient	90	66 : 44	67
3	1c	Ambient	94	70 : 30	78
4	1d	Ambient	94	93 : 7	73
5	1e	Ambient	83	92 : 8	60
6	1f	Ambient	89	96 : 4	68
7	1g	Ambient	88	74 : 26	50
8	1h	Ambient	99	95 : 5	93
9	1i	Ambient	91	96 : 4	15
10	1h	0	96	97 : 3	83
11	1h	−30	96	98 : 2	91

a Isolated yield.

b Determined by chiral-phase HPLC using an OD-H column.

To increase the potential for hydrogen bonding *via* the hydrogen atoms of the thiourea, we adjusted their acidities by introducing different groups (R^2^) on the urea moiety. Catalyst 1e bearing an electron-donating *p*-tolyl substituent gave product 4c with slightly lower enantioselectivity (60%) than 1b–1d, which contained electron-withdrawing groups (entries 2–5). However, the use of catalyst 1g, which also contained an electron-withdrawing group, led to much lower enantio-selectivity than that of 1e (entry 5), which indicates that the electronic nature of the R^2^ group does not necessarily determine the outcome of the reaction. Overall, the catalysts bearing electron-withdrawing substituents provide higher enantioselectivities than those with electron-donating groups. After securing the highest enantioselectivity with pentafluorophenyl-substituted catalyst 1h (Table 1, entry 8), Again, the 3-pentyl-substituted catalyst provided the highest enantioselectivity among the catalysts investigated.

We next probed the effect of lower reaction temperatures using catalyst 1h; unfortunately, lower enantioselectivities were obtained than at room temperature (entries 10–11). Based on the results summarized in Table 1, we conclude that the optimal reaction conditions involve the use of the pentafluorophenyl-substituted catalyst 1h at room temperature (Table 1, entry 8).

With the optimal catalyst and temperature conditions in hand, we examined the effect of solvent on reactivity and enantioselectivity (Table 2). Other than hexane, good overall enantioselectivities were obtained in all the solvents examined. Although the best diastereoselectivity was observed in trifluorotoluene (Table 1, entry 5), the catalyst was less enantioselective in this solvent than in toluene, and the reaction took longer for a lower yield (Table 1, entry 2). Therefore, we selected the optimal solvent as toluene with a reaction time of 12 h.

**Table 2:** Solvent optimization

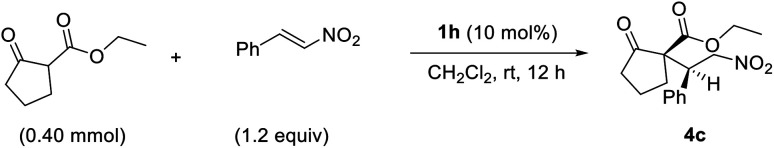					
Entry	Solvent	Time (h)	Yield^a^ (%)	dr^b^	ee^b^ (%)
1	Hexane	12	74	95 : 5	62
2	Toluene	12	87	98 : 2	97
3	Et_2_O	12	75	97 : 3	95
4	THF	96	67	95 : 5	92
5	Trifluorotoluene	72	76	99 : 1	94
6	CH_2_Cl_2_	12	99	95 : 5	93

a Isolated yields.

b Determined by chiral-phase HPLC using an OD-H column.

**Scheme 1:**
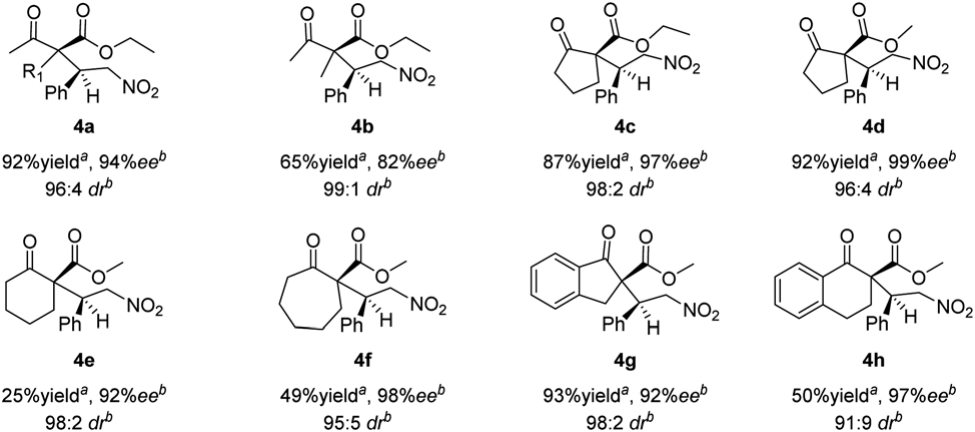
Asymmetric Michael additions of β-ketoesters to *trans*-β-nitrostyrene. ^*a*^Isolated yield. ^*b*^Determined by HPLC using a chiral column.

The Michael addition reactions of a variety of β-ketoesters with *trans*-β-nitrostyrene mediated by catalyst 1h were surveyed (Scheme 1). Good overall enantioselectivities were observed regardless of the substituent on the β-keto ester; however, the product yields were poor when cyclohexanone 2e, cycloheptanone 2f, and α-tetralone 2h were used as substrates. The results also show that 4a was produced from ethyl acetoacetate 2a with low diastereoselectivity, which is ascribed to epimerization.

Various nitroalkenes 3 were reacted with methyl 2-oxo-cyclopentanecarboxylate (2d) and methyl 2-oxocyclohexane-carboxylate (2e) (Table 3). Good enantioselectivities were observed when nitroalkenes 3 were reacted with 2d, regardless of whether the substituent was electron-withdrawing or electron-donating. However, lower yields were obtained when *trans*-nitroolefins bearing *ortho*-substituted aromatic substituents or aliphatic substituents were used. Good enantioselectivities were observed when 2e was reacted with nitroalkenes bearing both electron-withdrawing and electron-donating substituents. As previously observed, lower yields of 4 were obtained with 2e than with 2d.

**Table 3:** Asymmetric Michael additions of cyclic β-ketoesters to nitro-olefins

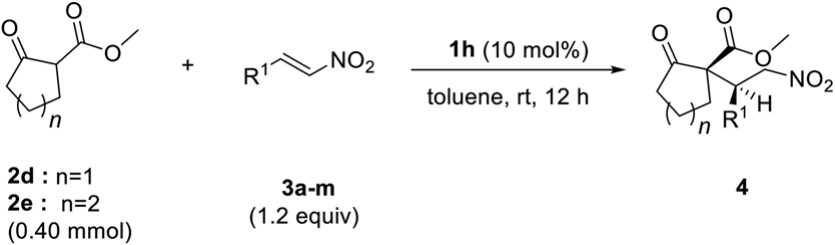						
Entry	2	R^1^	4	Yield^a^ (%)	dr^b^	ee^b^ (%)
1	2d	Ph	4d	92	96 : 4	99
2	2d	*p*-Tolyl	4i	85	88 : 12	90
3	2d	4-Cl-Ph	4j	85	96 : 4	98
4	2d	4-Br-Ph	4k	88	96 : 4	98
5	2d	4-MeO-Ph	4l	92	93 : 7	96
6	2d	2-MeO-Ph	4m	25	98 : 2	92
7	2d	Bu	4n	60	99 : 1	98
8	2d	Cyclohexyl	4o	42	99 : 1	98
9	2d	i-Pr	4p	41	99 : 1	98
10	2e	Ph	4e	25	98 : 2	92
11	2e	*p*-Tolyl	4q	24	95 : 5	98
12	2e	4-Cl-Ph	4r	50	99 : 1	98
13	2e	4-Br-Ph	4s	26	99 : 1	94
14	2e	4-OMe-Ph	4t	19	99 : 1	80

a Isolated yields.

b Determined by HPLC using a chiral column.

**Fig. 2:**
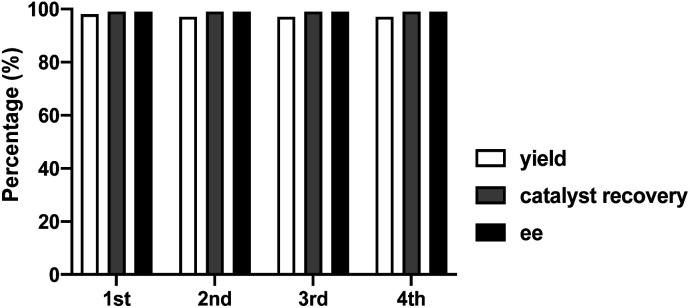
Recycling test for asymmetric Michael addition using chiral (*R*,*R*)-1,2-diphenylethylenediamine-derived thiourea catalyst (1d).

**Scheme 2:**
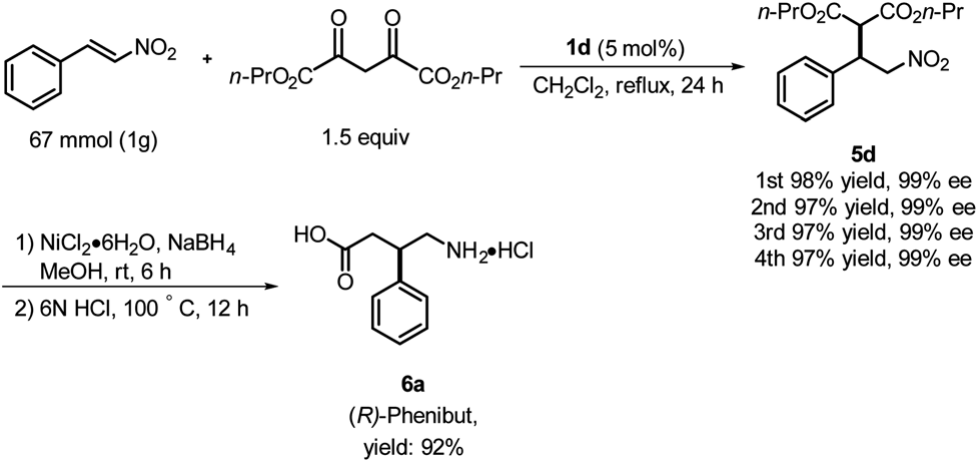
Synthesis of the inhibitory neurotransmitter Phenibut.

Finally, to fully explore substituent effects in these reactions, variously substituted malonates and nitroalkenes were subjected to the reaction. In these cases, we used catalyst 1d because when use 1h catalyst in this reactions, it give more lower ee than 1d catalyst. Although good overall yields and enantioselectivities were obtained, the yields decreased with the increasing size and length of the malonate ester groups (R^1^) (Table 4, entries 1–6). These results suggest that the reaction rate decreases with increasing steric bulk of the malonate substituent. Further experiments were performed using diethyl malonate and various β-nitrostyrenes.^11^ The product and ee yields were generally lower than those obtained using the parent β-nitrostyrene (Table 4, entries 7–13); however, good yields and enantioselectivities were obtained for β-nitrostyrenes with electron-withdrawing 4-bromo and 4-chloro substituents. In contrast, lower yields and enantioselectivities were observed for β-nitrostyrenes substituted with electron-donating 4- and 2-methoxy groups. These results indicate that β-nitrostyrenes bearing electron-withdrawing groups are better Michael acceptors toward the nucleophile because the double bond is more electron-deficient, which facilitates nucleophilic attack. We tested recycling of the catalysts 1h (Fig. 2, ESI Table 1†). In these four times test, we obtained data for the (*R*,*R*)-1,2-diphenylethylenediamine-derived thiourea catalyst (1d) could be recyclable (Scheme 2).

**Table 4:** Michael reactions using catalyst 1d (R^1^ = 3-pentyl; R^2^ = 3,5-di-CF_3_-C_6_H_3_)

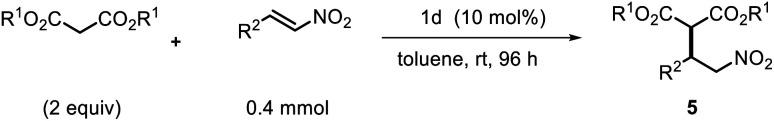					
Entry	R^1^	R^2^	Product	Yield^a^ [%]	ee^b^ [%]
1	Me	Ph	5a	89	80
2	Et	Ph	5b	85	90
3^c^	Et	Ph	5b	86	78
4	i-Pr	Ph	5c	49	99
5	*n*-Pr	Ph	5d	45	98
6^d^	*n*-Pr	Ph	5d	98	99
7	Bu	Ph	5e	41	94
8	Et	4-Br-Ph	5f	77	88
9	Et	4-Cl-Ph	5g	72	89
10	Et	4-Me-Ph	5h	60	84
11	Et	4-MeO-Ph	5i	40	89
12	Et	2-MeO-Ph	5j	51	70
13	Et	4-HO-Ph	5k	40	89
14	Et	Furyl	5l	78	72

a Isolated yield.

b Determined by chiral HPLC.

c Using the 1h catalyst for reaction.

d In CH_2_Cl_2_ for 12 h heated at reflux.

Based on the foregoing results, we envisioned that these Michael addition products would provide access to bioactive compounds such as the inhibitory neurotransmitter and antidepressant, (*R*)-Phenibut® (6a, Scheme 2).^12,13^ To achieve this objective, we used previously prepared adduct 5d from the reaction of *n*-propyl malonate with β-nitrostyrene.

The nitro group of 5d was reduced with NiCl_2_·6H_2_O and NaBH_4_ (Scheme 2), which resulted in formation of a cyclic amide, *i.e.*, an ester-bearing pyrrolidinone. Pyrrolidinone ring opening and decarboxylation were achieved by treatment with HCl, which afforded β-phenyl-γ-aminobutanoic acid (GABA) derivative 6a.^11^

**Fig. 3:**
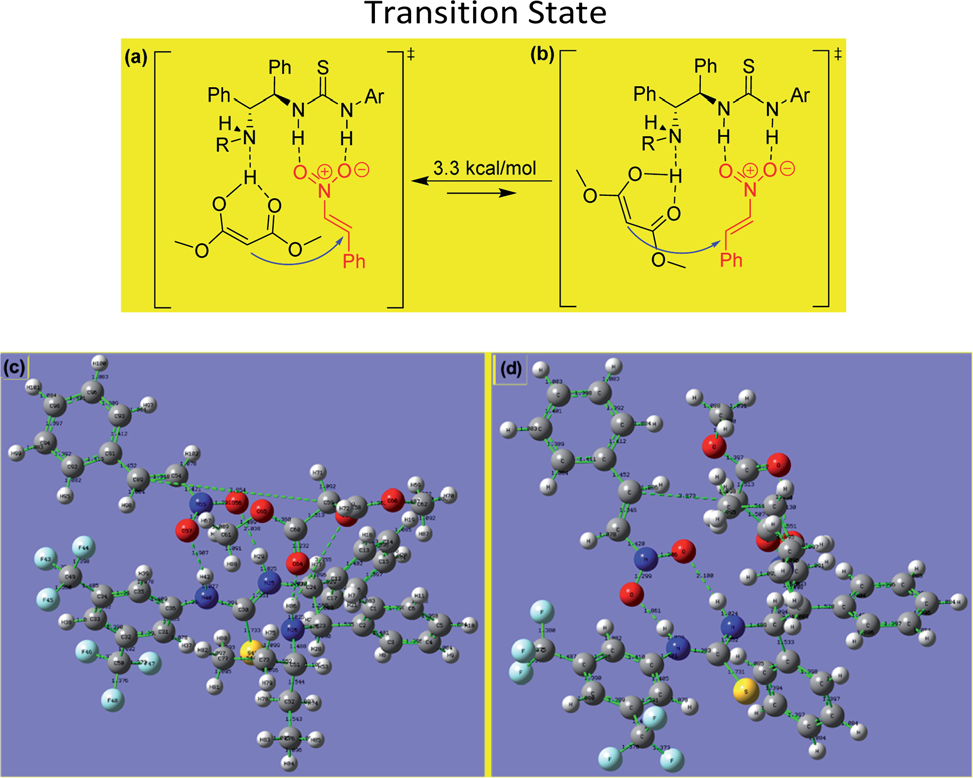
(a, b) Proposed transition state for asymmetric Michael addition using chiral (*R*,*R*)-1,2-diphenylethylenediamine-derived thiourea and difference of relative free energy. (c, d) B3LYP/6-31G(d,p)-calculated transition state of the DPEN-thiourea-catalyzed enantioselective Michael reaction. Transition state structures for the C–C bond formation, through which the main product (R) is possibly formed, are also shown.

The experimental results obtained for the stereoselective Michael addition reactions using malonate derivatives and nitroalkenes provide insight into the reaction mechanism and make it possible to infer the role of the catalyst in enabling the reaction (Fig. 3).

In the transition state involved in the present catalytic reaction, the nitro atoms of the thiourea group of the catalyst form hydrogen bonds with the oxygen atoms of the nitroalkene, fixing its position and increasing the reactivity of the electrophilic double bond. Additionally, the enol form of the malonate interacts *via* hydrogen bonding with the free amino group of the catalyst, situating it for attack of the nitroalkene from behind to form the (*R*)-product.

## Conclusions

The Michael additions of 1,3-dicarbonyl compounds to nitroalkenes catalyzed by *N*-monosubstituted thiourea derivatives of DPEN provided products with good-to-excellent enantio- and diastereoselectivities. These catalysts are cost-effective because they are more readily prepared than conventional organic catalysts. The Michael adducts possessed adjacent quaternary and tertiary stereocenters and were obtained in relatively good yields. Furthermore, since the γ-lactone intermediate prepared by this reaction can be used as an intermediate for the preparation of anti-depressant drugs, further studies on its medical applications must be conducted, due to the increasing need for investigations of the medical and biological applications of such compounds.

## Experimental

### General procedure for the asymmetric Michael reaction (4a–4t)

The β-nitrostyrene (1.2 equiv.), β-ketoester (2.0 equiv.), and 1d (10 mol%) were mixed in toluene (0.4 M) and the reaction mixture was stirred at ambient temperature. The reaction conversion was monitored by TLC. After completion, ethyl acetate (0.2 mL) was added in the reaction mixture. This solution was washed twice with water (2 × 1.0 mL), dried over magnesium sulfate, and concentrated to yield the desired product. The product was purified by chromatography on a silica-gel column (hexanes/ethyl acetate 5 : 1).

#### Ethyl 2-acetyl-4-nitro-3-phenylbutanoate (4a)

Colorless oil; [*α*]^20^_D_ = −196.3 (*c* = 0.0575, CH_2_Cl_2_); ^1^H NMR (300 MHz, CDCl_3_) *δ* 7.30 (m, 3H), 7.21 (d, *J* = 6.6 Hz, 2H), 4.80 (m, 2H), 4.22 (m, 1.8H), 4.12 (d, *J* = 10.1 Hz, 0.6H), 4.03 (d, *J* = 9.0 Hz, 0.4H), 3.96 (q, *J* = 15 Hz, 1.2H), 2.30 (s, 1.6H), 2.06 (s, 1.4H), 1.28 (t, *J* = 15.0 Hz, 1.2H), 1.00 (t, *J* = 12.0 Hz, 1.8H); ^13^C NMR (100 MHz, CDCl_3_) *δ* 201.3, 200.5, 167.7, 167.0, 136.6, 136.5, 129.3, 129.1, 128.4, 128.1, 78.1, 78.0, 62.4, 62.1, 61.8, 42.7, 42.5, 30.5, 30.3, 14.2, 13.8; IR (CHCl_3_) *ν* 3031, 2987, 1741, 1718, 1556, 1375 cm^−1^; HRMS (FAB^+^) calcd for [C_14_H_18_NO_5_]^+^: 280.1185, found: 280.1187; HPLC [Chiralcel AD-H, hexane/2-propanol = 80/20, 0.8 mL min^−1^, *λ* = 210 nm] retention times: (major diastereomer) 10.0, 11.4 min, (minor diastereomer) 8.0, 19.9 min.

#### Ethyl 2-acetyl-2-methyl-4-nitro-3-phenylbutanoate (4b)

Colorless solid; mp 73–75 °C; [*α*]^20^_D_ = −103.8 (*c* = 0.0115, CH_2_Cl_2_); ^1^H NMR (300 MHz, CDCl_3_) *δ* 7.30–7.26 (m, 3H), 7.23–7.20 (m, 2H), 4.95 (d, *J* = 9 Hz, 2H), 4.23 (t, *J* = 6 Hz, 1H), 4.14–4.08 (m, 1H), 4.07–4.01 (m, 1H), 2.12 (s, 3H), 1.43 (s, 3H), 1.20 (t, *J* = 7.5 Hz, 3H); ^13^C NMR (100 MHz, CDCl_3_) *δ* 205.6, 171.0, 135.6, 129.4, 128.9, 128.5, 76.9, 62.3, 62.1, 47.5, 27.8, 18.2, 14.0; IR (CHCl_3_) *ν* 3031, 1713, 1557 cm^−1^; HRMS (FAB^+^) calcd for [C_15_H_20_NO_5_]^+^: 294.1341, found: 294.1342; HPLC [Chiralcel OD-H, hexanes/2-propanol = 90/10, 0.5 mL min^−1^, *λ* = 210 nm] retention times: (major diastereomer) 21.4, 30.6 min, (minor diastereomers) 15.5, 19.2 min.

#### Ethyl 1-(2-nitro-1-phenylethyl)-2-oxocyclopentanecarboxylate (4c)

Colorless oil; [*α*]^20^_D_ = +10.7 (*c* = 1.000, CH_2_Cl_2_); ^1^H NMR (300 MHz, CDCl_3_) *δ* 7.20–7.27 (m, 5H), 5.15–5.21 (dd, *J* = 4.2 Hz, 3.9 Hz, 1H), 4.98–5.06 (dd, *J* = 11.2 Hz, 11.0 Hz, 1H), 4.18–4.25 (m, 2H), 4.06–4.11 (dd, *J* = 3.8 Hz, 3.8 Hz, 1H), 2.30–2.45 (m, 2H), 1.79–2.07 (m, 4H), 1.25–1.30 (t, *J* = 7.2 Hz, 3H); ^13^C NMR (100 MHz, CDCl_3_) *δ* 207.3, 169.8, 135.5, 129.6, 128.6, 128.4, 77.7, 62.17, 47.9, 41.6, 37.2, 28.1, 22.5, 14.2; IR (CHCl_3_) *ν* 3031, 2957, 1751, 1727, 1556 cm^−1^; HRMS (FAB^+^) calcd for [C_16_H_20_NO_5_]^+^: 306.1341, found: 306.1341; major diastereomer: ee was determined by HPLC [Chiralpak OD-H column, hexanes/2-propanol = 93/7, flow rate = 1 mL min^−1^, *λ* = 220 nm] retention times: (major enantiomer) 19.4 min, (minor enantiomer) 13.2 min, (minor diastereomers) 11.3, 15.7 min.

#### Methyl 1-(2-nitro-1-phenylethyl)-2-oxocyclopentanecarboxylate (4d)

Colorless oil; [*α*]^20^_D_ = −152.9 (*c* = 0.045, CH_2_Cl_2_); ^1^H NMR (300 MHz, CDCl_3_) *δ* 7.34–7.22 (m, 5H), 5.17 (dd, *J* = 3.8, 13.5 Hz, 1H), 5.01 (dd, *J* = 10.7, 13.5 Hz, 1H), 4.08 (dd, *J* = 3.9, 10.8 Hz, 1H), 3.76 (s, 3H), 2.42–2.30 (m, 2H), 2.09–1.79 (m, 4H); ^13^C NMR for major diastereomer (100 MHz, CDCl_3_) *δ* 212.5, 170.0, 135.4, 129.5, 129.0, 128.5, 76.6, 62.6, 53.2, 46.3, 38.1, 31.2, 19.5; IR (CHCl_3_) *ν* 3031, 2957, 1751, 1727, 1556 cm^−1^; HRMS (FAB^+^) calcd for [C_15_H_19_NO_5_]^+^: 292.1185, found: 292.1180; HPLC [Chiralcel OD-H, hexanes/2-propanol = 93/7, 0.5 mL min^−1^, *λ* = 210 nm] retention times: (major enantiomer) 28.8 min, (minor enantiomer) 23.3 min, (minor diastereomers) 15.7, 18.5 min.

#### Methyl 1-(2-nitro-1-phenylethyl)-2-oxocyclohexanecarboxylate (4e)

Colorless solid; mp 100–102 °C; [*α*]^20^_D_ = +61.7 (*c* = 0.018, CH_2_Cl_2_); ^1^H NMR (300 MHz, CDCl_3_) *δ* 7.30–7.27 (m, 3H), 7.17–7.12 (m, 2H), 5.06 (dd, *J* = 3.1, 13.5 Hz, 0.96H), 4.78 (dd, *J* = 11.2, 13.2 Hz, 1H), 4.01 (dd, *J* = 3, 11.3 Hz, 0.96H), 3.75 (s, 2.88H), 3.68 (s, 0.12H), 2.57–2.39 (m, 2H), 2.14–1.98 (m, 2H), 1.77–1.45 (m, 4H); ^13^C NMR (100 MHz, CDCl_3_) *δ* 207.1, 170.3, 135.4, 129.5, 128.7, 128.4, 77.6, 63.2, 52.7, 47.8, 41.6, 37.1, 28.1, 22.5; IR (CHCl_3_) *ν* 3027, 2951, 1713, 1556 cm^−1^; HRMS (FAB^+^) calcd for [C_16_H_20_NO_5_]^+^: 306.1341, found: 306.1340; HPLC [Chiralcel OJ-H, hexanes/2-propanol = 80/20, 0.5 mL min^−1^, *λ* = 210 nm] retention times: (major enantiomer) 50.8 min, (minor enantiomer) 45.6 min, (minor diastereomers) 71.7, 94.4 min.

#### Methyl 1-(2-nitro-1-phenylethyl)-2-oxocycloheptanecarboxylate (4f)^8,14*a*^

Colorless oil; [*α*]^20^_D_ = +123.3 (*c* = 0.025, CH_2_Cl_2_); ^1^H NMR (300 MHz, CDCl_3_) *δ* 7.30–7.25 (m, 3H), 7.17–7.10 (m, 2H), 4.99–4.89 (m, 2H), 4.07 (dd, *J* = 5, 9.1 Hz, 1H), 3.77 (s, 3H), 2.61–2.53 (m, 2H), 2.16–1.23 (m, 8H); ^13^C NMR (100 MHz, CDCl_3_) *δ* (major diastereomer) 208.4, 171.5, 135.7, 129.5, 128.9, 128.5, 78.0, 65.7, 52.7, 48.7, 41.6, 33.1, 29.2, 25.3, 24.7; IR (CDCl_3_) *ν* 3030, 2938, 2863, 1736, 1709, 1556 cm^−1^; HPLC [Chiralcel AD-H, hexanes/2-propanol = 90/10, 0.5 mL min^−1^, *λ* = 210 nm] retention times: (major enantiomer) 18.7 min, (minor enantiomer) 22.2 min, (minor diastereomers) 17.3, 20.4 min.

#### Methyl 2-(2-nitro-1-phenylethyl)-1-oxo-2,3-dihydro-1*H*-indene-2-carboxylate (4g)^8,14*a*^

Yellow amorphous; [*α*]^20^_D_ = −48.5 (*c* = 0.064, CH_2_Cl_2_); ^1^H NMR (300 MHz, CDCl_3_) *δ* 7.77 (d, *J* = 7.7 Hz, 0.57H), 7.68 (d, *J* = 7.4 Hz, 0.43H), 7.58 (t, *J* = 7.5 Hz, 0.43H), 7.51 (t, *J* = 7.5 Hz, 0.57H), 7.42–7.32 (m, 1.43H), 7.29–7.07 (m, 5.57H), 5.43 (dd, *J* = 3.9, 13.8 Hz, 0.43H), 5.24–5.14 (m, 1H), 5.06 (dd, *J* = 3.6, 13.5 Hz, 0.57H), 4.48 (dd, *J* = 3.5, 10.9 Hz, 0.57H), 4.21 (dd, *J* = 3.9, 11 Hz, 0.43H), 3.75 (s, 1.29H), 3.70 (s, 1.71H), 3.65 (d, *J* = 17.9 Hz, 0.43H), 3.49 (d, *J* = 17.6 Hz, 0.57H), 3.22 (d, *J* = 14.3 Hz, 0.43H), 3.16 (d, *J* = 14.3 Hz, 0.57H); ^13^C NMR (100 MHz, CDCl_3_) *δ* 202.1, 200.1, 171.4, 170.1, 152.7, 152.6, 136.3, 135.1, 135.0, 135.8, 134.9, 134.2, 129.26, 129.21, 129.03, 128.8, 128.55, 128.52, 128.26, 128.23, 126.3, 125.3, 124.6, 77.6, 77.03, 63.0, 61.9, 53.4, 47.7, 47.2, 36.76, 35.2; IR (CHCl_3_) *ν* 3035, 2955, 1739, 1711, 1607, 1556 cm^−1^; HPLC [Chiralcel OD-H, hexanes/2-propanol = 90/10, 1 mL min^−1^, *λ* = 210 nm] retention times: (major enantiomer) 42.1 min, (minor enantiomer) 26.6 min, (minor diastereomers) 54.2, 34.4 min.

#### Methyl 1,2,3,4-tetrahydro-2-(2-nitro-1-phenylethyl)-1-oxonaphthalene-2-carboxylate (4h)

Colorless solid; mp 101–103 °C; [*α*]^20^_D_ = +51.0 (*c* = 0.78, CHCl_3_); ^1^H NMR (300 MHz, CDCl_3_) *δ* 8.04 (d, *J* = 7.7 Hz, 1H), 7.50 (t, *J* = 7.6 Hz, 1H), 7.41–7.26 (m, 6H), 7.20 (d, *J* = 7.7 Hz, 1H), 5.16 (dd, *J* = 3.8, 13.5 Hz, 1H), 5.05 (dd, *J* = 10.1, 13.4 Hz, 1H), 4.21 (dd, *J* = 3.8, 10.4 Hz, 1H), 3.72 (s, 0.15H), 3.65 (s, 2.85H), 3.00–2.89 (m, 2H), 2.47–2.37 (m, 1H), 2.10–1.99 (m, 1H); ^13^C NMR (100 MHz, CDCl_3_) *δ* 194.4, 170.4, 142.7, 136.1, 134.3, 131.7, 130.0, 128.9, 128.8, 128.6, 128.4, 127.2, 78.0, 59.9, 52.9, 47.3, 30.9, 25.7; IR (CHCl_3_) *ν* 3031, 2954, 1736, 1687, 1601, 1556 cm^−1^; HRMS (FAB^+^) calcd for [C_20_H_20_NO_5_]^+^: 354.1341, found: 354.1345; HPLC [Chiralcel OD-H, hexanes/2-propanol = 90/10, 0.5 mL min^−1^, *λ* = 254 nm] retention times: (major enantiomer) 68.6 min, (minor enantiomer) 14.7 min, (minor diastereomers) 17.0, 37.4 min.

#### Methyl 1-(2-nitro-1-*p*-tolylethyl)-2-oxocyclopentanecarboxylate (4i)

Colorless oil; [*α*]^20^_D_ = −156.1 (*c* = 0.049, CH_2_Cl_2_); ^1^H NMR (CDCl_3_, 300 MHz) 1.80–2.07 (m, 4H), 2.30 (s, 3H), 2.35–2.42 (m, 2H), 3.75 (s, 3H), 4.05 (dd, *J* = 4.1, 11 Hz, 1H), 4.98 (dd, *J* = 10.7, 13.4 Hz, 1H), 5.13 (dd, *J* = 4.1, 13.7 Hz, 1H), 7.08–7.14 (m, 4H); ^13^C NMR (CDCl_3_, 100 MHz) 212.5, 170.0, 138.2, 132.2, 129.7, 129.3, 76.6, 62.7, 53.2, 46.0, 38.2, 31.2, 21.2, 19.5; IR (CH_2_Cl_2_) *ν* 2967, 2935, 1760, 1613, 1556 cm^−1^; HRMS (FAB^+^) calcd for [C_16_H_20_NO_5_]^+^: 306.1341, found: 306.1340; HPLC [Chiralcel OD-H, hexanes/2-propanol = 97/3, flow rate = 1 mL min^−1^, *λ* = 220 nm] retention times: (major enantiomer) 25.1 min, (minor enantiomer) 16.4 min, (minor diastereomers) 13.7, 18.0 min.

#### Methyl 1-{1-(4-chlorophenyl)-2-nitro}ethyl-2-oxocyclopentanecarboxylate (4j)

Colorless oil; [*α*]^20^_D_ = −135.8 (*c* = 0.052, CH_2_Cl_2_); ^1^H NMR (CDCl_3_, 300 MHz) 1.82–1.98 (m, 3H), 2.00–2.09 (m, 1H), 2.34–2.41 (m, 2H), 3.75 (s, 3H), 4.04 (dd, *J* = 3.9, 14.9 Hz, 1H), 4.98 (dd, *J* = 11.2, 13.7 Hz, 1H), 5.16 (dd, *J* = 3.8, 13.7 Hz, 1H), 7.13–7.29 (m, 4H); ^13^C NMR (CDCl_3_, 100 MHz) 212.3, 169.9, 134.4, 134.1, 130.9, 129.1, 76.4, 62.4, 53.3, 45.7, 38.0, 31.4, 19.5; IR (CH_2_Cl_2_) *ν* 2957, 2920, 2893, 1760, 1563 cm^−1^; HRMS (FAB^+^) calcd for [C_15_H_17_ClNO_5_]^+^: 326.0795, found: 326.0796; HPLC [Chiralcel OD-H, hexanes/2-propanol = 97/3, flow rate = 1 mL min^−1^, *λ* = 220 nm] retention times: (major enantiomer) 42.5 min, (minor enantiomer) 28.3 min, (minor diastereomers) 19.7, 23.7 min.

#### Methyl 1-{1-(4-bromophenyl)-2-nitroethyl}-2-oxocyclopentanecarboxylate (4k)

Colorless oil; [*α*]^20^_D_ = −99.4 (*c* = 0.058, CH_2_Cl_2_); ^1^H NMR (CDCl_3_, 300 MHz) *δ* 1.85–1.99 (m, 4H), 2.34–2.44 (m, 2H), 3.75 (s, 3H), 4.03 (dd, *J* = 3.9, 11 Hz, 1H), 4.98 (dd, *J* = 11, 13.5 Hz, 1H), 5.16 (dd, *J* = 3.8, 13.7 Hz, 1H), 7.13–7.29 (m, 4H); ^13^C NMR (CDCl_3_, 100 MHz) *δ* 212.3, 169.9, 134.4, 134.1, 130.9, 129.1, 76.4, 62.4, 53.3, 45.7, 38.0, 31.4, 19.5; IR (CH_2_Cl_2_) *ν* 2956, 2920, 2892, 1754, 1562 cm^−1^; HRMS (FAB^+^) calcd for [C_15_H_17_BrNO_5_]^+^: 370.0290, found: 370.0288; HPLC [Chiralcel OD-H, hexanes/2-propanol = 97/3, flow rate = 1 mL min^−1^, *λ* = 220 nm] retention times: (major enantiomer) 38.3 min, (minor enantiomer) 28.0 min, (minor diastereomers) 21.5, 27.1 min.

#### Methyl 1-{1-(4-methoxyphenyl)-2-nitroethyl}-2-oxocyclopentane-carboxylate (4l)

Colorless oil; [*α*]^20^_D_ = −177.6 (*c* = 0.05, CH_2_Cl_2_); ^1^H NMR (CDCl_3_, 300 MHz) *δ* 1.79–2.05 (m, 4H), 2.31–2.43 (m, 2H), 3.75 (s, 3H), 3.77 (s, 3H), 4.06 (dd, *J* = 4.1, 11 Hz, 1H), 4.98 (dd, *J* = 11, 13.2 Hz, 1H), 5.11 (dd, *J* = 4.1, 13.4 Hz, 1H), 6.83 (d, *J* = 8.7 Hz, 2H), 7.17 (d, *J* = 8.7 Hz, 2H); ^13^C NMR (CDCl_3_, 100 MHz) *δ* 212.6, 170.1, 159.5, 130.6, 127.1, 114.3, 76.7, 62.8, 55.3, 53.2, 45.7, 38.2, 31.1, 19.5; IR (CH_2_Cl_2_) *ν* 2952, 1711, 1553 cm^−1^; HRMS (FAB^+^) calcd for [C_16_H_19_NO_6_]^+^: 321.1212, found: 321.1210; HPLC [Chiralcel OD-H, hexanes/2-propanol = 97/3, flow rate = 1 mL min^−1^, *λ* = 220 nm] retention times: (major enantiomer) 87.1 min, (minor enantiomer) 73.4 min, (minor diastereomers) 46.1, 57.4 min.

#### Methyl 1-{1-(2-methoxyphenyl)-2-nitroethyl}-2-oxocyclopentane-carboxylate (4m)

Colorless oil; [*α*]^20^_D_ = −17.3 (*c* = 0.04, CH_2_Cl_2_); ^1^H NMR (CDCl_3_, 300 MHz) *δ* 1.83–2.04 (m, 4H), 2.32–2.42 (m, 2H), 3.75 (s, 3H), 3.80 (s, 3H), 4.33 (dd, *J* = 2.8, 9.9 Hz, 1H), 5.13 (dd, *J* = 10.4, 13.7 Hz, 1H), 5.39 (dd, *J* = 3.6, 13.8 Hz, 1H), 6.86–6.93 (m, 2H), 7.23–7.27 (m, 2H); ^13^C NMR (CDCl_3_, 100 MHz) *δ* 231.1, 169.5, 157.8, 130.2, 129.6, 124.7, 121.2, 111.4, 76.8, 62.4, 55.7, 52.9, 38.0, 32.5, 19.2; IR (CH_2_Cl_2_) *ν* 2956, 2841, 1754, 1600, 1556 cm^−1^; LRMS (ESI) calcd for [C_16_H_19_NO_6_Na]^+^: 344.1110, found: 344.1137; HPLC [Chiralcel OJ-H, hexane/2-propanol = 95/5, flow rate = 1 mL min^−1^, *λ* = 220 nm] retention times: (major enantiomer) 45.8 min, (minor enantiomer) 51.6 min, (minor diastereomers) 54.3, 61.1 min.

#### Methyl 1-(1-nitrohexan-2-yl)-2-oxocyclopentanecarboxylate (4n)

Colorless oil; [*α*]^20^_D_ = −152.3 (*c* = 0.024, CH_2_Cl_2_); ^1^H NMR (CDCl_3_, 300 MHz) *δ* 1.29–1.99 (m, 6H), 2.01–2.06 (m, 2H), 2.25–2.37 (m, 1H), 2.40–2.49 (m, 1H), 2.58–2.61 (m, 1H), 2.63–2.85 (m, 1H), 3.71 (s, 3H), 4.38 (dd, *J* = 5.5, 14 Hz, 1H), 4.88 (dd, *J* = 5, 14.1 Hz, 1H); ^13^C NMR (CDCl_3_, 100 MHz) *δ* 213.51, 170.0, 76.5, 63.1, 53.0, 40.8, 38.4, 31.1, 30.1, 29.9, 22.7, 19.6, 14.0; IR (CH_2_Cl_2_) *ν* 2957, 2841, 1751, 1726, 1553 cm^−1^; LRMS (ESI) calcd for [C_13_H_21_NO_5_Na]^+^: 294.1317, found: 294.1352; HPLC [Chiralcel OD-H, hexane/2-propanol = 95/5, flow rate = 0.5 mL min^−1^, *λ* = 215 nm] retention times: (major enantiomer) 23.4 min, (minor enantiomer) 17.1 min, (minor diastereomers) 19.5, 20.5 min.

#### Methyl 1-(1-cyclohexyl-2-nitroethyl)-2-oxocyclopentanecarboxylate (4o)^14*d*^

Colorless oil; [*α*]^20^_D_ = −92.4 (*c* = 0.02, CH_2_Cl_2_); ^1^H NMR (CDCl_3_, 300 MHz) *δ* 0.97–1.20 (m, 6H), 1.50–1.73 (m, 5H), 2.03–2.10 (m, 3H), 2.40–2.45 (m, 2H), 2.64–2.77 (m, 2H), 3.69 (s, 3H), 4.57 (dd, *J* = 6.9, 15.1 Hz, 1H), 5.09 (dd, *J* = 3.8, 15.1 Hz, 1H); ^13^C NMR (CDCl_3_, 100 MHz) *δ* 213.1, 170.1, 74.0, 62.2, 52.9, 45.2, 39.5, 38.0, 33.0, 32.4, 28.8, 27.0, 26.7, 26.0, 19.4; IR (CH_2_Cl_2_) *ν* 2930, 2854, 1752, 1724, 1552 cm^−1^; HPLC [Chiralcel OD-H, hexane/2-propanol = 95/5, flow rate = 0.5 mL min^−1^, *λ* = 215 nm] retention times: (major enantiomer) 19.1 min, (minor enantiomer) 17.5 min, (minor diastereomers) 12.2, 13.3 min.

#### Methyl 1-(3-methyl-1-nitrobutan-2-yl)-2-oxocyclopentanecarboxylate (4p)^14*d*^

Colorless oil; [*α*]^20^_D_ = −152.3 (*c* = 0.024, CH_2_Cl_2_); ^1^H NMR (CDCl_3_, 300 MHz) *δ* 0.86 (d, *J* = 6.9 Hz, 3H), 0.94 (d, *J* = 6.9 Hz, 3H), 1.93–2.06 (m, 4H), 2.39–2.44 (m, 2H), 2.70–2.74 (m, 1H), 2.79–2.83 (m, 1H), 3.70 (s, 3H), 4.51 (dd, *J* = 6, 15.1 Hz, 1H), 5.15 (dd, *J* = 4.1, 15.1 Hz, 1H); ^13^C NMR (CDCl_3_, 100 MHz) *δ* 212.9, 169.9, 73.3, 62.3, 52.9, 45.5, 38.1, 32.4, 28.9, 22.7, 19.4, 18.0; IR (CH_2_Cl_2_) *ν* 2965, 1753, 1725, 1552 cm^−1^; HPLC [Chiralcel OD-H, hexanes/2-propanol = 95/5, flow rate = 1 mL min^−1^, *λ* = 215 nm] retention times: (major enantiomer) 11.4 min, (minor enantiomer) 10.2 min, (minor diastereomers) 19.3, 36.1 min.

#### Methyl 1-(2-nitro-1-*p*-tolylethyl)-2-oxocyclohexanecarboxylate (4q)

Slightly yellow oil; [*α*]^20^_D_ = +351.0 (*c* = 0.007, CH_2_Cl_2_); ^1^H NMR (300 MHz, CDCl_3_) *δ* 7.10–7.07 (m, 2H), 7.01–6.99 (m, 2H), 5.05 (dd, *J* = 3.3, 13.4 Hz, 1H), 4.74 (dd, *J* = 11.3, 13.2 Hz, 1H), 3.98 (dd, *J* = 3, 11.3 Hz, 1H), 3.76 (s, 3H), 2.53–2.43 (m, 2H), 2.30 (s, 3H), 2.11–1.99 (m, 2H), 1.73–1.46 (m, 4H); ^13^C NMR (100 MHz, CDCl_3_) *δ* 207.2, 170.4, 138.2, 132.2, 129.4, 129.3, 77.7, 63.2, 52.7, 47.4, 41.6, 37.1, 28.1, 22.5, 21.2; IR (CH_2_Cl_2_) *ν* 2950, 1743, 1712, 1554 cm^−1^; LRMS (ESI) calcd for [C_17_H_21_NO_5_Na]^+^: 342.1317, found: 342.1297; HPLC [Chiralcel OJ-H, hexane/2-propanol = 80/20, 0.5 mL min^−1^, *λ* = 210 nm] retention times: (major enantiomer) 41.9 min, (minor enantiomer) 25.8 min, (minor diastereomers) 23.8, 35.2 min.

#### Methyl 1-{1-(4-chlorophenyl)-2-nitroethyl}-2-oxocyclohexanecarboxylate (4r)

Colorless oil; [*α*]^20^_D_ = +61.7 (*c* = 0.018, CH_2_Cl_2_); ^1^H NMR (300 MHz, CDCl_3_) *δ* 7.25–7.28 (m, 2H), 7.09–7.11 (m, 2H), 5.02 (dd, *J* = 3.3, 13.4 Hz, 1H), 4.74 (dd, *J* = 11.2, 13.4 Hz, 1H), 3.98 (dd, *J* = 3, 11.3 Hz, 1H), 3.74 (s, 3H), 2.50–2.44 (m, 2H), 2.13–2.00 (m, 2H), 1.75–1.47 (m, 4H); ^13^C NMR (100 MHz, CDCl_3_) *δ* 206.9, 170.2, 134.3, 134.1, 130.9, 128.9, 77.4, 63.1, 52.8, 47.3, 41.6, 37.1, 28.0, 22.5; IR (CH_2_Cl_2_) *ν* 2951, 2868, 1712, 1554 cm^−1^; LRMS (ESI) calcd for [C_16_H_18_ClNO_5_Na]^+^: 362.0771, found: 362.0763; HPLC [Chiralcel OJ-H, hexanes/2-propanol = 80/20, 0.5 mL min^−1^, *λ* = 210 nm] retention times: (major enantiomer) 31.2 min, (minor enantiomer) 25.1 min.

#### Methyl 1-{1-(4-bromophenyl)-2-nitroethyl}-2-oxocyclohexanecarboxylate (4s)

Colorless oil; [*α*]^20^_D_ = +61.7 (*c* = 0.018, CH_2_Cl_2_); ^1^H NMR (300 MHz, CDCl_3_) *δ* 7.44–7.41 (m, 2H), 7.05–7.02 (m, 2H), 5.02 (dd, *J* = 3.3, 13.5 Hz, 1H), 4.74 (dd, *J* = 11.2, 13.2 Hz, 1H), 3.97 (dd, *J* = 3.3, 11.3 Hz, 1H), 3.74 (s, 3H), 2.55–2.44 (m, 2H), 2.13–2.01 (m, 2H), 1.75–1.60 (m, 4H); ^13^C NMR (100 MHz, CDCl_3_) *δ* 206.9, 170.2, 134.6, 131.8, 131.3, 122.6, 77.3, 63.0, 52.9, 47.4, 41.6, 37.1, 28.0, 22.5; IR (CH_2_Cl_2_) *ν* 2950, 1711, 1553 cm^−1^; LRMS (ESI) calcd for [C_16_H_18_BrNO_5_Na]^+^: 406.0266, found: 408.0255; HPLC [Chiralcel OJ-H, hexane/2-propanol = 80/20, 0.5 mL min^−1^, *λ* = 210 nm] retention times: (major enantiomer) 40.5 min, (minor enantiomer) 37.1 min, (minor diastereomers) 17.7, 19.7 min.

#### Methyl 1-{1-(4-methoxyphenyl)-2-nitroethyl}-2-oxocyclohexane-carboxylate (4t)

Slightly yellow oil; [*α*]^20^_D_ = +103.6 (*c* = 0.01, CH_2_Cl_2_); ^1^H NMR (300 MHz, CDCl_3_) *δ* 7.07–7.04 (m, 2H), 6.82–6.79 (m, 2H), 5.02 (dd, *J* = 3.3, 13.2 Hz, 1H), 4.74 (dd, *J* = 11.3, 12.9 Hz, 1H), 3.96 (dd, *J* = 3, 11.3 Hz, 1H), 3.77 (s, 3H), 3.74 (s, 3H), 2.50–2.42 (m, 2H), 2.14–1.99 (m, 2H), 1.74–1.51 (m, 4H); ^13^C NMR (100 MHz, CDCl_3_) *δ* 207.3, 170.4, 159.5, 130.6, 127.2, 114.0, 76.9, 63.4, 55.3, 52.7, 47.2, 41.6, 37.1, 28.1, 22.5; IR (CH_2_Cl_2_) *ν* 2952, 1711, 1553, 1514 cm^−1^; LRMS (ESI) calcd for [C_17_H_21_NO_6_Na]^+^: 358.1267, found: 358.1252; HPLC [Chiralcel OJ-H, hexanes/2-propanol = 80/20, 0.5 mL min^−1^, *λ* = 210 nm] retention times: (major enantiomer) 92.4 min, (minor enantiomer) 76.7 min, (minor diastereomers) 46.1, 57.4 min.

## Conflicts of interest

There are no conflicts to declare.

## Supplementary Material

RA-010-D0RA03550E-s001
