# Supplementary material for: Enantioselective organocatalytic Michael reactions using chiral (R,R)-1,2-diphenylethylenediamine-derived thioureas
Source: RSC Adv. 2020 Aug 27;10(53):31808–14. doi: 10.1039/d0ra03550e (PMC9056493; doi:10.1039/d0ra03550e)
Supplement: RA-010-D0RA03550E-s001 [file RA-010-D0RA03550E-s001.pdf]

Electronic Supplementary Material (ESI) for RSC Advances.
This journal is © The Royal Society of Chemistry 2020

Enantioselective Organocatalytic Michael Reactions Using Chiral (*R*,*R*)-1,2-diphenylethylenediamine-derived Thioureas

Jae Ho Shim,[a] Min Ji Lee,[a] Min Ho Lee,[a] Byeng-Seon Kim[b,]* and Deok-Chan Ha[a,]*

Laboratory of Organic Synthesis, Department of Chemistry, Research Institute for Natural Sciences, Korea University, Seoul 136-713, Korea
Department of Chemistry Education and Research Institute of Natural Science, Gyeongsang National University, Jinju 52828, Korea

Supporting Information

Table of Contents

General Information **S-2**
Synthesis of catalysts and products **S-2**
Compound Characterization Data **S-2-6**
Copy of HPLC Chromatograms **S-7-18**
- Recycling test for asymmetric Michael addition using chiral catalyst 1d (Table 1.) **S-18**
Copy of NMR Spectra **S-18-34**
Copy of MASS Spectra **S-34-46**
Computational Results of DFT Calculations for all Calculated Structures **S-46-49**

General information

IR spectra were recorded on a NICOLET 380 FT–IR spectrophotometer. Optical rotations were measured with a Rudolph Automatic polarimeter (model name: A20766 APV/6w). ^{1}H NMR spectra were recorded on a Varian Mercury 400 (400 MHz), or Varian Germini 300 (300 MHz) with TMS as an internal reference. ^{13}C NMR spectra were recorded on a Varian Mercury 400 (100 MHz) with TMS or $CDCl_3$ as an internal reference. Chiral HPLC analysis was performed on a Jasco LC-1500 Series HPLC system with a UV detector. All reactions were carried out in oven dried glassware under an argon atmosphere. Toluene (CaH_2), THF (Na, benzophenone) were dried by distillation before use.

Synthesis of catalysts (1a-n); general procedure

1. Synthesis of monoalkylated thiourea catalysts.

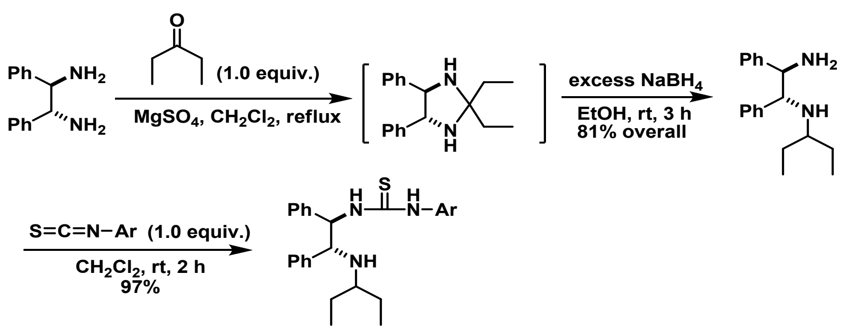

To a suspension of (*R*, *R*)-1,2-diphenylethylenediamine (1.0 equiv.) in toluene (0.1 M) was added a solution of 3-pentanone (1.1 equiv.), $MgSO_4$ and the mixture was reflux for 48h. then remove the $MgSO_4$ to celite filter and concentrated in vacuo. $NaBH_4$ (4.0 equiv.) and ethanol were added and the mixture was stirred at room temperature for 1h. quenched with 1*N* NaOH solution and the mixture was extracted with ethyl acetate. The combined organic extracts were washed with brine, dried ($MgSO_4$),and concentrated in vacuo. The product was purified by chromatography on a silica-gel column (methanol/methylene chloride 1:20). The monoalkylated DPEN (1.0 equiv.) in CH_2Cl_2 (0.1 M) was added thourea (1.1 equiv.) and the mixture was stirred at room temperature for 1h. purified by flash column chromatography on silica gel with ethyl acetate/hexanes (1:5) to give the pure amide product(quantitative yield) as a white foamy solid.

2. Synthesis of arylated thiourea catalysts.

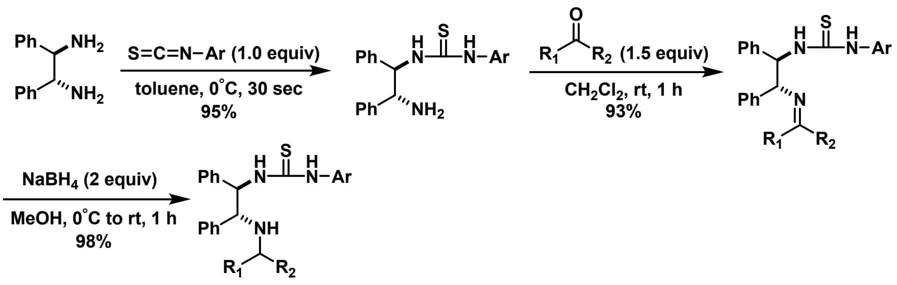

To a suspension of (*R*, *R*)-1,2-diphenylethylenediamine (1.0 equiv.) in toluene (0.5 M) was added thourea (1.0 equiv.) at 0°Cand the mixture was stirred for 30 sec. the product concentrated in vacuo and purified by flash column chromatography on silica gel with ethyl (methanol/methylene chloride 1:20). The thiourea substituted DPEN (1.0 equiv.) in CH_2Cl_2 (0.1 M) was added aryl kethone (1.1 equiv.) and the mixture was stirred at room temperature for 1h. $NaBH_4$ (2.0 equiv.) and ethanol were added at 0°Cand the mixture was stirred at room temperature for 1h. and the products celite filter and the mixture was extracted with ethyl acetate. The combined organic extracts were washed with brine, dried ($MgSO_4$) and concentrated in vacuo. The product was purified by chromatography on a silica-gel column (methanol/methylene chloride 1:20) to give the pure amide product(quantitative yield) as a brown foamy solid.

3. Synthesis of product compounds.

General Procedure of the Asymmetric Michael reaction

The β-nitrostyrene (0.4 mmol), malonate (2.0 equiv.) and 10 mol% of **1d** were added in the toluene (0.4 M) an d the reaction mixture was stirred at rt. The reaction conversion was monitored by TLC. After completion, eth yl acetate (0.2ml) was added in the reaction mixture. This solution was washed twice with water (2×1.0 mL), dried over magnesium sulfate and concentrated to yield the desired product. The product was purified by chrom atography on a silica-gel column (hexanes/ethyl acetate 5:1).

General Procedure of recycling test for the Asymmetric Michael reaction using 1d catalyst

The *β*-nitrostyrene (67 mmol), malonate (1.5 equiv.) and 5 mol% of **1d** were added in the CH_2Cl_2 (5ml) and the reaction mixture was stirred at 40 °C. The reaction conversion was monitored by TLC. After completion, CH_2Cl_2 (0.5ml) was added in the reaction mixture. This solution was washed twice with water (2×3.0 mL), dried over magnesium sulfate and concentrated to yield the desired product. The product was purified by chromatography on a silica-gel column (hexanes/ethyl acetate 5:1). Next purified by flash column chromatography on same silica gel (hexanes/ethyl acetate 1:1) to give the pure **1d** catalyst.

4. synthesis of (*R*)-phenibut

Under argon atmosphere, to the suspension of 5e (1.0 equiv, >99% ee) and $NiCl_2 \cdot 6H_2O$ (1.0 equiv) in MeOH (8.0 ml) was added $NaBH_4$ (10 equiv) at 0 °C. After the reaction mixture was stirred 7.5 h at rt, the reaction mixture was quenched with NH_4Cl and diluted with $CHCl_3$. The organic layer was separated and dried over $MgSO_4$, filtrated and concentrated in vacuo. The residue was purified by column chromatography on silica gel ($MeOH/CHCl_3$ = 1/20 as eluent) to afford desired product (98%) as colorless powder. The powder (1.0 equiv) in 6*N* HCl (2.7 ml) was refluxed at 100 °C. After 12 h, the reaction mixture was concentrated in vacuo to afford (*R*)-(–)- Phenibut (92%) as colorless solid.

Compound Characterization Data

1-[(1*R*,2*R*)-2-Amino-1,2-diphenylethyl]-3-phenylthiourea (1a)

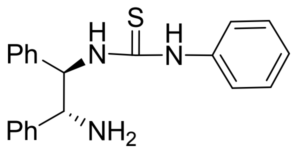

94% yield; $[\alpha]_D^{20}$ = +62.0 (c = 0.02, CH_2Cl_2); ^{1}H NMR (300 MHz, $CDCl_3$) δ 7.76 (s, 1 H), 7.54~7.19 (m, 15 H), 5.54 (s, 1 H), 4.42 (d, 1 H, J = 5 Hz), 1.35 (br s, 1 H); ^{13}C NMR (100 MHz, DMSO-d_6) δ 182.09, 134.48, 133.93, 129.89, 128.70, 128.10, 127.91, 127.15, 126.94, 126.82, 126.74, 126.23, 125.59, 125.24, 122.98, 63.07,59.09; IR (KBr) 3287.86, 3027.84, 1521.63, 1241.99, 1072.28, 939.20, 698.13 cm^{-1}; HRMS (FAB$^+$) for $C_{21}H_{22}N_3S$ $[M+H]^+$ Calcd: 348.1534, Found: 348.1530.

1-(4-Nitrophenyl)-3-[(1*R*,2*R*)-2-(pentan-3-ylamino)-1,2-diphenylethyl]thiourea (1b)

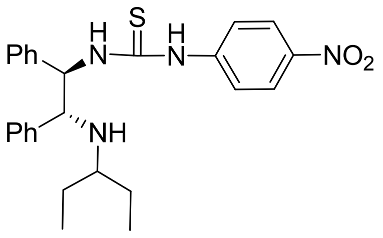

89% yield; $[\alpha]_D^{20}$ = +37.7 (c = 0.02, CH_2Cl_2); ^{1}H NMR (300 MHz, DMSO-d_6) δ 10.5 (s, 1 H), 8.16 (m, 2 H), 7.90 (d, J = 9.1 Hz, 2 H), 7.37~7.15 (m, 10 H), 5.54 (br s, 1 H), 4.16 (d, J = 5.5 Hz, 1 H), 2.07 (m, 1 H), 1.30~1.15 (m, 4 H), 0.75 (t, J = 7.4 Hz, 3 H), 0.50 (t, J = 7.4 Hz, 3 H); ^{13}C NMR (100 MHz, DMSO-d_6) δ 180.51, 146.95, 142.46, 141.92, 140.92, 128.68, 128.56, 127.72, 125.16, 120.92, 64.35, 63.80, 56.35, 55.59, 26.70, 23.96, 11.03, 8.61; IR (KBr) 3330.5, 2960.2, 2599.6, 2456.4, 2345.0, 1951.6, 1743.3, 1496.5, 1346.1, 1110.8, 1072.2, 852.4, 700.0, 586.3 cm^{-1}; HRMS (FAB$^+$) for $C_{26}H_{31}N_4O_2S$ $[M+H]^+$ Calcd: 463.2168, Found: 463.2165.

1-[(1*R*,2*R*)-2-Amino-1,2-diphenylethyl]-3-[3,5-Bis(trifluoromethyl)phenyl]thiourea (1b)

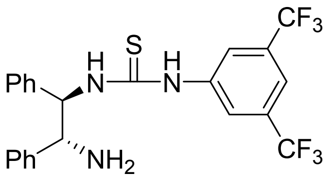

$[\alpha]_D^{25}$ +13.5 (c 1.00, CH_3Cl); ^{1}H NMR (300 MHz, DMSO-d_6) δ 10.70 (s, 1H), 8.32 (s, 2H), 7.71(s, 1H), 7.22~7.43(m, 13H), 5.57(d, J = 3 Hz, 1H), 4.44 (d, J = 3 Hz, 1H) ppm; ^{13}C NMR (100 MHz, DMSO-d_6) δ 180.80, 143.41, 142.67, 130.94, 130.62, 128.81, 128.61 127.75, 127.57, 127.51, 125.25, 122.54, 121.68, 116.40, 63.86, 60.06 ppm; IR (KBr) 3305, 3032, 2963, 1652, 1601, 1557, 1383, 1277, 1262, 803, 700 cm^{-1}; HRMS (FAB+) for $C_{23}H_{19}F_6N_3S$ [M+H]+ Calcd: 483.1282, Found: 484.1280

1-(4-Fluorophenyl)-3-[(1*R*,2*R*)-2-(pentan-3-ylamino)-1,2-diphenylethyl]thiourea (1c)

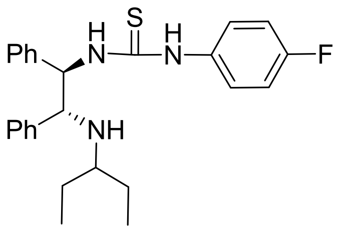

84% yield; $[\alpha]_D^{20}$ = +17.9 (c = 0.02, CH_2Cl_2); ^{1}H NMR (300 MHz, DMSO-d_6) δ 9.83 (s, 1 H), 8.00 (d, J = 6.7 Hz, 1 H), 7.48~7.43 (m, 2 H), 7.31~7.16 (m, 11 H), 5.46 (br s, 1 H), 4.09 (d, J = 5.22 Hz, 1 H), 2.03 (br s , 1 H), 1.44 (br s, 1 H), 1.14 (m, 4 H), 0.70 (t, J = 10.1, 3 H), 0.44 (t, J = 7.0 Hz, 3 H); ^{13}C NMR (100 MHz, DMSO-d_6) δ 181.42, 161.03, 158.62, 141.90, 141.44, 135.97, 128.65, 128.51, 127.57, 127.42, 126.49, 116.13, 115.90, 64.39, 63.76, 56.03, 26.72, 24.02, 10.98, 8.39; IR (KBr) 3193.7, 2962.3, 1889.9, 1511.9, 1218.8, 848.6, 701.9, 555.42 cm^{-1}; HRMS (FAB$^+$) for $C_{26}H_{31}FN_3S$$[M+H]^+$ Calcd: 436.2223, Found: 436.2201

1-[3,5-Bis(trifluoromethyl)phenyl]-3-[(1*R*,2*R*)-2-(pentan-3-ylamino)-1,2-diphenylethyl]thiourea (1d)

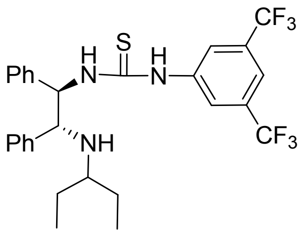

90% yield; $[\alpha]_D^{20}$ = +0.31 (c = 0.11, CH_2Cl_2); ^{1}H NMR (300 MHz, DMSO-d_6) δ 10.5 (br, 1 H), 8.30 (s, 2 H), 7.74 (s, 1 H), 7.40~7.19 (m, 10 H), 5.57 (br, 1 H), 4.18 (d, J = 4.9 Hz, 1 H), 2.09 (m, 1 H), 1.24~1.20 (m, 4 H), 0.75 (t, J = 7.1 Hz, 3 H), 0.50 (t, J = 6.0 Hz, 3 H); ^{13}C NMR (100 MHz, DMSO-d_6) δ 181.10, 142.49, 140.88, 130.96, 130.64, 128.70, 128.59, 128.56, 127.60, 125.22, 122.52, 122.19, 116.70, 64.34, 63.62, 56.48, 26.64, 23.90, 10.98, 8.54; IR (KBr) 3239.9, 2964.2, 1471.5, 1278.6, 1135.9, 885.2, 700.1 cm^{-1}; HRMS (FAB$^+$) for $C_{28}H_{30}F_6N_3S$ $[M+H]^+$ Calcd: 554.2065, Found: 554.2020

1-[(1*R*,2*R*)-2-(Pentan-3-ylamino)-1,2-diphenylethyl]-3-(*p*-tolyl)thiourea (1e)

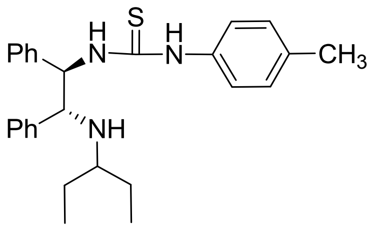

86% yield; $[\alpha]_D^{20}$ = +0.19 (c = 1.00, CH_2Cl_2); ^{1}H NMR (300 MHz, DMSO-d_6) δ 9.76 (s, 1 H), 7.89 (d, J = 7.0 Hz, 1 H), 7.32~7.18 (m, 14 H), 5.44 (s, 1 H), 4.08 (d, J = 5.1 Hz, 1 H), 2.29 (s, 2 H), 2.02 (s, 1 H), 1.39 (s, 1 H), 1.20~1.06 (m, 4 H), 0.68 (t, J = 7.5 Hz, 3 H), 0.41 (t, J = 7.1 Hz, 3 H); ^{13}C NMR (100 MHz, DMSO-d_6) δ 181.04, 141.83, 141.55, 136.71, 134.88, 130.03, 128.67, 128.51, 127.49, 127.40, 124.67, 64.28, 63.77, 55.84, 26.71, 24.02, 21.20, 10.94, 8.30; IR (KBr) 3180.2, 2958.4, 1948.8, 1510.1, 1240.1, 821.6, 700.1, 565.1 cm^{-1}; HRMS (FAB$^+$) for $C_{27}H_{34}N_3S$ $[M+H]^+$ Calcd: 432.2473, Found: 432.6537, pattern 432.5, 345.3, 266.4, 176.3, 106.01 cm^{-1}; HRMS (FAB$^+$) for $C_{27}H_{34}N_3S$ $[M+H]^+$ Calcd: 432.2473, Found: 432.2460

1-(4-Cyanophenyl)-3-[(1*R*,2*R*)-2-(pentan-3-ylamino)-1,2-diphenylethyl]thiourea (1f)

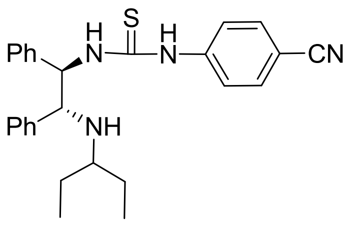

69% yield; $[\alpha]_D^{20}$ = +55.5 (c = 0.02, CH_2Cl_2); ^1H NMR (300 MHz, DMSO-d_6) δ 10.3 (br s, 1 H), 8.54 (br s, 1 H), 7.84~7.72 (m, 4 H), 7.35~7.17 (m, 10 H), 5.54 (br s, 1 H), 4.14 (d, J = 5.2 Hz, 1 H), 2.07 (br s, 1 H), 1.56 (br s, 1 H), 1.21 (m, 4H), 0.74 (t, J = 7.4 Hz, 3 H), 0.49 (t, J = 6.9 Hz, 3H); ^{13}C NMR (100 MHz, DMSO-d_6) δ 180.62, 144.83, 141.92, 141.05, 133.40, 128.67, 128.33, 127.68, 127.64, 127.51, 121.76, 119.76, 105.41, 64.41, 63.72, 60.43, 56.33, 26.74, 23.98, 21.42, 14.74, 11.02, 8.56; IR (KBr) 3317.0, 2960.2, 2360.4, 2225.5, 1949.7, 1739.5, 1508.1, 1315.2, 1176.4, 1072.2, 837.0, 700.0, 545.8 cm^{-1}; HRMS (FAB$^+$) for $C_{27}H_{31}N_4S$ $[M+H]^+$ Calcd: 443.2269, Found: 443.2279

1-[(1*R*,2*R*)-2-(Pentan-3-ylamino)-1,2-diphenylethyl]-3-[4-(trifluoromethyl) phenyl]thiourea (1g)

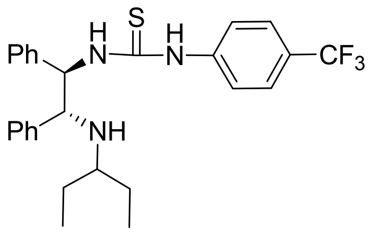

88% yield; $[\alpha]_D^{20}$ = +45.5 (c = 0.02, CH_2Cl_2); ^1H NMR (300 MHz, DMSO-d_6) δ 10.2 (br s, 1H), 8.41 (br s, 1H), 7.79 (d, J = 8.0 Hz, 2H), 7.64 (d, J = 8.5 Hz, 2H), 7.35~7.15 (m, 10H), 5.53 (br s, 1H), 4.13 (d, J = 5.5 Hz, 1H), 2.07 (m, 1H), 1.30~1.15 (m, 4H), 0.73 (t, J = 7.1 Hz, 3H), 0.49 (t, J = 6.9 Hz, 3H); ^{13}C NMR (100 MHz, DMSO-d_6) δ 180.91, 143.97, 141.18, 128.64, 128.52, 127.69, 127.48, 126.29, 122.41, 64.43, 63.71, 56.32, 26.68, 23.98, 10.98, 8.53; IR(KBr) 3205.3, 2962.3, 1945.9, 1741.5, 1517.8, 1324.9, 1245.9, 1066.5, 840.9, 700.1, 597.9 cm^{-1}; HRMS(FAB$^+$) for $C_{27}H_{31}F_3N_3S[M+H]^+$ Calcd: 486.2191, Found: 486.2163.

1-[(1*R*,2*R*)-2-(Pentan-3-ylamino)-1,2-diphenylethyl]-3-(perfluorophenyl)thiourea (1h)

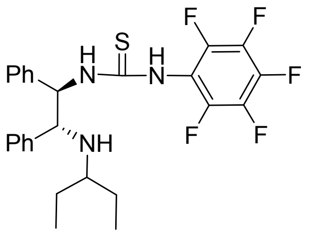

89% yield; $[\alpha]_D^{20}$ = +80.4 (c = 0.02, CH_2Cl_2); ^1H NMR (300 MHz, DMSO-d_6) δ 9.47 (s, 1 H), 8.61 (s, 1 H), 7.30~7.15 (m, 10 H), 5.48 (br s, 1 H), 4.13 (d, J = 6.1 Hz, 1 H), 2.08 (m, 1 H), 1.54 (br, 1 H), 1.30~1.14 (m, 4 H), 0.74 (t, J = 7.4 Hz, 3 H), 0.55 (t, J = 6.3 Hz, 3 H); ^{13}C NMR (100 MHz, DMSO-d_6) δ 183.63, 145.82, 143.43, 141.90, 140.77, 139.01, 138.84, 136.56, 129.39, 128.61, 128.43, 127.68, 127.60, 115.93, 64.77, 64.53, 56.37, 26.72, 24.16, 10.86, 8.58; IR (KBr) 3299.8, 2964.2, 1525.5, 1344.2, 1145.6, 991.3, 912.2, 700.1, 605.6 cm^{-1}; HRMS (FAB$^+$) for $C_{26}H_{27}F_5N_3S$ $[M+H]^+$ Calcd: 508.1846, Found: 508.1823.

1-(2,6-Difluorophenyl)-3-[(1R,2R)-2-(pentan-3-ylamino)-1,2-diphenylethyl]thiourea (1i)

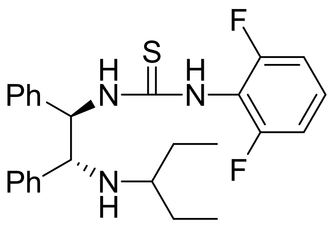

91% yield; $[\alpha]_D^{20}$ = + 391.0 (c 0.12, CH_2Cl_2); ^1H NMR (300 MHz, DMSO-d_6) δ 9.28 (s, 1 H), 8.21 (d, J = 3.57 Hz, 1 H), 7.44~7.34 (m, 2 H), 7.27~7.14 (m, 10 H), 5.43 (br, 1 H), 4.10 (d, J = 5.77 Hz, 1 H), 2.04 (br, 1H), 1.48 (br, 1H), 1.23~1.10 (m, 4H), 0.603 (t, J = 7.42 3H), 0.492 (br, 3H); IR(KBr) 3155.0, 2960.2, 2360.4, 1951.6, 1735.6, 1469.5, 1294.0, 1241.9, 1006.7, 837.0, 700.0, 572.8 cm^{-1}; HRMS(FAB$^+$) for C26H30F2$N_3S[M+H]^+$ Calcd: 454.2129, Found: 454.2133

(*R*)-Dimethyl 2-(2-nitro-1-phenylethyl)malonate (5a)

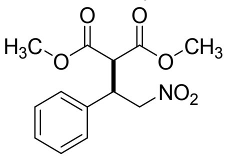

$[\alpha]_D^{20}$ = −1.98 (c 1.33, CH_2Cl_2); ^1H NMR (300 MHz, $CDCl_3$) δ 7.40~7.15 (m, 5H), 5.15~5.03 (m, 1H), 4.93 (dd, J = 4.5, 12.8 Hz, 1H), 4.88~4.76 (m, 2H), 4.20 (td, J = 4.5, 9.5 Hz, 1H), 3.76 (d, J = 9.5 Hz, 1H), 1.24 (d, J = 6.1 Hz, 3H), 1.07 (d, J = 6.4 Hz, 3H), 1.01 (d, J = 6.4 Hz, 3H) ppm; ^{13}C NMR (100 MHz, $CDCl_3$) δ 167.1, 166.4, 136.3, 128.9, 128.3, 128.2, 77.9, 69.9, 69.5, 55.1, 42.9, 21.5, 21.4, 21.19, 21.17 ppm; IR(KBr) 3030, 2985,1727, 1557 cm^{-1}; HRMS(ESI) for $C_{13}H_{16}N_1O_6[M+H]^+$ Calcd: 282.09726, Found: 282.09728; HPLC [Chiralcel AD-H, hexane/2-propanol = 95/5, 1.0 mL/min, λ = 254 nm, retention times: (major) 23.3 min, (minor) 38.0 min].

(*R*)-Diethyl 2-(2-nitro-1-phenylethyl)malonate (5b)

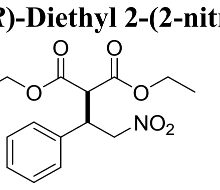

$[\alpha]_D^{20}$ = −4.61 (c 0.23, CH_2Cl_2); ^1H NMR (300 MHz, $CDCl_3$) δ 7.30~7.20 (m, 5H), 4.93 (dd, J = 4.6, 13.1 Hz, 1H), 4.86 (dd, J = 9.2, 13.1 Hz, 1H), 4.24~4.17 (m, 3H), 3.98~3.97 (q, J = 7.2 Hz, 2H), 3.81~3.79 (d, J = 9.5 Hz, 1H), 1.25 (t, J = 7.2 Hz, 3H), 1.03 (t, J = 7.2 Hz, 3H) ppm; ^{13}C NMR (100 MHz, $CDCl_3$) δ 167.4, 166. 7, 136.2, 128.8, 128.2, 127.9, 77.6, 62.0, 61.8, 54.9, 42.9, 13.9, 13.6 ppm; IR(KBr) 2989, 2938, 1731, 1557 cm^{-1}; HRMS(ESI) for $C_{15}H_{20}N_1O_6[M+H]^+$ Calcd: 310.12906, Found: 310.12936; HPLC [Chiralcel AD-H, hexane/ethanol = 90/10, 1.0 mL/min, λ = 254 nm, retention times: (major) 11.5 min, (minor) 15.3 min].

(*R*)-Diisopropyl 2-(2-nitro-1-phenylethyl)malonate (5c)

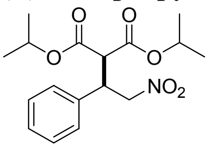

$[\alpha]_D^{20}$ = −1.24 (c 1.00, CH_2Cl_2); ^1H NMR (300 MHz, $CDCl_3$) δ 7.32~7.22 (m, 5H), 5.10 (dd, J = 5.0,13.1 Hz, 1H), 4.91~4.979 (m, 3H), 4.21~4.19 (m, 1H), 1.25 (d, J = 2.0 Hz, 6H), 1.07 (dd, J = 2.0,2.0 Hz, 6H) ppm; ^{13}C NMR (100 MHz, $CDCl_3$) δ 167.27, 166.54, 136.47, 129.07, 128.34, 127.9, 78.15, 70.15, 69.75, 55.35, 43.14, 21.80, 21.67, 21.48 ppm; IR(KBr) 3029, 2956, 1737, 1558 cm^{-1}; HRMS(ESI) for $C_{17}H_{24}N_1O_6[M+H]^+$ Calcd: 338.16036 Found: 338.10532; HPLC [Chiralcel AD-H, hexane/2-propanol = 95/5, 1.0 mL/min, λ = 254 nm, retention times: (major) 14.8 min, (minor) 34.4 min].

(*R*)-Dipropyl 2-(2-nitro-1-phenylethyl)malonate (5d)

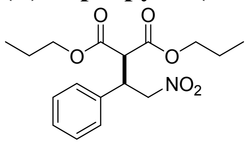

$[\alpha]_D^{20}$ = −1.73 (*c* 0.10, CH_2Cl_2); ^{1}H NMR (300 MHz, $CDCl_3$) δ 7.31~7.22 (m, 5H), 4.92~4.87 (t, *J* = 5.0,9.5 Hz, 2H), 4.24 (m, 1H), 4.15~4.09 (m, 2H), 3.92~3.83 (dd,s, *J* =6.6 9.7 Hz, 3H), 1.68~1.61 (m, 2H), 1.49~1.42 (m, 2H), 0.93~0.88 (t, *J* = 7.4,7.4 Hz, 3H), 0.82~0.77 (t, *J* = 7.4,7.4 Hz, 3H) ppm; ^{13}C NMR (100 MHz, $CDCl_3$) δ 167.79, 167.17, 136.46, 129.14, 128.52, 128.17, 77.85, 67.86, 67.65, 55.17, 43.16, 21.97, 21.81, 10.48 ppm; IR(KBr) 3029, 2956, 1737, 1558 cm^{-1}; HRMS(ESI) for $C_{17}H_{24}N_1O_6[M+H]^+$ Calcd: 338.16036 Found: 338.16336; HPLC [Chiralcel AD-H, hexane/2-propanol = 95/5, 1.0 mL/min, λ = 254 nm, retention times: (major) 18.4 min, (minor) 38.9 min].

(*R*)-dibutyl 2-(2-nitro-1-phenylethyl)malonate (5e)

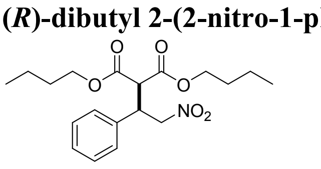

$[\alpha]_D^{20}$ = −2.55 (*c* 0.10, CH_2Cl_2); ^{1}H NMR (400 MHz, $CDCl_3$) δ 7.31~7.22 (m, 5H), 4.92~4.87 (t, *J* = 5.0,9.5 Hz, 2H), 4.24 (m, 1H), 4.15~4.09 (m, 2H), 3.92~3.83 (dd,s, *J* =6.6 9.7 Hz, 3H), 1.68~1.61 (m, 2H), 1.49~1.42 (m, 2H), 0.93~0.88 (t, *J* = 7.4,7.4 Hz, 3H), 0.82~0.77 (t, *J* = 7.4,7.4 Hz, 3H) ppm; ^{13}C NMR (100 MHz, $CDCl_3$) δ 167.79, 167.17, 136.46, 129.14, 128.52, 128.17, 77.85, 67.86, 67.65, 55.17, 43.16, 21.97, 21.81, 10.48 ppm; HRMS(EI) for $C_{19}H_{27}NO_6[M]^+$ Calcd: 365.1838 Found: 365.1830; HPLC [Chiralcel AD-H, hexane/2-propanol = 95/5, 1.0 mL/min, λ = 254 nm, retention times: (major) 18.4 min, (minor) 38.9 min].

(*R*)-Diethyl 2-[1-(4-bromophenyl)-2-nitroethyl]malonate (5f)

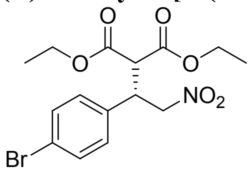

77 % yield; $[\alpha]_D^{20}$ = −3.56 (*c* 2.33, CH_2Cl_2); ^{1}H NMR (300 MHz, $CDCl_3$) δ 7.44~7.42 (d, *J* = 8.5 Hz, 2H), 7.13~7.11 (d, *J* = 8.2 Hz, 2H), 4.88~4.81 (m, 2H), 4.22~4.16 (m, 3H), 4.04~3.97 (q, *J* = 7.1,6.9 Hz, 2H), 3.78~3.75 (d, *J* = 9.4 Hz, 1H), 1.26~1.21 (t, *J* = 7.2,7.1 Hz, 3H), 1.08~1.03 (t, *J* = 7.1,7.1 Hz, 3H) ppm; ^{13}C NMR (100 MHz, $CDCl_3$) δ 167.42, 166.83, 135.52, 132.29, 130.00, 122.62, 77.55, 62.50, 62.26, 54.86, 42.60, 14.17, 13.99 ppm; IR (KBr) 2983, 2950, 1732, 1556, 1490, 1445 cm^{-1} HRMS(ESI) for $C_{15}H_{19}N_1O_6Br[M+H]^+$ Calcd: 388.03957 Found: 388.04495; HPLC [Chiralcel AD-H, hexane/ethanol = 95/5, 1.0 mL/min, λ = 254 nm, retention times: (major) 35.9 min, (minor) 44.4 min].

(*R*)-diethyl 2-(1-(4-chlorophenyl)-2-nitroethyl)malonate (5g)

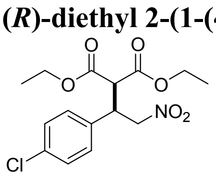

$[\alpha]_D^{20}$ = −0.24 (*c* 0.43, CH_2Cl_2); ^{1}H NMR (300 MHz, $CDCl_3$) δ 7.29~7.17 (dd, *J* = 20.6,8.2 Hz, 4H), 4.88~4.81 (m, 2H), 4.23~4.16 (m, 3H), 4.04~3.97 (q, *J* = 7.1,7.1 Hz, 2H), 3.78~3.75 (d, *J* = 9.3 Hz, 1H), 1.26~1.21 (t, *J* = 7.1,7.2 Hz, 3H), 1.08~1.03 (t, *J* = 7.2,6.8 Hz, 3H) ppm; ^{13}C NMR (100 MHz, $CDCl_3$) δ 167.44, 166.83, 134.98, 134.46, 129.69, 129.32, 77.63, 62.49, 62.23, 54.92, 42.55, 14.15, 13.97 ppm; IR (KBr) 2984, 1733, 1557, 1478, 1445, 1371 cm^{-1} HRMS(ESI) for $C_{15}H_{19}N_1O_6Cl[M+H]^+$ Calcd: 344.08954 Found: 344.09119; HPLC [Chiralcel AD-H, hexane/ethanol = 90/10, 1.0 mL/min, λ = 254 nm, retention times: (major) 17.9 min, (minor) 24.1 min].

(*R*)-Diethyl 2-[2-nitro-1-(*p*-tolyl)ethyl]malonate (5h)

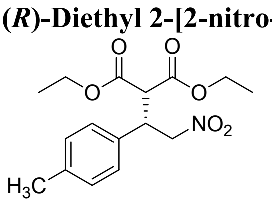

60 % yield; $[\alpha]_D^{20}$ = −1.56 (*c* 1.33, CH_2Cl_2); ^{1}H NMR (300 MHz, $CDCl_3$) δ 7.09 (d, *J* = 15.2 Hz, 4H), 4.89~4.78 (m, 2H), 4.22~4.14 (m, 3H), 4.01~3.96 (q, *J* = 7.0,7.1 Hz, 2H), 3.79 (d, *J* = 9.3 Hz, 1H), 2.27 (s, 3H), 1.25~1.22 (t, *J* = 7.1,7.0 Hz, 3H), 1.06~1.02 (t, *J* = 7.1,8.6 Hz, 3H) ppm; ^{13}C NMR (100 MHz, $CDCl_3$) δ 167.74, 167.08, 138.27, 138.23, 133.30, 129.80, 128.05, 78.00, 62.32, 62.06, 55.24, 42.84, 21.28, 14.18, 13.97 ppm; IR(KBr) 3030, 2987, 1732, 1612, 1557 cm^{-1}; HRMS(ESI) for $C_{16}H_{22}N_1O_6[M+H]^+$ Calcd: 324.14416 Found: 324.14648; HPLC [Chiralcel AD-H, hexane/ethanol = 98/2, 1.0 mL/min, λ = 254 nm, retention times: (major) 36.0 min, (minor) 42.8 min].

(*R*)-Diethyl 2-[1-(4-methoxyphenyl)-2-nitroethyl]malonate (5i)

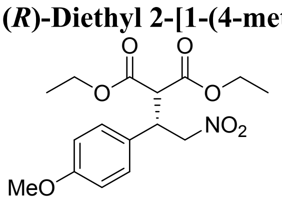

47 % yield; $[\alpha]_D^{20}$ = −1.37 (*c* 0.80, CH_2Cl_2); ^{1}H NMR (300 MHz, $CDCl_3$) δ 7.16~7.13 (d, *J* = 8.5 Hz, 2H), 6.84~6.81 (d, *J* = 8.8 Hz, 2H), 4.87~4.80 (m, 2H), 4.24~4.16 (m, 3H), 4.04~3.97 (q, *J* = 7.1,7.1 Hz, 2H), 3.79~3.78 (d, *J* = 2.7 Hz, 1H), 3.76 (s, 3H), 1.28~1.23 (t, *J* = 7.1,7.2 Hz, 3H), 1.08~1.03 (t, *J* = 7.1,7.1 Hz, 3H) ppm; ^{13}C NMR (100 MHz, $CDCl_3$) δ 167.73, 167.08, 159.61, 129.36, 128.17, 114.48, 78.12, 62.34, 62.06, 55.42, 55.30, 42.53, 14.19, 14.01 ppm; IR(KBr) 2988, 2936, 2904, 1730, 1612, 1552 cm^{-1} HRMS(ESI) for $C_{16}H_{22}N_1O_7[M+H]^+$ Calcd: 340.13908 Found: 340.13901; HPLC [Chiralcel AD-H, hexane/ethanol = 90/10, 1.0 mL/min, λ = 254 nm, retention times: (major) 23.8 min, (minor) 39.5 min].

(*R*)-Diethyl 2-[1-(2-methoxyphenyl)-2-nitroethyl]malonate (5j)

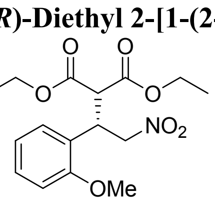

51 % yield; $[\alpha]_D^{20}$ = −7.08 (*c* 1.30, CH_2Cl_2); ^{1}H NMR (300 MHz, $CDCl_3$) δ 7.26~7.21(m, 1H), 7.15~7.12 (m, 1H), 6.87~6.83 (m, 2H), 5.06~4.98 (dd, *J* = 3.6,1.1 Hz, 1H), 4.89~4.83 (dd, *J* = 3.6,1.1 Hz, 1H), 4.37~4.34 (m, 1H), 4.24~4.12 (m, 3H), 3.97~3.90 (q, *J* = 7.2,7.2 Hz, 2H), 3.85 (s, 3H), 1.28~1.23 (t, *J* = 7.1,6.9 Hz, 3H), 1.01~0.96 (t, *J* = 7.2,7.1 Hz, 3H) ppm; ^{13}C NMR (100 MHz, $CDCl_3$) δ 168.14, 167.41, 157.62, 131.09, 129.83, 123.87, 120.96, 111.27, 76.40, 62.18, 61.77, 55.62, 52.89, 40.74, 14.20,

13.94 ppm; IR (KBr) 2984, 2939, 2908, 1732, 1613, 1556 cm^{-1} HRMS(ESI) for $C_{16}H_{22}N_1O_6$[M] Calcd: 339.13125 Found: 339.12933; HPLC [Chiralcel AD-H, hexane/2-propanol = 95/5, 1.0 mL/min, λ = 254 nm, retention times: (major) 14.9 min, (minor) 20.6 min].

(*R*)-Diethyl 2-[1-(4-hydroxyphenyl)-2-nitroethyl]malonate (5k)

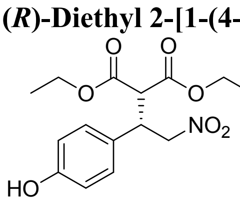

40 % yield; $[\alpha]_D^{20}$ = −1.56 (*c* = 0.50, CH_2Cl_2); ^1H NMR (300 MHz, $CDCl_3$) δ 7.09~7.06 (d, *J* = 8.3 Hz, 2H), 6.72~6.70 (d, *J* = 8.2 Hz, 2H), 5.63 (br, 1H), 4.91~4.74 (m, 2H), 4.25~4.12 (m, 3H), 4.05~3.98 (q, *J* = 7.1,6.8 Hz, 2H), 3.79 (d, *J* = 9.7 Hz, 1H), 1.29~1.24 (t, *J* = 7.1,6.6 Hz, 3H), 1.09~1.05 (t, *J* = 7.1,7.2 Hz, 3H) ppm; ^{13}C NMR (100 MHz, $CDCl_3$) δ 167.74, 167.28, 155.88, 129.54, 128.06, 78.17, 62.44, 62.23, 55.29, 42.53, 29.92, 14.20, 14.00 ppm; HRMS(ESI) for $C_{15}H_{20}N_1O_7$$[M+H]^+$ Calcd: 326.12343 Found: 326.12903; HPLC [Chiralcel AD-H, hexane/ethanol = 90/10, 1.0 mL/min, λ = 254 nm, retention times: (major) 20.4 min, (minor) 50.6 min].

(*R*)-Diethyl 2-[1-(furan-2-yl)-2-nitroethyl]malonate (5l)

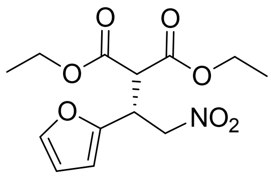

78 % yield; $[\alpha]_D^{20}$ = +5.06 (*c* 0.33, CH_2Cl_2); ^1H NMR (300 MHz, $CDCl_3$) δ 7.34~7.26 (d, *J* = 23.9 Hz, 1H), 6.29~6.28 (t, *J* = 2.9,1.6 Hz, 1H), 6.22~6.21 (d, *J* = 3.0 Hz, 1H), 4.91~4.88 (m, 2H), 4.39~4.37 (m, 1H), 4.25~4.11 (m, 4H), 3.91~3.88 (d, *J* = 7.9 Hz, 1H), 1.28~1.23 (t, *J* = 7.1,6.9 Hz, 3H), 1.22~1.17 (t, *J* = 7.1,6.9 Hz, 3H) ppm; ^{13}C NMR (100 MHz, $CDCl_3$) δ 167.35, 167.04, 149.74, 142.95, 110.74, 108.67, 75.64, 62.38, 53.20, 37.03, 29.92, 14.17, 14.11 ppm; IR (KBr) 2985, 2940, 1734,1559, 1506, 1466, 1448**;** HRMS(ESI) for $C_{13}H_{18}N_1O_7$$[M+H]^+$ Calcd: 300.10778 Found: 300.10742; HPLC [Chiralcel AD-H, hexane/2-propanol = 95/5, 0.6 mL/min, λ = 254 nm, retention times: (major) 22.7 min, (minor) 29.2 min].

(*R*)-4-amino-3-phenylbutanoic acid (6a)

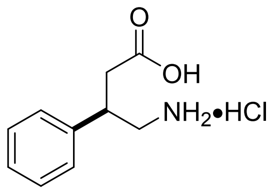

$[\alpha]_D^{20}$ = + 3.12 (*c* 2.33, MeOH); ^1H NMR (400 MHz, D_2O) δ 7.27~7.19 (m, 5H), 3.21 (m, 2H), 3.11~3.08 (d, 1H), 2.69 (dd, 1H, J = 16.0, 6.0 Hz), 2.59~2.55 (dd, 1H, J = 16.5, 8.5 Hz) ppm; ^{13}C NMR (100 MHz, DMSO-d_6) δ 175.29, 138.61, 129.57, 128.11, 44.10, 39.94, 38.35 ppm; HRMS(ESI^+) for $C_9H_{12}ClNO_2$$[M-HCl]^+$ Calcd: 180.10, Found: 180.20.

Copy of HPLC Chromatograms

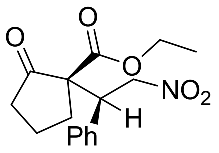

HPLC [Chiralpak OD-H column, hexanes/2-propanol = 93/7, flow rate = 1 mL/min, λ = 220 nm] retention times: (major enantiomer) = 19.4 min, (minor enantiomer) = 13.2 min, (minor diastereomers) 11.3, 15.7 min.

Racemic mixture

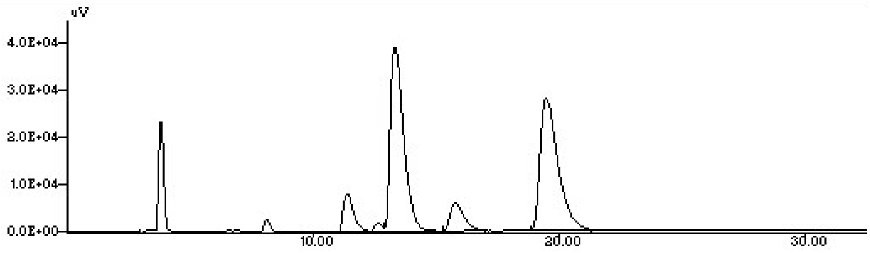

#	Name	RT	Area[uV.Sec]	Quantity
1		11.350	195373.080	0.000
2		13.292	1312951.365	0.000
3		15.750	206874.000	0.000
4		19.450	1310257.006	0.000

Total Area of Peak = 3025455.450 [uV.Sec]

Table 1-1

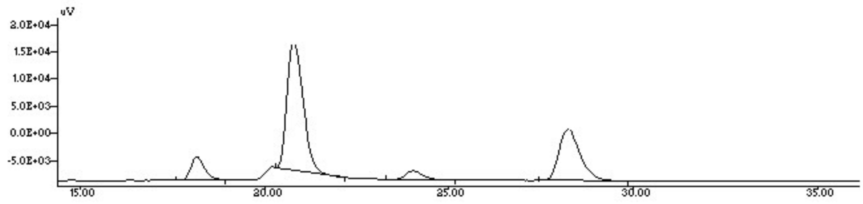

#	Name	RT	Area[uV.Sec]	Quantity
1		18.167	107469.690	0.000
2		20.767	679283.480	0.000
3		24.017	51497.500	0.000
4		28.217	376120.967	0.000

Total Area of Peak = 1214371.637 [uV.Sec]

Table 1-2

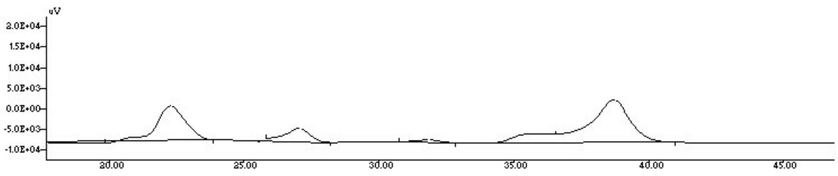

#	Name	RT	Area[uV.Sec]	Quantity
1		22.275	613910.291	0.000
2		27.033	209671.207	0.000
3		31.808	38249.000	0.000
4		38.675	1057066.760	0.000

Total Area of Peak = 1918897.258 [uV.Sec]

Table 1-3

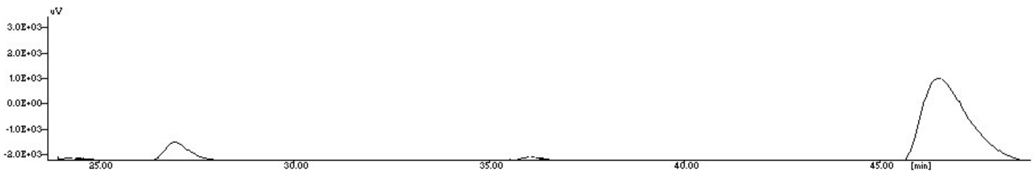

#	Name	RT	Area[uV.Sec]	Quantity
1		24.350	1681.528	0.000
2		26.933	30405.500	0.000
3		35.992	4159.697	0.000
4		46.508	277753.500	0.000

Total Area of Peak = 314000.225 [uV.Sec]

Table 1-4

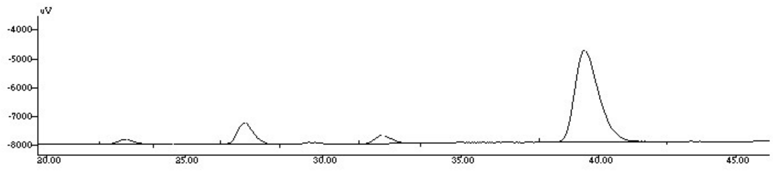

#	Name	RT	Area[uV.Sec]	Quantity
1		22.658	6702.667	0.000
2		26.817	28523.927	0.000
3		31.550	11934.529	0.000
4		38.567	183550.441	0.000

Total Area of Peak = 230711.564 [uV.Sec]

Table 1-5

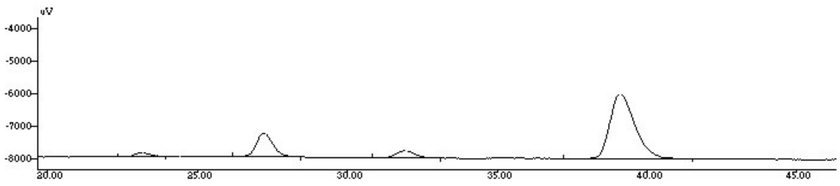

#	Name	RT	Area[uV.Sec]	Quantity
1		23.100	3527.582	0.000
2		27.225	27211.397	0.000
3		31.933	9354.426	0.000
4		39.100	115476.894	0.000

Total Area of Peak = 155570.299 [uV.Sec]

Table 1-6

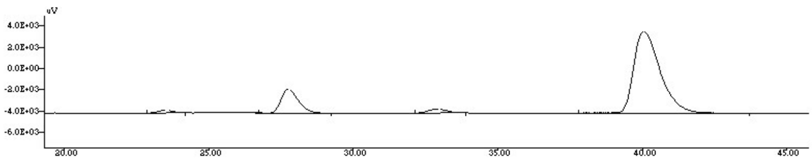

#	Name	RT	Area[uV.Sec]	Quantity
1		23.492	9057.771	0.000
2		27.750	90877.987	0.000
3		32.917	16670.317	0.000
4		40.017	475406.545	0.000

Total Area of Peak = 592012.620 [uV.Sec]

Table 1-7

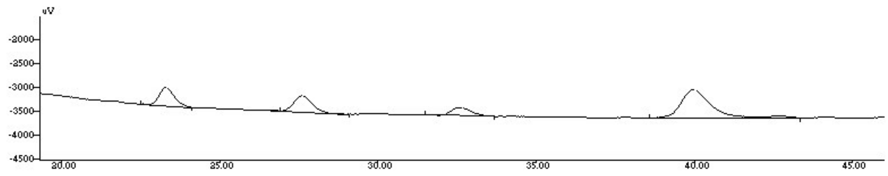

#	Name	RT	Area[uV.Sec]	Quantity
1		23.300	12629.394	0.000
2		27.600	13772.279	0.000
3		32.592	6729.778	0.000
4		39.975	40892.005	0.000

Total Area of Peak = 74023.456 [uV.Sec]

Table 1-8

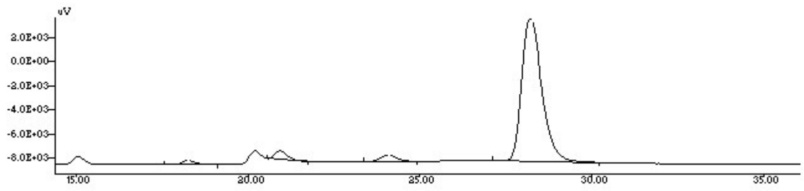

#	Name	RT	Area[uV.Sec]	Quantity
1		18.225	8143.243	0.000
2		20.933	17234.385	0.000
3		24.058	17373.441	0.000
4		28.192	473902.584	0.000

Total Area of Peak = 516653.653 [uV.Sec]

Table 1-9

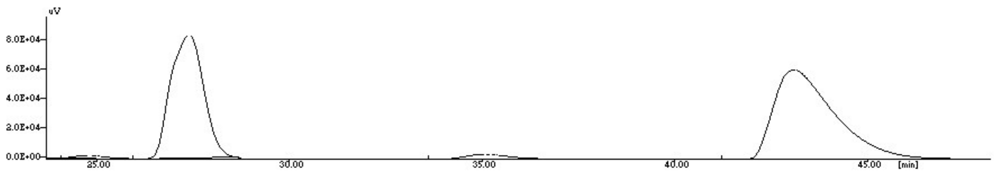

#	Name	RT	Area[uV.Sec]	Quantity
1		24.767	113410.757	0.000
2		27.383	4790171.109	0.000
3		35.050	238650.438	0.000
4		43.058	6496952.627	0.000

Total Area of Peak = 11639184.931 [uV.Sec]

Table 1-10

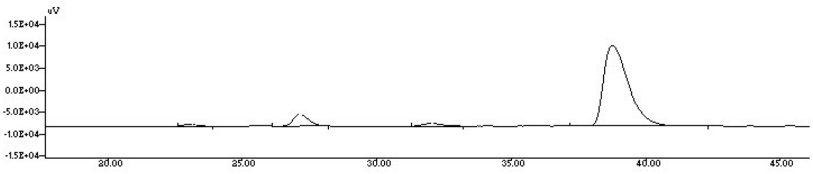

#	Name	RT	Area[uV.Sec]	Quantity
1		23.033	15540.833	0.000
2		27.133	106738.827	0.000
3		32.008	30123.500	0.000
4		38.750	1146387.500	0.000

Total Area of Peak = 1298790.660 [uV.Sec]

Table 1-11

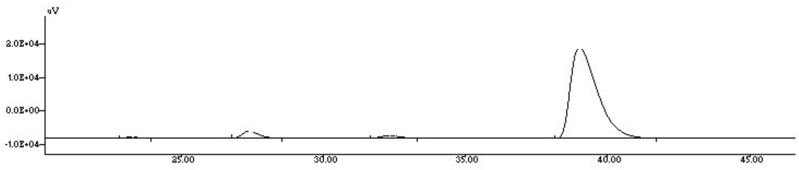

#	Name	RT	Area[uV.Sec]	Quantity
1		23.258	11272.750	0.000
2		27.400	80813.000	0.000
3		32.325	34228.500	0.000
4		39.000	1715774.000	0.000

Total Area of Peak = 1842088.250 [uV.Sec]

Table 2-1

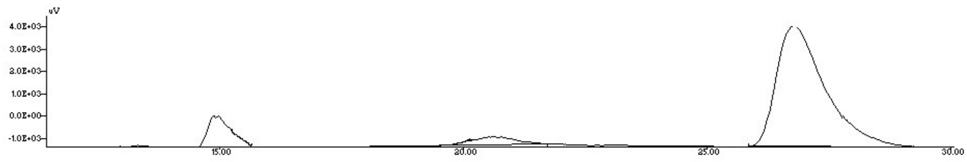

#	Name	RT	Area[uV.Sec]	Quantity
1		12.875	3464.500	0.000
2		15.400	64993.571	0.000
3		18.558	14985.613	0.000
4		23.142	278168.000	0.000

Total Area of Peak = 361611.683 [uV.Sec]

Table 2-2

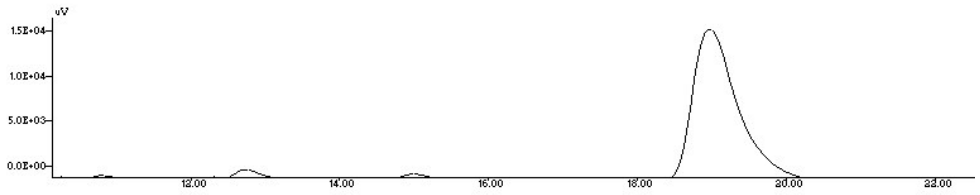

#	Name	RT	Area[uV.Sec]	Quantity
1		10.800	10446.500	0.000
2		12.733	33284.986	0.000
3		14.958	20855.099	0.000
4		18.958	708711.500	0.000

Total Area of Peak = 773298.085 [uV.Sec]

Table 2-3

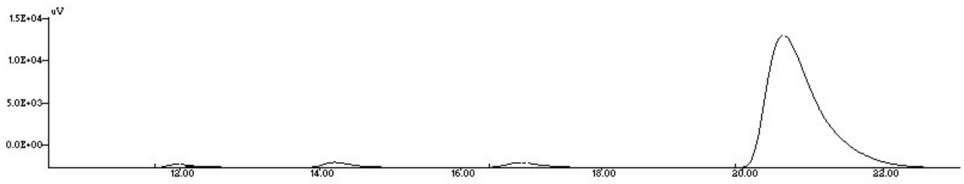

#	Name	RT	Area[uV.Sec]	Quantity
1		11.967	9032.000	0.000
2		14.192	17037.750	0.000
3		16.833	18151.500	0.000
4		20.575	773422.000	0.000

Total Area of Peak = 817643.250 [uV.Sec]

Table 2-4

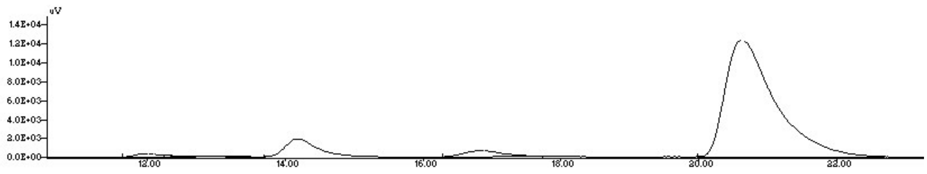

#	Name	RT	Area[uV.Sec]	Quantity
1		11.983	7555.750	0.000
2		14.175	53969.500	0.000
3		16.850	23893.500	0.000
4		20.608	599360.750	0.000

Total Area of Peak = 684779.500 [uV.Sec]

Table 2-5

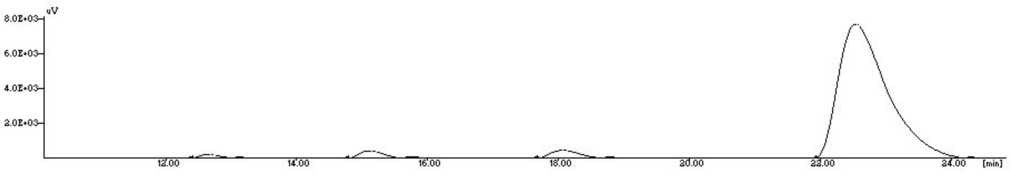

#	Name	RT	Area[uV.Sec]	Quantity
1		12.658	5720.820	0.000
2		15.117	10307.920	0.000
3		18.042	13619.910	0.000
4		22.542	396788.250	0.000

Total Area of Peak = 426436.901 [uV.Sec]

Table 2-6

#	Name	RT	Area[uV.Sec]	Quantity
1		8.075	999079.308	0.000
2		10.800	21685612.295	0.000
3		11.450	14579985.667	0.000
4		19.925	579822.500	0.000

Total Area of Peak = 37844499.769 [uV.Sec]

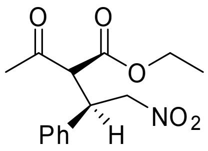

HPLC [Chiralcel AD-H, hexane/2-propanol = 80/20, 0.8 mL/min, λ = 210 nm, retention times: (major diastereomer) 10.0, 11.4 min, (minor diastereomer) 8.0, 19.9 min].

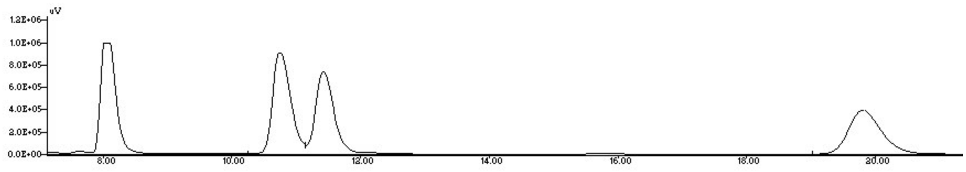

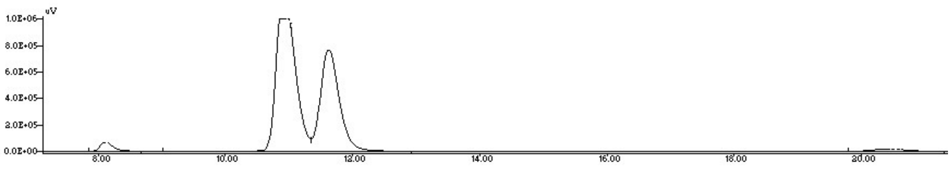

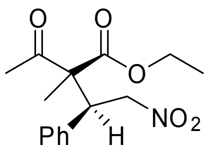

#	Name	RT	Area[uV.Sec]	Quantity
1		25.775	21847099.500	0.000
2		33.400	4525516.000	0.000
3		41.133	21951201.500	0.000
4		52.708	4436148.000	0.000

Total Area of Peak = 52759965.000 [uV.Sec]

HPLC [Chiralcel OD-H, hexane/2-propanol = 90/10, 0.5 mL/min, λ = 210 nm, retention times: (major diastereomer) 41.1, 52.7 min, (minor diastereomer) 25.7, 33.4 min].

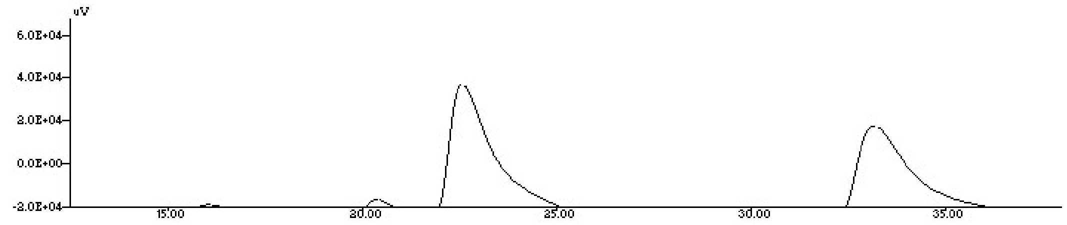

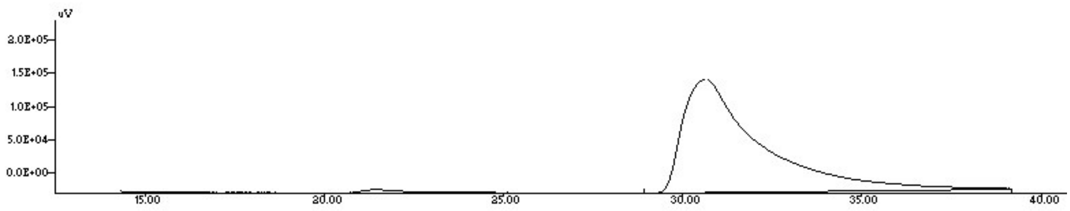

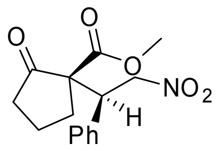

#	Name	RT	Area[uV.Sec]	Quantity
1		15.742	306537.004	0.000
2		18.517	997411.375	0.000
3		23.392	216029.330	0.000
4		28.858	871957.569	0.000

Total Area of Peak = 2391935.278 [uV.Sec]

HPLC [Chiralcel OD-H, hexane/2-propanol = 93/7, 0.5 mL/min, λ = 210 nm, retention times: (major enantiomer) 28.8 min, (minor enantiomer) 23.3 min, (minor diastereomers) 15.7, 18.5 min].

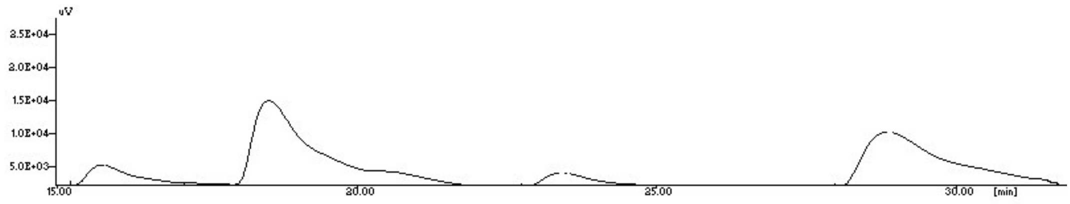

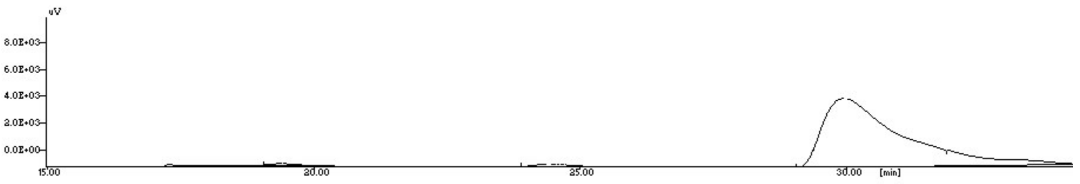

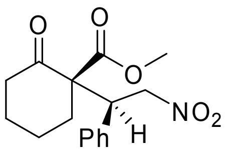

#	Name	RT	Area[uV.Sec]	Quantity
1		45.617	26882150.251	0.000
2		50.875	23058528.250	0.000
3		71.742	4202070.500	0.000
4		94.417	3951120.871	0.000

Total Area of Peak = 58093869.871 [uV.Sec]

HPLC [Chiralcel OJ-H, hexane/2-propanol = 80/20, 0.5 mL/min, λ = 210 nm, retention times: (major enantiomer) 50.8 min, (minor enantiomer) 45.6 min, (minor diastereomers) 71.7, 94.4 min].

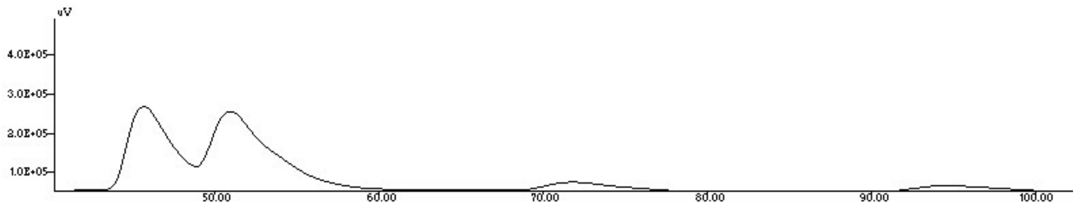

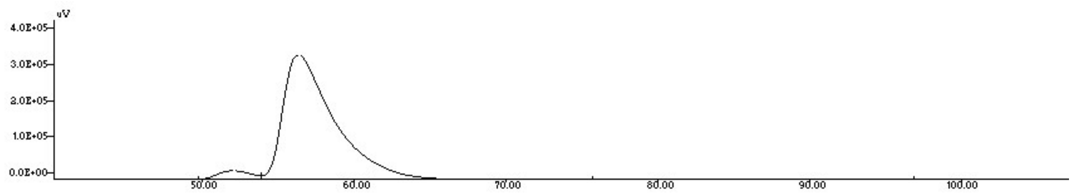

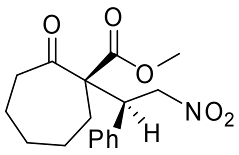

#	Name	RT	Area[uV.Sec]	Quantity
1		17.375	1125496.998	0.000
2		18.717	32770035.805	0.000
3		20.400	755597.780	0.000
4		22.258	1629143.717	0.000

Total Area of Peak = 36280274.300 [uV.Sec]

HPLC [Chiralcel AD-H, hexane/2-propanol = 90/10, 0.5 mL/min, λ = 210 nm, retention times: (major enantiomer) 18.7 min, (minor enantiomer) 22.2 min, (minor diastereomers) 17.3, 20.4 min].

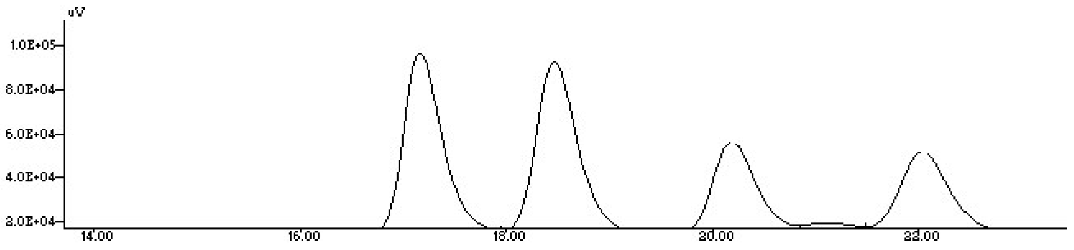

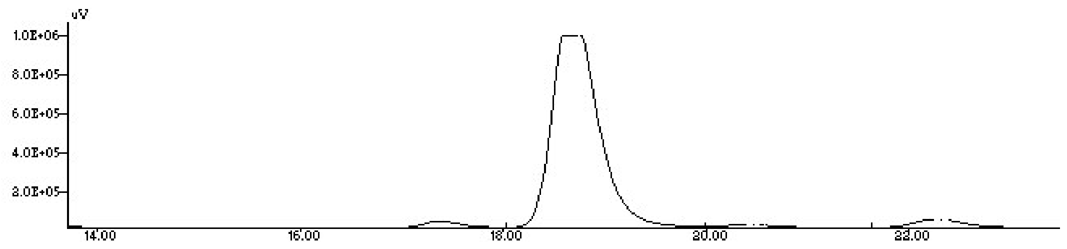

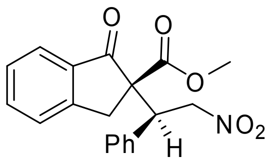

#	Name	RT	Area[uV.Sec]	Quantity
1		14.758	23687076.790	0.000
2		17.067	12451976.710	0.000
3		37.392	51315109.000	0.000
4		68.642	256725602.500	0.000

Total Area of Peak = 344179765.000 [uV.Sec]

HPLC [Chiralcel OD-H, hexane/2-propanol = 90/10, 1 mL/min, λ = 210 nm, retention times: (major enantiomer) 68.6 min, (minor enantiomer) 14.7 min, (minor diastereomers) 17.0, 37.4 min].

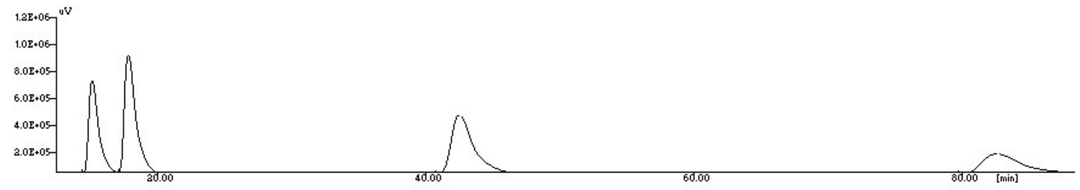

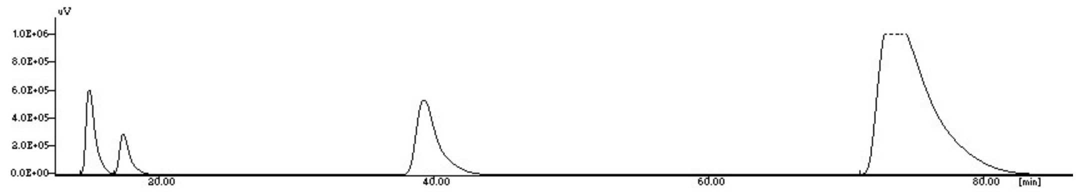

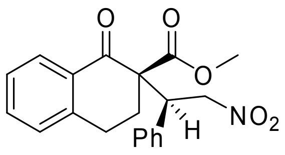

#	Name	RT	Area[uV.Sec]	Quantity
1		26.600	2068457.000	0.000
2		34.483	2050302.290	0.000
3		42.175	69383439.750	0.000
4		54.225	5310285.500	0.000

Total Area of Peak = 78812484.541 [uV.Sec]

HPLC [Chiralcel OD-H, hexane/2-propanol = 90/10, 0.5 mL/min, λ = 254 nm, retention times: (major enantiomer) 42.1 min, (minor enantiomer) 26.6 min, (minor diastereomers) 34.4, 54.2 min].

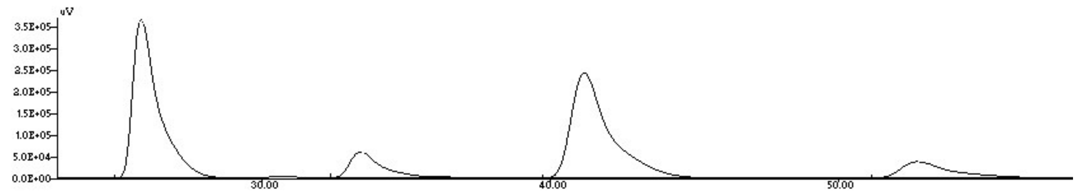

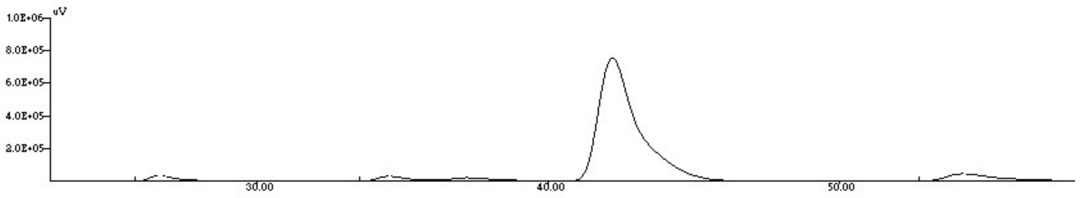

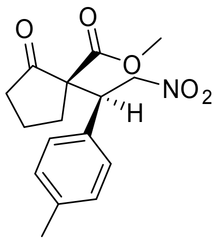

#	Name	RT	Area[uV.Sec]	Quantity
1		13.725	3601024.709	0.000
2		16.492	3829099.826	0.000
3		18.042	2579399.430	0.000
4		25.117	20101765.874	0.000

Total Area of Peak = 30111289.838 [uV.Sec]

HPLC [Chiralcel OD-H, hexane/2-propanol = 97/3, flow rate = 1 mL/min, λ = 220 nm: retention times: (major enantiomer) 25.1 min, (minor enantiomer) 16.4 min, (minor diastereomers) 13.7, 18.0 min].

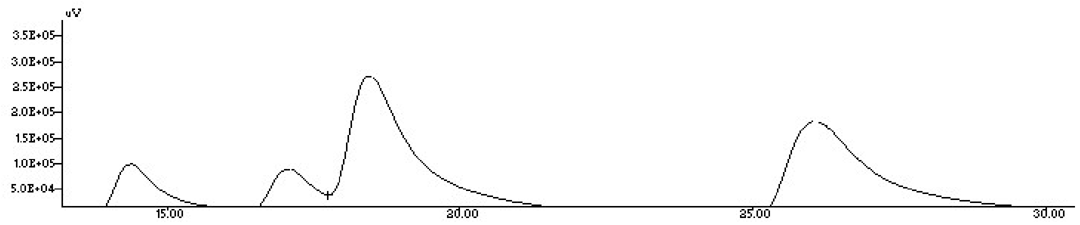

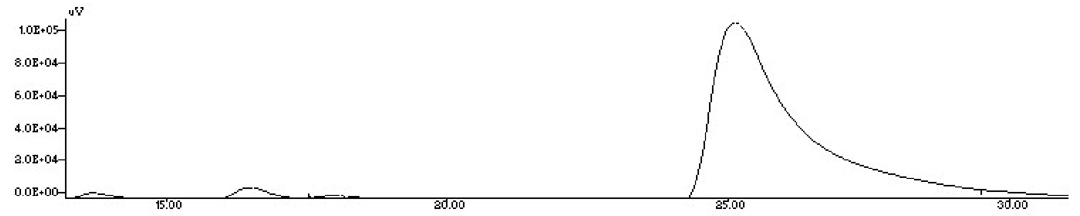

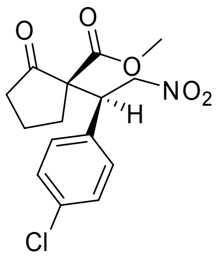

#	Name	RT	Area[uV.Sec]	Quantity
1		19.758	63514.816	0.000
2		23.767	50440.492	0.000
3		28.342	132361.135	0.000
4		42.500	3650280.255	0.000

Total Area of Peak = 3896596.698 [uV.Sec]

HPLC [Chiralcel OD-H, hexane/2-propanol = 97/3, flow rate = 1 mL/min, λ = 220 nm: retention times: (major enantiomer) 42.5 min, (minor enantiomer) 28.3 min, (minor diastereomers) 19.7, 23.7 min].

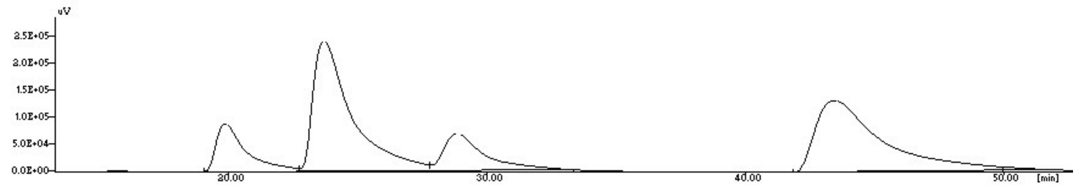

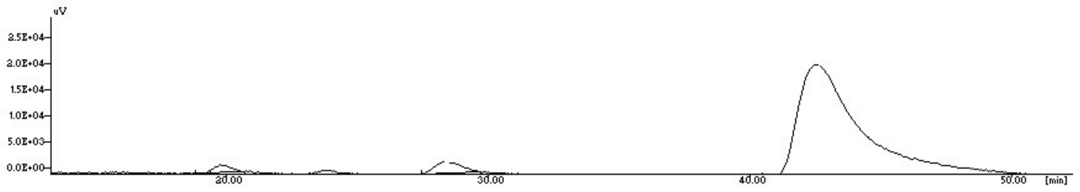

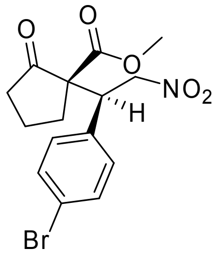

#	Name	RT	Area[uV.Sec]	Quantity
1		21.558	102612.000	0.000
2		27.100	51384.339	0.000
3		28.067	232165.719	0.000
4		38.333	8224147.000	0.000

Total Area of Peak = 8610309.058 [uV.Sec]

HPLC [Chiralcel OD-H, hexane/2-propanol = 97/3, flow rate = 1 mL/min, λ = 220 nm: retention times: (major enantiomer) 38.3 min, (minor enantiomer) 28.0 min, (minor diastereomers) 21.5, 27.1 min].

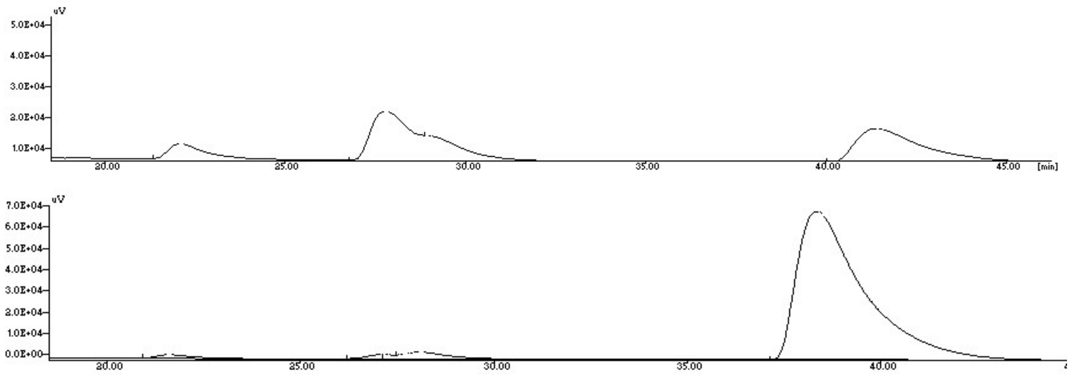

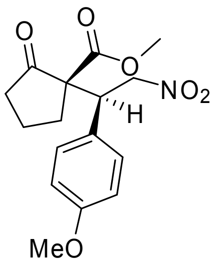

#	Name	RT	Area[uV.Sec]	Quantity
1		29.558	2458846.250	0.000
2		35.608	5248699.500	0.000
3		41.392	2549363.000	0.000
4		47.833	99354544.500	0.000

Total Area of Peak = 109611453.250 [uV.Sec]

HPLC [Chiralcel OD-H, hexane/2-propanol = 97/3, flow rate = 1 mL/min, λ = 220 nm: retention times: (major enantiomer) 47.8 min, (minor enantiomer) 35.6 min, (minor diastereomers) 29.5, 41.3 min].

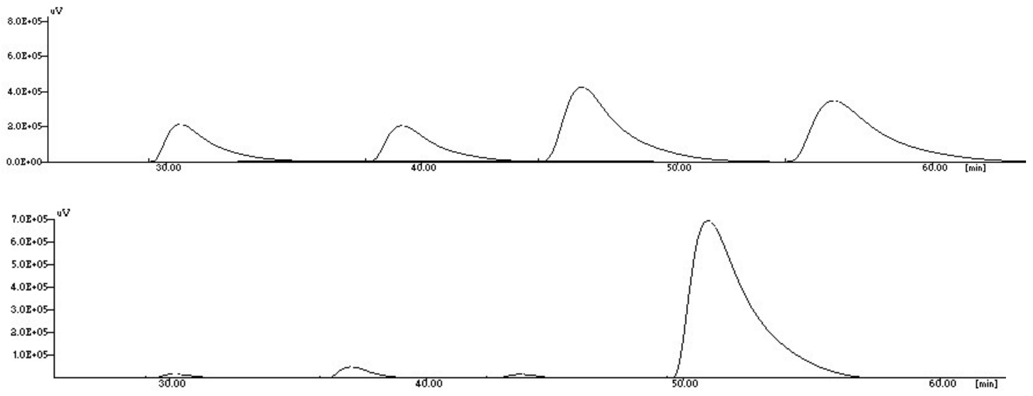

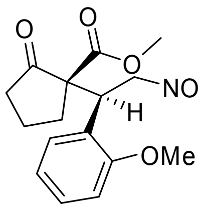

#	Name	RT	Area[uV.Sec]	Quantity
1		45.842	23268493.702	0.000
2		51.633	8688388.588	0.000
3		54.383	13760978.045	0.000
4		61.158	24242080.942	0.000

Total Area of Peak = 69959941.278 [uV.Sec]

HPLC [Chiralcel OJ-H, hexane/2-propanol = 95/5, flow rate = 1 mL/min, λ = 220 nm: retention times: (major enantiomer) 45.8 min, (minor enantiomer) 51.6 min, (minor diastereomers) 54.3, 61.1 min].

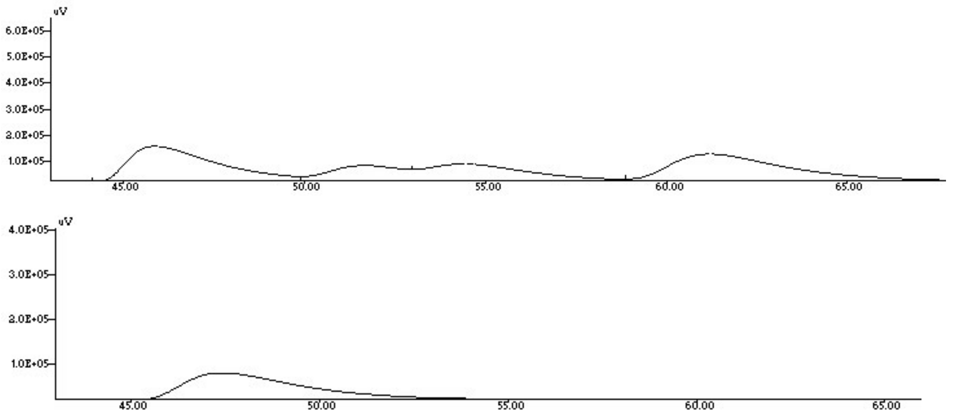

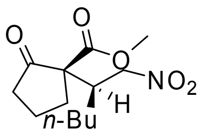

#	Name	RT	Area[uV.Sec]	Quantity
1		17.125	138674.097	0.000
2		19.533	34286.500	0.000
3		20.550	25535.500	0.000
4		23.467	11243377.000	0.000

Total Area of Peak = 11441873.097 [uV.Sec]

HPLC [Chiralcel OD-H, hexane/2-propanol = 95/5, flow rate = 0.5 mL/min, λ = 215 nm: retention times: (major enantiomer) 23.4 min, (minor enantiomer) 17.1 min, (minor diastereomers) 19.5, 20.5 min].

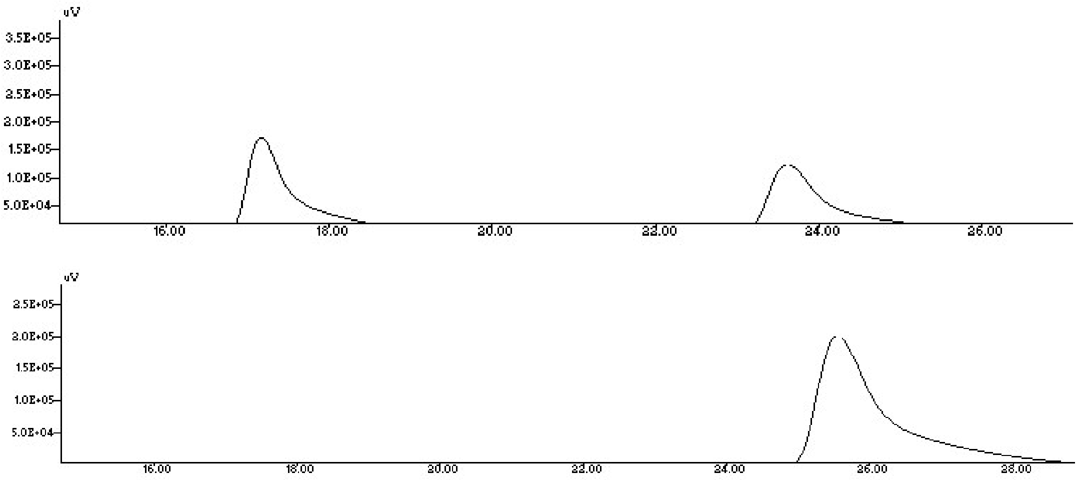

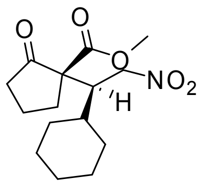

#	Name	RT	Area[uV.Sec]	Quantity
1		12.258	234762.862	0.000
2		12.683	233306.982	0.000
3		17.300	15429728.384	0.000
4		19.192	19723716.998	0.000

Total Area of Peak = 35621515.226 [uV.Sec]

HPLC [Chiralcel OD-H, hexane/2-propanol = 95/5, flow rate = 0.5 mL/min, λ = 215 nm: retention times: (major enantiomer) 19.1 min, (minor enantiomer) 17.3 min, (minor diastereomers) 12.2, 12.6 min].

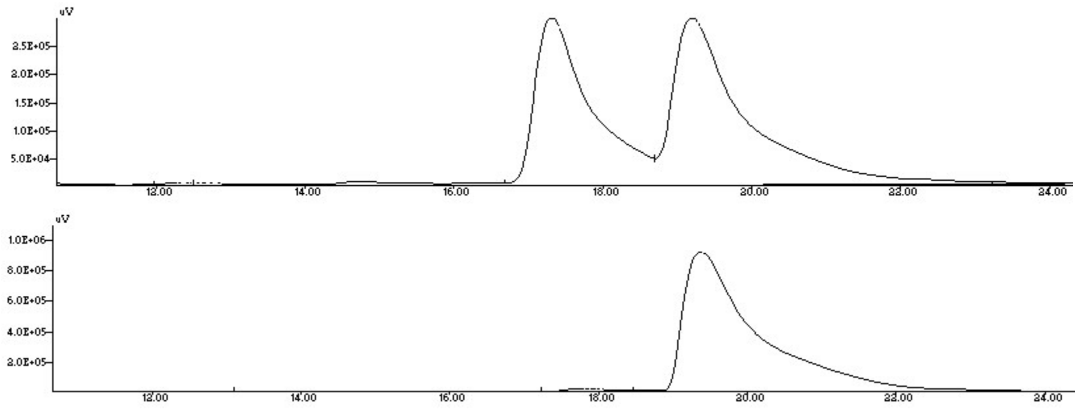

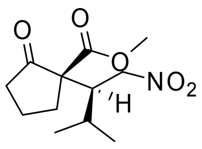

#	Name	RT	Area[uV.Sec]	Quantity
1		10.283	181057.925	0.000
2		11.417	11934421.750	0.000

Total Area of Peak = 12115479.675 [uV.Sec]

HPLC [Chiralcel OD-H, hexane/2-propanol = 95/5, flow rate = 1 mL/min, λ = 215 nm: retention times: (major enantiomer) 11.4 min, (minor enantiomer) 10.2 min, (minor diastereomers) 19.3, 36.1 min].

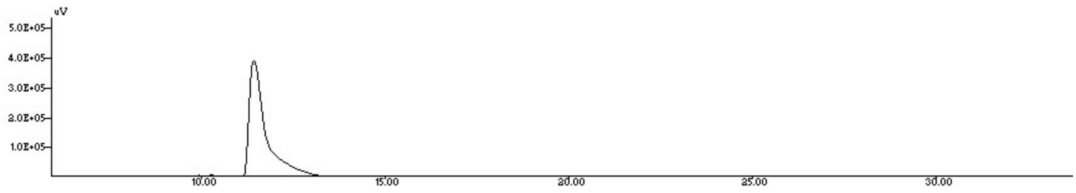

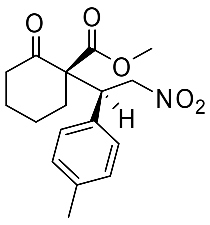

#	Name	RT	Area[uV.Sec]	Quantity
1		23.875	707145.501	0.000
2		25.892	668944.701	0.000
3		35.267	13748602.776	0.000
4		41.950	12519083.891	0.000

Total Area of Peak = 27643776.869 [uV.Sec]

HPLC [Chiralcel OJ-H, hexane/2-propanol = 80/20, 0.5 mL/min, λ = 210 nm, retention times: (major enantiomer) 41.9 min, (minor enantiomer) 25.8 min, (minor diastereomers) 35.2, 23.8 min].

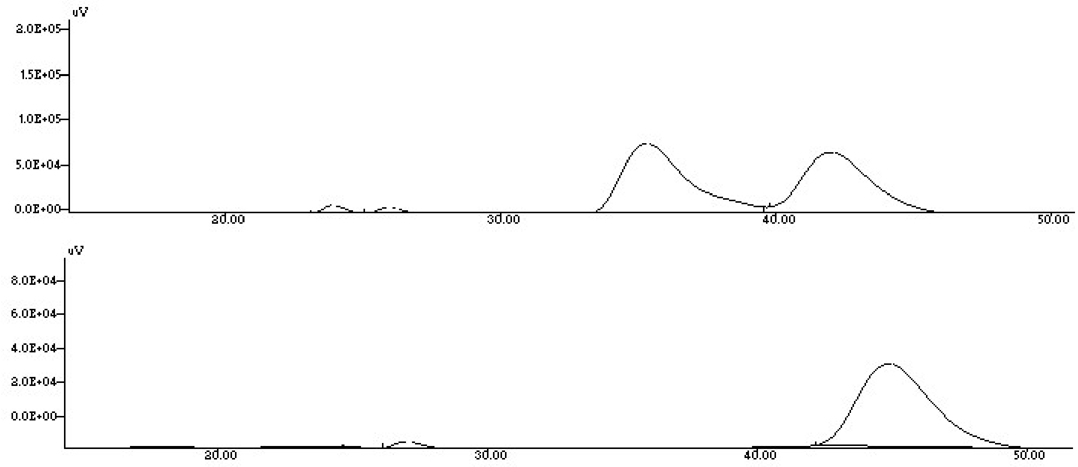

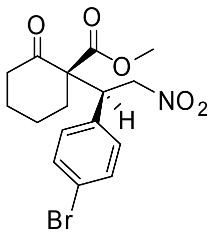

#	Name	RT	Area[uV.Sec]	Quantity
1		17.783	64424.000	0.000
2		19.742	75275.000	0.000
3		38.500	9593487.875	0.000
4		42.942	12434138.625	0.000

Total Area of Peak = 22167325.500 [uV.Sec]

HPLC [Chiralcel OJ-H, hexane/2-propanol = 80/20, 0.5 mL/min, λ = 210 nm, retention times: (major enantiomer) 42.9 min, (minor enantiomer) 38.5 min, (minor diastereomers) 17.7, 19.7 min].

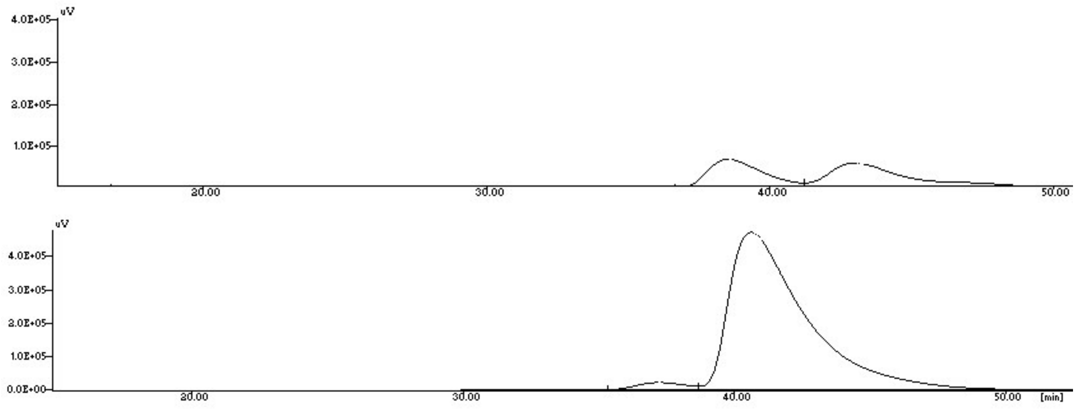

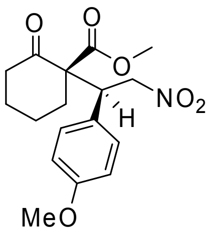

#	Name	RT	Area[uV.Sec]	Quantity
1		46.125	422053.658	0.000
2		57.408	360830.957	0.000
3		76.758	2534193.945	0.000
4		92.450	3004670.000	0.000

Total Area of Peak = 6321748.560 [uV.Sec]

HPLC [Chiralcel OJ-H, hexane/2-propanol = 80/20, 0.5 mL/min, λ = 210 nm, retention times: (major enantiomer) 92.4 min, (minor enantiomer) 76.7 min, (minor diastereomers) 46.1, 57.4 min].

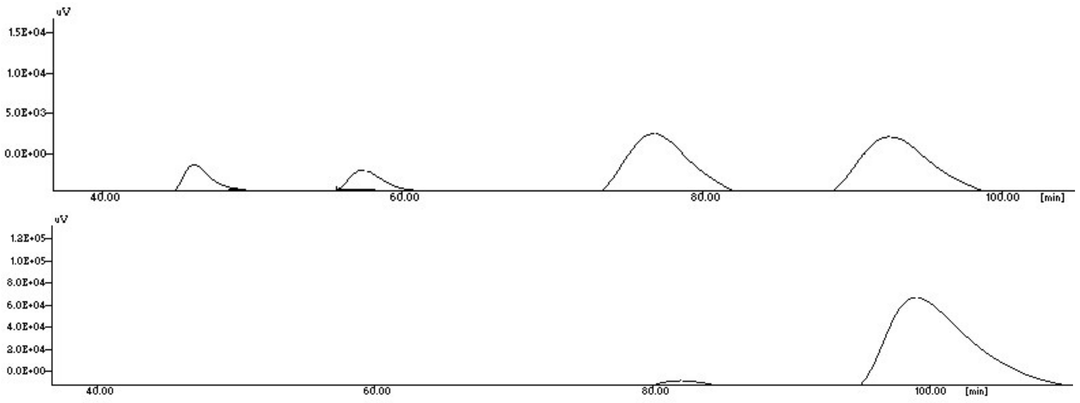

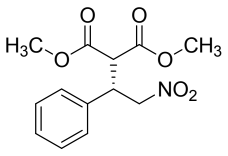

racemic

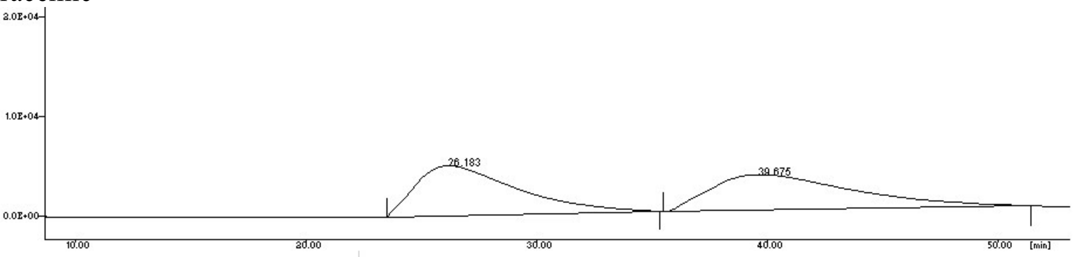

#	Name	RT	Area[uV.Sec]	Quantity
1		26.183	1542949.250	0.000
2		39.675	1517415.518	0.000

Total Area of Peak = 3060364.768 [uV.Sec]

asymmetric

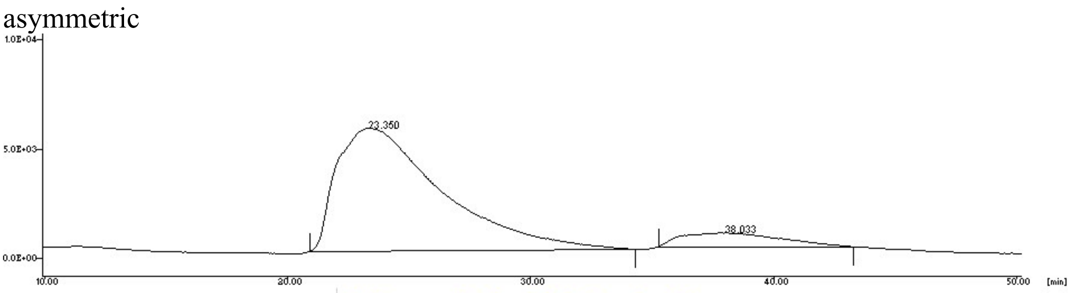

#	Name	RT	Area[uV.Sec]	Quantity
1		23.350	1665563.654	0.000
2		38.033	182731.754	0.000

Total Area of Peak = 1848295.407 [uV.Sec]

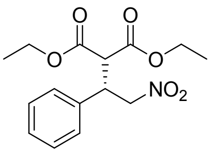

Racemic mixture

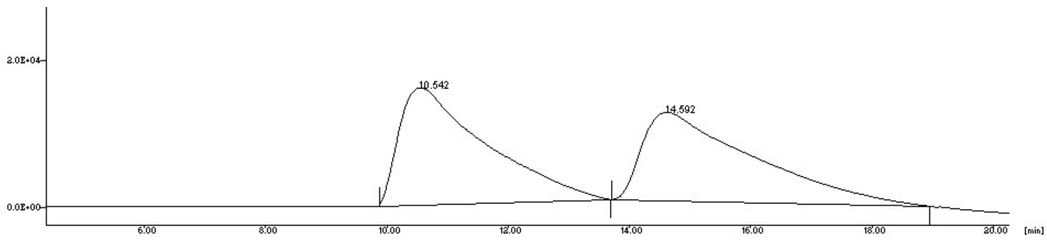

#	Name	RT	Area[uV.Sec]	Quantity
1		10.542	1553861.500	0.000
2		14.592	1514396.259	0.000

Total Area of Peak = 3068257.759 [uV.Sec]

asymmetric

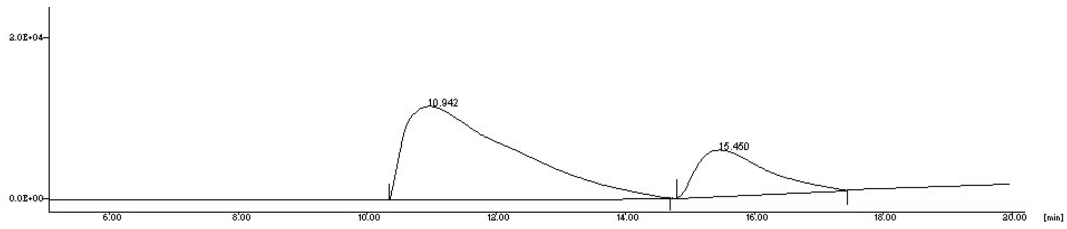

#	Name	RT	Area[uV.Sec]	Quantity
1		10.942	1398174.488	0.000
2		15.450	429596.250	0.000

Total Area of Peak = 1827770.738 [uV.Sec]

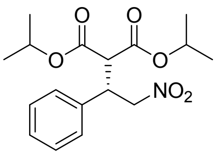

racemic

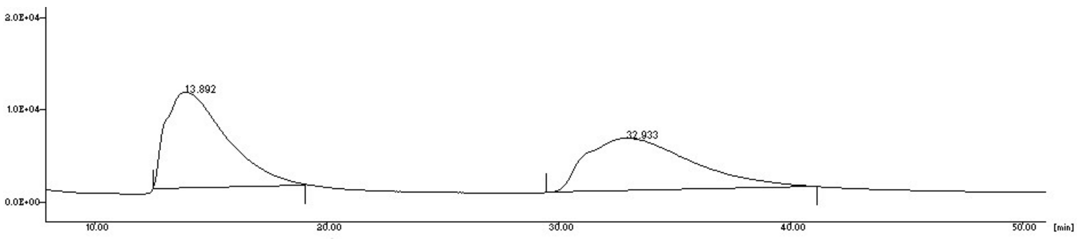

#	Name	RT	Area[uV.Sec]	Quantity
1		13.892	1836013.500	0.000
2		32.933	1790358.272	0.000

Total Area of Peak = 3626371.772 [uV.Sec]

asymmetric

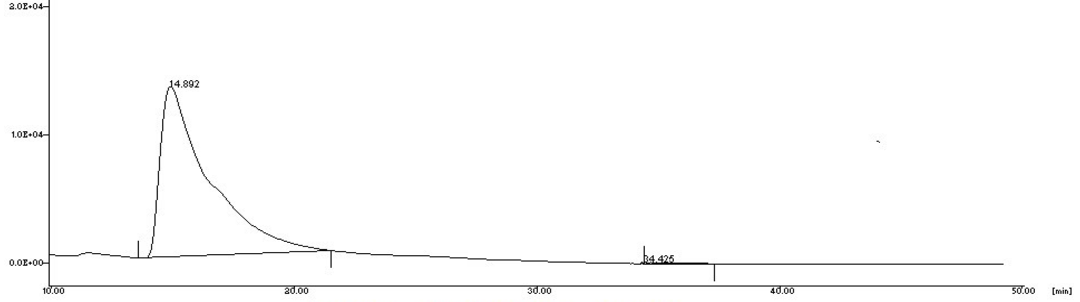

#	Name	RT	Area[uV.Sec]	Quantity
1		14.892	1819330.618	0.000
2		34.425	7135.957	0.000

Total Area of Peak = 1826466.575 [uV.Sec]

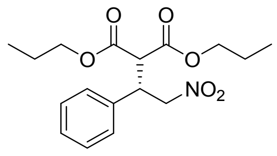

racemic

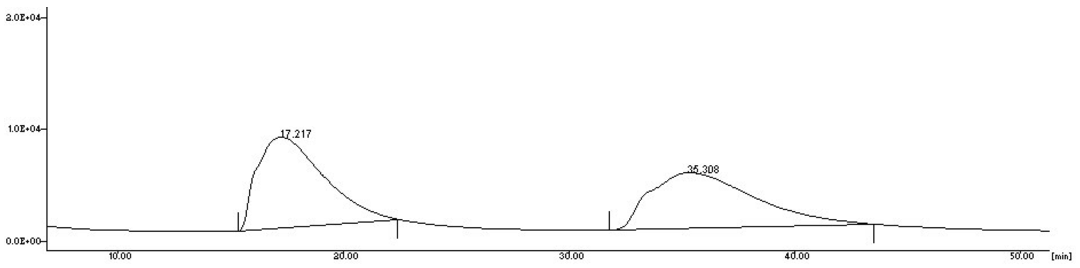

#	Name	RT	Area[uV.Sec]	Quantity
1		17.217	1630453.750	0.000
2		35.308	1582824.235	0.000

Total Area of Peak = 3213277.985 [uV.Sec]

asymmetric

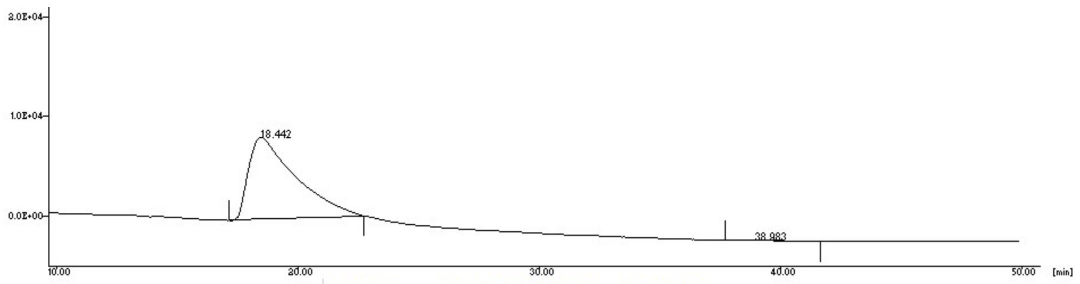

#	Name	RT	Area[uV.Sec]	Quantity
1		18.442	1095660.083	0.000
2		38.983	10333.713	0.000

Total Area of Peak = 1105993.796 [uV.Sec]

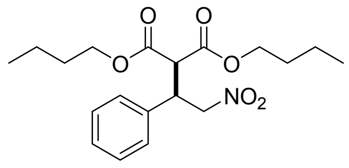

racemic

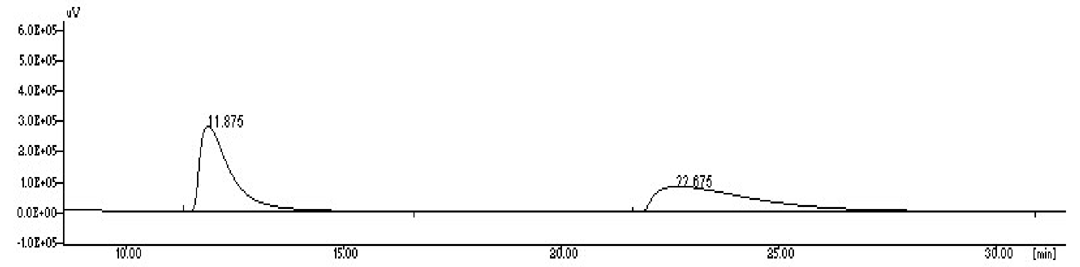

#	Name	RT	Area[uV.Sec]	Quantity
1		11.875	13421786.797	0.000
2		22.675	13376909.000	0.000

Total Area of Peak = 26798695.797 [uV.Sec]

asymmetric

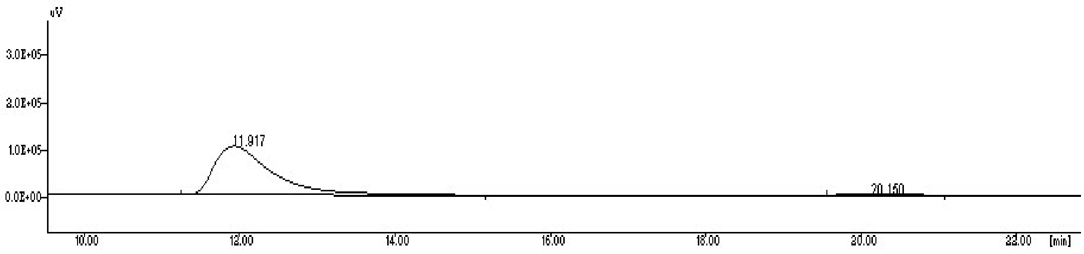

#	Name	RT	Area[uV.Sec]	Quantity
1		11.917	5341073.000	0.000
2		20.150	164684.000	0.000

Total Area of Peak = 5505757.000 [uV.Sec]

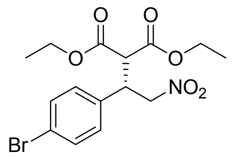

racemic

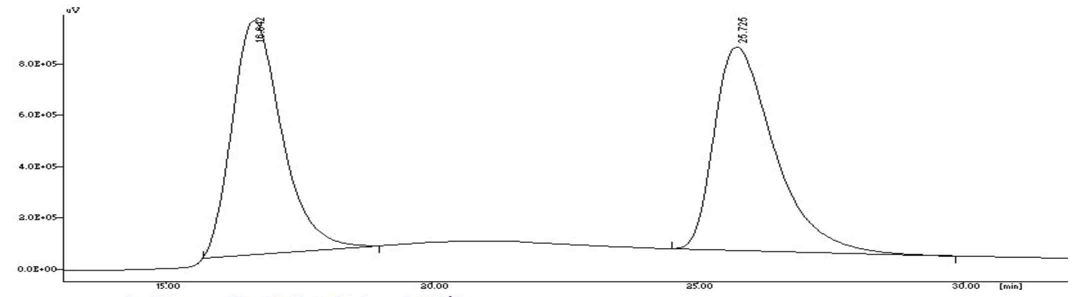

#	Name	RT	Area[uV.Sec]	Quantity
1		16.642	60491517.000	0.000
2		25.725	61270776.944	0.000

Total Area of Peak = 121762293.940 [uV.Sec]

asymmetric

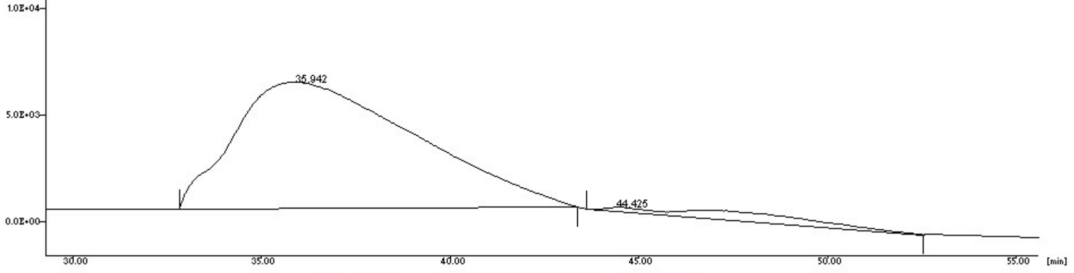

#	Name	RT	Area[uV.Sec]	Quantity
1		35.942	1972322.854	0.000
2		44.425	119808.443	0.000

Total Area of Peak = 2092131.297 [uV.Sec]

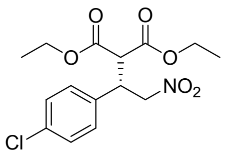

racemic

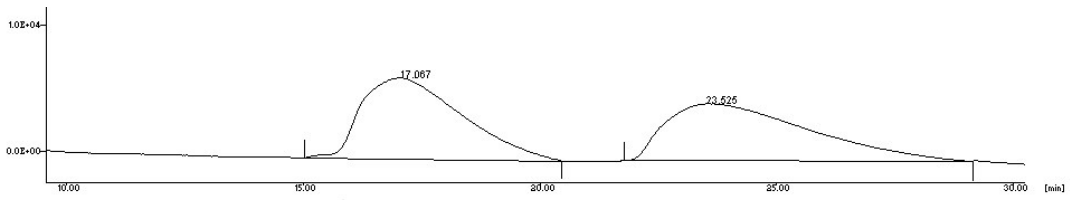

#	Name	RT	Area[uV.Sec]	Quantity
1		17.067	932562.750	0.000
2		23.525	943874.557	0.000

Total Area of Peak = 1876437.307 [uV.Sec]

asymmetric

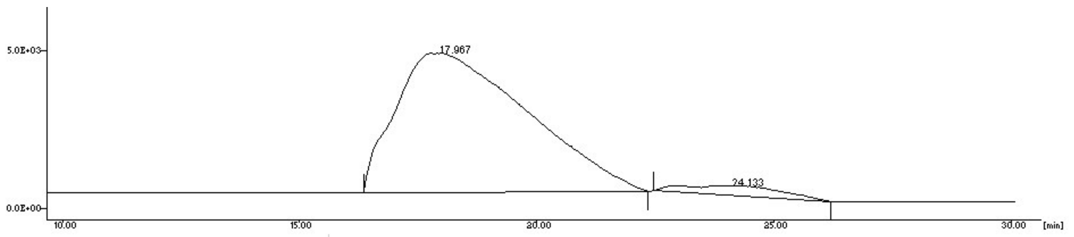

#	Name	RT	Area[uV.Sec]	Quantity
1		17.967	829056.000	0.000
2		24.133	39260.000	0.000

Total Area of Peak = 868316.000 [uV.Sec]

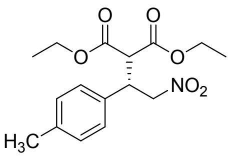

racemic

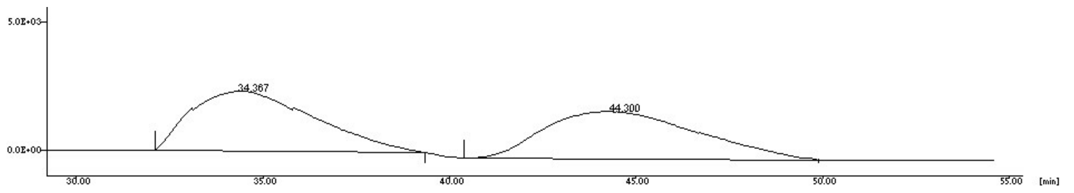

#	Name	RT	Area[uV.Sec]	Quantity
1		34.367	528153.367	0.000
2		44.300	522110.714	0.000

Total Area of Peak = 1050264.081 [uV.Sec]

asymmetric

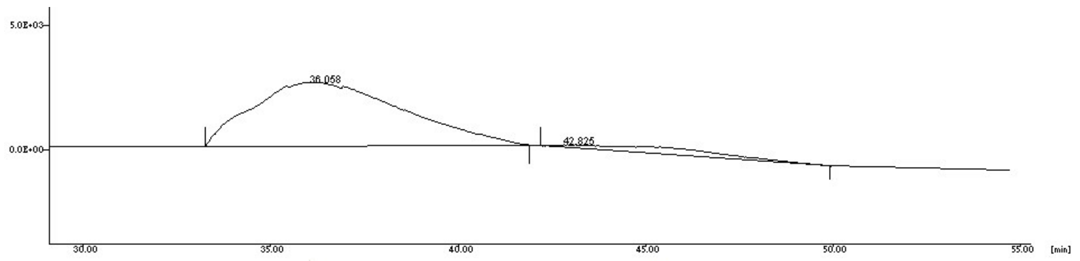

#	Name	RT	Area[uV.Sec]	Quantity
1		36.058	714876.500	0.000
2		42.825	68679.473	0.000

Total Area of Peak = 783555.973 [uV.Sec]

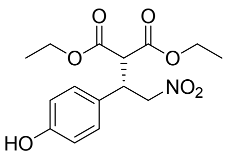

racemic

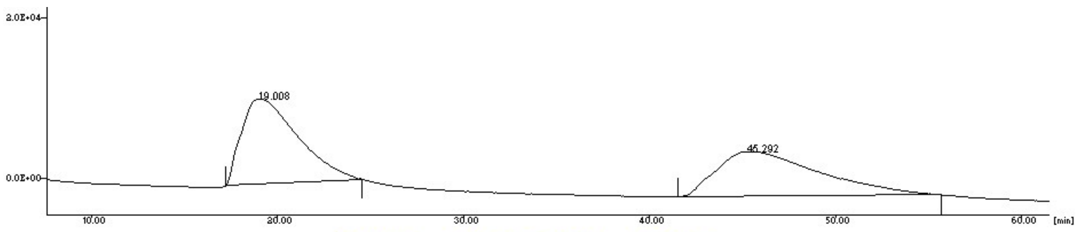

#	Name	RT	Area[uV.Sec]	Quantity
1		19.008	2185303.500	0.000
2		45.292	2130083.798	0.000

Total Area of Peak = 4315387.298 [uV.Sec]

asymmetric

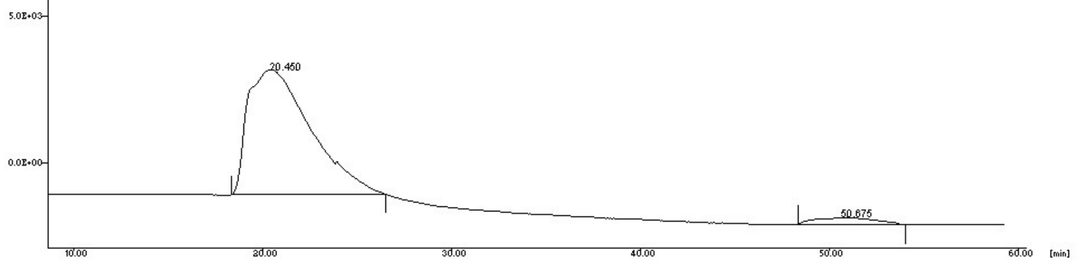

#	Name	RT	Area[uV.Sec]	Quantity
1		20.450	977542.507	0.000
2		50.675	52967.106	0.000

Total Area of Peak = 1030509.613 [uV.Sec]

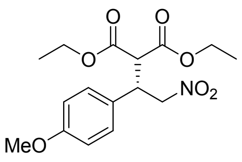

racemic

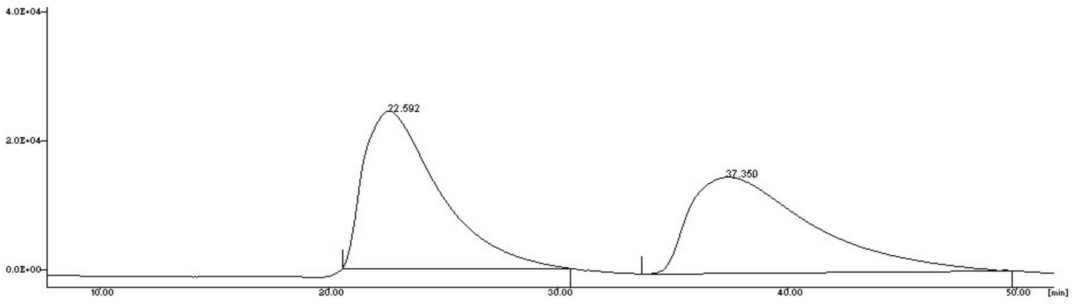

#	Name	RT	Area[uV.Sec]	Quantity
1		22.592	5516084.082	0.000
2		37.350	5478535.787	0.000

Total Area of Peak = 10994619.870 [uV.Sec]

asymmetric

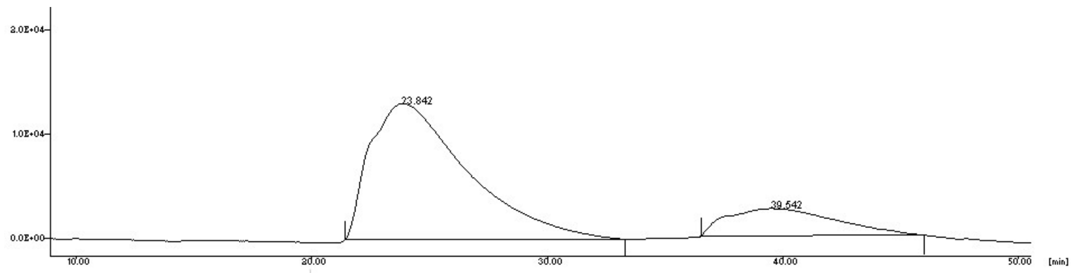

#	Name	RT	Area[uV.Sec]	Quantity
1		23.842	3696676.981	0.000
2		39.542	831848.000	0.000

Total Area of Peak = 4528524.981 [uV.Sec]

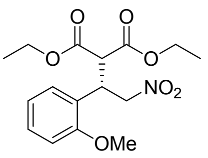

racemic

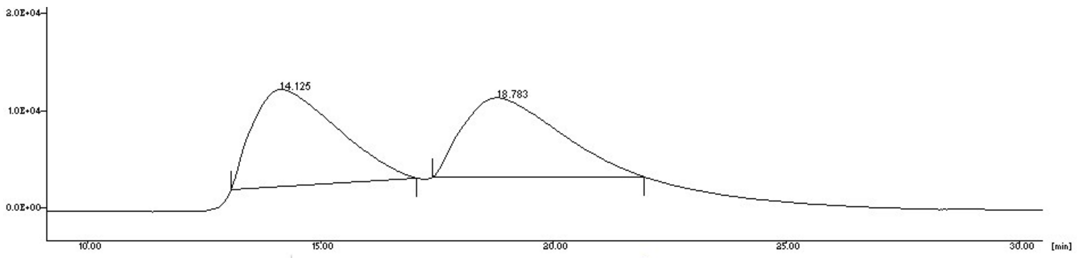

#	Name	RT	Area[uV.Sec]	Quantity
1		14.125	1241599.500	0.000
2		18.783	1159655.500	0.000

Total Area of Peak = 2401255.000 [uV.Sec]

asymmetric

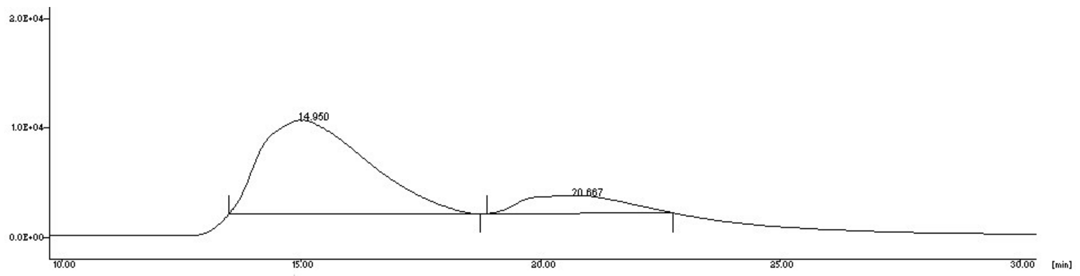

#	Name	RT	Area[uV.Sec]	Quantity
1		14.950	1319133.644	0.000
2		20.667	238418.500	0.000

Total Area of Peak = 1557552.144 [uV.Sec]

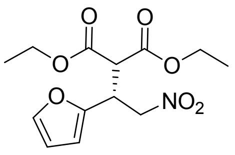

racemic

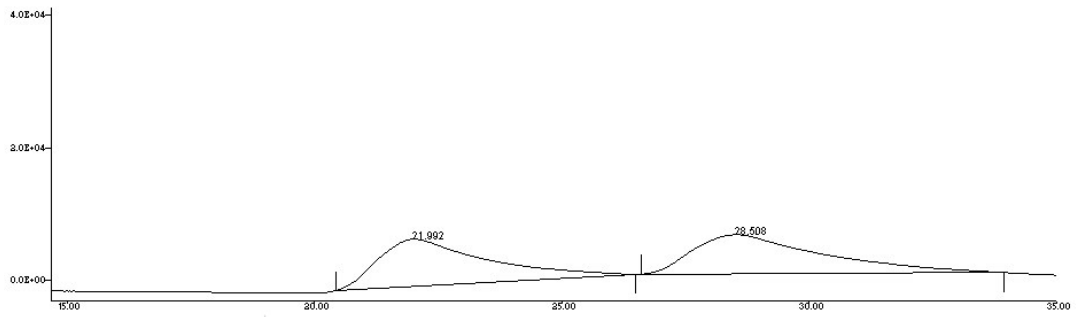

#	Name	RT	Area[uV.Sec]	Quantity
1		21.992	1070477.324	0.000
2		28.508	1060758.250	0.000

Total Area of Peak = 2131235.574 [uV.Sec]

asymmetric

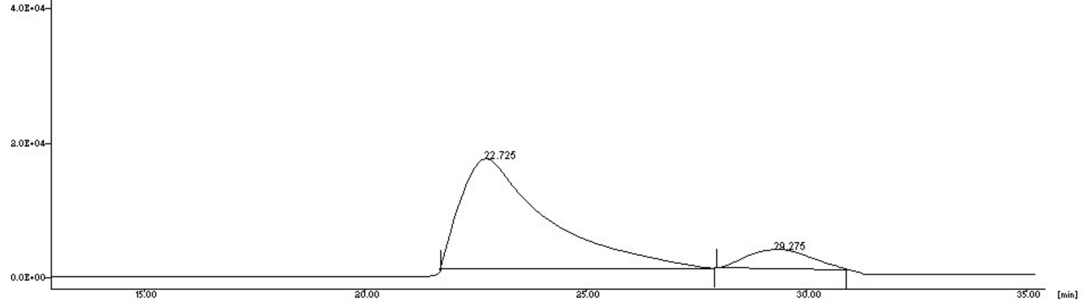

#	Name	RT	Area[uV.Sec]	Quantity
1		22.725	2206332.877	0.000
2		29.275	279742.500	0.000

Total Area of Peak = 2486075.377 [uV.Sec]

Table 1. Michael reactions using catalyst **1d**.

Cycle	product	Catalyst recovery [%][a]	yield [%][a]	ee [%][b]
1st	**5d**	99	98	99
2nd	**5d**	99	97	99
3rd	**5d**	99	97	99
4th	**5d**	99	97	99

[a]Isolated yield. [b]Determined by chiral HPLC.

1st cycle

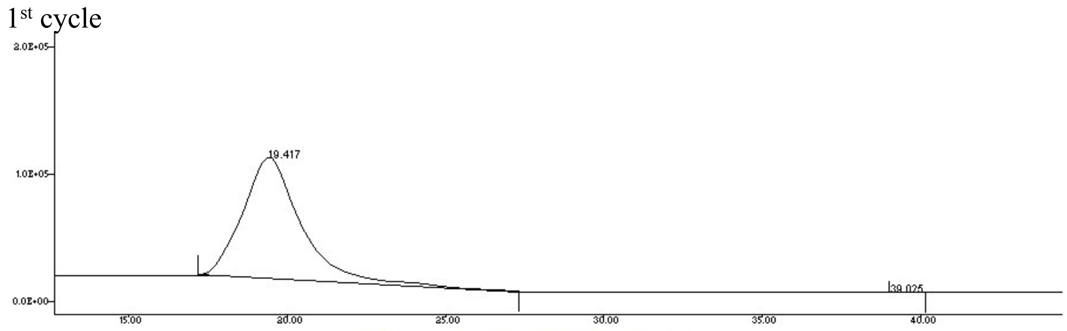

#	Name	RT	Area[uV.Sec]	Quantity
1		19.417	11935101.706	0.000
2		39.025	6669.500	0.000

Total Area of Peak = 11941771.206 [uV.Sec]

2nd cycle

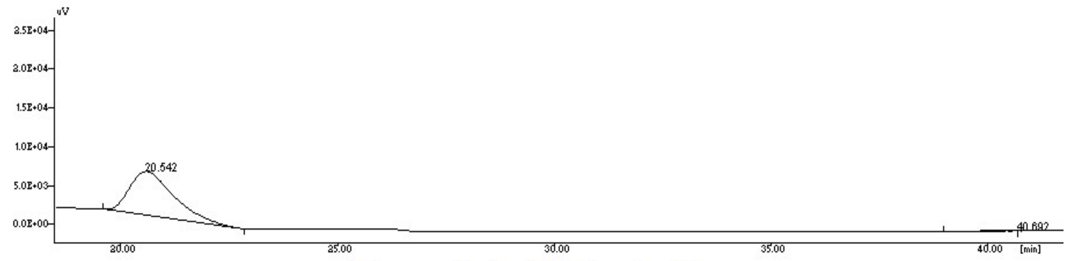

#	Name	RT	Area[uV.Sec]	Quantity
1		20.542	390327.913	0.000
2		40.692	676.451	0.000

Total Area of Peak = 391004.364 [uV.Sec]

3rd cycle

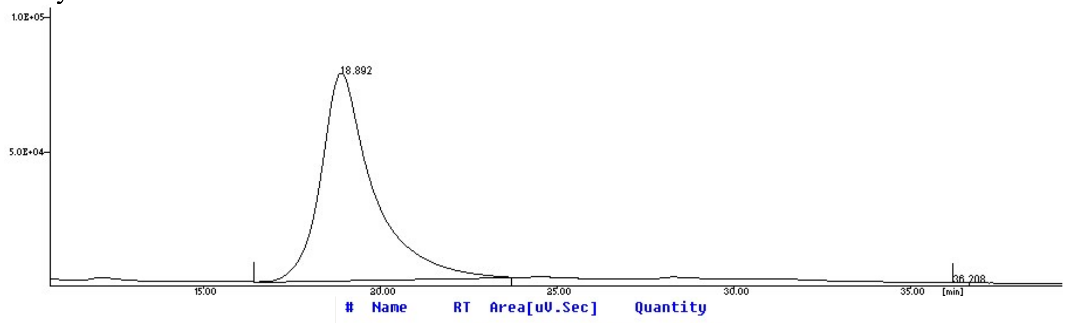

#	Name	RT	Area[uV.Sec]	Quantity
1		18.892	7788954.382	0.000
2		36.208	201.614	0.000

Total Area of Peak = 7789155.996 [uV.Sec]

4th cycle

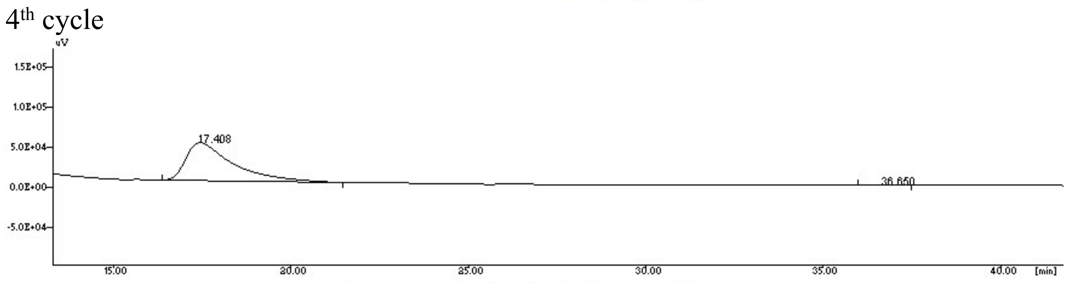

#	Name	RT	Area[uV.Sec]	Quantity
1		17.408	4239882.250	0.000
2		36.650	20305.500	0.000

Total Area of Peak = 4260187.750 [uV.Sec]

NMR Spectra

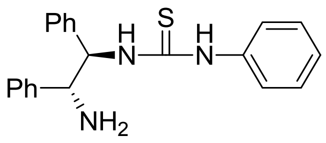

1a

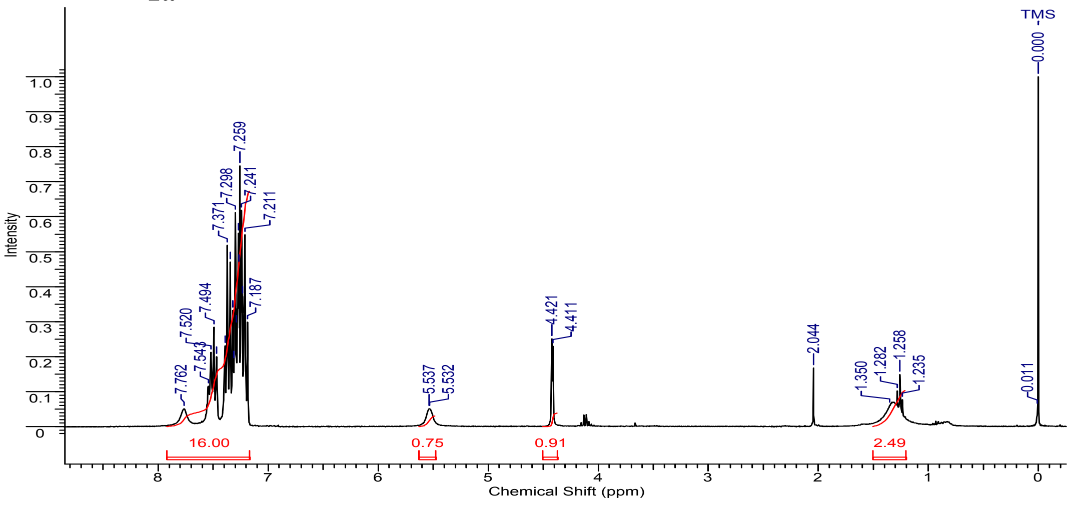

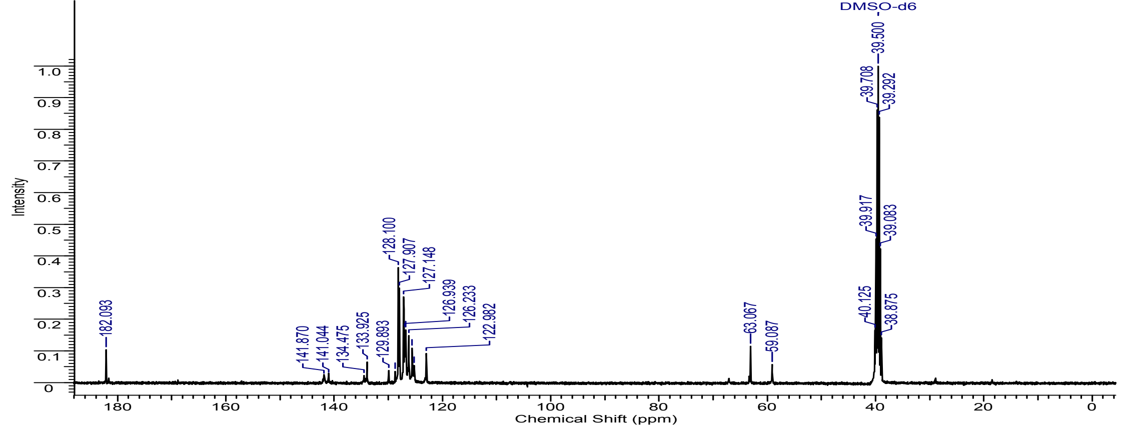
DMSO-d6
Intensity
Chemical Shift (ppm)

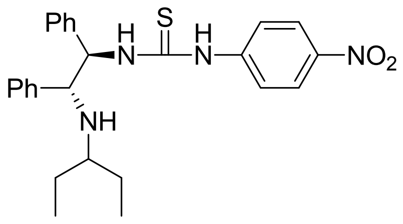

1b

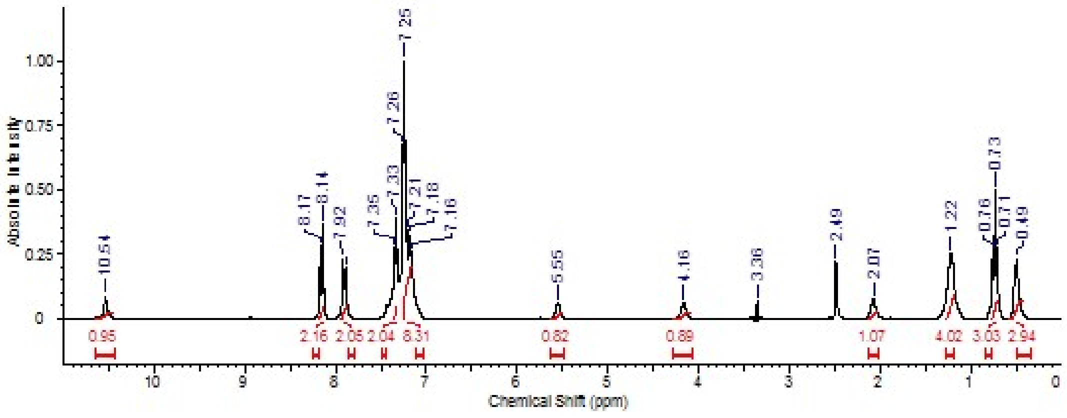
Absolute Intensity
Chemical Shift (ppm)

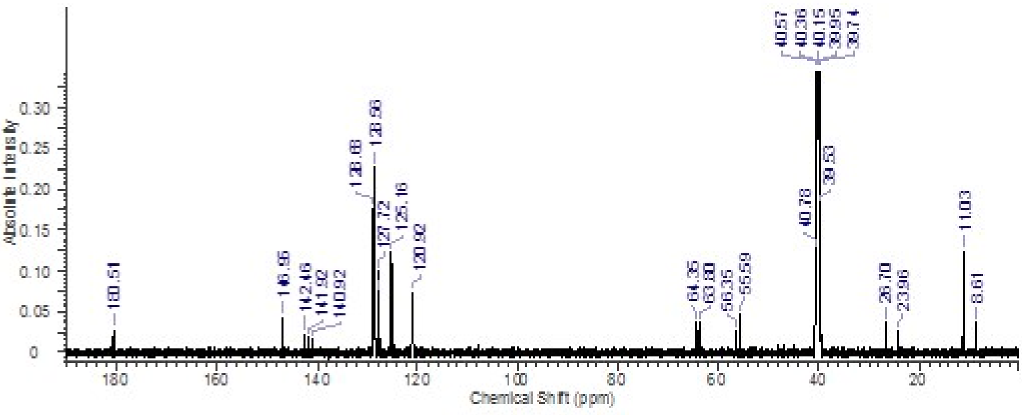
Absolute Intensity
Chemical Shift (ppm)

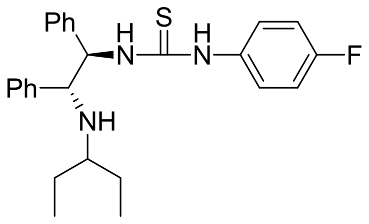

1c

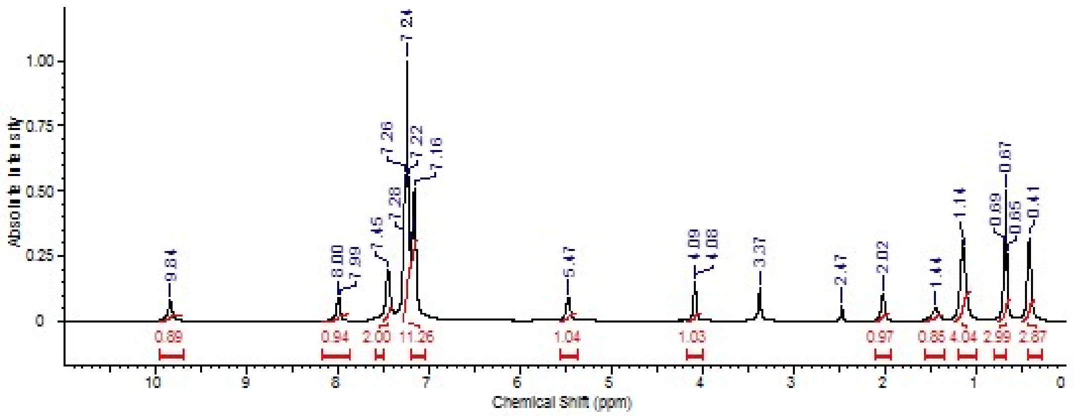
Absolute Intensity
Chemical Shift (ppm)

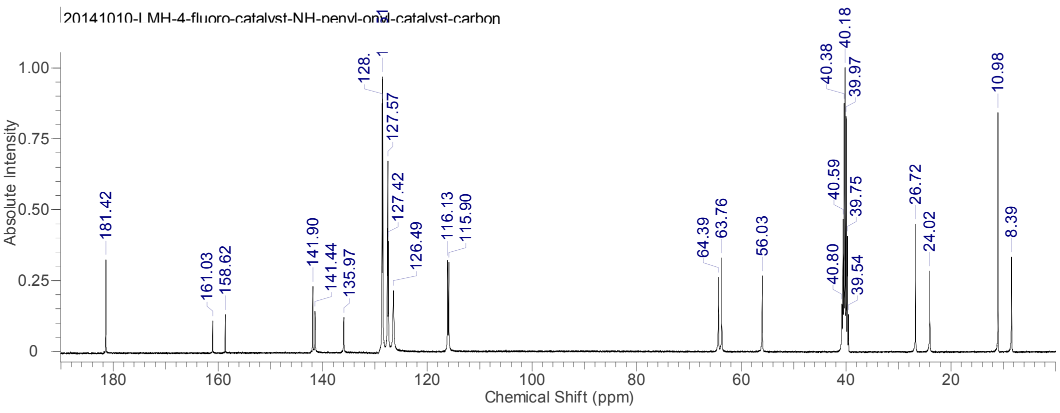
20141010-LMH-4-fluoro-catalyst-NH-pentyl-one-catalyst-carbon
Absolute Intensity
Chemical Shift (ppm)

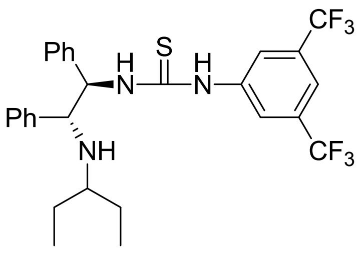

1d

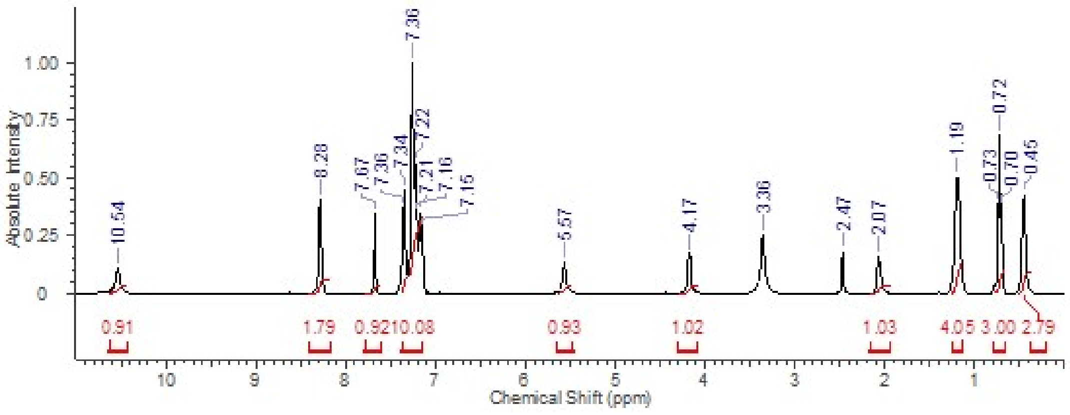
Absolute Intensity
Chemical Shift (ppm)

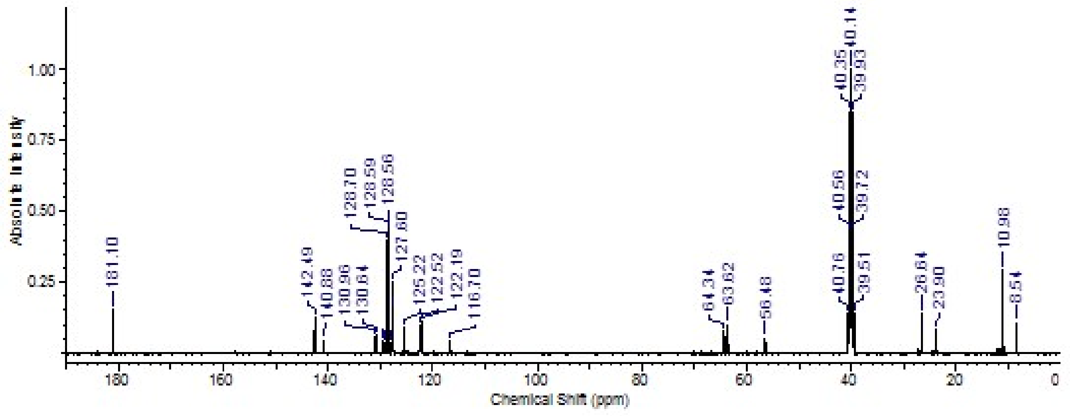
Absolute Intensity
Chemical Shift (ppm)

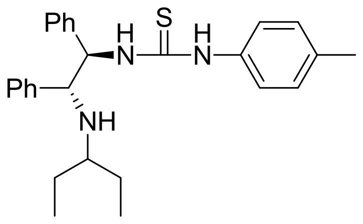
Ph
H
N
S
H
N
Ph
NH

1e

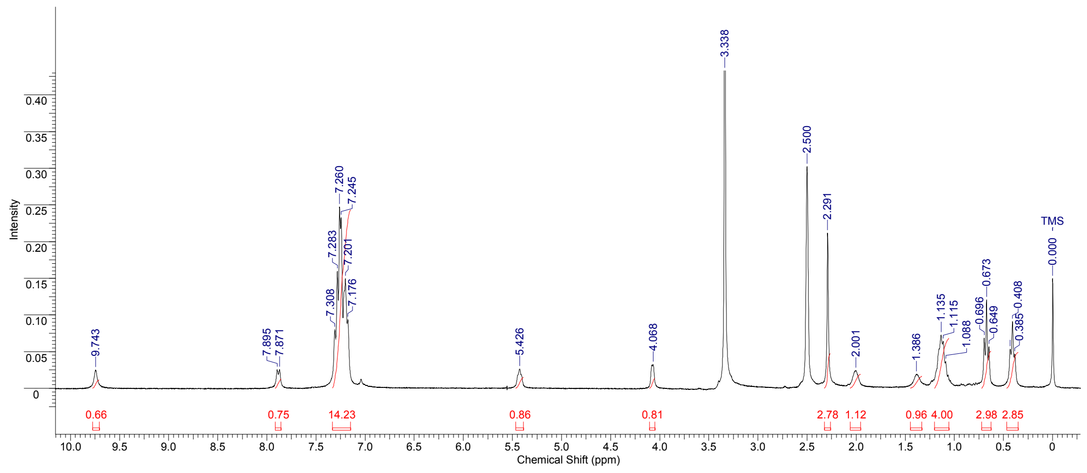
Intensity
TMS
Chemical Shift (ppm)

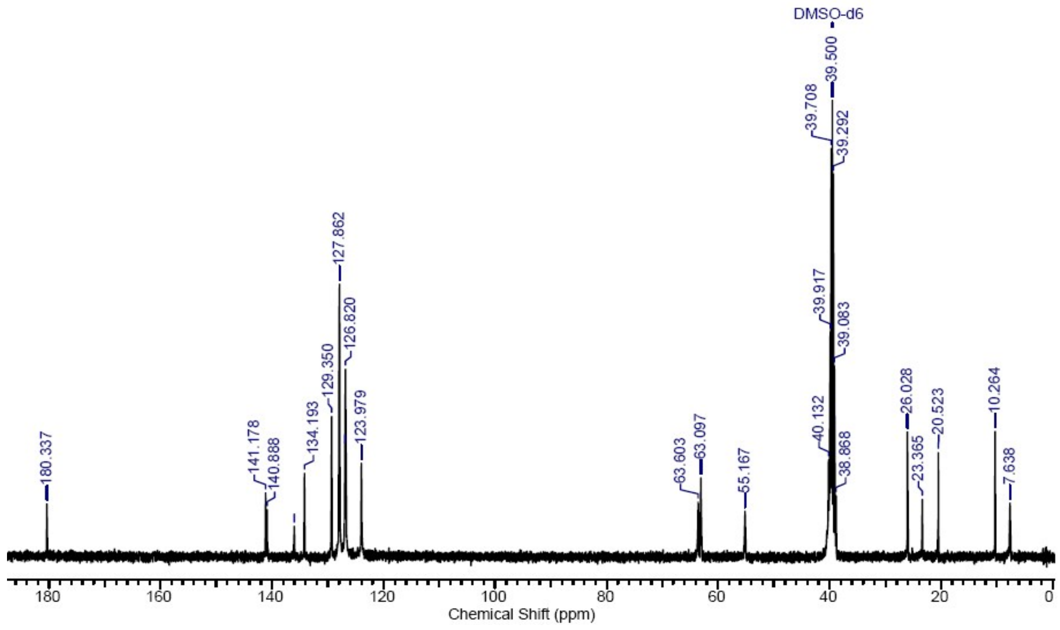
DMSO-d6
Chemical Shift (ppm)

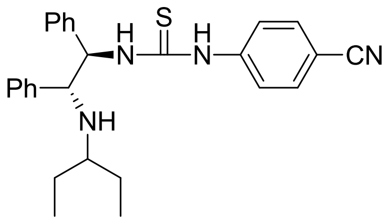
Ph
H
N
S
H
N
CN
Ph
NH

1f

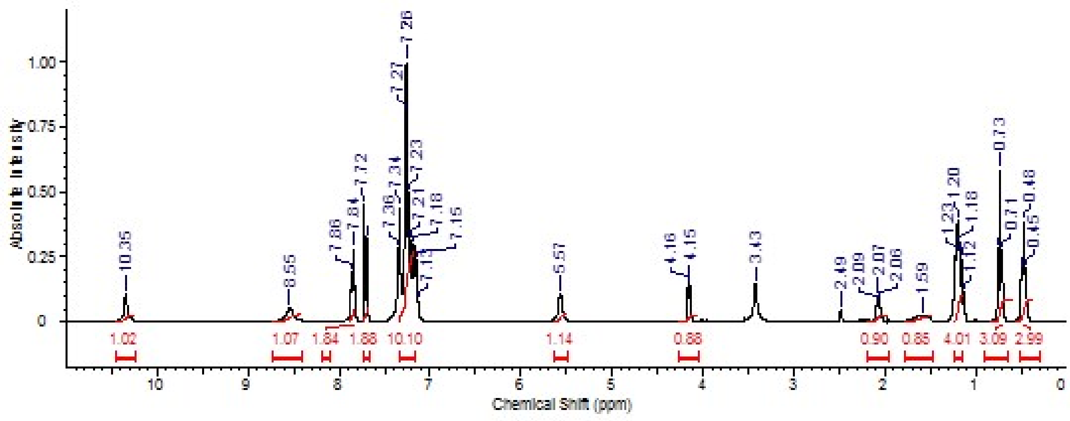
Absolute Intensity
Chemical Shift (ppm)

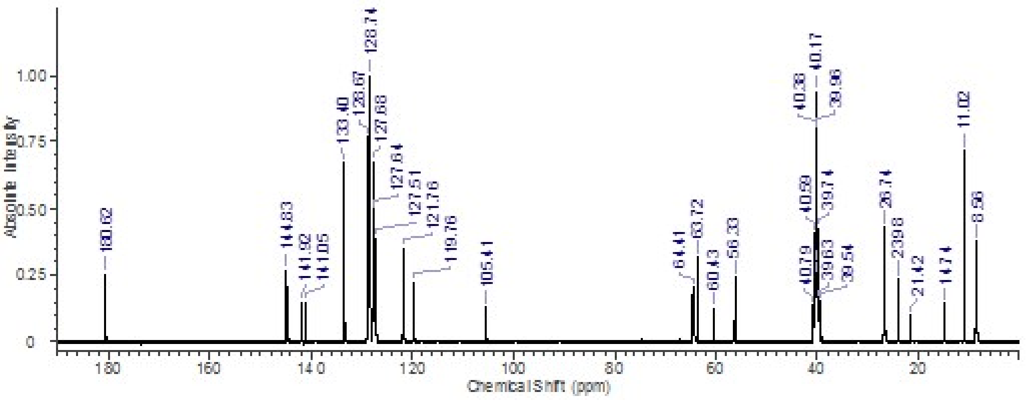
Absolute Intensity
Chemical Shift (ppm)
180.62
144.83
141.92
141.05
133.40
128.67
128.74
127.68
127.64
127.51
121.76
119.76
105.41
64.41
63.72
60.43
56.33
40.38
40.17
39.96
40.59
39.74
40.79
39.63
39.54
26.74
23.98
21.42
14.74
11.02
8.56

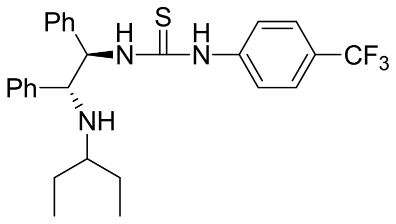
Ph
H
N
S
H
N
CF3
Ph
NH

1g

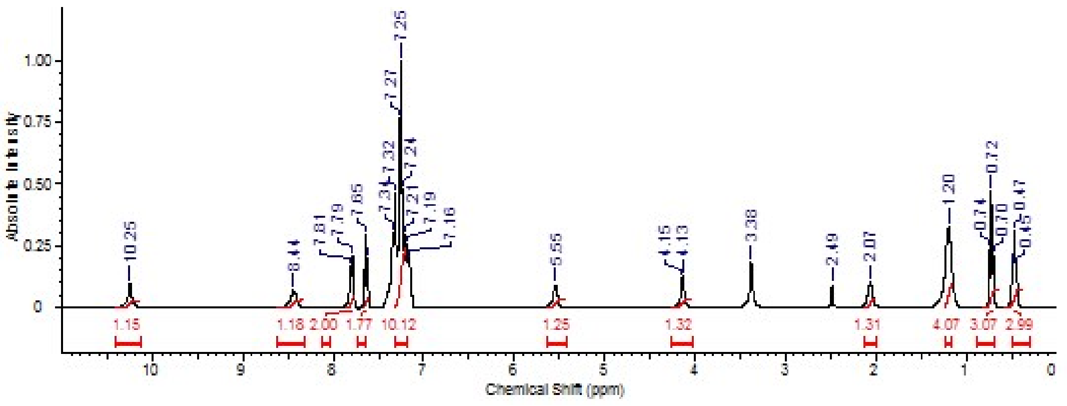
Absolute Intensity
Chemical Shift (ppm)
10.25
8.44
7.81
7.79
7.65
7.34
7.32
7.27
7.25
7.24
7.21
7.19
7.16
5.55
4.15
4.13
3.38
2.49
2.07
1.20
0.74
0.72
0.70
0.47
0.45
1.15
1.18
2.00
1.77
10.12
1.25
1.32
1.31
4.07
3.07
2.99

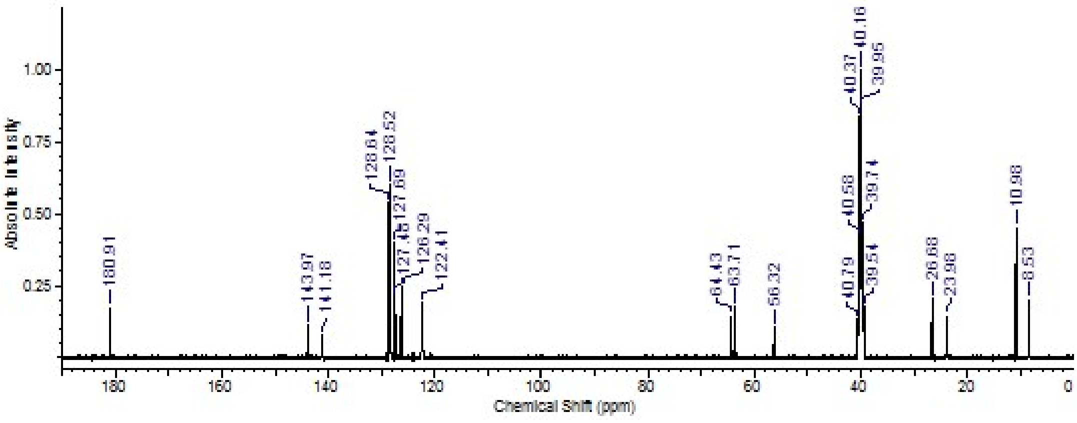
Absolute Intensity
Chemical Shift (ppm)
180.91
143.97
141.18
128.64
128.52
127.69
127.46
126.29
122.41
64.43
63.71
56.32
40.79
40.58
40.37
40.16
39.95
39.74
39.54
26.68
23.98
10.98
8.53

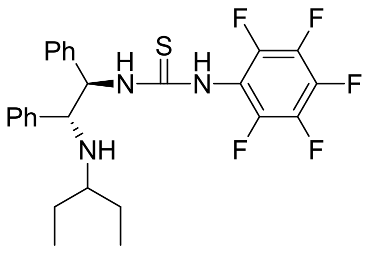
Ph
H
N
S
H
N
F
F
F
F
F
Ph
NH

1h

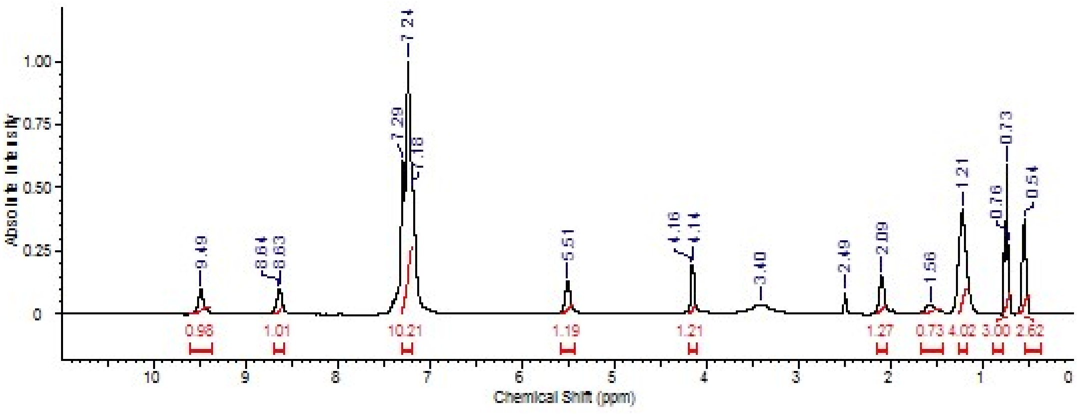
Absolute Intensity
Chemical Shift (ppm)
9.49
8.64
8.63
7.29
7.24
7.18
5.51
4.16
4.14
3.40
2.49
2.09
1.56
1.21
0.76
0.73
0.54
0.98
1.01
10.21
1.19
1.21
1.27
0.73
4.02
3.00
2.62

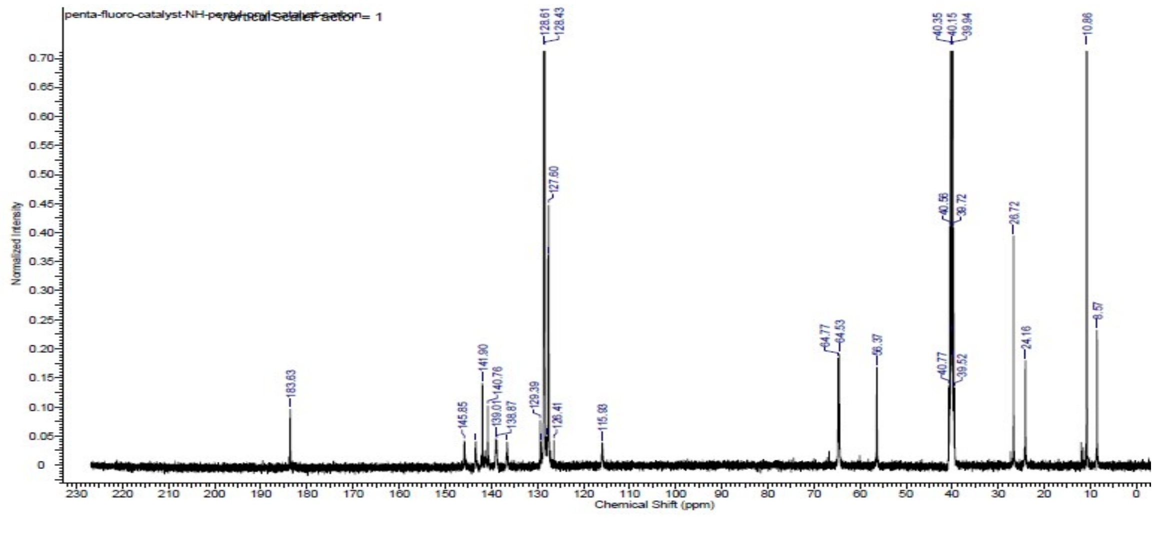
Normalized Intensity
Chemical Shift (ppm)

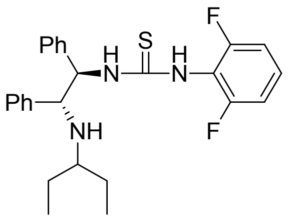
Ph
H
N
S
H
N
F
F
Ph
NH

1i

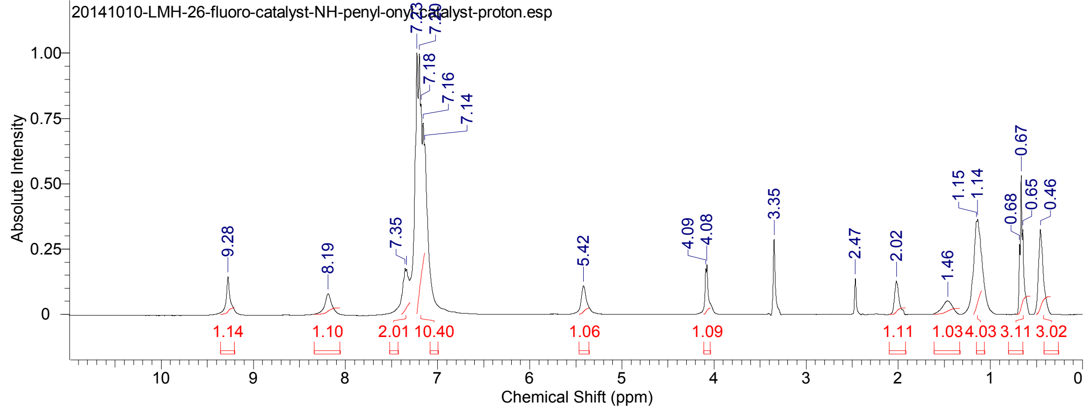
20141010-LMH-26-fluoro-catalyst-NH-penyl-onyl-catalyst-proton.esp
Absolute Intensity
Chemical Shift (ppm)

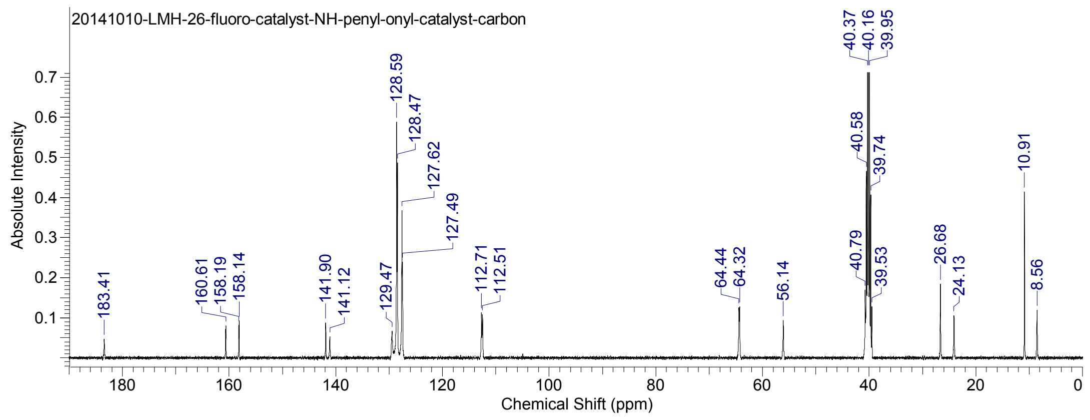
20141010-LMH-26-fluoro-catalyst-NH-penyl-onyl-catalyst-carbon
Absolute Intensity
Chemical Shift (ppm)

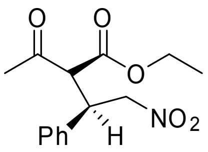
O
O
O
Ph
H
NO2

4a

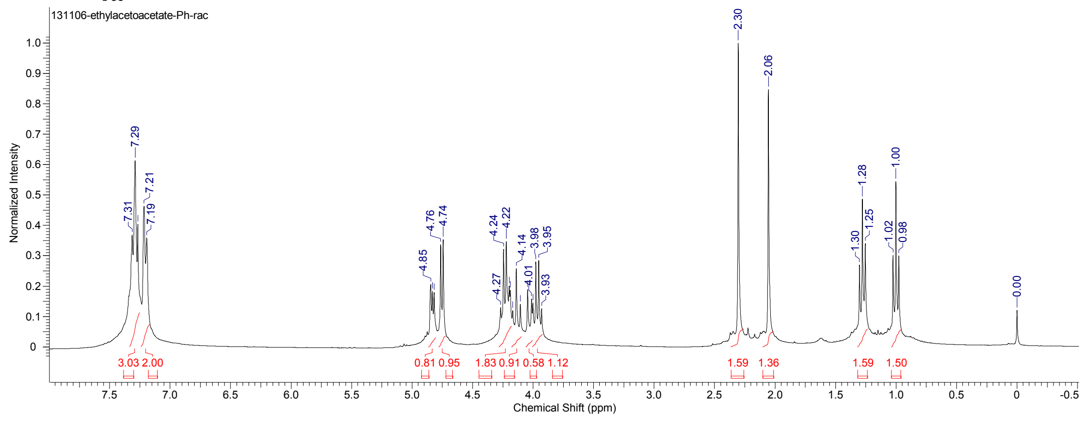
131106-ethylacetoacetate-Ph-rac
Normalized Intensity
Chemical Shift (ppm)

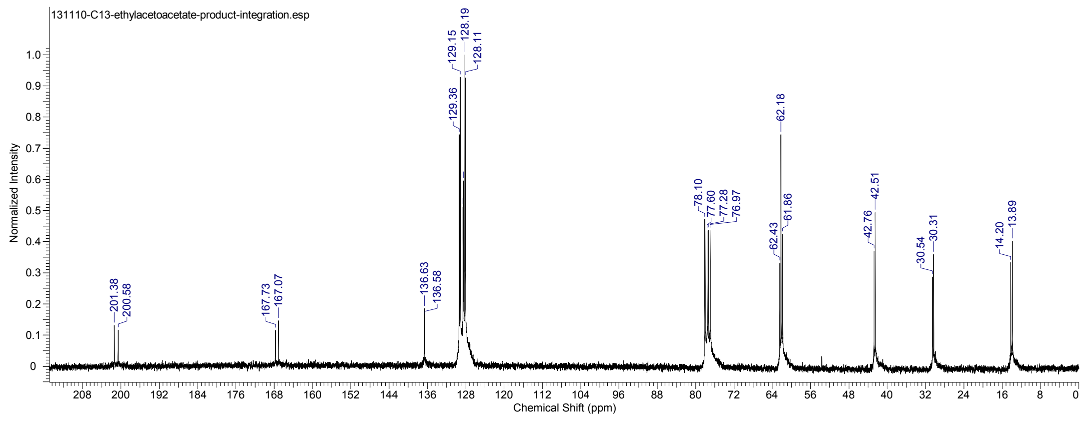
131110-C13-ethylacetoacetate-product-integration.esp
Normalized Intensity
Chemical Shift (ppm)

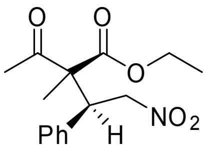
O
O
O
Ph
H
NO2

4b

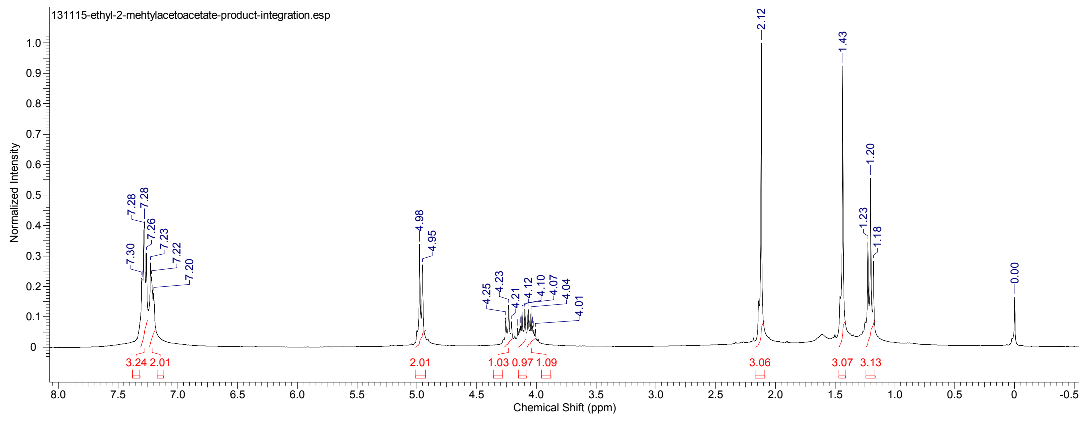
131115-ethyl-2-mehtylacetoacetate-product-integration.esp
Normalized Intensity
Chemical Shift (ppm)

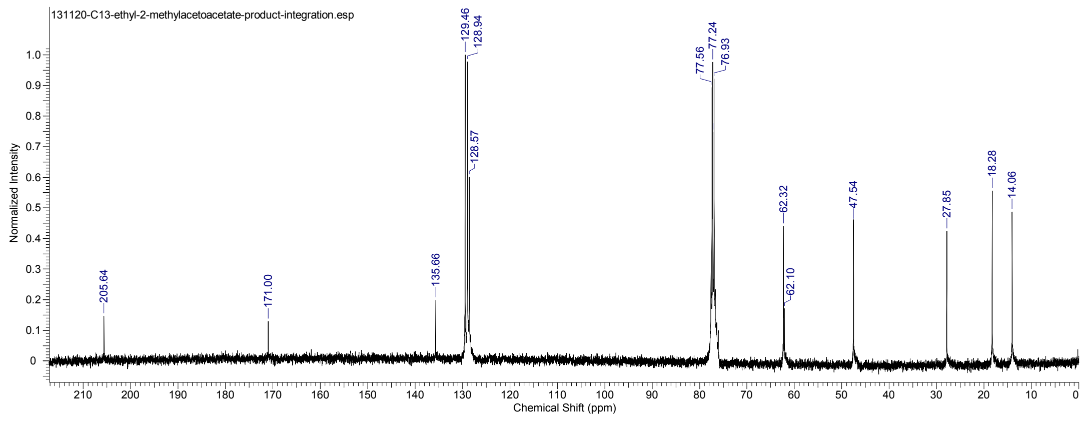
131120-C13-ethyl-2-methylacetoacetate-product-integration.esp
Normalized Intensity
Chemical Shift (ppm)

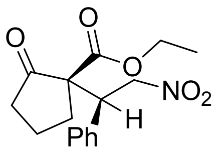
O
O
O
NO2
Ph
H

4c

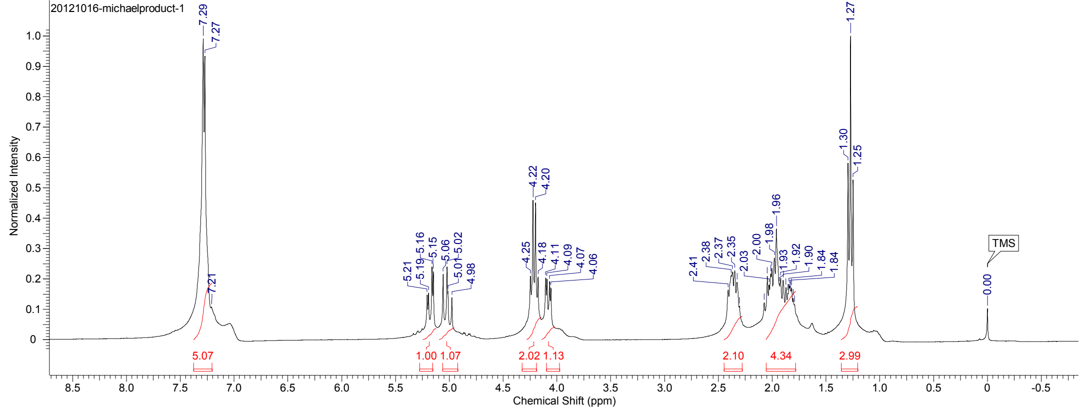
20121016-michaelproduct-1
Normalized Intensity
Chemical Shift (ppm)
TMS

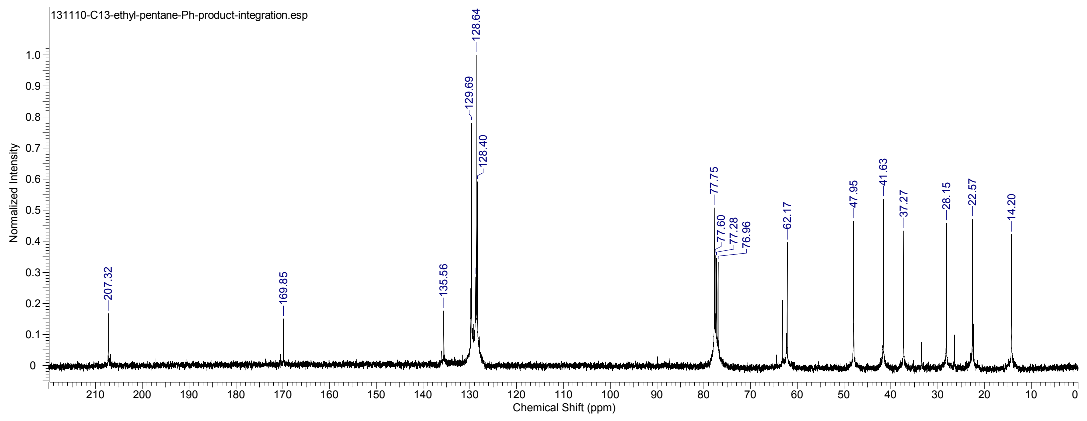
131110-C13-ethyl-pentane-Ph-product-integration.esp
Normalized Intensity
Chemical Shift (ppm)

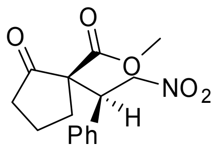
O
O
O
NO2
H
Ph

4d

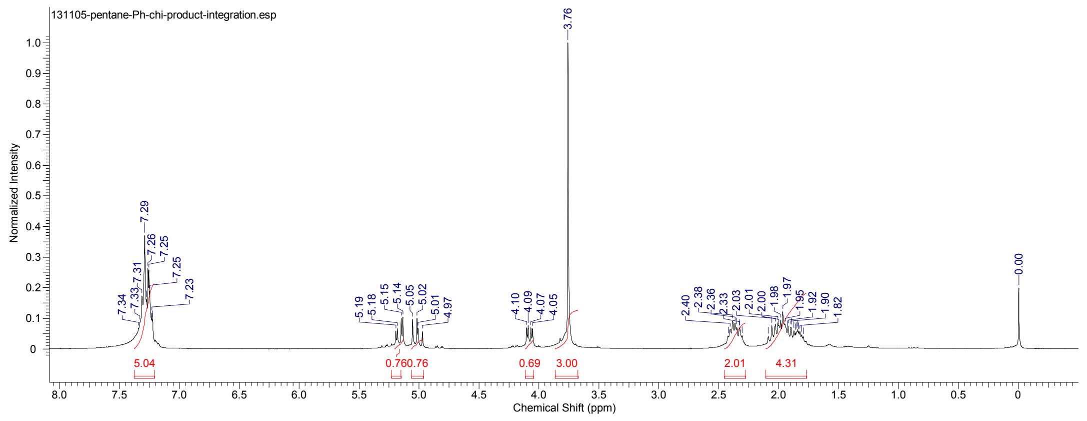
131105-pentane-Ph-chi-product-integration.esp
Normalized Intensity
Chemical Shift (ppm)

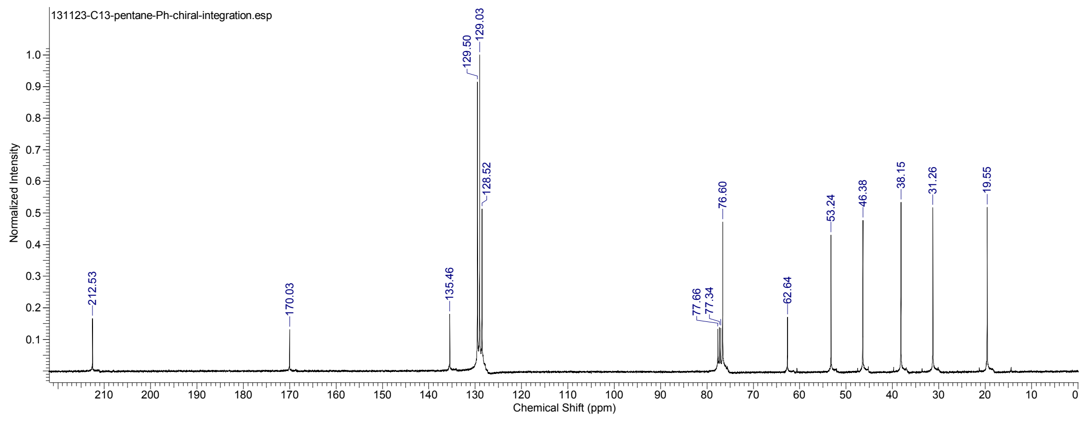
131123-C13-pentane-Ph-chiral-integration.esp
Normalized Intensity
Chemical Shift (ppm)

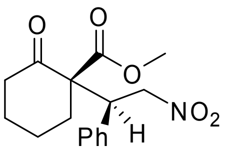
O
O
O
NO2
Ph
H

4e

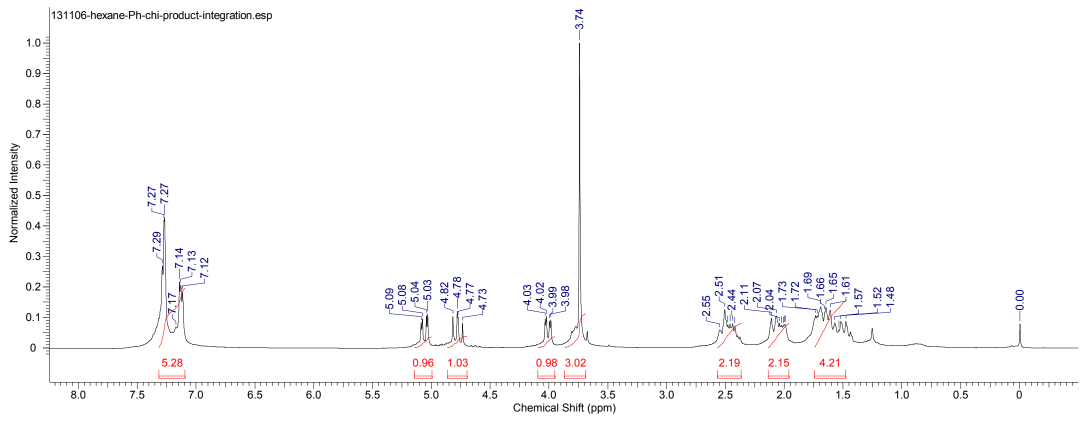
131106-hexane-Ph-chi-product-integration.esp
Normalized Intensity
Chemical Shift (ppm)

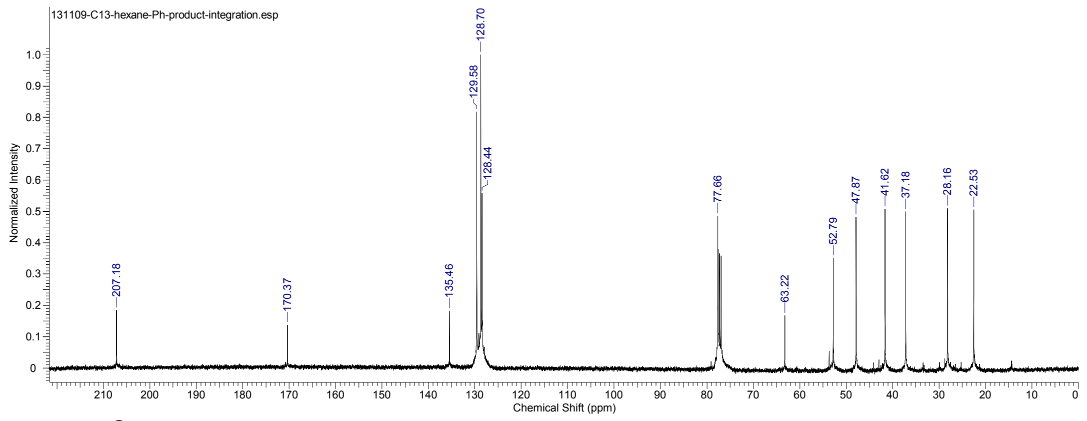
131109-C13-hexane-Ph-product-integration.esp
Normalized Intensity
Chemical Shift (ppm)

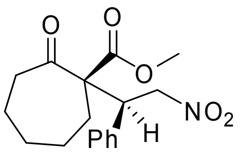
O
O
O
NO2
Ph
H

4f

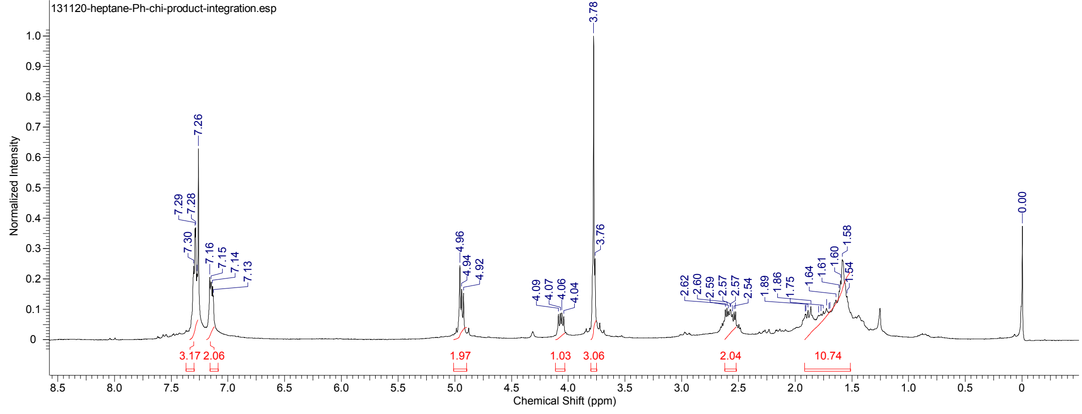
131120-heptane-Ph-chi-product-integration.esp
Normalized Intensity
Chemical Shift (ppm)

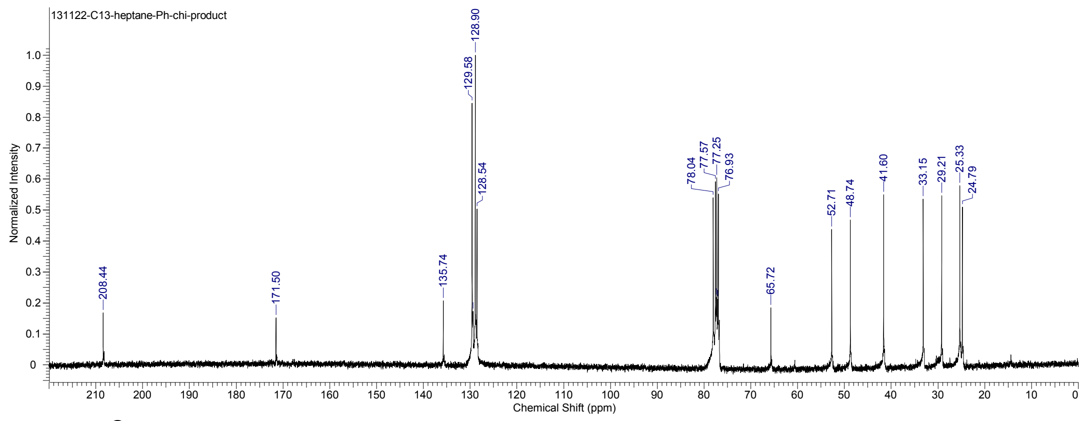
131122-C13-heptane-Ph-chi-product
Normalized Intensity
Chemical Shift (ppm)

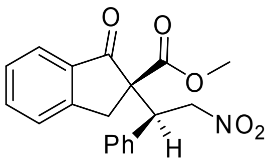
O
O
O
NO2
Ph
H

4g

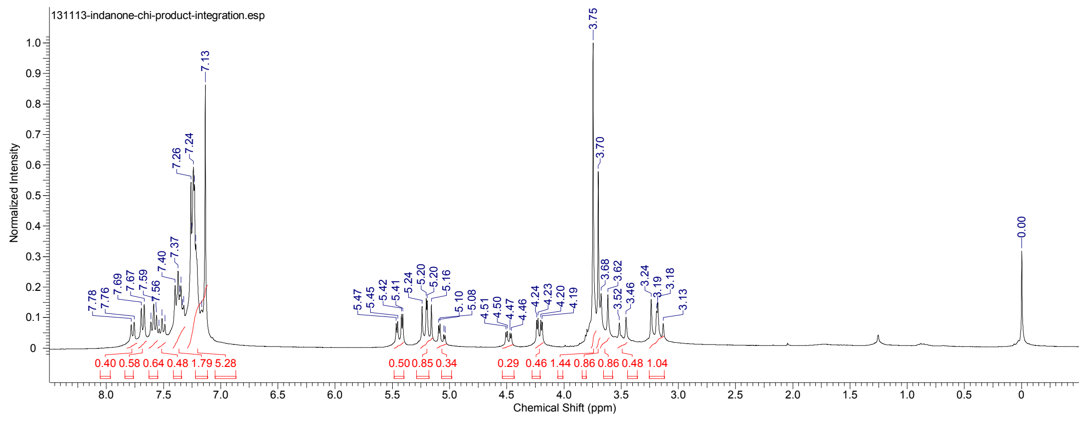
131113-indanone-chi-product-integration.esp
Normalized Intensity
Chemical Shift (ppm)

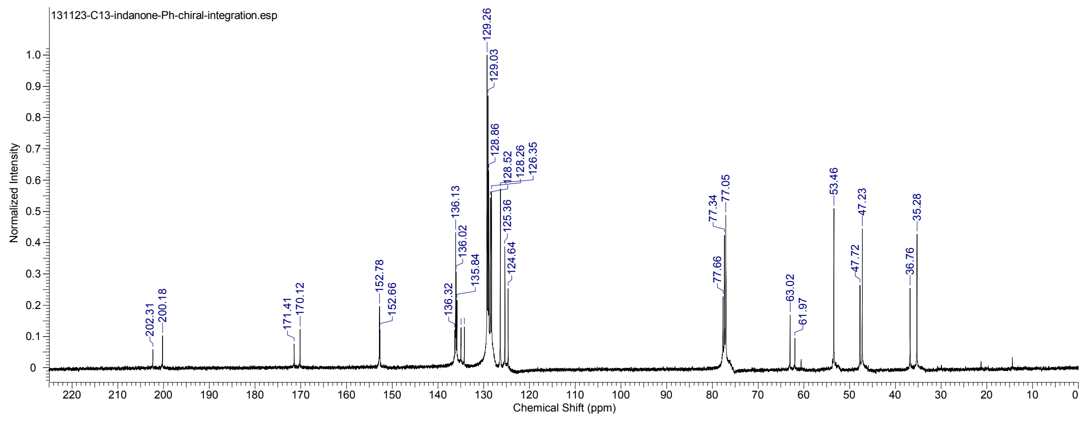
131123-C13-indanone-Ph-chiral-integration.esp
Normalized Intensity
Chemical Shift (ppm)

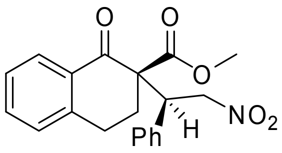
O
O
O
NO2
Ph
H

4h

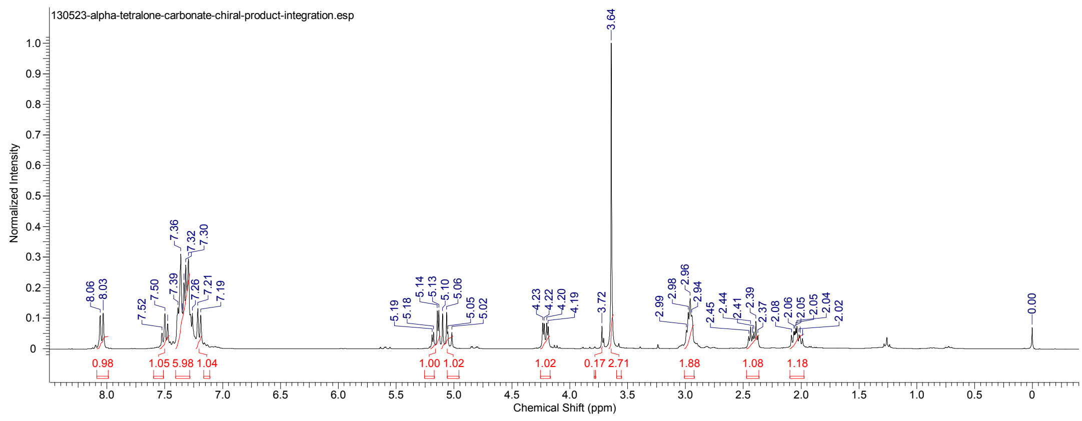
130523-alpha-tetralone-carbonate-chiral-product-integration.esp
Normalized Intensity
Chemical Shift (ppm)

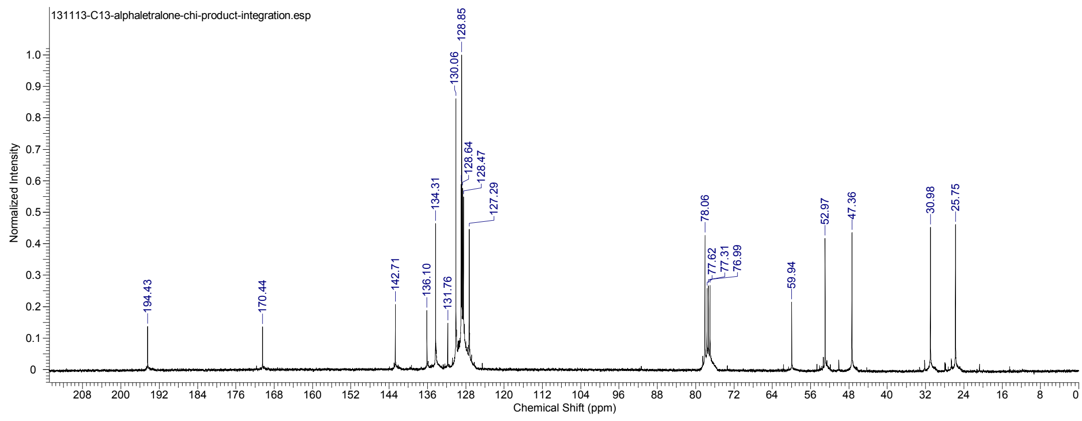
131113-C13-alphaletralone-chi-product-integration.esp
Normalized Intensity
Chemical Shift (ppm)

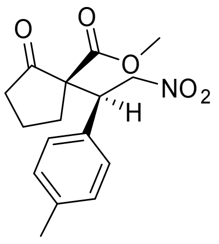
O
O
O
NO2
H

4i

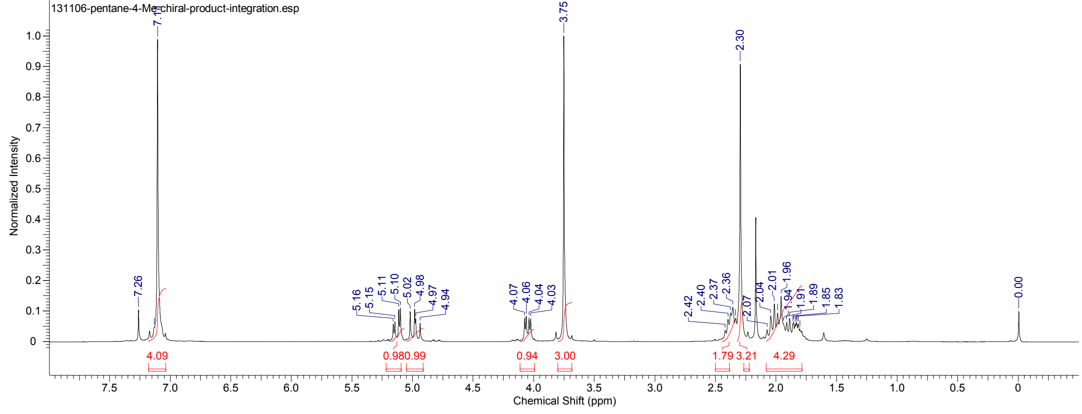
131106-pentane-4-Mechiral-product-integration.esp
Normalized Intensity
Chemical Shift (ppm)
7.26
3.75
2.30
5.16
5.15
5.11
5.10
5.02
4.98
4.97
4.94
4.07
4.06
4.04
4.03
2.42
2.40
2.37
2.36
2.07
2.04
2.01
1.96
1.94
1.91
1.89
1.85
1.83
0.00
4.09
0.980.99
0.94
3.00
1.79
3.21
4.29

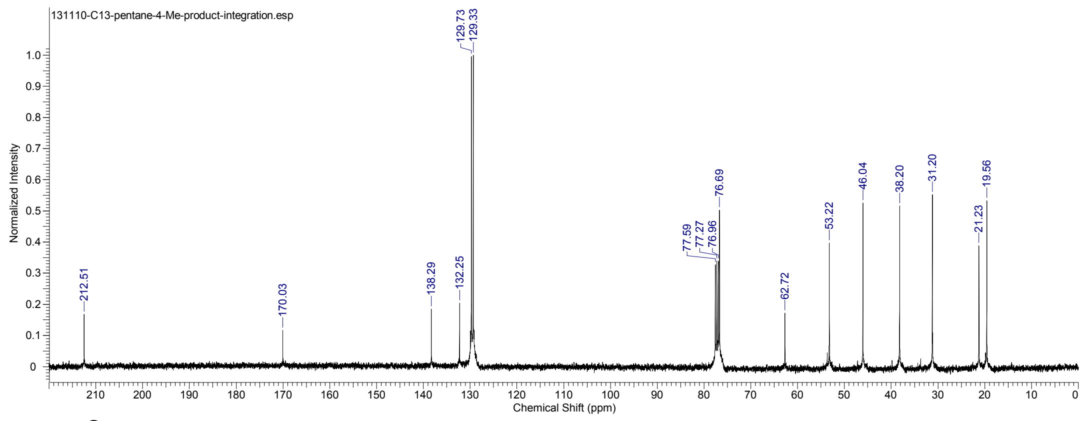
131110-C13-pentane-4-Me-product-integration.esp
Normalized Intensity
Chemical Shift (ppm)
212.51
170.03
138.29
132.25
129.73
129.33
77.59
77.27
76.96
76.69
62.72
53.22
46.04
38.20
31.20
21.23
19.56

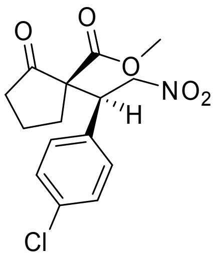
O
O
O
NO2
H
Cl

4j

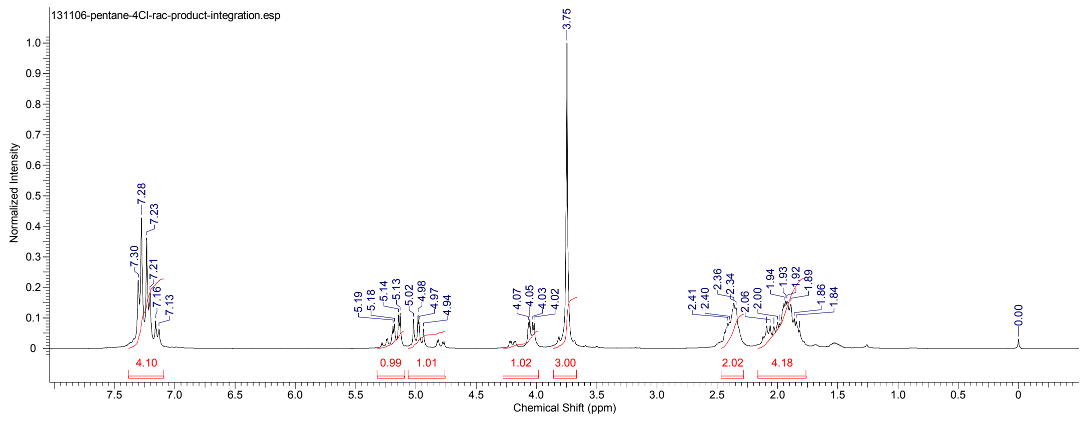
131106-pentane-4Cl-rac-product-integration.esp
Normalized Intensity
Chemical Shift (ppm)
7.30
7.28
7.23
7.21
7.16
7.13
5.19
5.18
5.14
5.13
5.02
4.98
4.97
4.94
4.07
4.05
4.03
4.02
3.75
2.41
2.40
2.36
2.34
2.06
2.00
1.94
1.93
1.92
1.89
1.86
1.84
0.00
4.10
0.99
1.01
1.02
3.00
2.02
4.18

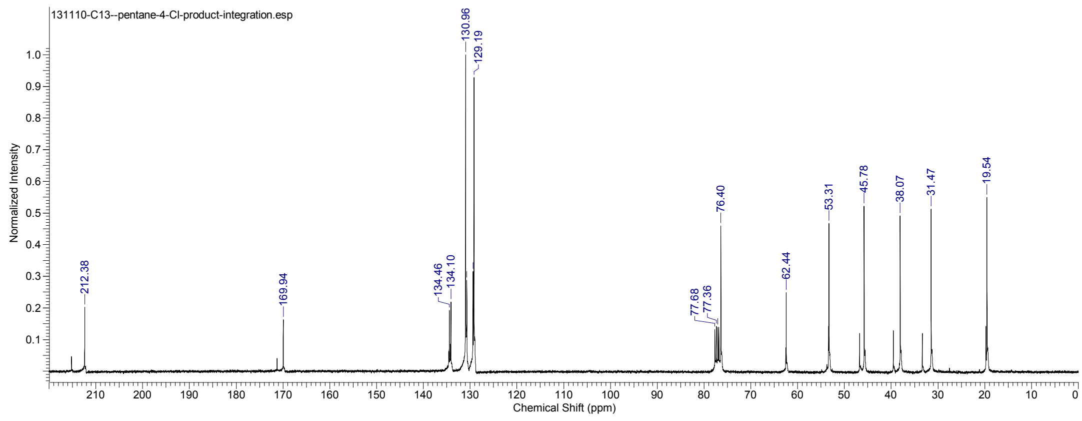
131110-C13--pentane-4-Cl-product-integration.esp
Normalized Intensity
Chemical Shift (ppm)
212.38
169.94
134.46
134.10
130.96
129.19
77.68
77.36
76.40
62.44
53.31
45.78
38.07
31.47
19.54

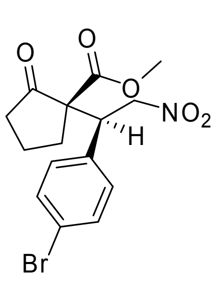
O
O
O
NO2
H
Br

4k

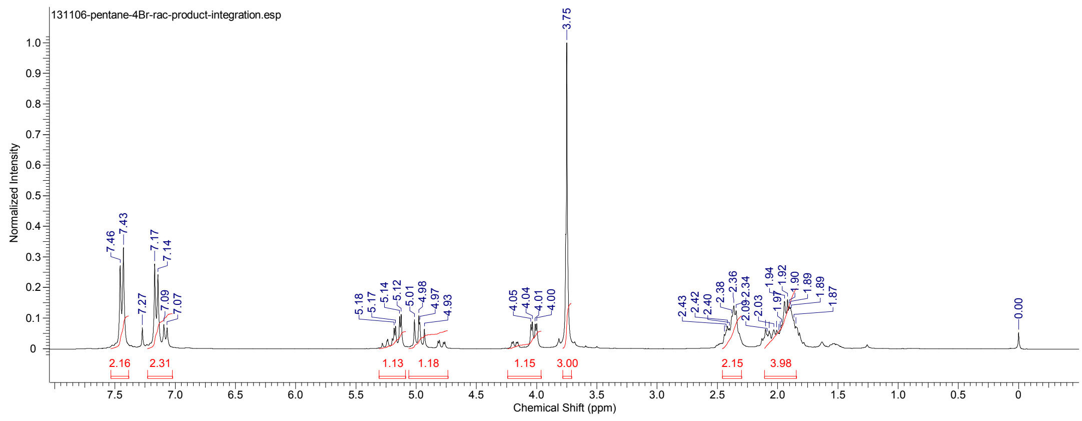
131106-pentane-4Br-rac-product-integration.esp
Normalized Intensity
Chemical Shift (ppm)
7.46
7.43
7.27
7.17
7.14
7.09
7.07
5.18
5.17
5.14
5.12
5.01
4.98
4.97
4.93
4.05
4.04
4.01
4.00
3.75
2.43
2.42
2.40
2.38
2.36
2.34
2.09
2.03
1.97
1.94
1.92
1.90
1.89
1.87
0.00
2.16
2.31
1.13
1.18
1.15
3.00
2.15
3.98

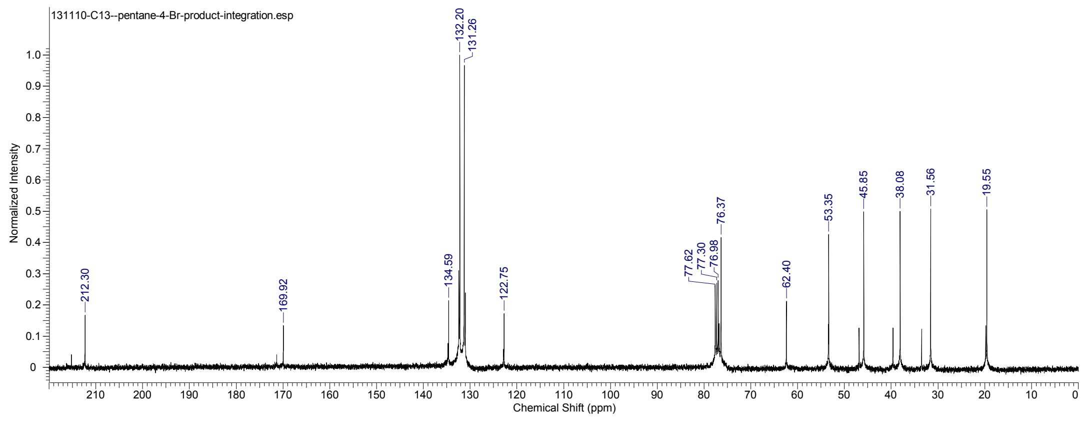
131110-C13--pentane-4-Br-product-integration.esp
Normalized Intensity
Chemical Shift (ppm)
212.30
169.92
134.59
132.20
131.26
122.75
77.62
77.30
76.98
76.37
62.40
53.35
45.85
38.08
31.56
19.55

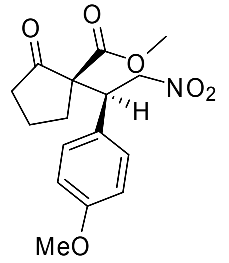
MeO
NO2
H

4l

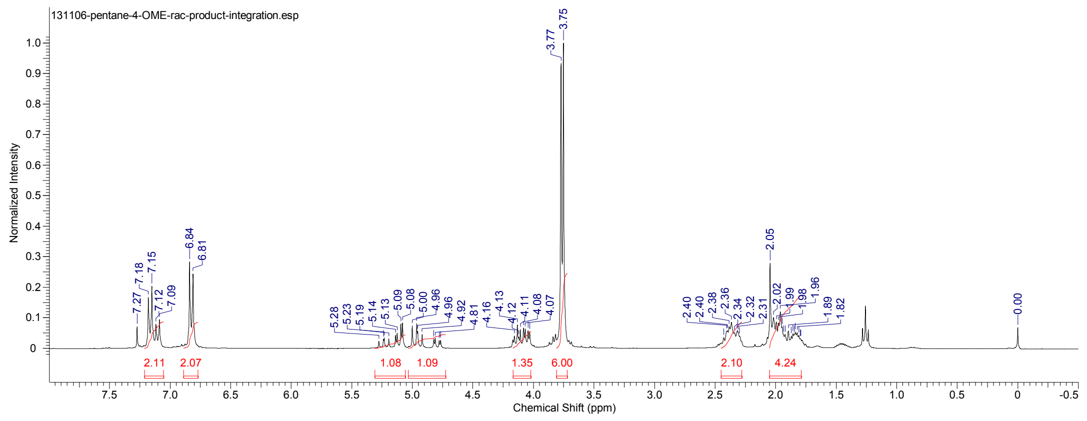
131106-pentane-4-OME-rac-product-integration.esp
Normalized Intensity
Chemical Shift (ppm)

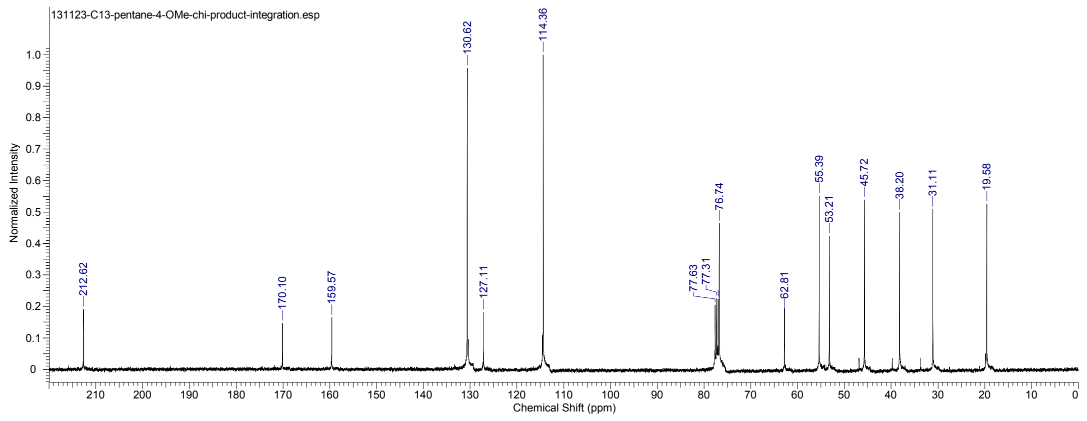
131123-C13-pentane-4-OMe-chi-product-integration.esp
Normalized Intensity
Chemical Shift (ppm)

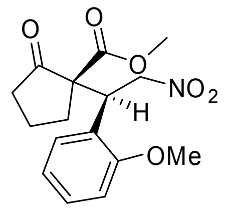
NO2
H
OMe

4m

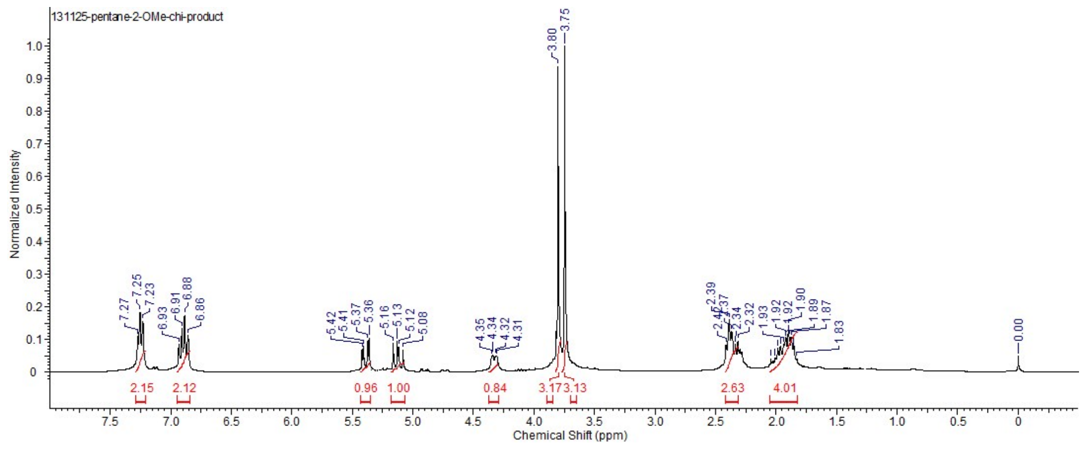
131125-pentane-2-OMe-chi-product
Normalized Intensity
Chemical Shift (ppm)

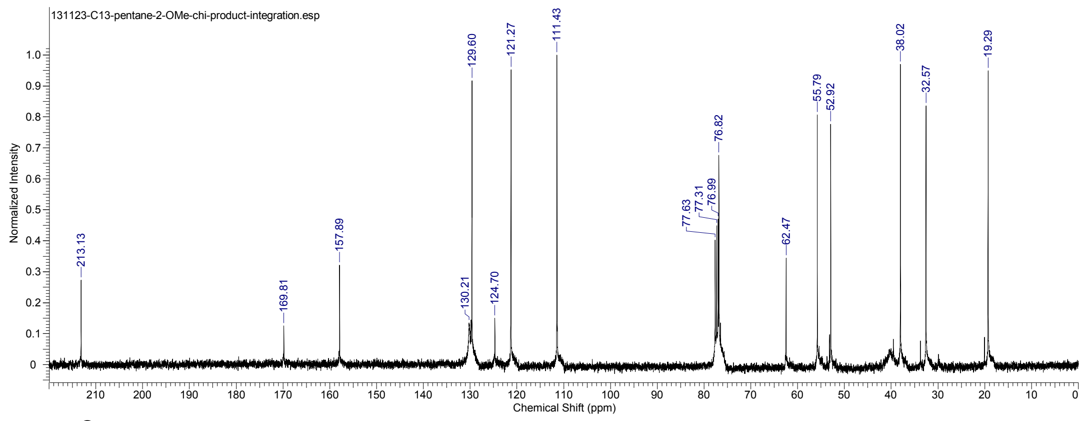
131123-C13-pentane-2-OMe-chi-product-integration.esp
Normalized Intensity
Chemical Shift (ppm)

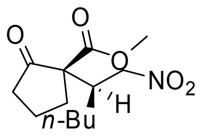
NO2
H
n-Bu

4n

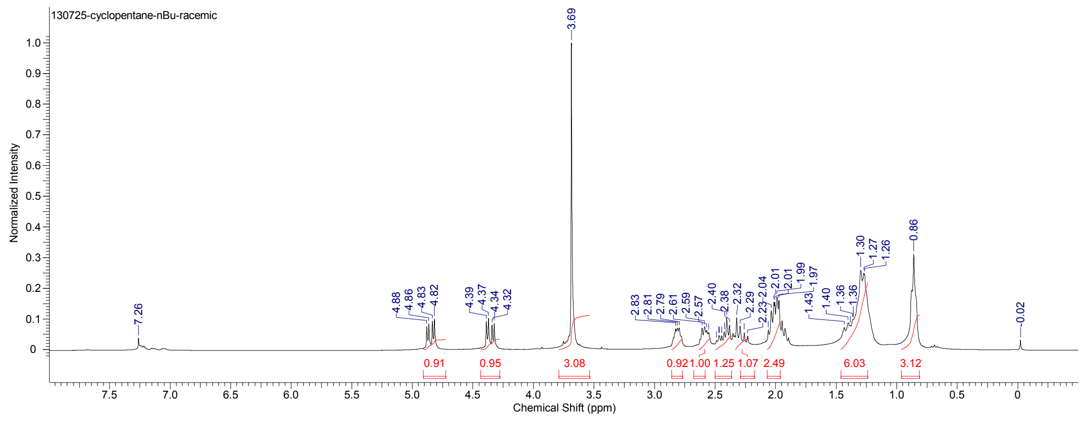
130725-cyclopentane-nBu-racemic
Normalized Intensity
Chemical Shift (ppm)

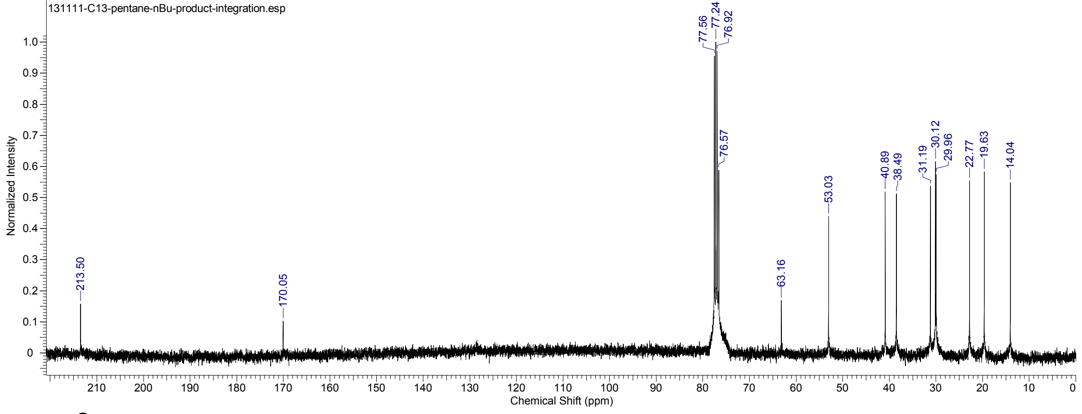
131111-C13-pentane-nBu-product-integration.esp
Normalized Intensity
Chemical Shift (ppm)
213.50
170.05
77.56
77.24
76.92
76.57
63.16
53.03
40.89
38.49
31.19
30.12
29.96
22.77
19.63
14.04

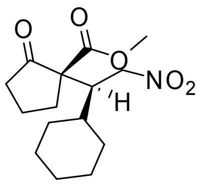

4o

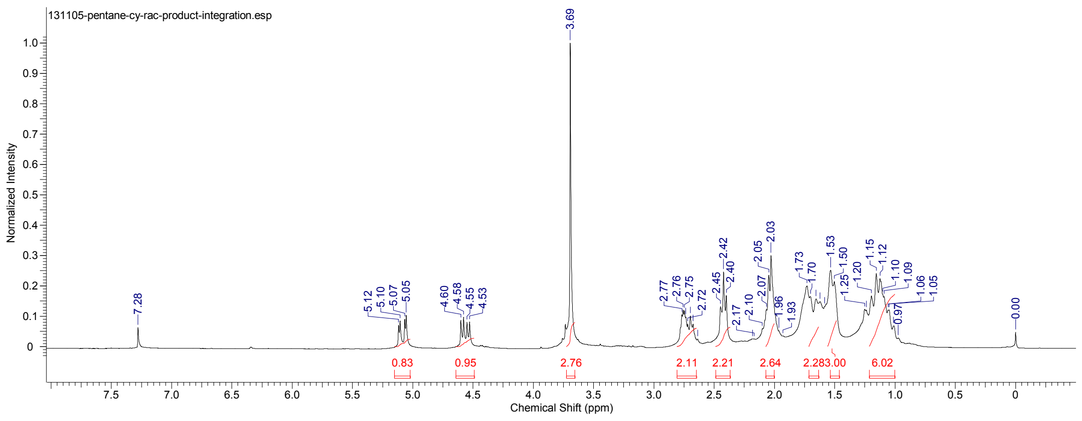
131105-pentane-cy-rac-product-integration.esp
Normalized Intensity
Chemical Shift (ppm)
3.69
7.28
5.12
5.10
5.07
5.05
4.60
4.58
4.55
4.53
2.77
2.76
2.75
2.72
2.45
2.42
2.40
2.17
2.10
2.07
2.05
2.03
1.96
1.93
1.73
1.70
1.53
1.50
1.25
1.20
1.15
1.12
1.10
1.09
1.06
1.05
0.97
0.00
0.83
0.95
2.76
2.11
2.21
2.64
2.28
3.00
6.02

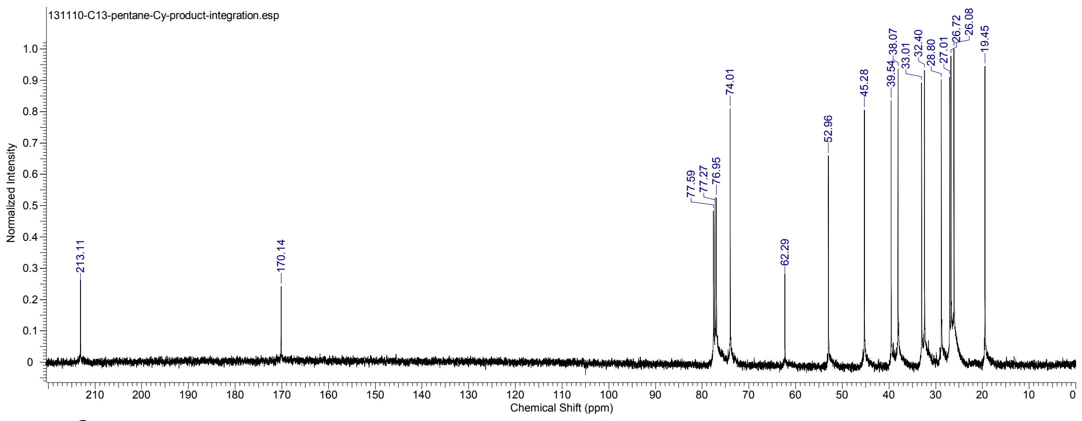
131110-C13-pentane-Cy-product-integration.esp
Normalized Intensity
Chemical Shift (ppm)
213.11
170.14
77.59
77.27
76.95
74.01
62.29
52.96
45.28
39.54
38.07
33.01
32.40
28.80
27.01
26.72
26.08
19.45

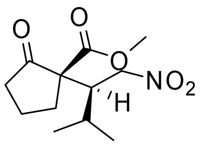

4p

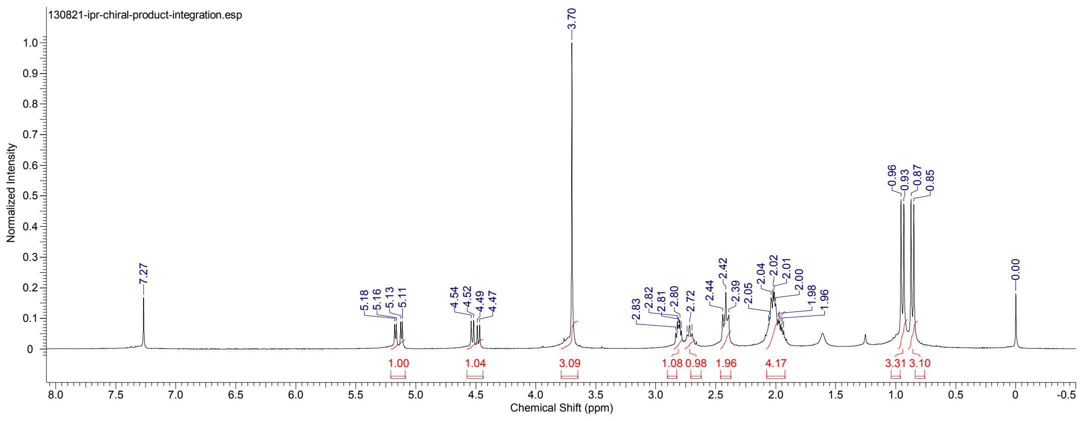
130821-ipr-chiral-product-integration.esp
Normalized Intensity
Chemical Shift (ppm)
3.70
7.27
5.18
5.16
5.13
5.11
4.54
4.52
4.49
4.47
2.83
2.82
2.81
2.80
2.72
2.44
2.42
2.39
2.05
2.04
2.02
2.01
2.00
1.98
1.96
0.96
0.93
0.87
0.85
0.00
1.00
1.04
3.09
1.08
0.98
1.96
4.17
3.31
3.10

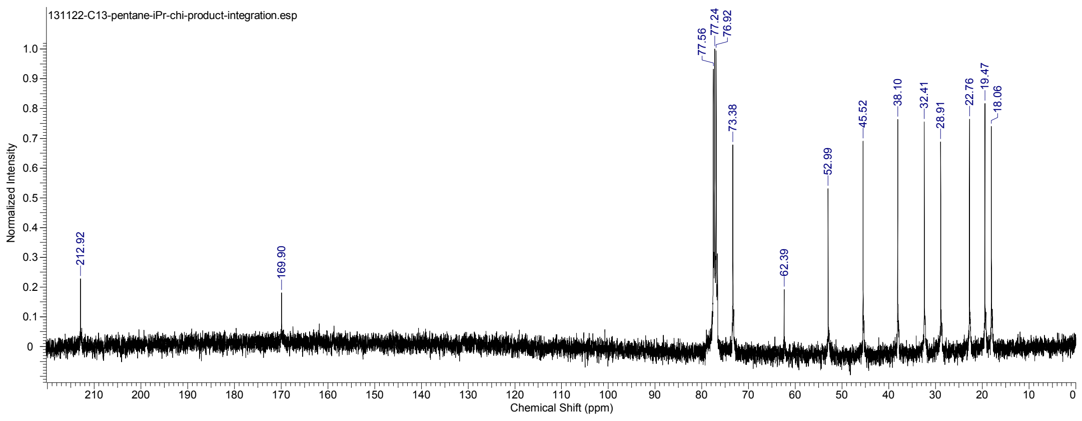
131122-C13-pentane-iPr-chi-product-integration.esp
Normalized Intensity
Chemical Shift (ppm)
212.92
169.90
77.56
77.24
76.92
73.38
62.39
52.99
45.52
38.10
32.41
28.91
22.76
19.47
18.06

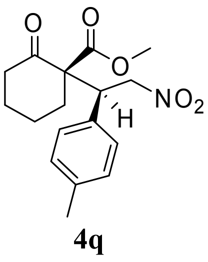

4q

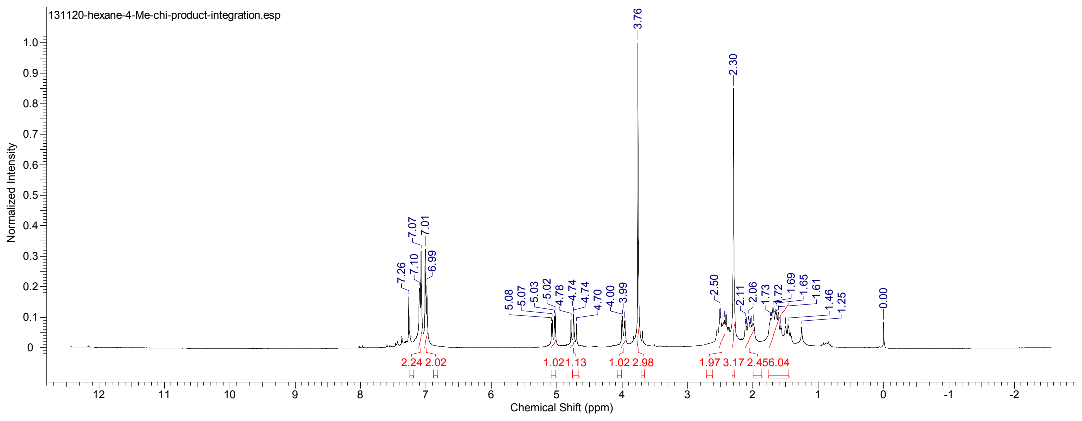
131120-hexane-4-Me-chi-product-integration.esp
Normalized Intensity
Chemical Shift (ppm)
3.76
2.30
7.26
7.10
7.07
7.01
6.99
5.08
5.07
5.03
5.02
4.78
4.74
4.74
4.70
4.00
3.99
2.50
2.11
2.06
1.73
1.72
1.69
1.65
1.61
1.46
1.25
0.00
2.24
2.02
1.02
1.13
1.02
2.98
1.97
3.17
2.45
6.04

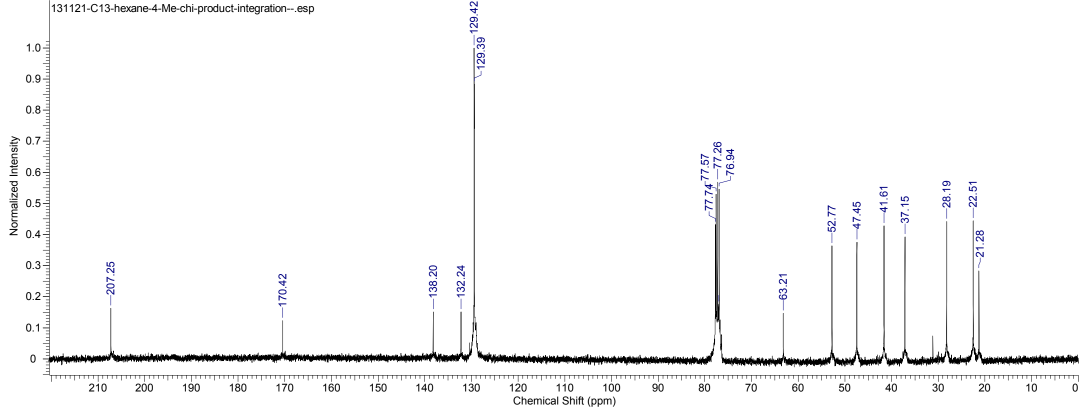
131121-C13-hexane-4-Me-chi-product-integration--.esp
Normalized Intensity
Chemical Shift (ppm)

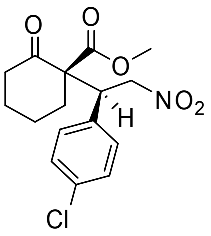
O
O
O
NO2
H
Cl

4r

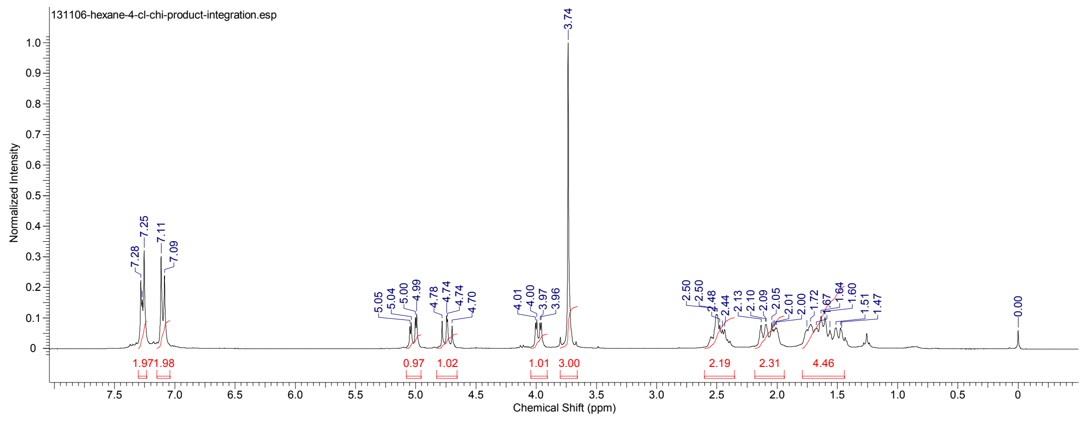
131106-hexane-4-cl-chi-product-integration.esp
Normalized Intensity
Chemical Shift (ppm)

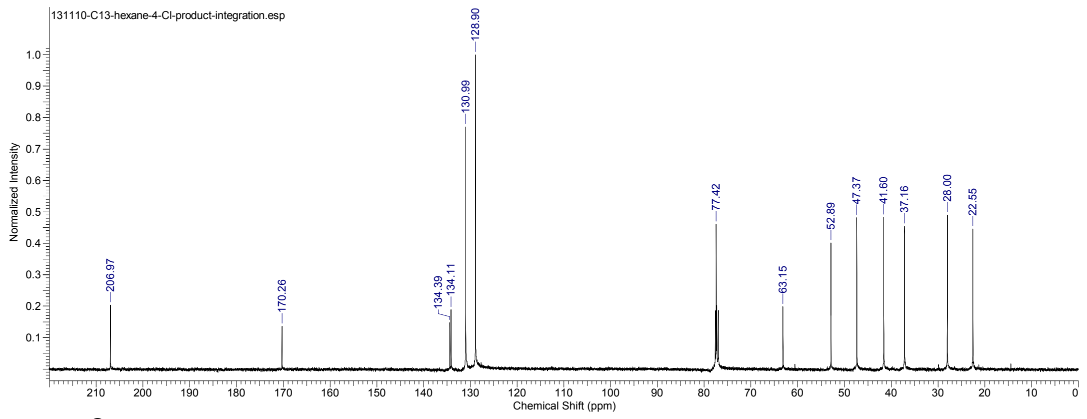
131110-C13-hexane-4-Cl-product-integration.esp
Normalized Intensity
Chemical Shift (ppm)

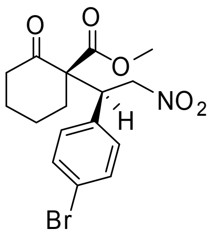
O
O
O
NO2
H
Br

4s

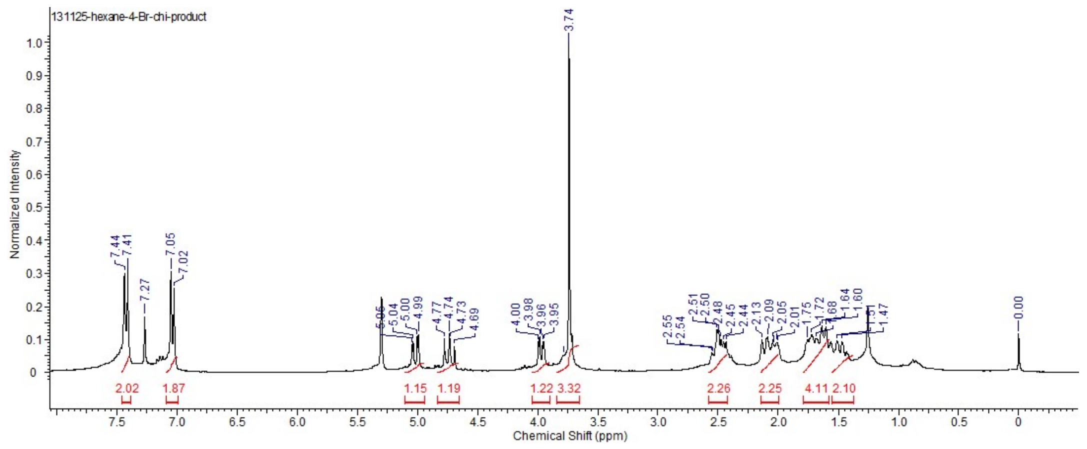
131125-hexane-4-Br-chi-product
Normalized Intensity
Chemical Shift (ppm)

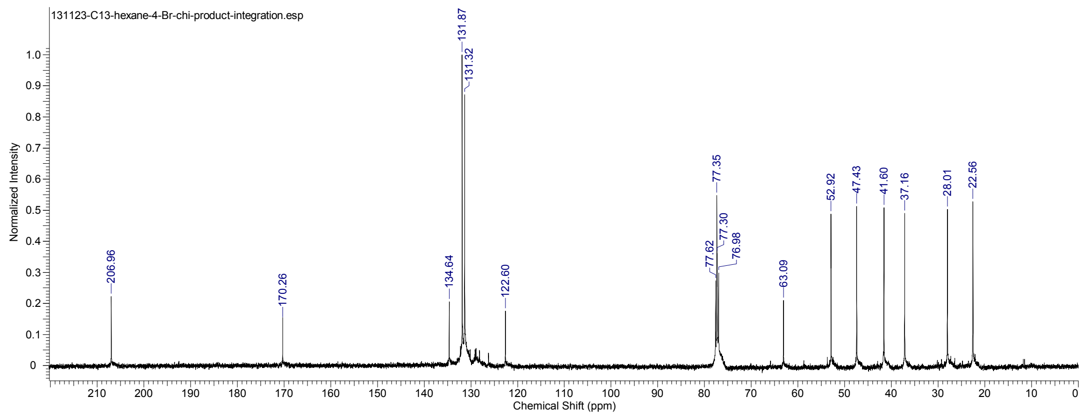
131123-C13-hexane-4-Br-chi-product-integration.esp
Normalized Intensity
Chemical Shift (ppm)

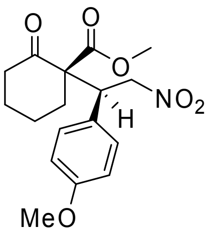
O
O
O
NO2
H
MeO

4t

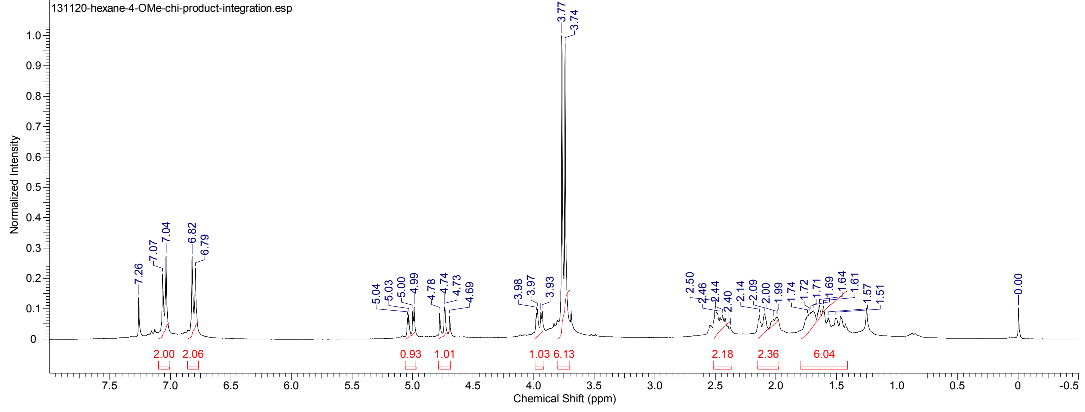
131120-hexane-4-OMe-chi-product-integration.esp
Normalized Intensity
Chemical Shift (ppm)
7.26
7.07
7.04
6.82
6.79
5.04
5.03
5.00
4.99
4.78
4.74
4.73
4.69
3.98
3.97
3.93
3.77
3.74
2.50
2.46
2.44
2.40
2.14
2.09
2.00
1.99
1.74
1.72
1.71
1.69
1.64
1.61
1.57
1.51
0.00
2.00
2.06
0.93
1.01
1.03
6.13
2.18
2.36
6.04

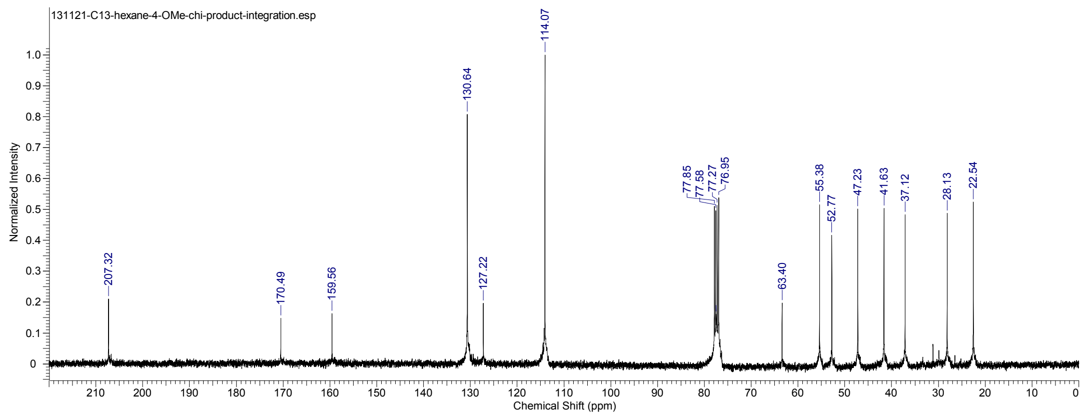
131121-C13-hexane-4-OMe-chi-product-integration.esp
Normalized Intensity
Chemical Shift (ppm)
207.32
170.49
159.56
130.64
127.22
114.07
77.85
77.58
77.27
76.95
63.40
55.38
52.77
47.23
41.63
37.12
28.13
22.54

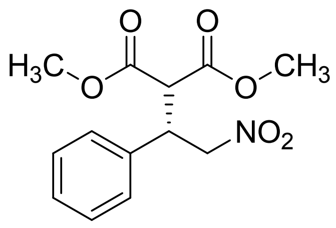
H3C
O
O
O
O
CH3
NO2

5a

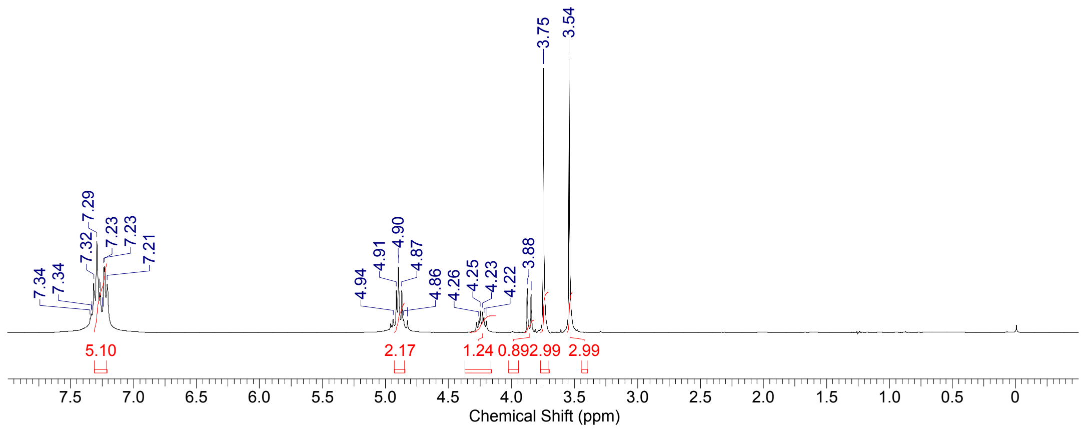
7.34
7.34
7.32
7.29
7.23
7.23
7.21
4.94
4.91
4.90
4.87
4.86
4.26
4.25
4.23
4.22
3.88
3.75
3.54
5.10
2.17
1.24
0.89
2.99
2.99
Chemical Shift (ppm)

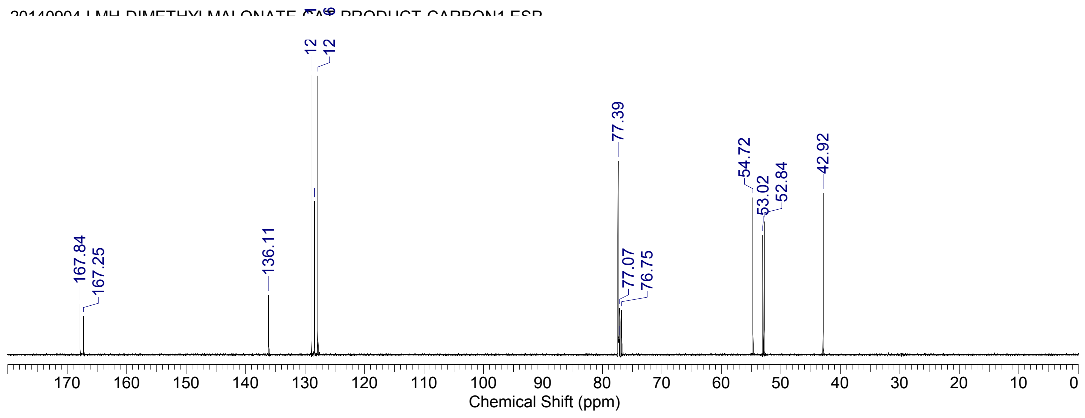
167.84
167.25
136.11
77.39
77.07
76.75
54.72
53.02
52.84
42.92
Chemical Shift (ppm)

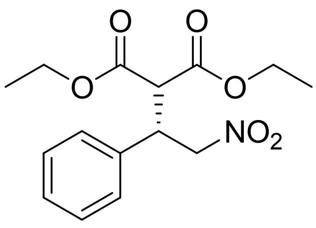
O
O
O
O
NO2

5b

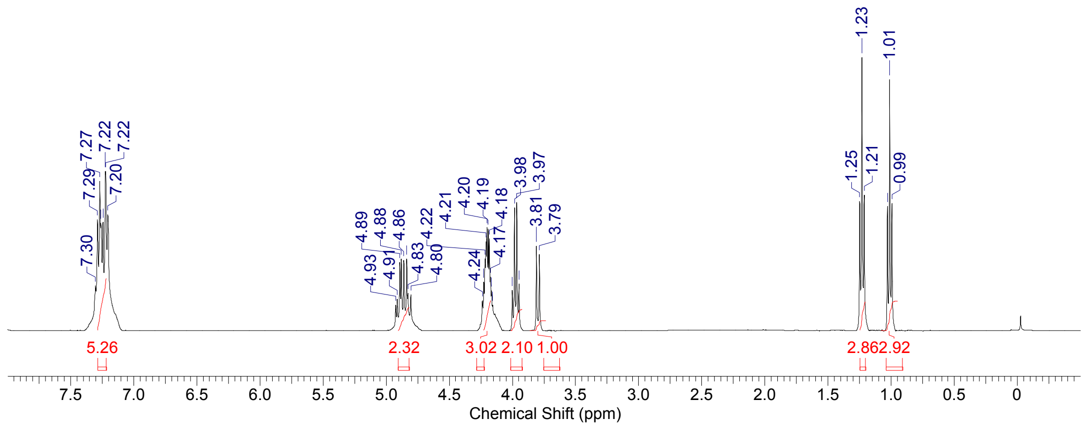
7.30
7.29
7.27
7.22
7.22
7.20
4.93
4.91
4.89
4.88
4.86
4.83
4.80
4.24
4.22
4.21
4.20
4.19
4.18
4.17
3.98
3.97
3.81
3.79
1.25
1.23
1.21
1.01
0.99
5.26
2.32
3.02
2.10
1.00
2.86
2.92
Chemical Shift (ppm)

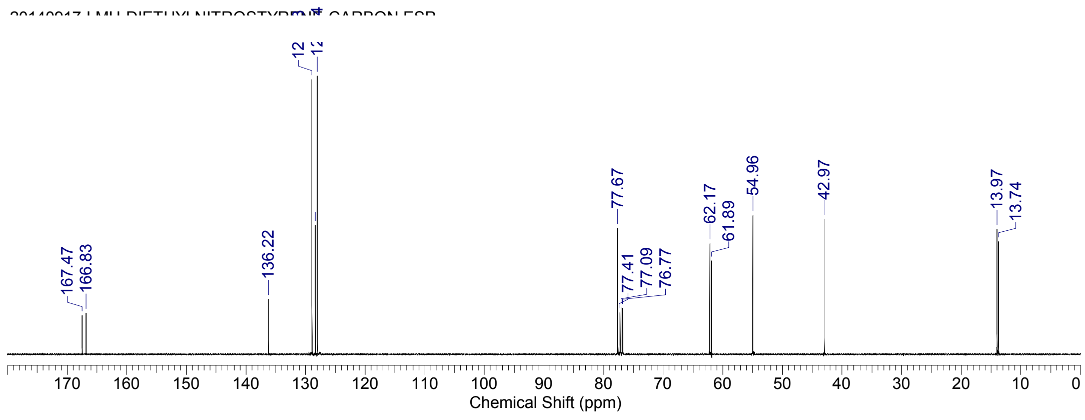
167.47
166.83
136.22
77.67
77.41
77.09
76.77
62.17
61.89
54.96
42.97
13.97
13.74
Chemical Shift (ppm)

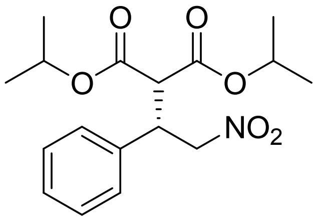
NO2

5c

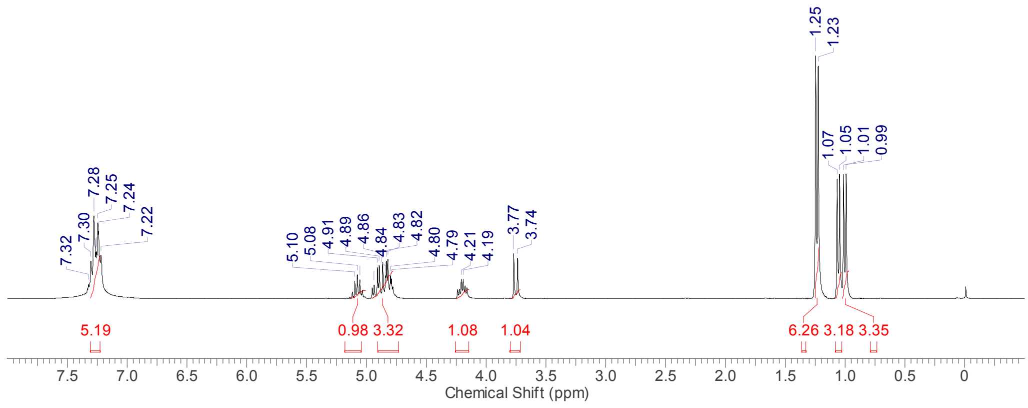
7.32
7.30
7.28
7.25
7.24
7.22
5.10
5.08
4.91
4.89
4.86
4.84
4.83
4.82
4.80
4.79
4.21
4.19
3.77
3.74
1.25
1.23
1.07
1.05
1.01
0.99
5.19
0.98
3.32
1.08
1.04
6.26
3.18
3.35
Chemical Shift (ppm)

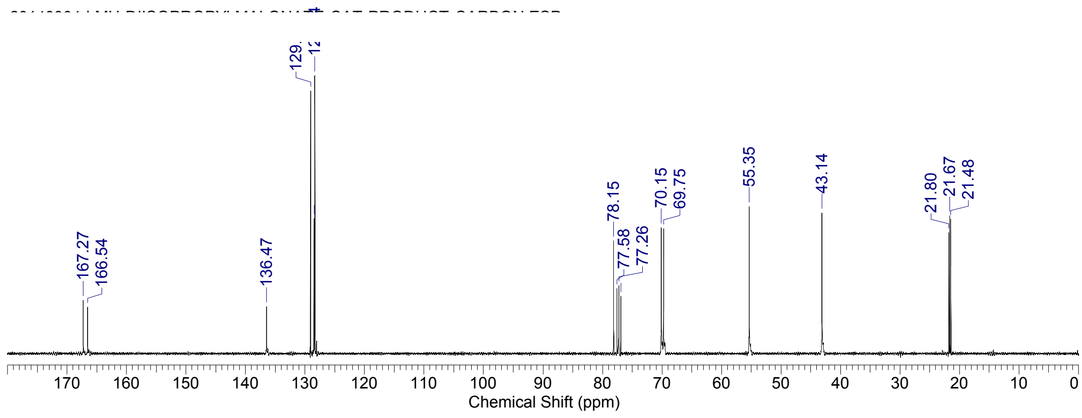
167.27
166.54
136.47
78.15
77.58
77.26
70.15
69.75
55.35
43.14
21.80
21.67
21.48
Chemical Shift (ppm)

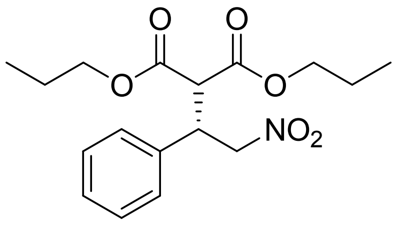
NO2

5d

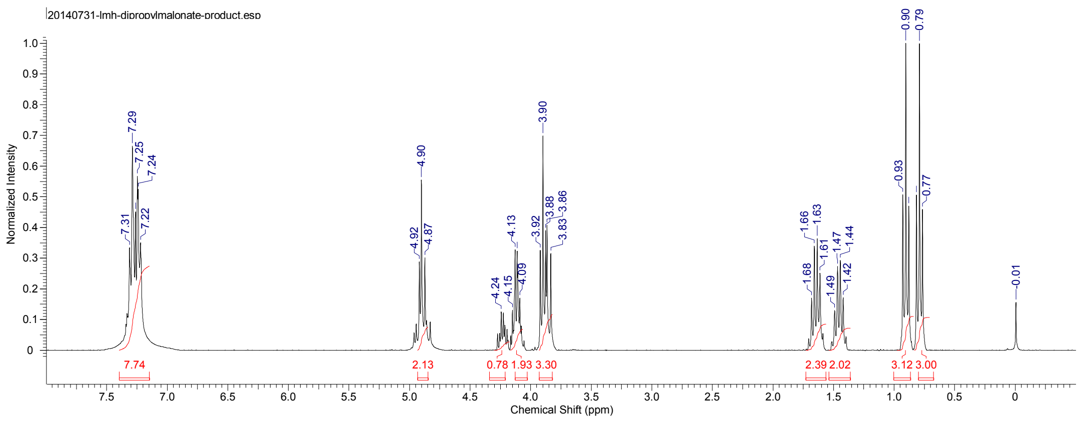
20140731-lmh-dipropylmalonate-product.esp
Normalized Intensity
7.31
7.29
7.25
7.24
7.22
4.92
4.90
4.87
4.24
4.15
4.13
4.09
3.92
3.90
3.88
3.86
3.83
1.68
1.66
1.63
1.61
1.49
1.47
1.44
1.42
0.93
0.90
0.79
0.77
0.01
7.74
2.13
0.78
1.93
3.30
2.39
2.02
3.12
3.00
Chemical Shift (ppm)

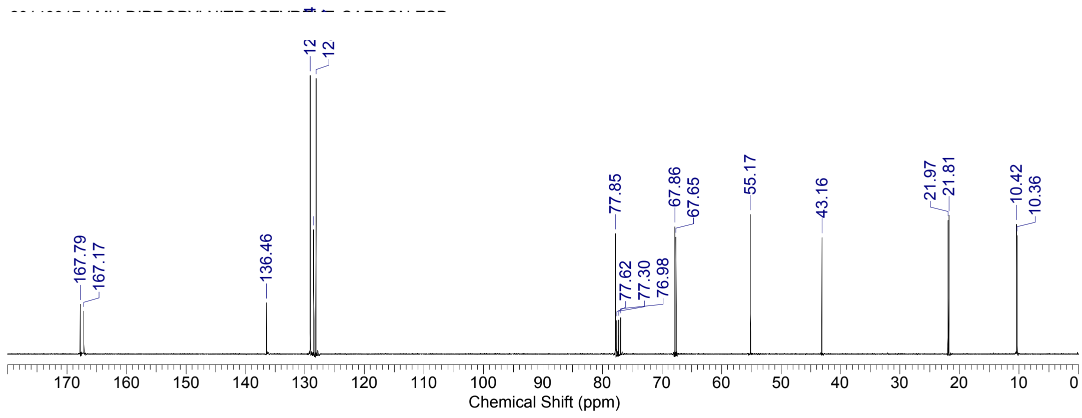
167.79
167.17
136.46
77.85
77.62
77.30
76.98
67.86
67.65
55.17
43.16
21.97
21.81
10.42
10.36
Chemical Shift (ppm)

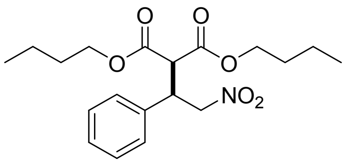
NO2

5e

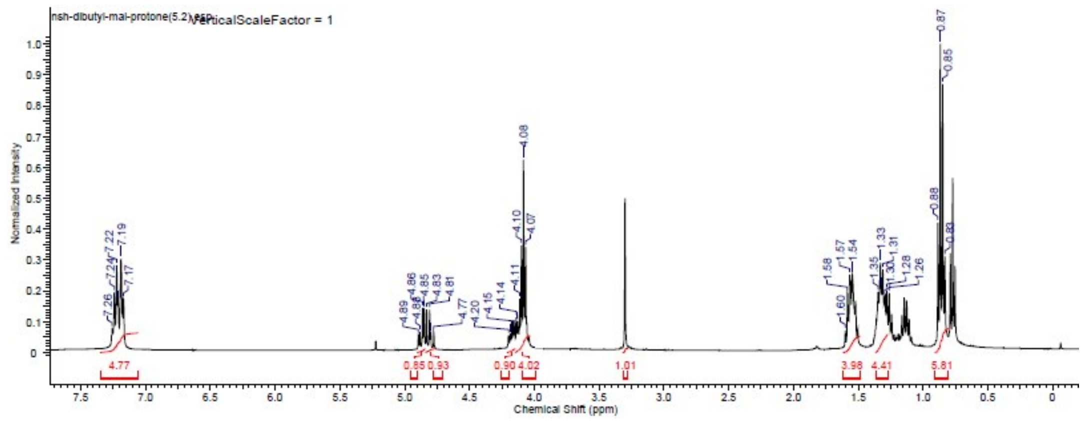
VerticalScaleFactor = 1
Normalized Intensity
7.22
7.19
7.17
4.86
4.85
4.83
4.81
4.77
4.15
4.14
4.11
4.10
4.08
4.07
1.58
1.57
1.54
1.35
1.33
1.31
1.28
1.26
0.88
0.87
0.85
4.77
0.85
0.93
0.90
4.02
1.01
3.98
4.41
5.81
Chemical Shift (ppm)

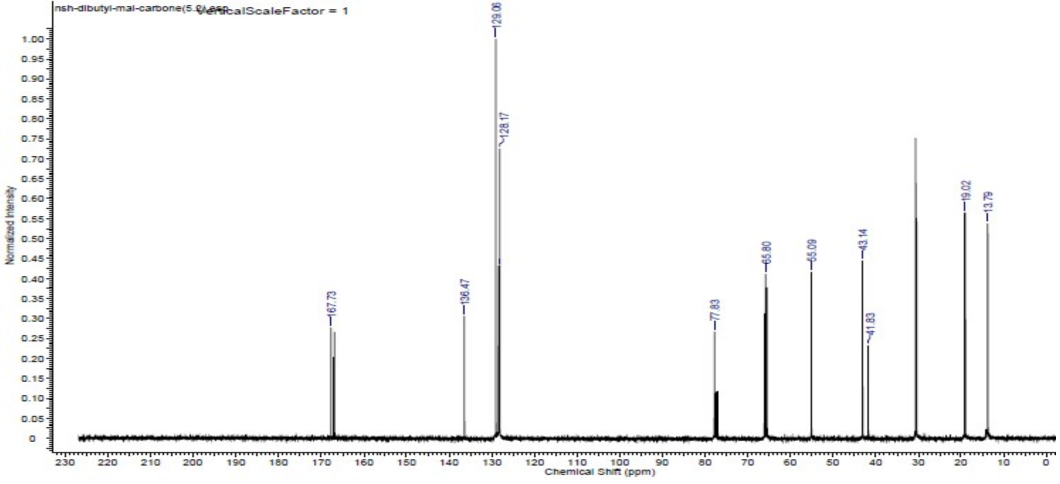
Chemical Shift (ppm)

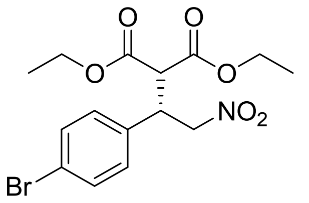

5f

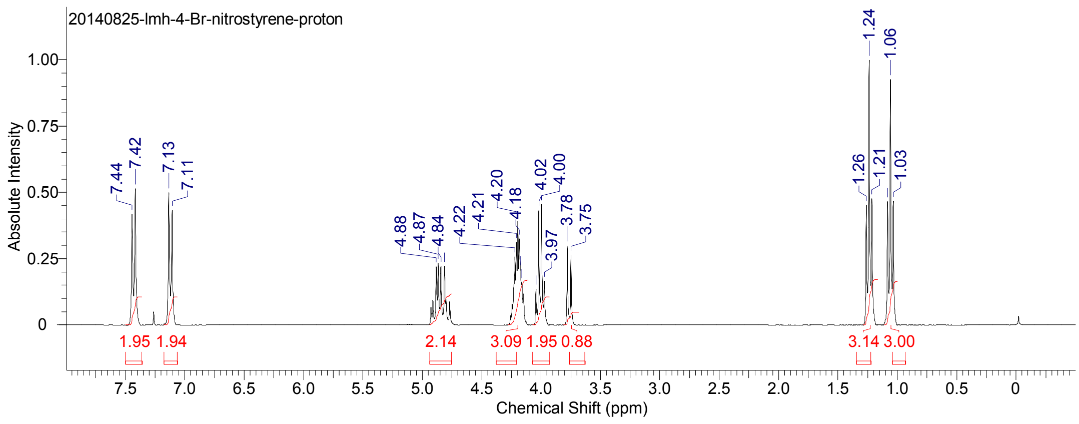
20140825-lmh-4-Br-nitrostyrene-proton
Absolute Intensity
Chemical Shift (ppm)

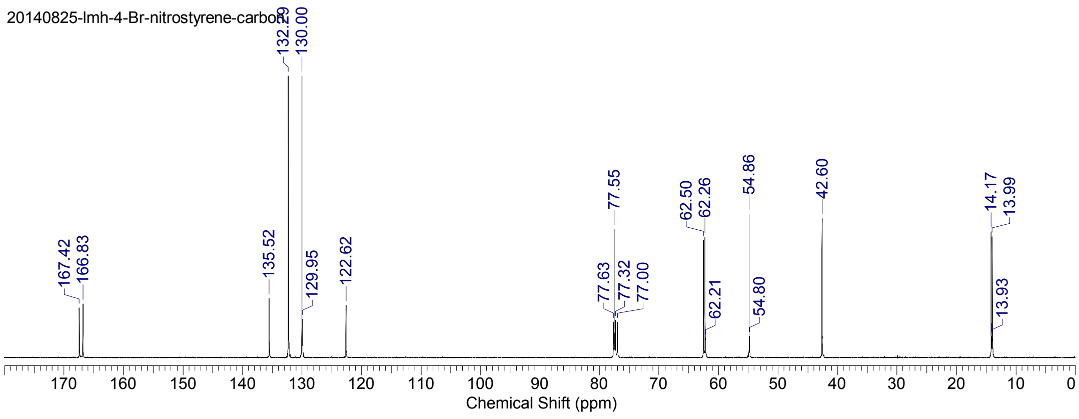
Chemical Shift (ppm)

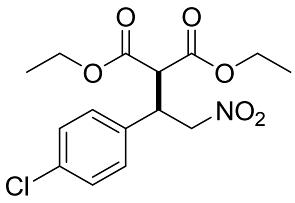

5g

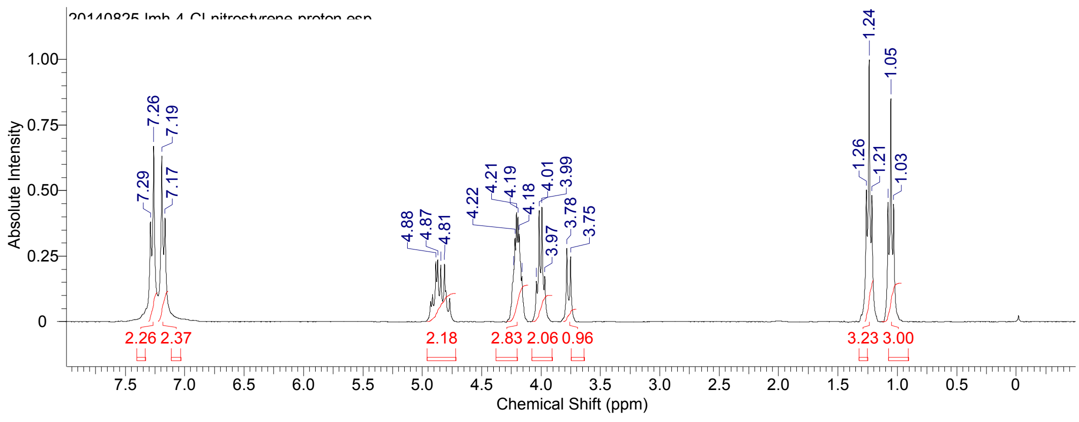
Absolute Intensity
Chemical Shift (ppm)

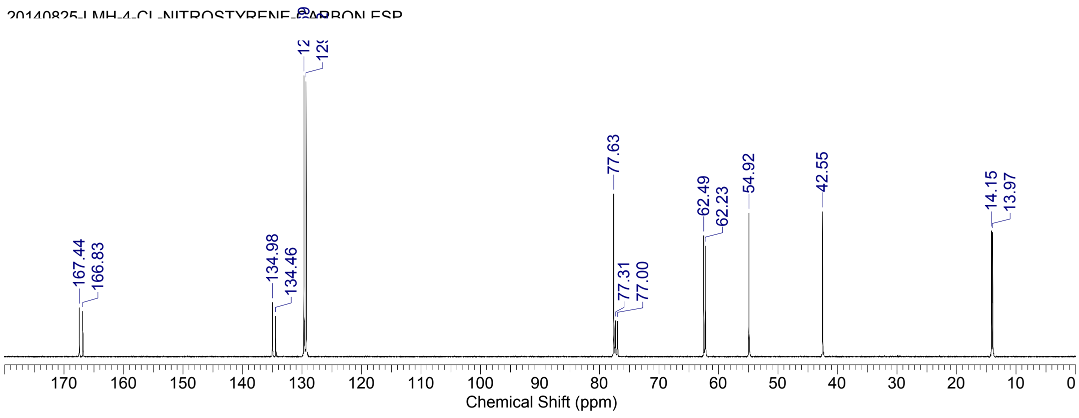
Chemical Shift (ppm)

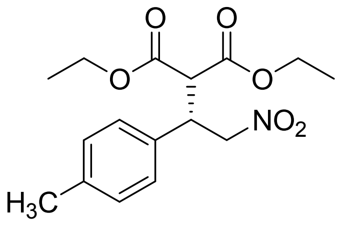

5h

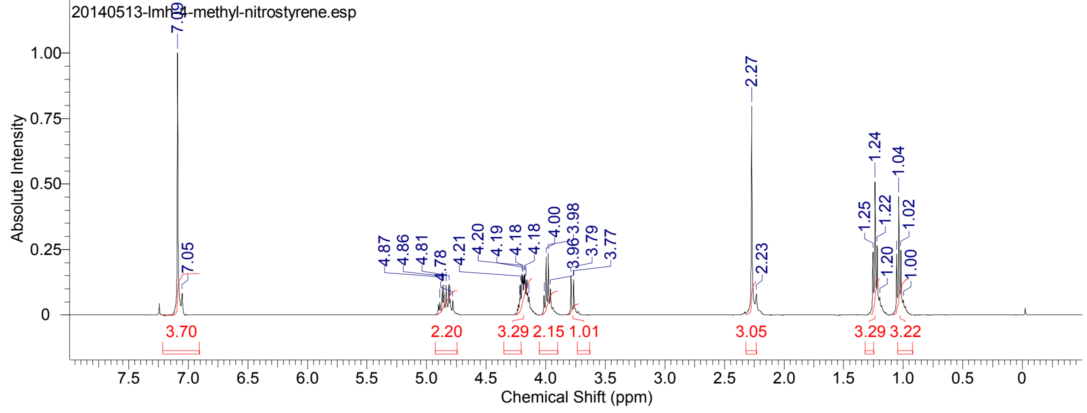
Absolute Intensity
Chemical Shift (ppm)

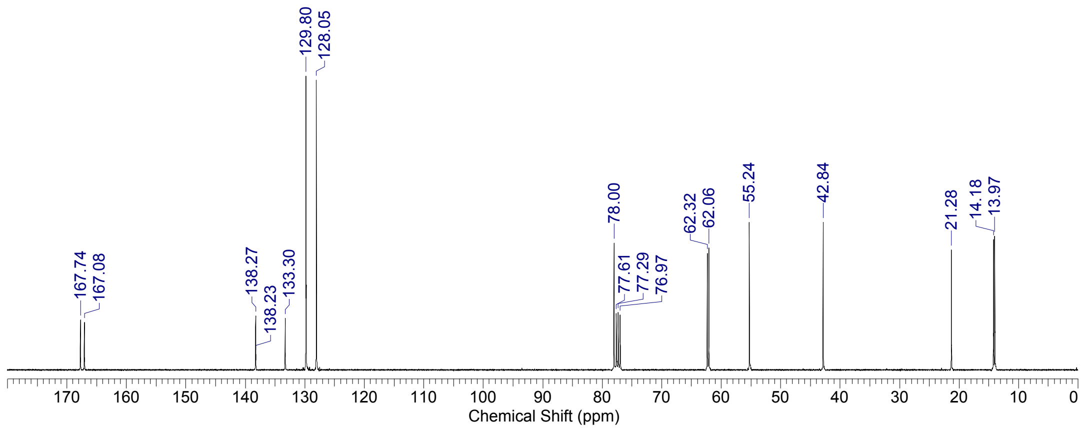
Chemical Shift (ppm)

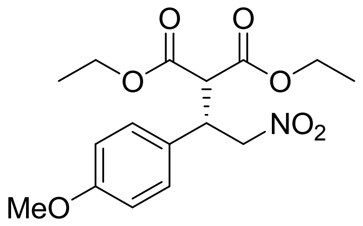
MeO
NO2

5i

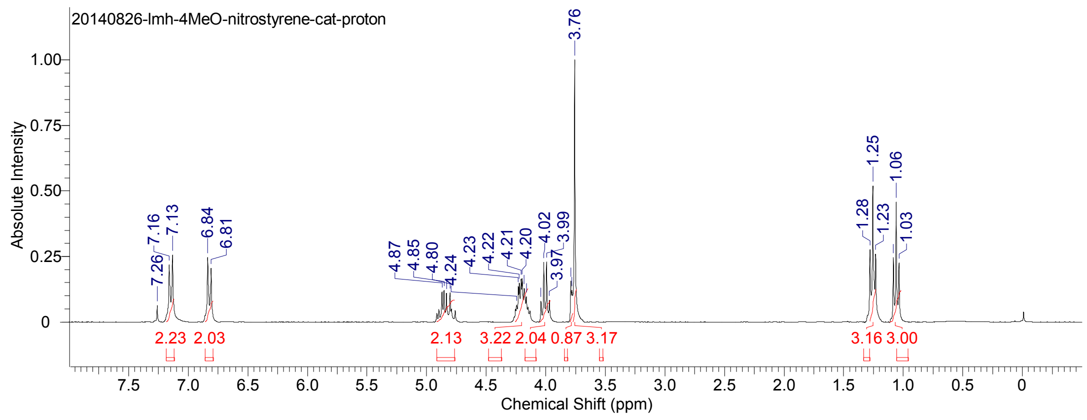
20140826-lmh-4MeO-nitrostyrene-cat-proton
Absolute Intensity
Chemical Shift (ppm)

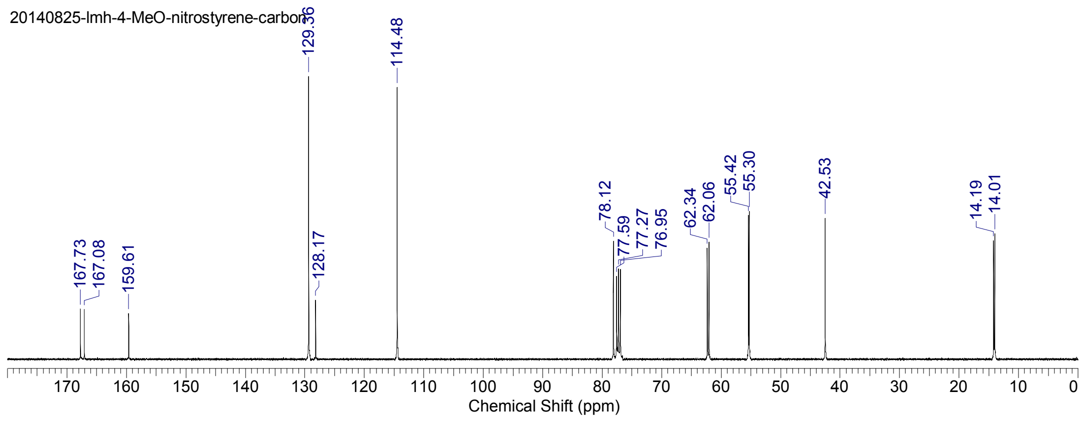
20140825-lmh-4-MeO-nitrostyrene-carbon
Chemical Shift (ppm)

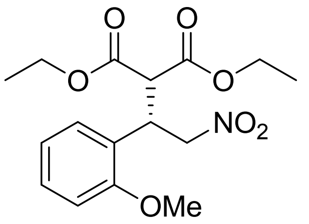
NO2
OMe

5j

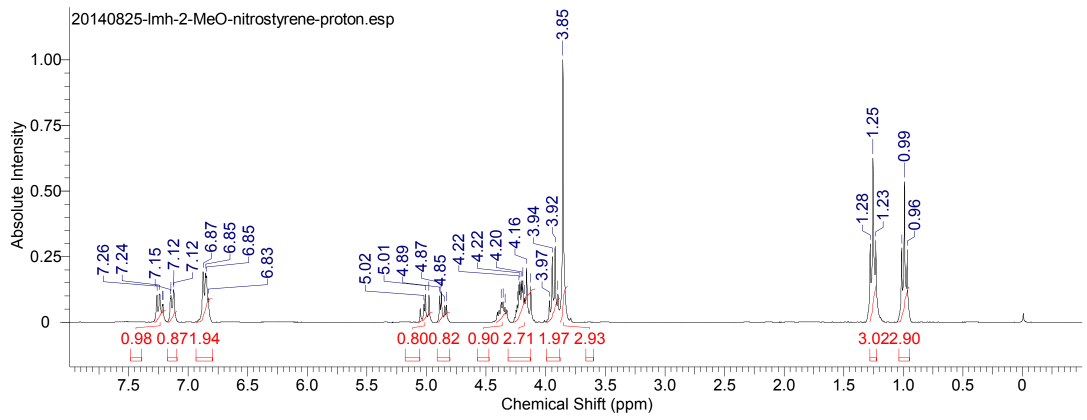
20140825-lmh-2-MeO-nitrostyrene-proton.esp
Absolute Intensity
Chemical Shift (ppm)

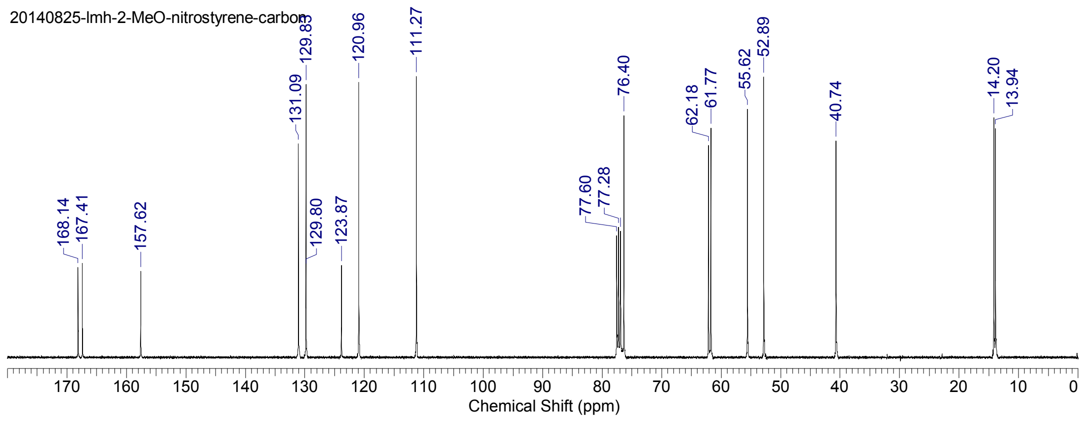
20140825-lmh-2-MeO-nitrostyrene-carbon
Chemical Shift (ppm)

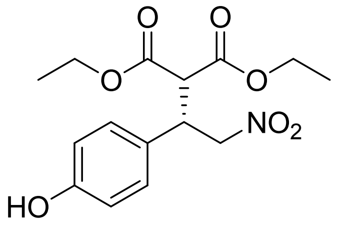

5k

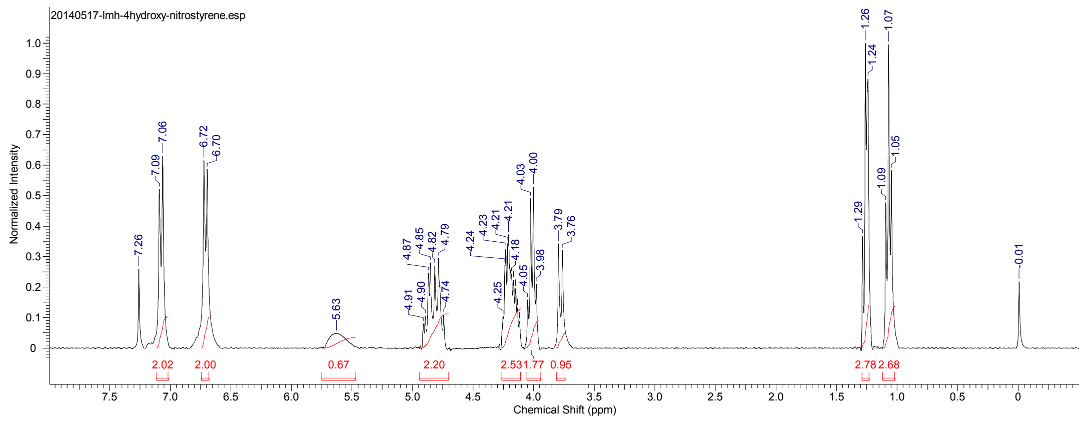
20140517-lmh-4hydroxy-nitrostyrene.esp
Normalized Intensity
Chemical Shift (ppm)

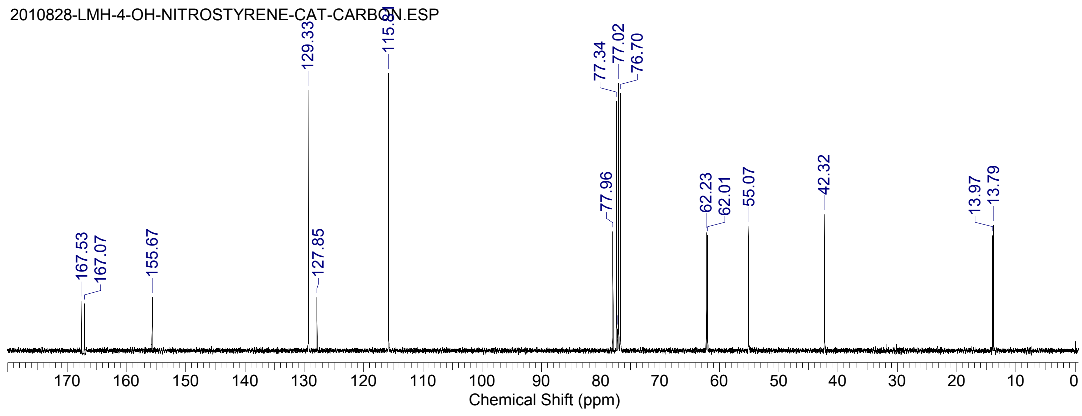
2010828-LMH-4-OH-NITROSTYRENE-CAT-CARBON.ESP
Chemical Shift (ppm)

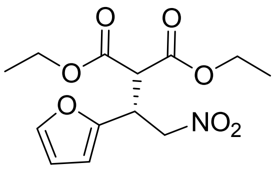

5l

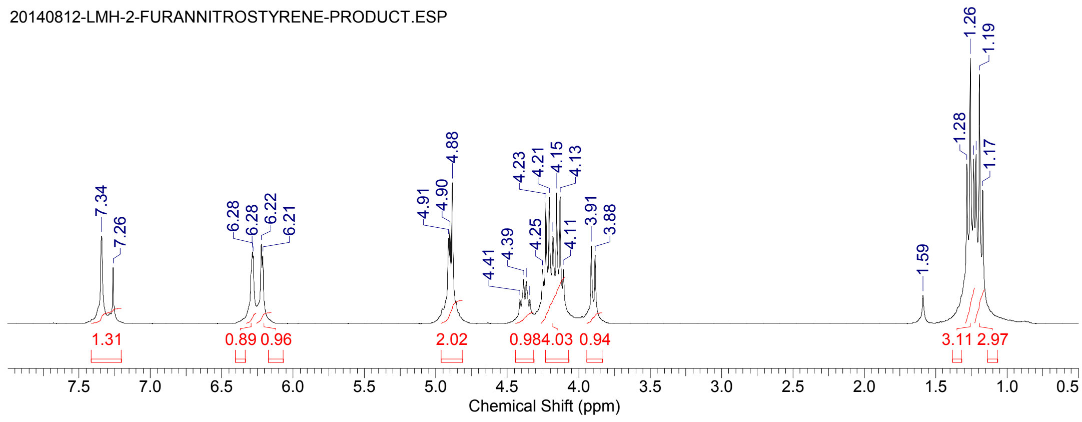
20140812-LMH-2-FURANNITROSTYRENE-PRODUCT.ESP
Chemical Shift (ppm)

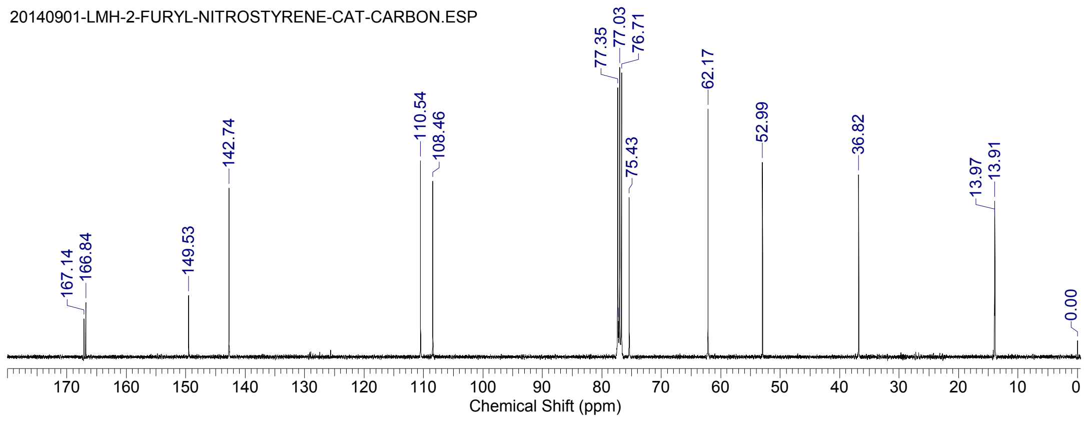
20140901-LMH-2-FURYL-NITROSTYRENE-CAT-CARBON.ESP
Chemical Shift (ppm)

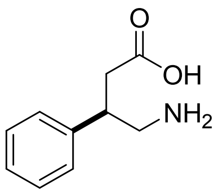

6a

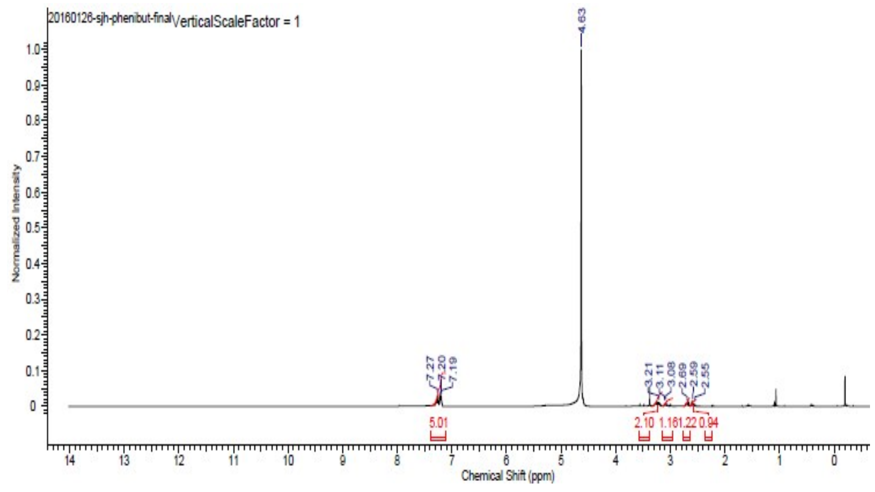
VerticalScaleFactor = 1
Normalized Intensity
Chemical Shift (ppm)

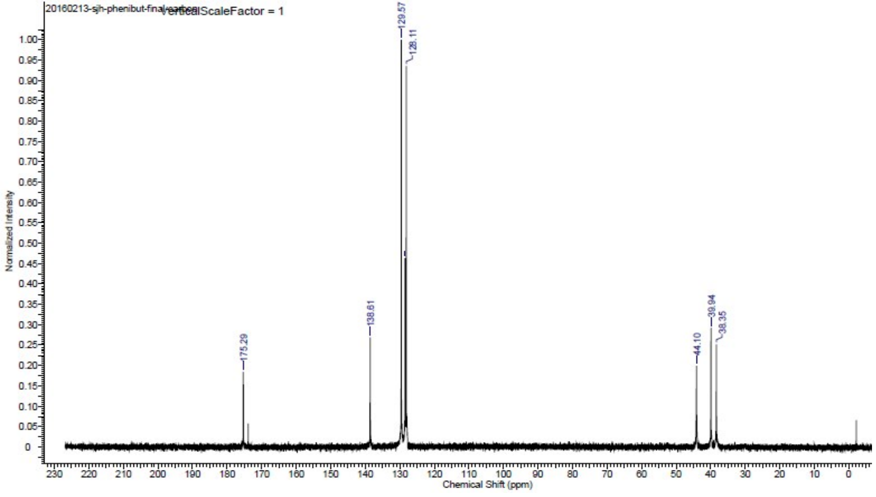

MASS Spectra

1-[(1*R*,2*R*)-2-Amino-1,2-diphenylethyl]-3-phenylthiourea (1a)

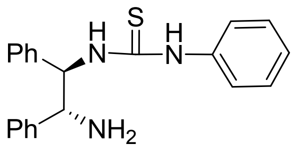

HRMS (FAB$^+$) for $C_{21}H_{22}N_3S$ [M+H]$^+$ Calcd: 348.1534, Found: 348.1530.

```
[ Elemental Composition ]
Data   : HFAB20080507-008              Date : 07-May-108 21:22
Sample: JS-Cat7
Note : in  with MeOH
Inlet : Direct                         Ion Mode : FAB+
RT : 2.23 min                          Scan#: 24
Elements : C 21/0, H 22/0, N 3/0, S 1/0
Mass Tolerance      : 20ppm, 10mmu if m/z < 500, 50mmu if m/z > 2500
Unsaturation (U.S.) : 0.0 - 100.0

Observed m/z   Int%
  348.1530      8.1
Estimated m/z  Error[ppm]  U.S.    C     H     N     S
  348.1534       -1.3      13.5   21    22     3     1
```

1-(4-Nitrophenyl)-3-[(1*R*,2*R*)-2-(pentan-3-ylamino)-1,2-diphenylethyl]thiourea (1b)

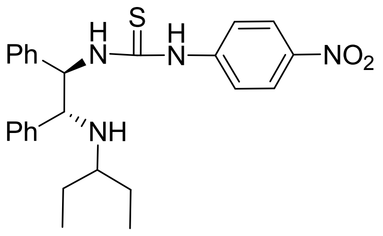

HRMS (FAB$^+$) for $C_{26}H_{31}N_4O_2S$ [M+H]$^+$ Calcd: 463.2168, Found: 463.2186.

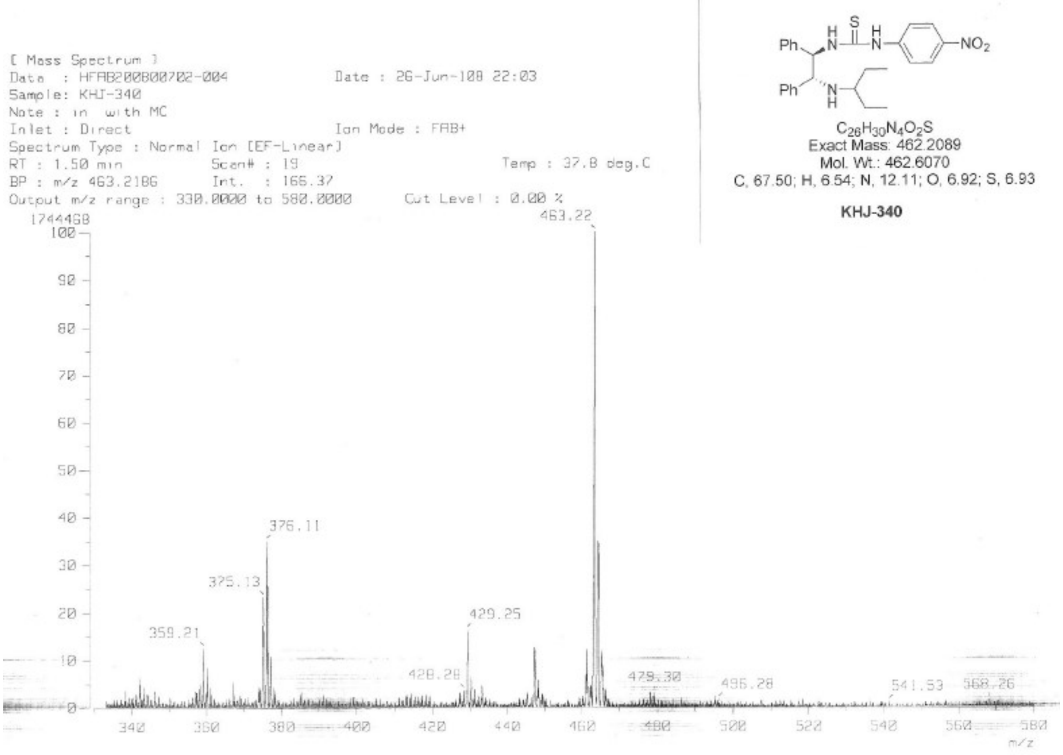

1-[(1*R*,2*R*)-2-Amino-1,2-diphenylethyl]-3-[3,5-Bis(trifluoromethyl)phenyl]thiourea (1b)

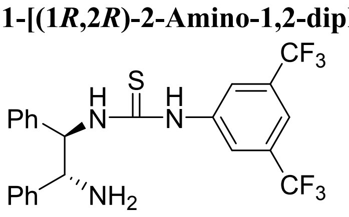

HRMS (FAB+) for $C_{23}H_{20}F_6N_3S$ [M+H]+ Calcd: 484.1282, Found: 484.1280

Instrument : MStation
Sample : 11
Note : m-NBA
Inlet : Direct Ion Mode : FAB+
RT : 0.16 min Scan# : (7,17)
Elements : C 100/0, H 100/0, F 10/0, N 5/2, S 2/0
Mass Tolerance : 1000ppm, 5mmu if m/z < 5, 10mmu if m/z > 10
Unsaturation (U.S.) : 5.0 - 20.0

	Observed m/z	Int%	Err[ppm / mmu]	U.S.	Composition
1	484.1280	12.32	-19.5 / -9.4	17.0	C27 H18 F6 N2
2			+19.5 / +9.4	14.0	C24 H16 F8 N2
3			+6.5 / +3.2	17.5	C26 H16 F6 N3
4			-6.1 / -3.0	10.0	C20 H17 F9 N4
5			-19.0 / -9.2	13.5	C22 H17 F7 N5
6			+19.9 / +9.6	10.5	C19 H15 F9 N5
7			+12.5 / +6.1	10.0	C21 H20 F8 N2 S
8			-0.4 / -0.2	13.5	C23 H20 F6 N3 S
9			-13.4 / -6.5	17.0	C25 H20 F4 N4 S
10			-13.1 / -6.3	6.0	C17 H21 F9 N4 S
11			+12.6 / +6.1	17.5	C24 H18 F4 N5 S
12			+12.9 / +6.3	6.5	C16 H19 F9 N5 S
13			+5.2 / +2.5	17.0	C26 H23 F3 N2 S2
14			+5.5 / +2.7	6.0	C18 H24 F8 N2 S2
15			-7.4 / -3.6	9.5	C20 H24 F6 N3 S2
16			-20.4 / -9.9	13.0	C22 H24 F4 N4 S2
17			+18.6 / +9.0	10.0	C19 H22 F6 N4 S2
18			+5.6 / +2.7	13.5	C21 H22 F4 N5 S2

1-(4-Fluorophenyl)-3-[(1*R*,2*R*)-2-(pentan-3-ylamino)-1,2-diphenylethyl]thiourea (1c)

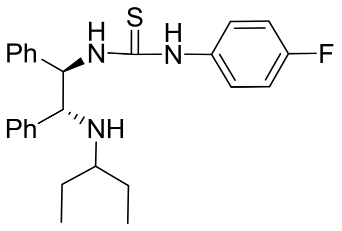

HRMS (FAB$^+$) for $C_{26}H_{31}FN_3S$[M+H]$^+$ Calcd: 436.2223, Found: 436.2201

[Elemental Composition]
Data : HFAB20080507-010 Date : 07-May-108 21:34
Sample: JS-Cat11
Note : in with MeOH
Inlet : Direct Ion Mode : FAB+
RT : 2.03 min Scan#: 22
Elements : C 26/0, H 31/0, N 3/0, F 1/0, S 1/0
Mass Tolerance : 20ppm, 50mmu if m/z > 2500
Unsaturation (U.S.) : 0.0 - 100.0

Observed m/z	Int%						
436.2201	30.9						
**Estimated m/z**	**Error[ppm]**	**U.S.**	**C**	**H**	**N**	**F**	**S**
436.2223	-5.0	13.5	26	31	3	1	1

1-[3,5-Bis(trifluoromethyl)phenyl]-3-[(1*R*,2*R*)-2-(pentan-3-ylamino)-1,2-diphenylethyl]thiourea (1d)

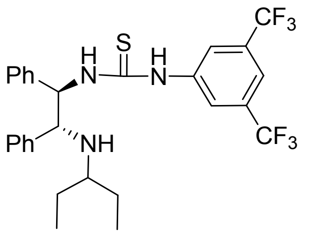

HRMS (FAB$^+$) for $C_{28}H_{30}F_6N_3S$ [M+H]$^+$ Calcd: 554.2065, Found: 554.2020

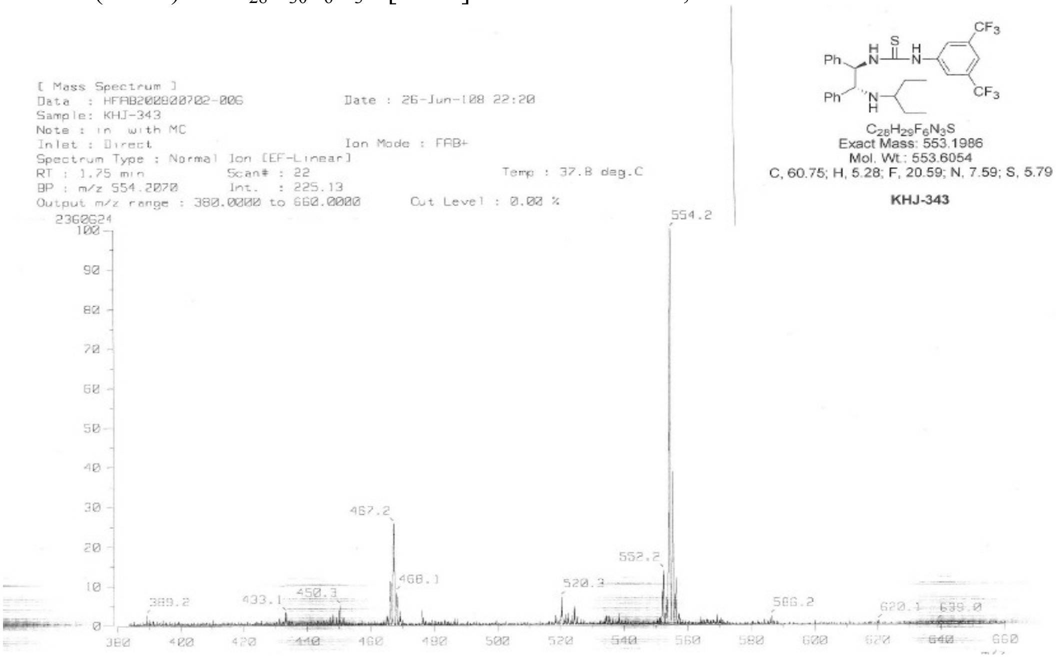

1-[(1*R*,2*R*)-2-(Pentan-3-ylamino)-1,2-diphenylethyl]-3-(*p*-tolyl)thiourea (1e)

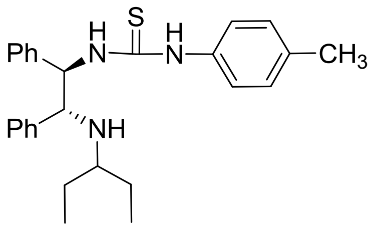

HRMS (FAB$^+$) for $C_{27}H_{34}N_3S$ [M+H]$^+$ Calcd: 432.2473, Found: 432.2460

[Elemental Composition] Page: 1
Data : HFAB20080507-009 Date : 07-May-108 21:27
Sample: JS-Cat10
Note : in with MeOH
Inlet : Direct Ion Mode : FAB+
RT : 1.36 min Scan#: 15
Elements : C 27/0, H 34/0, N 3/0, S 1/0
Mass Tolerance : 20ppm, 10mmu if m/z < 500, 50mmu if m/z > 2500
Unsaturation (U.S.) : 0.0 - 100.0

Observed m/z	Int%					
432.2460	64.5					
**Estimated m/z**	**Error[ppm]**	**U.S.**	**C**	**H**	**N**	**S**
432.2473	-3.1	13.5	27	34	3	1

1-(4-Cyanophenyl)-3-[(1*R*,2*R*)-2-(pentan-3-ylamino)-1,2-diphenylethyl]thiourea (1f)

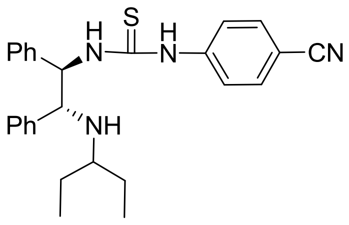

HRMS (FAB+) for $C_{27}H_{31}N_4S$ $[M+H]^+$ Calcd: 443.2269, Found: 443.2279.

```
Sample: KHJ-341
Note : in  with MC
Inlet : Direct                        Ion Mode : FAB+
Spectrum Type : Normal Ion [EF-Linear]
RT : 1.75 min          Scan# : 22                    Temp : 37.7 deg.C
BP : m/z 443.2279      Int.   : 282.10
Output m/z range : 330.0000 to 580.0000       Cut Level : 0.00 %
```

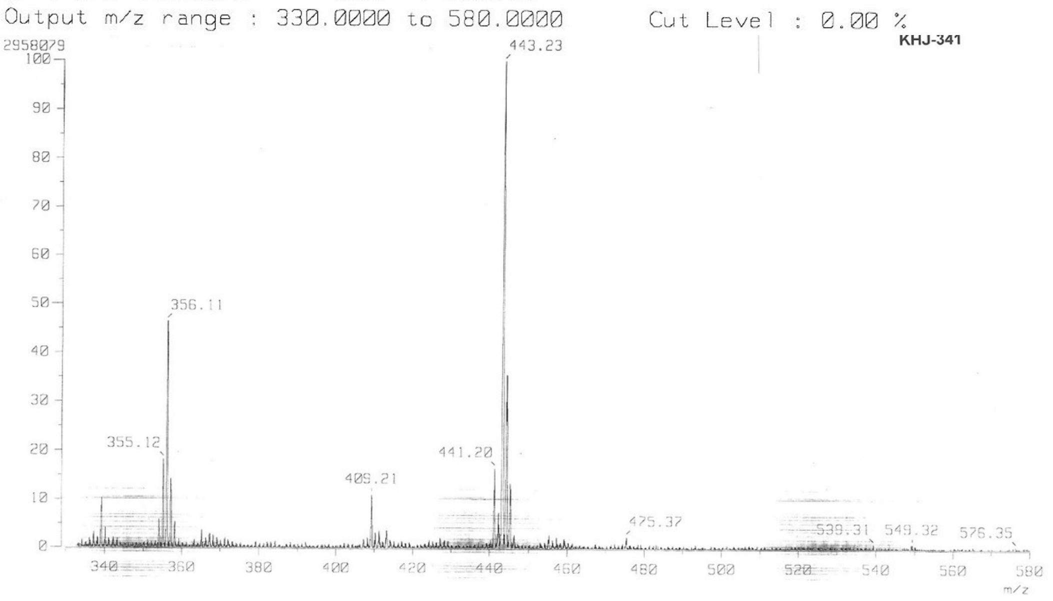

1-[(1*R*,2*R*)-2-(Pentan-3-ylamino)-1,2-diphenylethyl]-3-[4-(trifluoromethyl) phenyl]thiourea (1g)

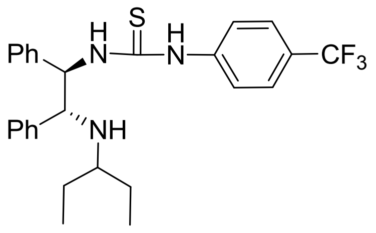

HRMS(FAB+) for $C_{27}H_{31}F_3N_3S[M+H]^+$ Calcd: 486.2191, Found: 486.2163.

```
[ Elemental Composition ]
Data   : HFAB2008000818-009            Date : 04-Aug-108 14:13
Sample: KHJ-375
Note : in  with MC
Inlet : Direct                         Ion Mode : FAB+
RT : 0.92 min                          Scan#: 12
Elements : C 27/0, H 31/0, N 3/0, F 3/0, S 1/0
Mass Tolerance      : 20ppm, 10mmu if m/z < 500, 50mmu if m/z > 2500
Unsaturation (U.S.) : 0.0 - 100.0

Observed m/z   Int%
  486.2163     100.0
Estimated m/z  Error[ppm]   U.S.     C     H     N     F     S
  486.2191       -5.8       13.5    27    31     3     3     1
```

1-[(1*R*,2*R*)-2-(Pentan-3-ylamino)-1,2-diphenylethyl]-3-(perfluorophenyl)thiourea (1h)

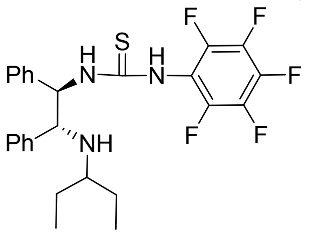

HRMS (FAB+) for $C_{26}H_{27}F_5N_3S$ $[M+H]^+$ Calcd: 508.1846, Found: 508.1823.

```
[ Elemental Composition ]
Data   : HFAB2008000818-010            Date : 04-Aug-108 14:20
Sample: KHJ-376
Note : in  with MC
Inlet : Direct                         Ion Mode : FAB+
RT : 1.25 min                          Scan#: 16
Elements : C 26/0, H 27/0, N 3/0, F 5/0, S 1/0
Mass Tolerance      : 20ppm, 10mmu if m/z < 500, 50mmu if m/z > 2500
Unsaturation (U.S.) : 0.0 - 100.0

Observed m/z   Int%
  508.1823     100.0
Estimated m/z  Error[ppm]   U.S.     C     H     N     F     S
  508.1846       -4.5       13.5    26    27     3     5     1
```

1-(2,6-Difluorophenyl)-3-[(1R,2R)-2-(pentan-3-ylamino)-1,2-diphenylethyl]thiourea (1i)

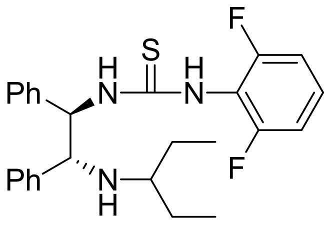

HRMS(FAB+) for C26H30F2N_3S$[M+H]^+$ Calcd: 454.2129, Found: 454.2133

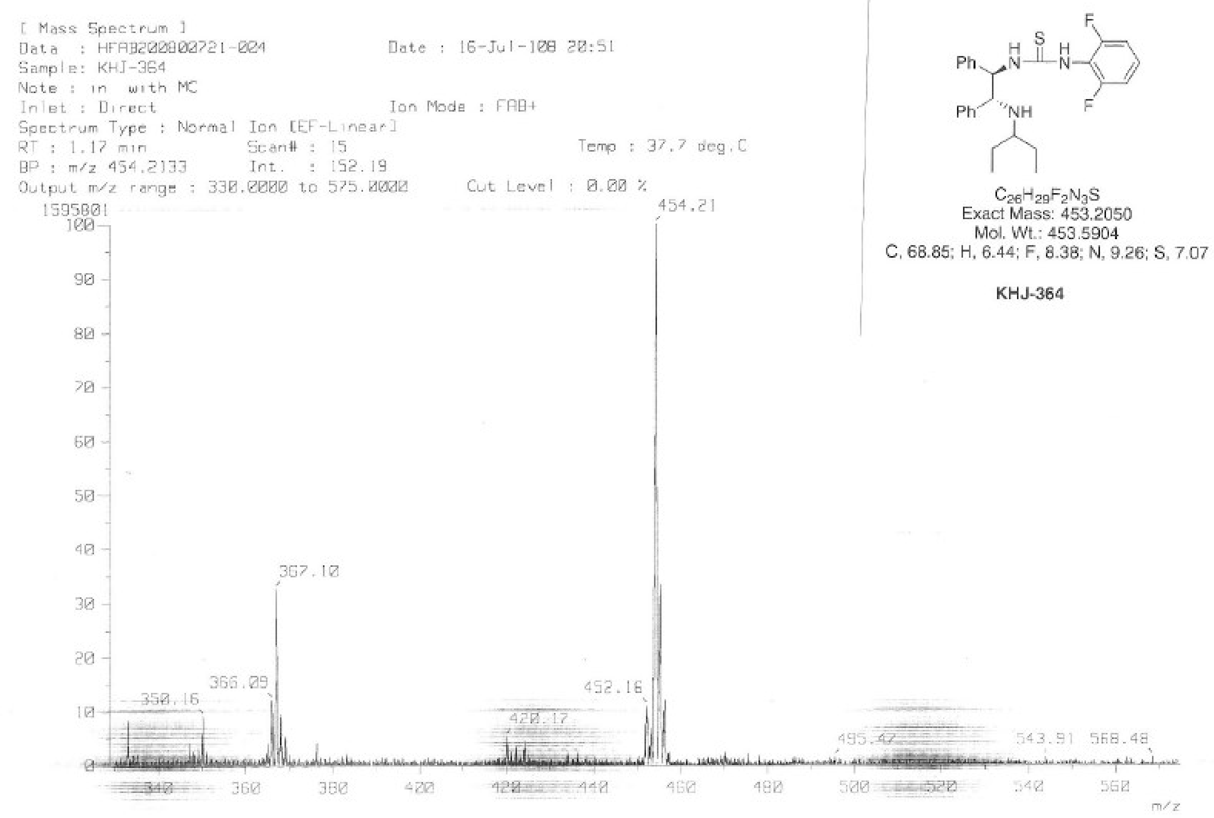

Ethyl-2-acetyl-4-nitro-3-phenylbutanoate(4a)

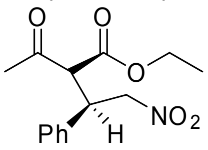

HRMS (FAB$^+$) Calcd. for $[C_{14}H_{18}NO_5]^+$: 280.1185; Found: 280.1187

Instrument : MStation
Sample : 1
Note : m-NBA
Inlet : Direct   Ion Mode : FAB+
RT : 0.85 min   Scan# : (25,113)
Elements : C 100/0, H 100/0, N 5/0, O 10/0
Mass Tolerance   : 1000ppm, 5mmu if m/z < 5, 10mmu if m/z > 10
Unsaturation (U.S.) : -0.5 - 10.0

	Observed m/z	Int%	Err[ppm / mmu]	U.S.	Composition
1	280.1187	60.05	+5.5 / +1.5	7.0	C12 H16 N4 O4
2			+0.7 / +0.2	6.5	C14 H18 N O5
3			-25.0 / -7.0	2.5	C8 H18 N5 O6
4			-29.8 / -8.4	2.0	C10 H20 N2 O7
5			+15.1 / +4.2	2.5	C9 H18 N3 O7
6			+10.3 / +2.9	2.0	C11 H20 O8

Ethyl-2-acetyl-2-methyl-4-nitro-3-phenylbutanoate(4b)

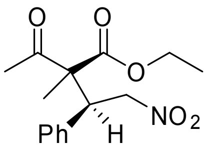

HRMS (FAB$^+$) Calcd. for $[C_{15}H_{20}NO_5]^+$: 294.1341

Instrument : MStation
Sample : 9
Note : m-NBA
Inlet : Direct   Ion Mode : FAB+
RT : 0.66 min   Scan# : (20,28)
Elements : C 100/0, H 100/0, N 5/0, O 10/0
Mass Tolerance   : 1000ppm, 5mmu if m/z < 5, 10mmu if m/z > 10
Unsaturation (U.S.) : 0.0 - 15.0

	Observed m/z	Int%	Err[ppm / mmu]	U.S.	Composition
1	294.1342	29.44	-22.6 / -6.7	15.0	C23 H18
2			-4.4 / -1.3	11.5	C16 H16 N5 O
3			-8.9 / -2.6	11.0	C18 H18 N2 O2
4			+33.8 / +9.9	11.5	C17 H16 N3 O2
5			+29.3 / +8.6	11.0	C19 H18 O3
6			+4.7 / +1.4	7.0	C13 H18 N4 O4
7			+0.2 / +0.1	6.5	C15 H20 N O5
8			-24.3 / -7.2	2.5	C9 H20 N5 O6
9			-28.9 / -8.5	2.0	C11 H22 N2 O7
10			+13.9 / +4.1	2.5	C10 H20 N3 O7
11			+9.3 / +2.7	2.0	C12 H22 O8

Ethyl-1-(2-nitro-1-phenylethyl)-2-oxocyclopentanecarboxylate(4c)

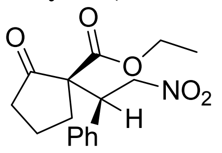

HRMS(FAB$^+$) Calcd. for $C_{16}H_{20}NO_5$ Na:306.1341, found: 306.1341

Instrument : MStation
Sample : 2
Note : m-NBA
Inlet : Direct   Ion Mode : FAB+
RT : 0.14 min   Scan# : (5,11)
Elements : C 100/0, H 100/0, N 5/0, O 10/0
Mass Tolerance   : 1000ppm, 5mmu if m/z < 5, 10mmu if m/z > 10
Unsaturation (U.S.) : 0.0 - 15.0

	Observed m/z	Int%	Err[ppm / mmu]	U.S.	Composition
1	306.1341	21.87	-4.5 / -1.4	12.5	C17 H16 N5 O
2			-8.9 / -2.7	12.0	C19 H18 N2 O2
3			+32.2 / +9.8	12.5	C18 H16 N3 O2
4			+27.8 / +8.5	12.0	C20 H18 O3
5			+4.2 / +1.3	8.0	C14 H18 N4 O4
6			-0.2 / -0.0	7.5	C16 H20 N O5
7			-23.7 / -7.3	3.5	C10 H20 N5 O6
8			-28.1 / -8.6	3.0	C12 H22 N2 O7
9			+13.0 / +4.0	3.5	C11 H20 N3 O7
10			+8.6 / +2.6	3.0	C13 H22 O8

Methyl-1-(2-nitro-1-phenylethyl)-2-oxocyclopentanecarboxylate (4d)

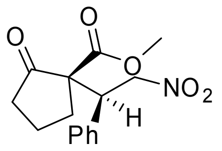

HRMS(FAB$^+$) Calcd. for $C_{15}H_{19}NO_5$: 292.1185, found: 292.1180

Instrument : MStation
Sample : 8
Note : m-NBA
Inlet : Direct   Ion Mode : FAB+
RT : 2.79 min   Scan# : (82,97)
Elements : C 100/0, H 100/0, N 5/0, O 10/0
Mass Tolerance   : 1000ppm, 5mmu if m/z < 5, 10mmu if m/z > 10
Unsaturation (U.S.) : 0.0 - 15.0

	Observed m/z	Int%	Err[ppm / mmu]	U.S.	Composition
1	292.1180	3.08	-6.3 / -1.8	12.5	C16 H14 N5 O
2			-10.9 / -3.2	12.0	C18 H16 N2 O2
3			+32.2 / +9.4	12.5	C17 H14 N3 O2
4			+27.6 / +8.1	12.0	C19 H16 O3
5			+2.9 / +0.8	8.0	C13 H16 N4 O4
6			-1.7 / -0.5	7.5	C15 H18 N O5
7			-26.4 / -7.7	3.5	C9 H18 N5 O6
8			-31.0 / -9.1	3.0	C11 H20 N2 O7
9			+12.1 / +3.5	3.5	C10 H18 N3 O7
10			+7.5 / +2.2	3.0	C12 H20 O8

Methyl-1-(2-nitro-1-phenylethyl)-2-oxocyclohexanecarboxylate(4e)

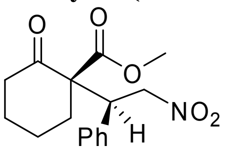

HRMS(FAB$^+$) Calcd. for $C_{16}H_{20}NO_5$: 306.1341, found: 306.1340

Instrument : MStation
Sample : 4
Note : m-NBA
Inlet : Direct   Ion Mode : FAB+
RT : 2.62 min   Scan# : (77,79)
Elements : C 100/0, H 100/0, N 5/0, O 10/0
Mass Tolerance   : 1000ppm, 5mmu if m/z < 5, 10mmu if m/z > 10
Unsaturation (U.S.) : -0.5 - 10.0

	Observed m/z	Int%	Err[ppm / mmu]	U.S.	Composition
1	306.1340	38.33	+3.9 / +1.2	8.0	C14 H18 N4 O4
2			-0.5 / -0.1	7.5	C16 H20 N O5
3			-24.0 / -7.4	3.5	C10 H20 N5 O6
4			-28.4 / -8.7	3.0	C12 H22 N2 O7
5			+12.7 / +3.9	3.5	C11 H20 N3 O7
6			+8.3 / +2.5	3.0	C13 H22 O8
7			+25.8 / +7.9	-0.5	C6 H20 N5 O9

Methyl-1,2,3,4-tetrahydro-2-(2-nitro-1-phenylethyl)-1-oxonapht-halene-2-carboxylate(4h)

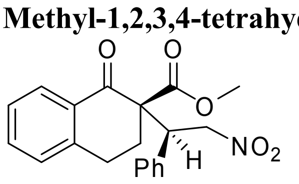

HRMS(FAB$^+$) Calcd. for $C_{20}H_{20}NO_5$: 354.1341, found: 354.1345

Instrument : MStation
Sample : 3
Note : m-NBA
Inlet : Direct   Ion Mode : FAB+
RT : 0.34 min   Scan# : (9,35)
Elements : C 100/0, H 100/0, N 5/0, O 10/0
Mass Tolerance   : 1000ppm, 5mmu if m/z < 5, 10mmu if m/z > 10
Unsaturation (U.S.) : 5.0 - 20.0

	Observed m/z	Int%	Err[ppm / mmu]	U.S.	Composition
1	354.1345	21.38	-17.9 / -6.4	20.0	C28 H18
2			-2.8 / -1.0	16.5	C21 H16 N5 O
3			-6.6 / -2.3	16.0	C23 H18 N2 O2
4			+25.1 / +8.9	16.0	C24 H18 O3
5			+4.8 / +1.7	12.0	C18 H18 N4 O4
6			+1.0 / +0.4	11.5	C20 H20 N O5
7			-19.4 / -6.9	7.5	C14 H20 N5 O6
8			-23.2 / -8.2	7.0	C16 H22 N2 O7
9			+12.4 / +4.4	7.5	C15 H20 N3 O7
10			+8.6 / +3.0	7.0	C17 H22 O8

Methyl-1-(2-nitro-1-p-tolylethyl)-2-oxocyclopentanecarboxylate(4i)

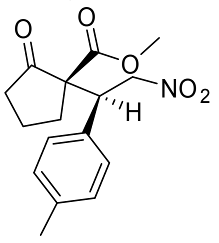

HRMS(FAB$^+$) Calcd. for $[C_{16}H_{20}NO_5]^+$: 306.1341, found: 306.1340

Instrument : MStation
Sample : 4
Note : m-NBA
Inlet : Direct   Ion Mode : FAB+
RT : 2.62 min   Scan# : (77,79)
Elements : C 100/0, H 100/0, N 5/0, O 10/0
Mass Tolerance   : 1000ppm, 5mmu if m/z < 5, 10mmu if m/z > 10
Unsaturation (U.S.) : -0.5 - 10.0

	Observed m/z	Int%	Err[ppm / mmu]	U.S.	Composition
1	306.1340	38.33	+3.9 / +1.2	8.0	C14 H18 N4 O4
2			-0.5 / -0.1	7.5	C16 H20 N O5
3			-24.0 / -7.4	3.5	C10 H20 N5 O6
4			-28.4 / -8.7	3.0	C12 H22 N2 O7
5			+12.7 / +3.9	3.5	C11 H20 N3 O7
6			+8.3 / +2.5	3.0	C13 H22 O8
7			+25.8 / +7.9	-0.5	C6 H20 N5 O9

Methyl-1-{1-(4-chlorophenyl)-2-nitro}ethyl-2-oxocyclopentaneca-rboxylate(4j)

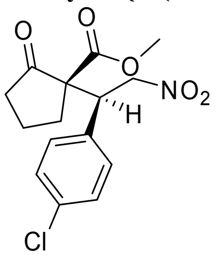

HRMS(FAB$^+$) Calcd. For $[C_{15}H_{16}ClNO_5]^+$: 325.0717 , found: 325.0720

Instrument : MStation
Sample : 6
Note : m-NBA
Inlet : Direct Ion Mode : FAB+
RT : 2.20 min Scan# : (65,68)
Elements : C 100/0, H 100/0, 35Cl 2/0, 37Cl 2/0, N 5/0, O 10/0
Mass Tolerance : 1000ppm, 5mmu if m/z < 5, 10mmu if m/z > 10
Unsaturation (U.S.) : 0.0 - 15.0

	Observed m/z	Int%	Err[ppm / mmu]	U.S.	Composition
1	326.0796	13.19	-12.6 / -4.1	3.5	C15 H23 35Cl 37Cl2 N
2			+26.0 / +8.5	4.0	C14 H21 35Cl 37Cl2 N2
3			+7.5 / +2.4	3.5	C13 H21 35Cl2 37Cl N3
4			-7.0 / -2.3	8.5	C16 H18 37Cl2 N3
5			-25.5 / -8.3	8.0	C15 H18 35Cl 37Cl N4
6			+13.1 / +4.3	8.5	C14 H16 35Cl 37Cl N5
7			+3.4 / +1.1	3.0	C15 H23 35Cl2 37Cl O
8			-11.1 / -3.6	8.0	C18 H20 37Cl2 O
9			-29.6 / -9.6	7.5	C17 H20 35Cl 37Cl N O
10			+27.5 / +9.0	8.5	C17 H18 37Cl2 N O
11			+9.0 / +2.9	8.0	C16 H18 35Cl 37Cl N2 O
12			-9.5 / -3.1	7.5	C15 H18 35Cl2 N3 O
13			-24.0 / -7.8	12.5	C18 H15 37Cl N3 O
14			+29.1 / +9.5	8.0	C14 H16 35Cl2 N4 O
15			+14.6 / +4.8	13.0	C17 H13 37Cl N4 O
16			-3.9 / -1.3	12.5	C16 H13 35Cl N5 O
17			-13.6 / -4.4	7.0	C17 H20 35Cl2 O2
18			-28.1 / -9.2	12.0	C20 H17 37Cl O2
19			+25.0 / +8.1	7.5	C16 H18 35Cl2 N O2
20			+10.5 / +3.4	12.5	C19 H15 37Cl N O2
21			-8.0 / -2.6	12.0	C18 H15 35Cl N2 O2
22			+30.6 / +10.0	12.5	C17 H13 35Cl N3 O2
23			+5.4 / +1.7	4.5	C11 H18 37Cl2 N5 O2
24			+26.5 / +8.6	12.0	C19 H15 35Cl O3
25			+1.2 / +0.4	4.0	C13 H20 37Cl2 N2 O3
26			-17.2 / -5.6	3.5	C12 H20 35Cl 37Cl N3 O3
27			+21.3 / +7.0	4.0	C11 H18 35Cl 37Cl N4 O3
28			+2.9 / +0.9	3.5	C10 H18 35Cl2 N5 O3
29			-11.6 / -3.8	8.5	C13 H15 37Cl N5 O3
30			-21.4 / -7.0	3.0	C14 H22 35Cl 37Cl O4
31			+17.2 / +5.6	3.5	C13 H20 35Cl 37Cl N O4
32			-1.3 / -0.4	3.0	C12 H20 35Cl2 N2 O4
33			-15.7 / -5.1	8.0	C15 H17 37Cl N2 O4
34			+22.8 / +7.4	8.5	C14 H15 37Cl N3 O4
35			+4.3 / +1.4	8.0	C13 H15 35Cl N4 O4
36			-28.6 / -9.3	12.5	C15 H12 N5 O4
37			+18.7 / +6.1	8.0	C16 H17 37Cl O5
38			+0.2 / +0.1	7.5	C15 H17 35Cl N O5

Methyl-1-{1-(4-bromophenyl)-2-nitroethyl}-2-oxocyclopentaneca-rboxylate(4k)

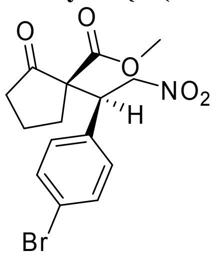

HRMS(FAB+) Calcd. for $[C_{15}H_{17}BrNO_5]^+$:370.0290, found: 370.0288

Instrument : MStation
Sample : 5
Note : m-NBA
Inlet : Direct Ion Mode : FAB+
RT : 2.62 min Scan# : (88,93)
Elements : C 100/0, H 100/0, 79Br 2/0, 81Br 2/0, N 3/0, O 10/0
Mass Tolerance : 1000ppm, 5mmu if m/z < 5, 10mmu if m/z > 10
Unsaturation (U.S.) : 0.0 - 15.0

	Observed m/z	Int%	Err[ppm / mmu]	U.S.	Composition
1	370.0288	3.42	+6.1 / +2.3	2.0	C13 H24 81Br2 N2
2			-7.7 / -2.8	1.5	C12 H24 79Br 81Br N3
3			-11.3 / -4.2	1.0	C14 H26 79Br 81Br O
4			-25.2 / -9.3	0.5	C13 H26 79Br2 N O
5			+22.7 / +8.4	1.5	C13 H24 79Br 81Br N O
6			+8.8 / +3.3	1.0	C12 H24 79Br2 N2 O
7			-24.3 / -9.0	12.5	C18 H15 81Br N3 O
8			+6.0 / +2.2	12.5	C19 H15 81Br N O2
9			-7.8 / -2.9	12.0	C18 H15 79Br N2 O2
10			+26.2 / +9.7	12.5	C17 H13 79Br N3 O2
11			+22.6 / +8.3	12.0	C19 H15 79Br O3
12			-17.1 / -6.3	8.0	C15 H17 81Br N2 O4
13			+16.9 / +6.3	8.5	C14 H15 81Br N3 O4
14			+13.3 / +4.9	8.0	C16 H17 81Br O5
15			-0.6 / -0.2	7.5	C15 H17 79Br N O5
16			-9.8 / -3.6	3.5	C12 H19 81Br N O7
17			-23.7 / -8.8	3.0	C11 H19 79Br N2 O7
18			+24.1 / +8.9	4.0	C11 H17 81Br N2 O7
19			+10.3 / +3.8	3.5	C10 H17 79Br N3 O7
20			+6.7 / +2.5	3.0	C12 H19 79Br O8
21			-6.3 / -2.3	14.5	C16 H8 N3 O8
22			-10.0 / -3.7	14.0	C18 H10 O9
23			+24.0 / +8.9	14.5	C17 H8 N O9

Methyl-1-(1-(4-methoxyphenyl)-2-nitroethyl)-2-oxocyclopentane-carboxylate(4l)

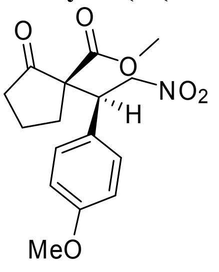

HRMS(FAB+) Calcd. for $[C_{16}H_{19}NO_6]^+$: 321.1212, found: 321.1210

Instrument : MStation
Sample : 7
Note : m-NBA
Inlet : Direct Ion Mode : FAB+
RT : 0.21 min Scan# : (7,43)
Elements : C 100/0, H 100/0, N 5/0, O 10/0
Mass Tolerance : 1000ppm, 5mmu if m/z < 5, 10mmu if m/z > 10
Unsaturation (U.S.) : 0.0 - 15.0

	Observed m/z	Int%	Err[ppm / mmu]	U.S.	Composition
1	321.1210	81.74	-4.9 / -1.6	13.0	C17 H15 N5 O2
2			-9.1 / -2.9	12.5	C19 H17 N2 O3
3			+30.1 / +9.7	13.0	C18 H15 N3 O3
4			+25.9 / +8.3	12.5	C20 H17 O4
5			+3.4 / +1.1	8.5	C14 H17 N4 O5
6			-0.7 / -0.2	8.0	C16 H19 N O6
7			-23.2 / -7.4	4.0	C10 H19 N5 O7
8			-27.4 / -8.8	3.5	C12 H21 N2 O8
9			+11.8 / +3.8	4.0	C11 H19 N3 O8
10			+7.6 / +2.4	3.5	C13 H21 O9
11			+24.3 / +7.8	0.0	C6 H19 N5 O10

Methyl-1-(1-(2-methoxyphenyl)-2-nitroethyl)-2-oxocyclopentane-carboxylate(4m)

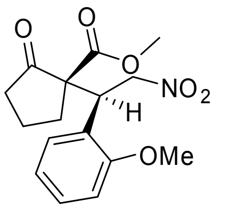

LRMS(ESI) Calcd. for $[C_{16}H_{19}NO_6Na]^+$:344.1110, found: 344.1137

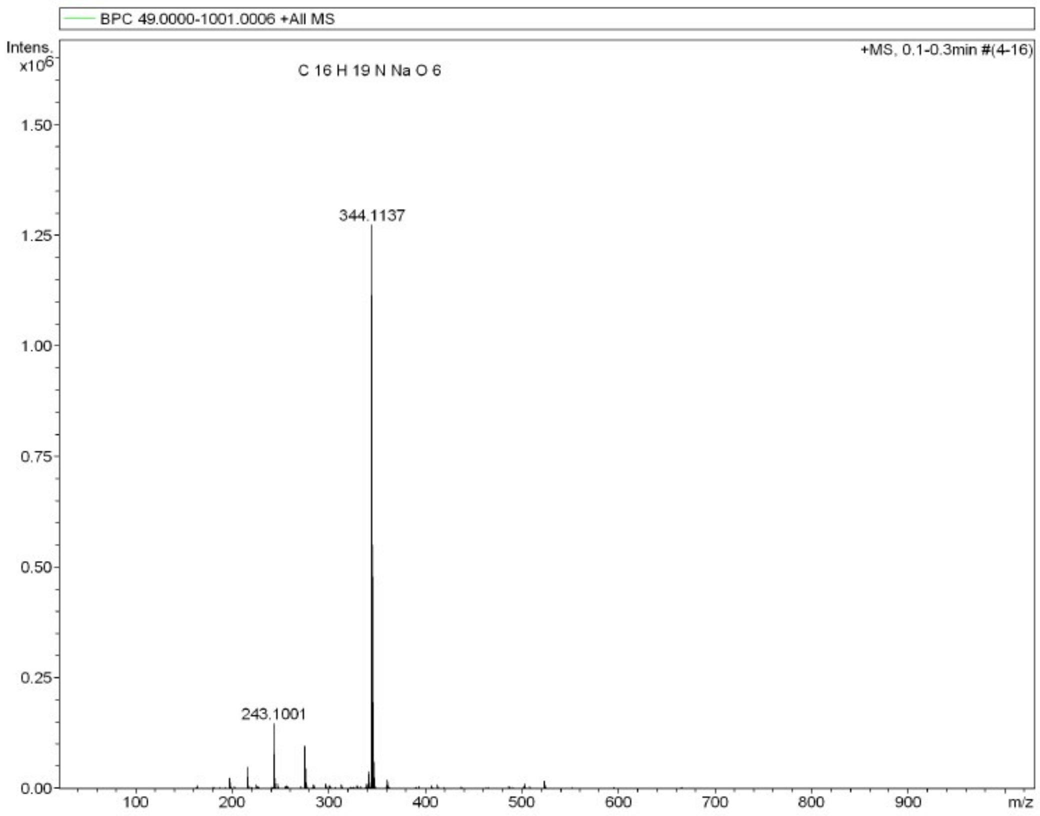

Methyl-1-(1-nitrohexan-2-yl)-2-oxocyclopentanecarboxylate(4n)

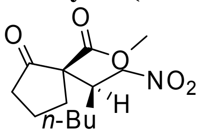

LRMS(ESI) Calcd. for $[C_{13}H_{21}NO_5Na]^+$:294.1317, found: 294.1352

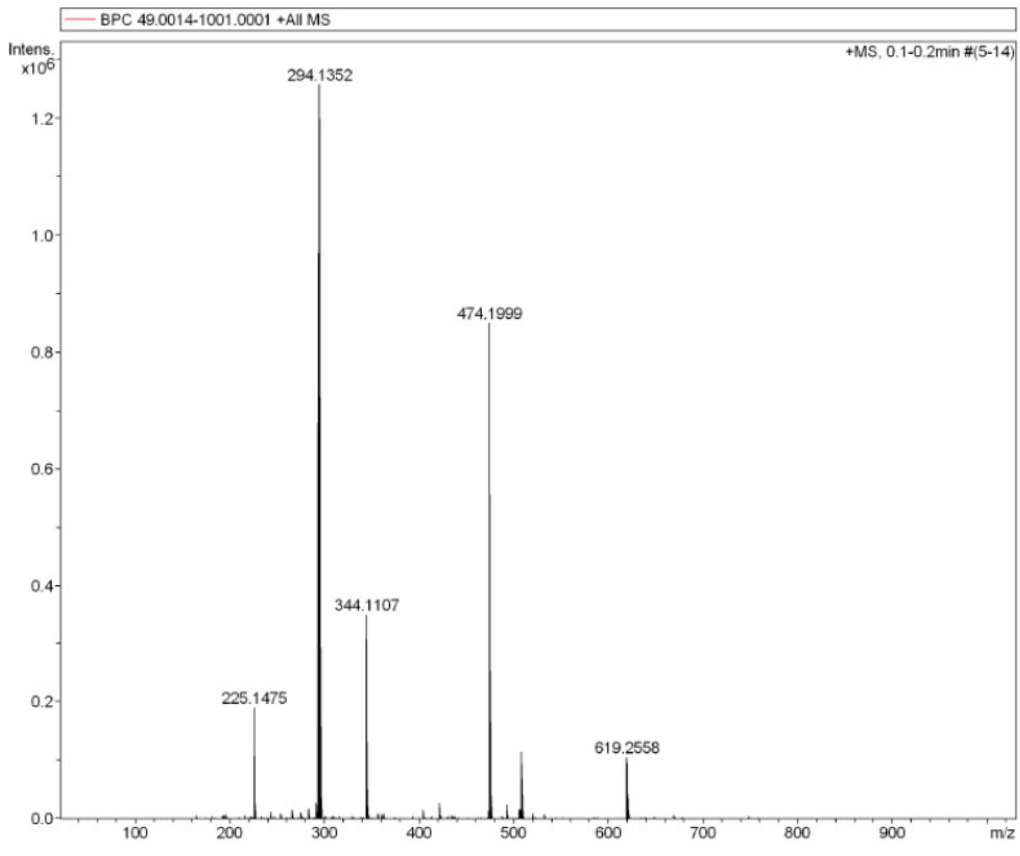

Methyl-1-(2-nitro-1-p-tolylethyl)-2-oxocyclohexanecarboxylate(4q)

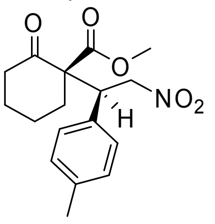

LRMS(ESI) Calcd. for $[C_{17}H_{21}NO_5Na]^+$: 342.1317, found: 342.1297

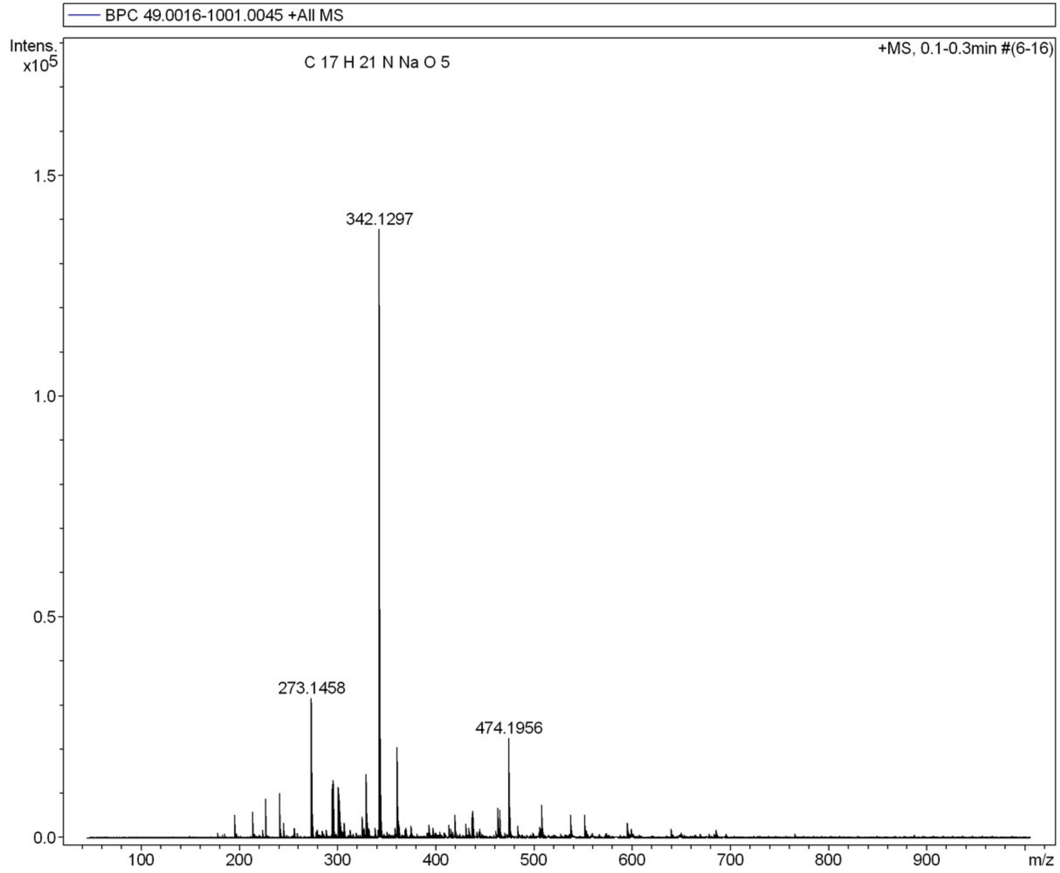

Methyl-1-(1-(4-chlorophenyl)-2-nitroethyl)-2-oxocyclohexanecar-boxylate(4r)

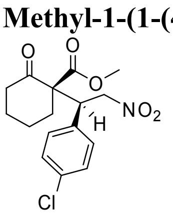

LRMS(ESI) Calcd for $[C_{16}H_{18}ClNO_5Na]^+$: 362.0771, found: 362.0763

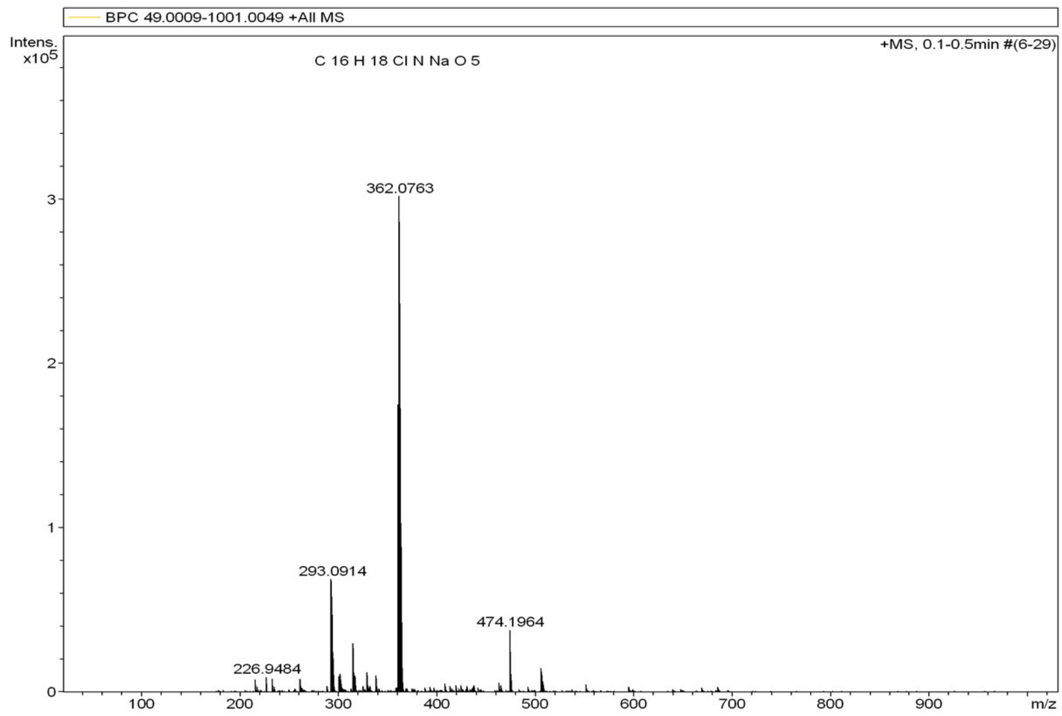

Methyl-1-(1-(4-bromophenyl)-2-nitroethyl)-2-oxocyclohexanecar-boxylate(4s)

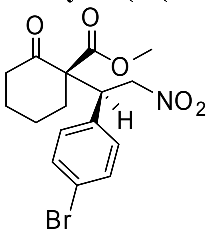

LRMS(ESI) Calcd. for $[C_{16}H_{18}BrNO_5Na]^+$: 406.0266, found: 408.0255

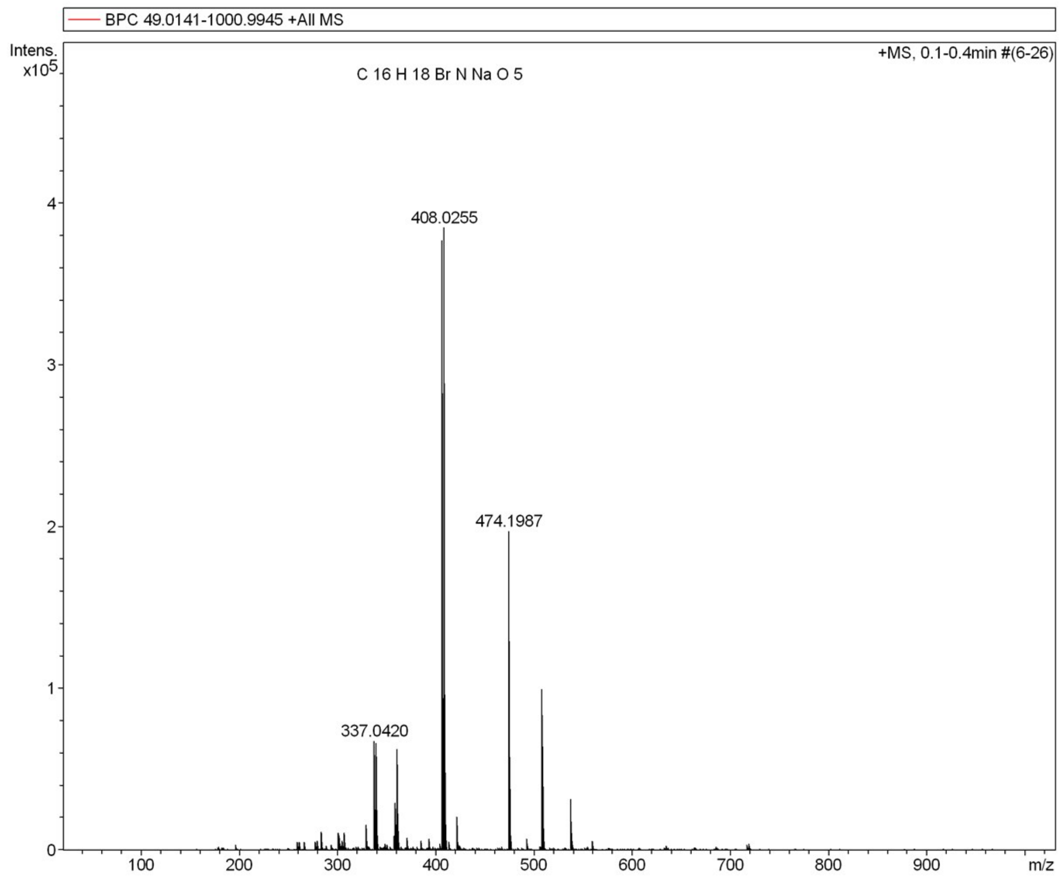

Methyl-1-(1-(4-methoxyphenyl)-2-nitroethyl)-2-oxocyclohexane-carboxylate(4t)

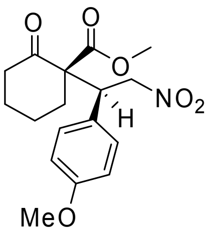

LRMS(ESI) Calcd. for $[C_{17}H_{21}NO_6Na]^+$: 358.1267, found: 358.1252

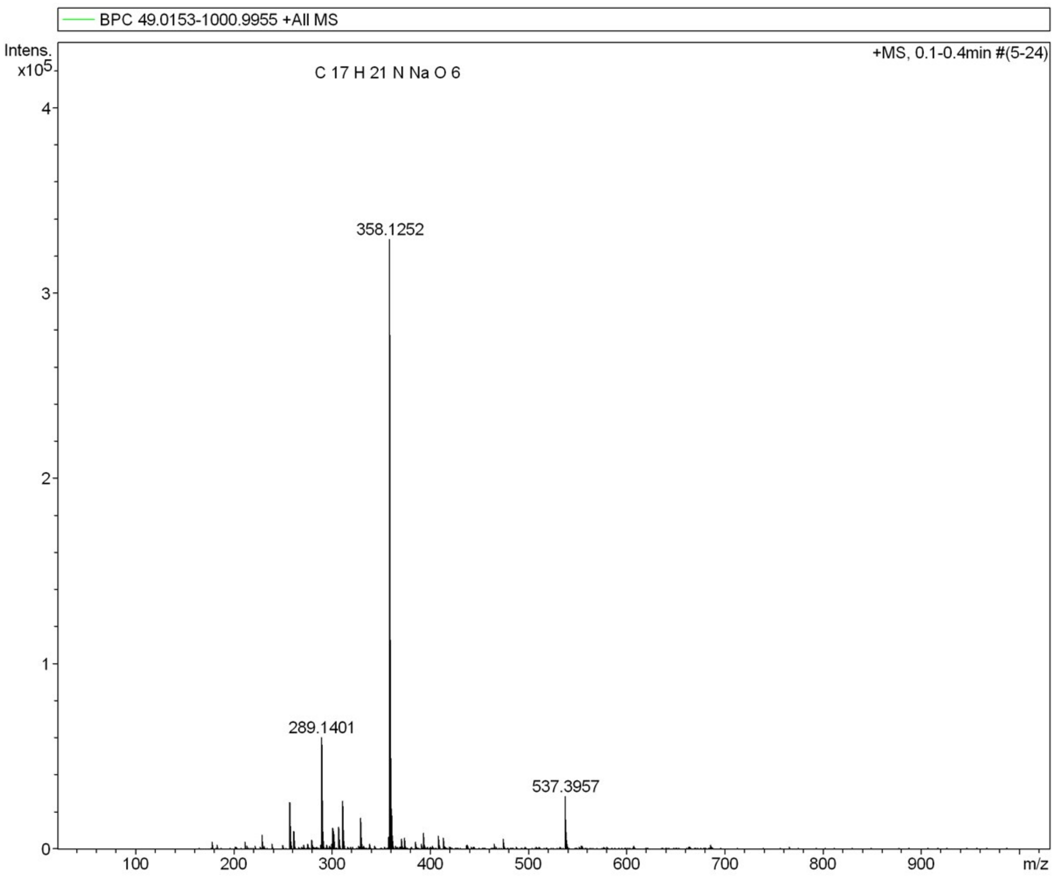

Dimethyl 2-(2-nitro-1-phenylethyl)malonate (5a)

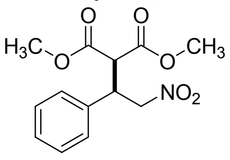

HRMS(ESI) for $C_{13}H_{16}N_1O_6[M+H]^+$ Calcd: 282.09721, Found: 282.09726

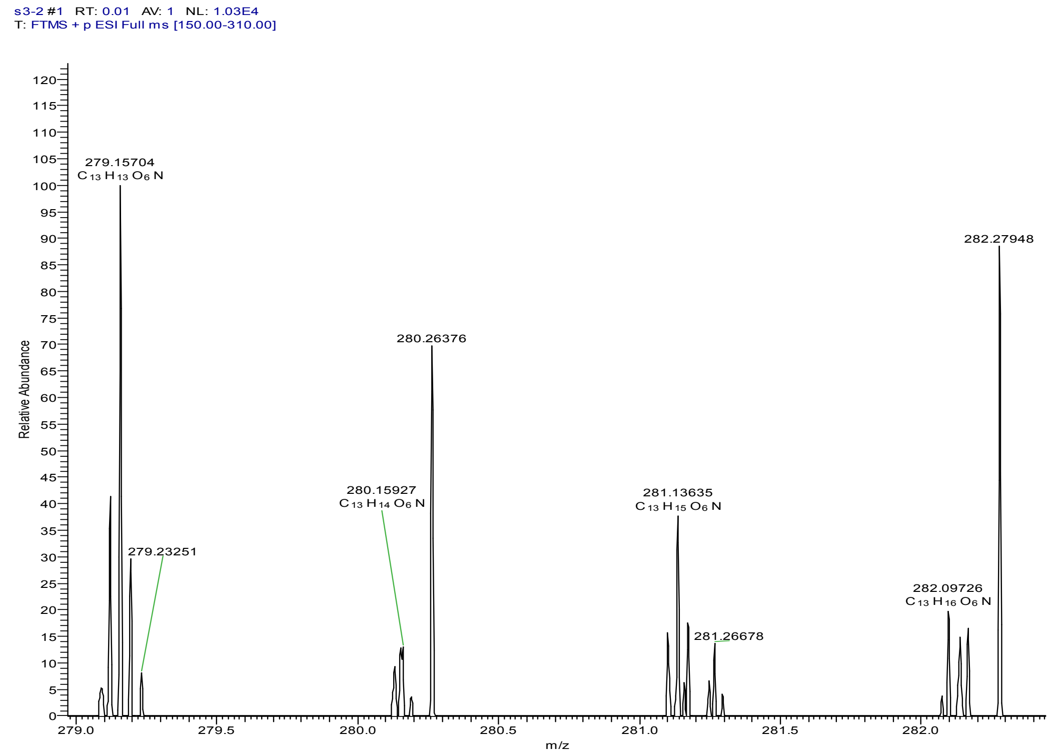

Diethyl 2-(2-nitro-1-phenylethyl)malonate (5b)

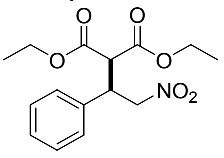

HRMS(ESI) for $C_{15}H_{20}N_1O_6[M+H]^+$ Calcd: 310.12851, Found: 310.12936

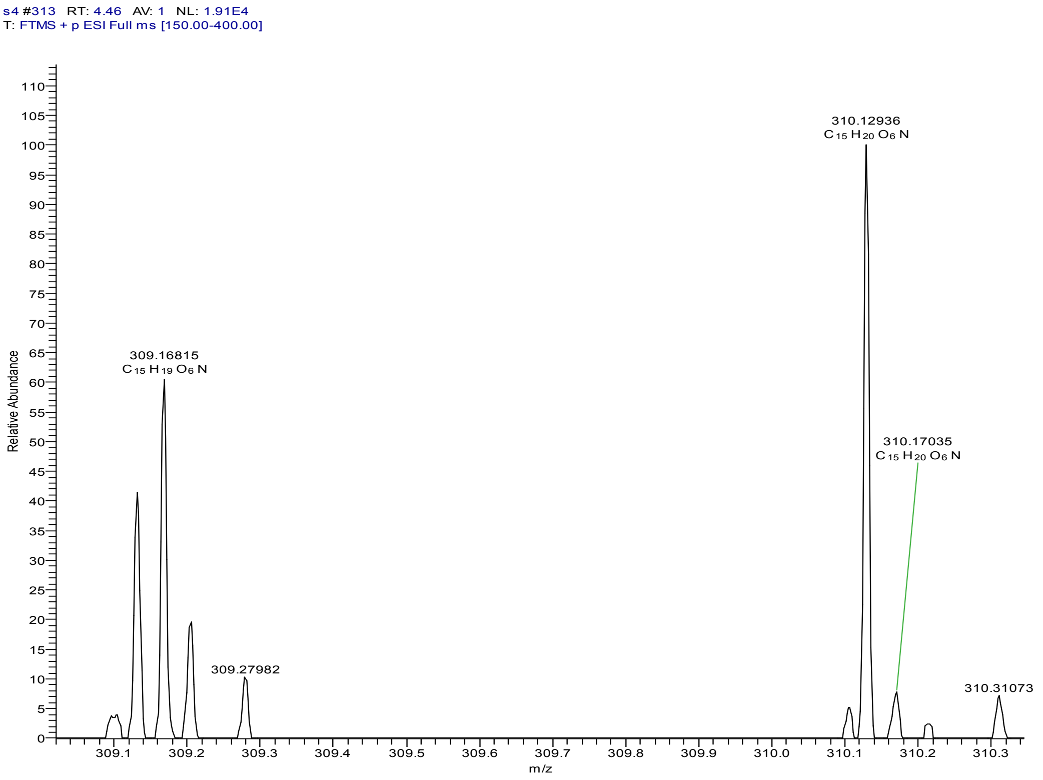

Diisopropyl 2-(2-nitro-1-phenylethyl)malonate (5c)

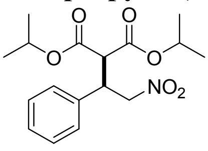

HRMS(ESI) for $C_{17}H_{24}N_1O_6[M+H]^+$ Calcd: 338.15981 Found: 338.16336

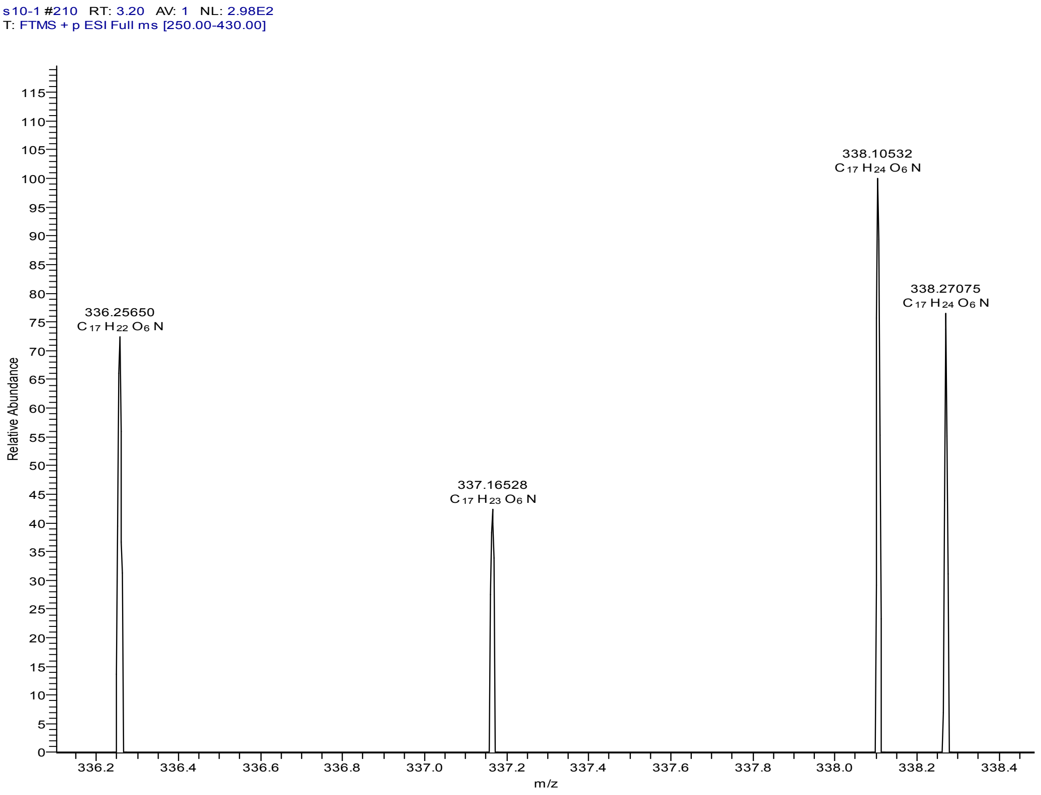

Dipropyl 2-(2-nitro-1-phenylethyl)malonate (5d)

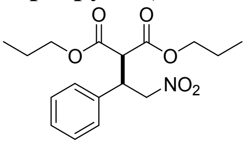

HRMS(ESI) for $C_{17}H_{24}N_1O_6[M+H]^+$ Calcd: 338.15981 Found: 338.16336

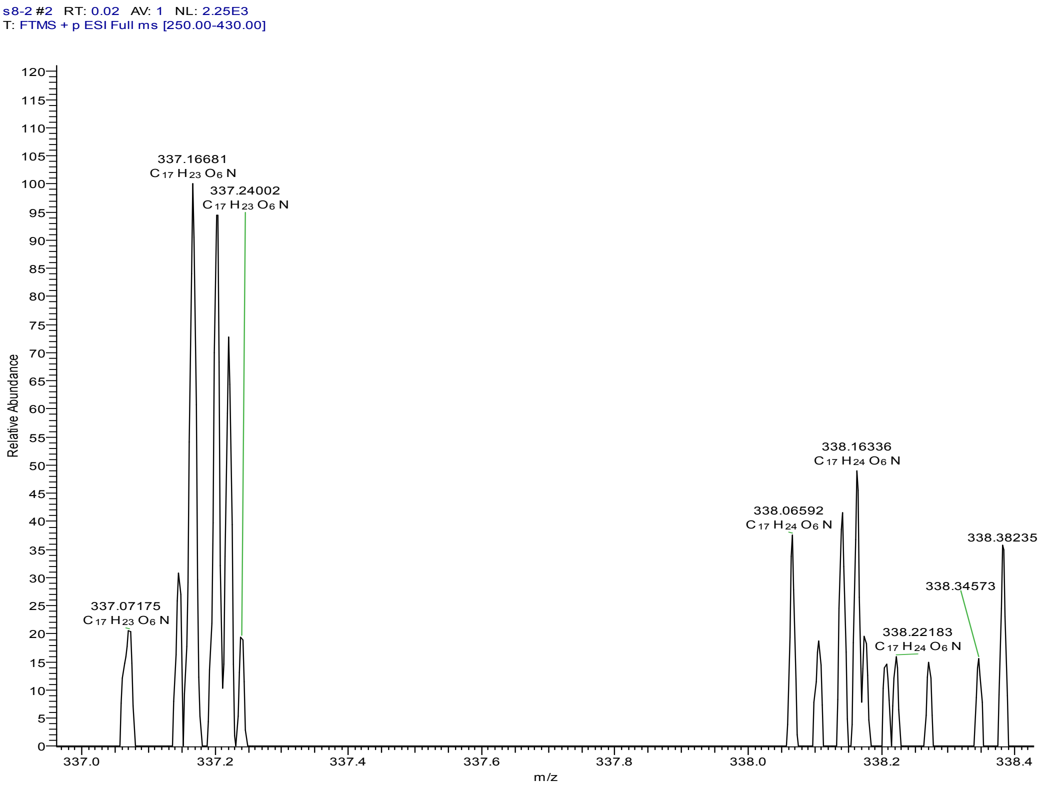

Dibutyl 2-(2-nitro-1-phenylethyl)malonate (5e)

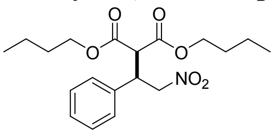

HRMS(EI) for $C_{19}H_{27}NO_6[M]^+$ Calcd: 365.1838 Found: 365.1830

```
Sample: CSY-(dibutyl-mal)
Note : -
Inlet : Direct                     Ion Mode : EI+
RT : 0.70 min                      Scan#: 22
Elements : C 19/0, H 27/0, N 1/0, O 6/0
Mass Tolerance      : 1000ppm, 10mmu if m/z < 10, 20mmu if m/z > 20
Unsaturation (U.S.) : -0.5 - 50.0

Observed m/z Int%   Err[ppm / mmu]    U.S.  Composition
  365.1830    0.2     -2.2 /  -0.8     7.0  C 19 H 27 N O 6
```

Diethyl 2-[1-(4-bromophenyl)-2-nitroethyl]malonate (5f)

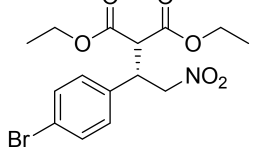

HRMS(ESI) for $C_{15}H_{19}N_1O_6Br[M+H]^+$ Calcd: 388.03903 Found: 388.04495

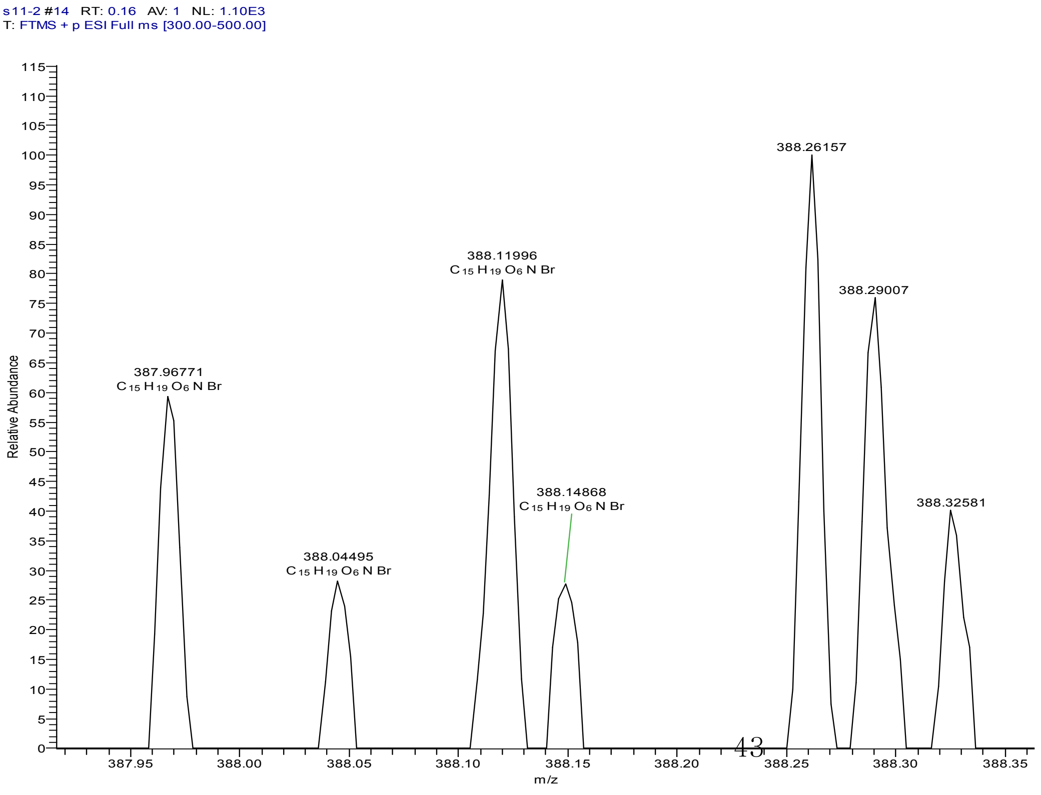

Diethyl 2-(1-(4-chlorophenyl)-2-nitroethyl)malonate (5g)

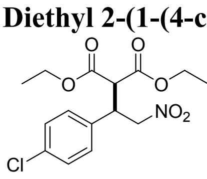

HRMS(ESI) for $C_{15}H_{19}N_1O_6Cl[M+H]^+$ Calcd: 344.08954 Found: 344.09119

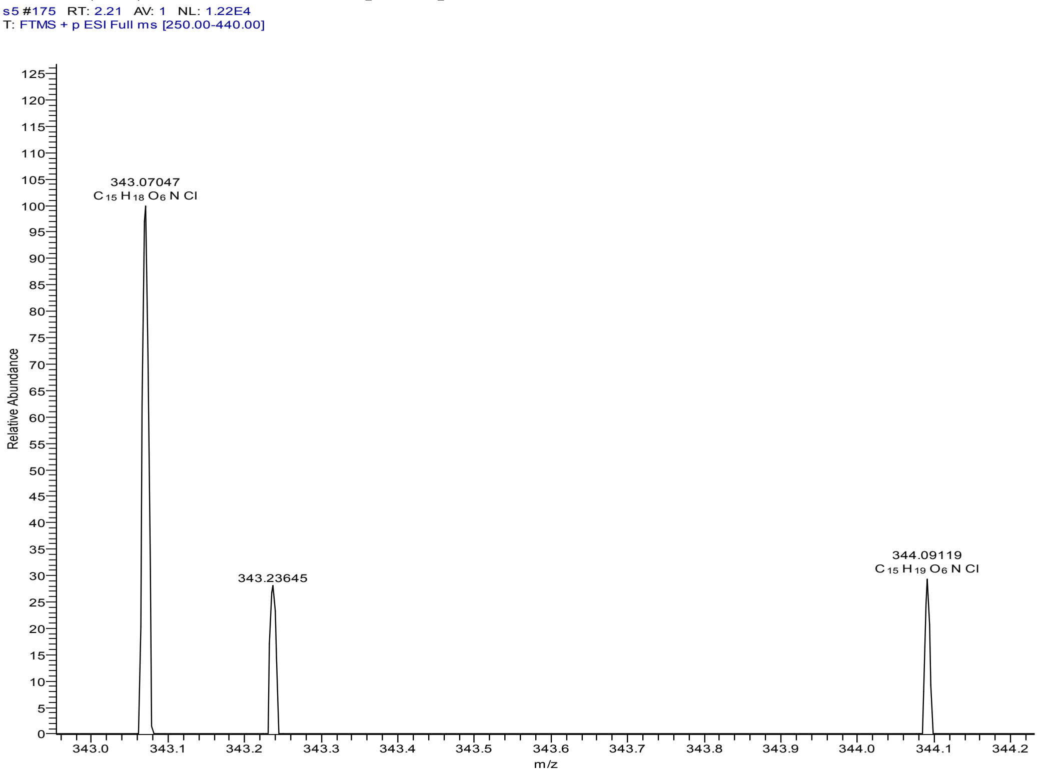

Diethyl 2-[2-nitro-1-(*p*-tolyl)ethyl]malonate (5h)

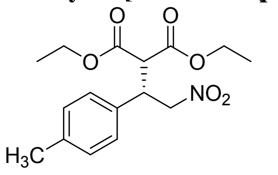

HRMS(ESI) for $C_{16}H_{22}N_1O_6[M+H]^+$ Calcd: 324.14416 Found: 324.14648

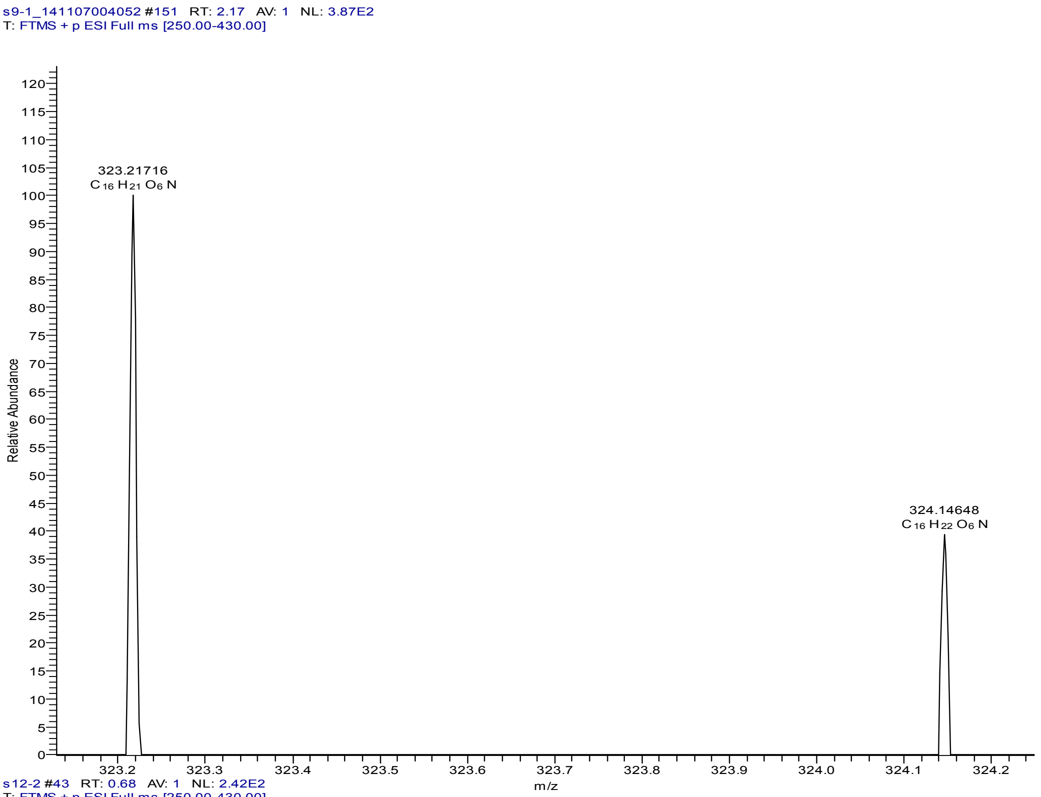

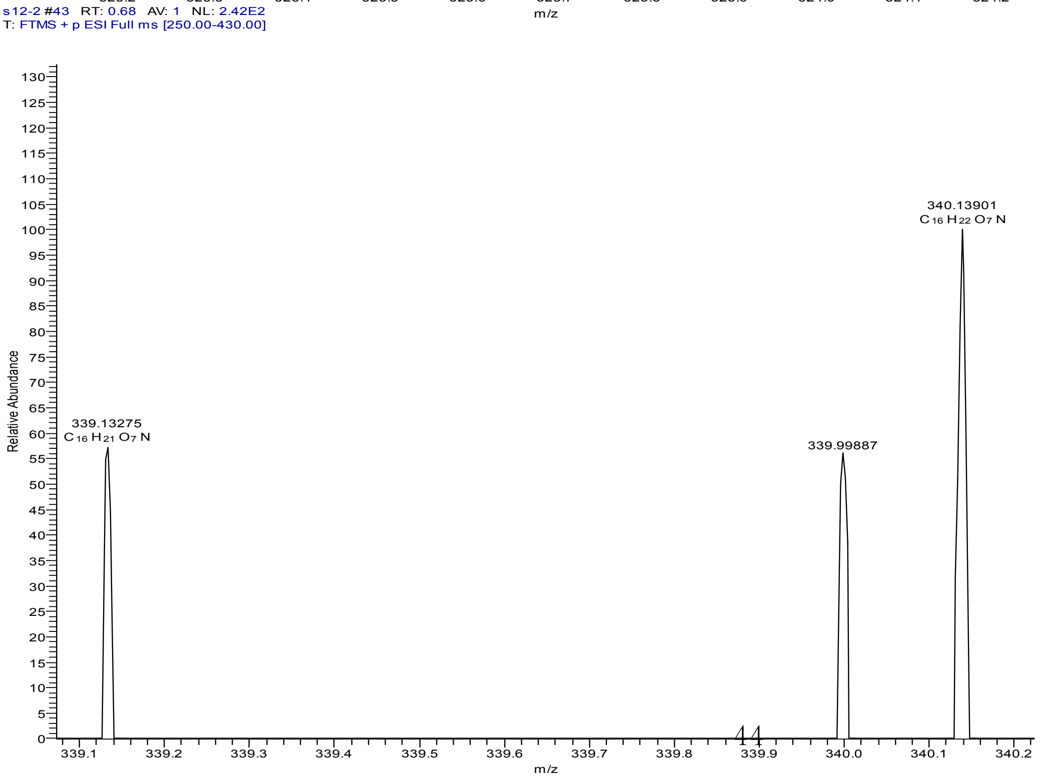

Diethyl 2-[1-(4-methoxyphenyl)-2-nitroethyl]malonate (5i)

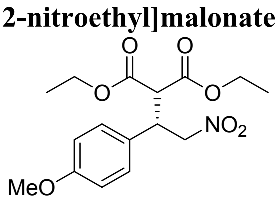

HRMS(ESI) for $C_{16}H_{22}N_1O_7[M+H]^+$ Calcd: 340.13908 Found: 340.13901

Diethyl 2-[1-(2-methoxyphenyl)-2-nitroethyl]malonate (5j)

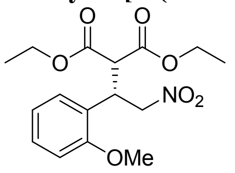

HRMS(ESI) for $C_{16}H_{22}N_1O_6$[M] Calcd: 339.13125 Found: 339.12933

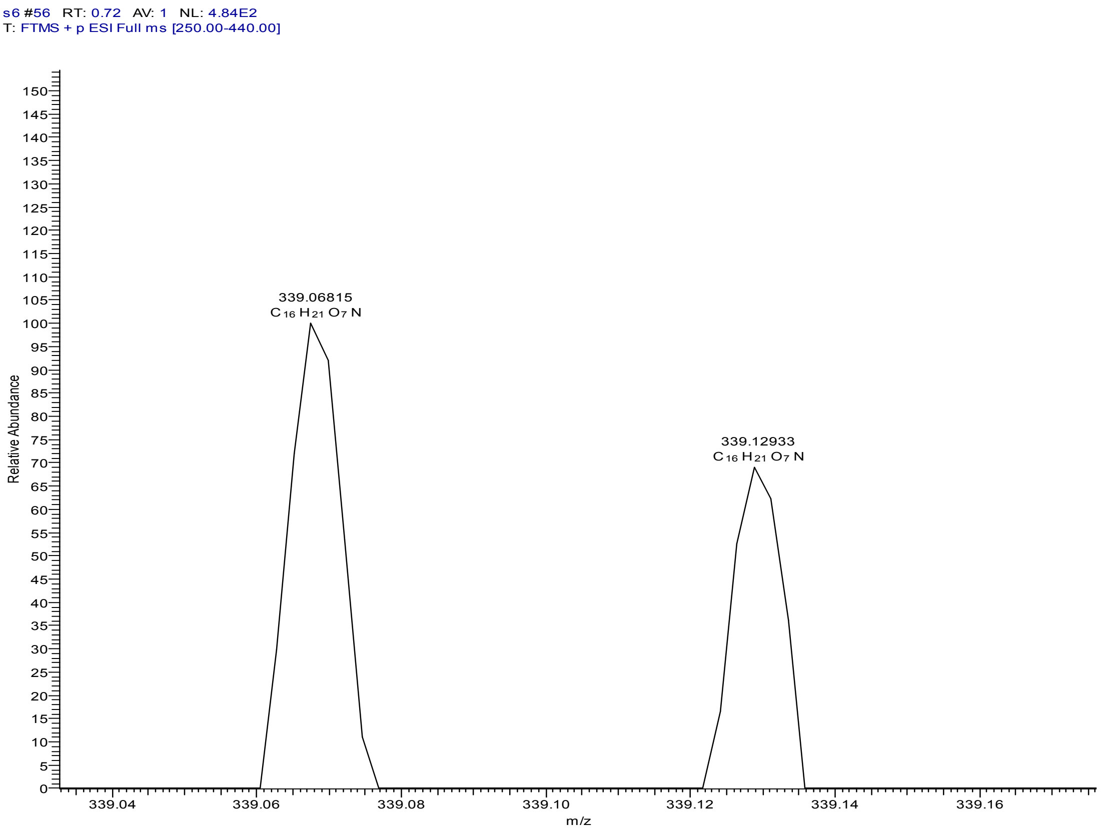

Diethyl 2-[1-(4-hydroxyphenyl)-2-nitroethyl]malonate (5k)

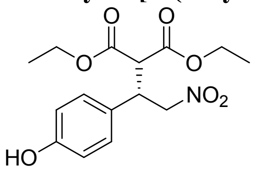

HRMS(ESI) for $C_{15}H_{20}N_1O_7[M+H]^+$ Calcd: 326.12343 Found: 326.12903

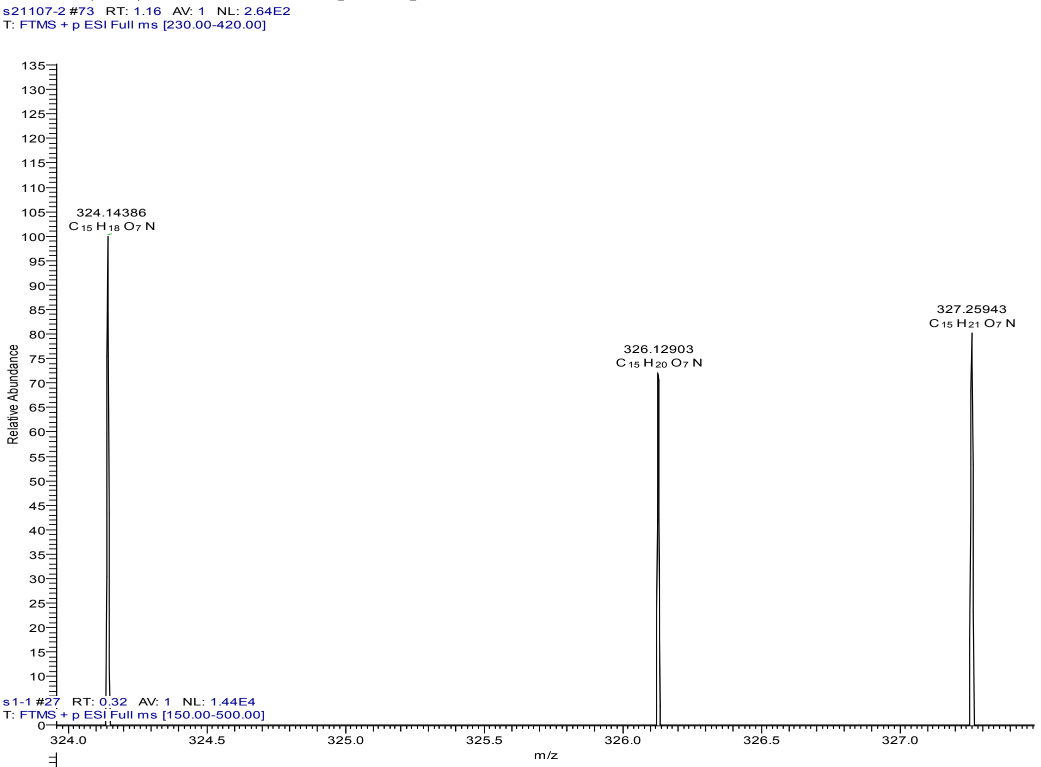

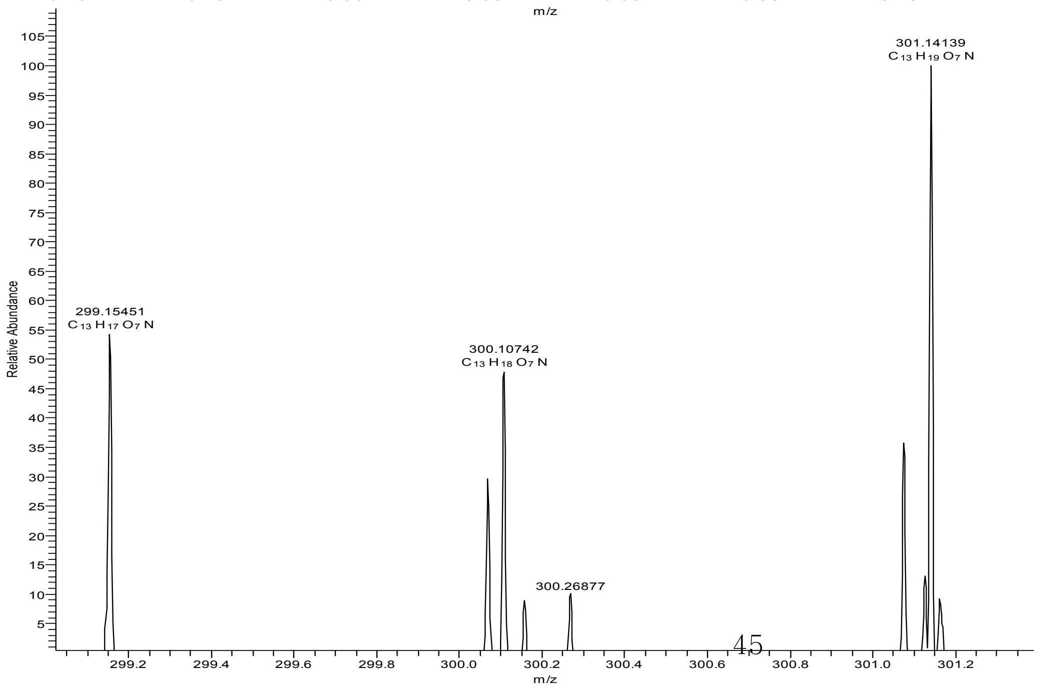

Diethyl 2-[1-(furan-2-yl)-2-nitroethyl]malonate (5l)

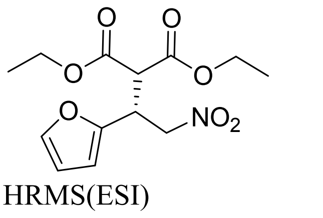

HRMS(ESI) for $C_{13}H_{18}N_1O_7[M+H]^+$ Calcd: 300.10778 Found: 300.10742

4-amino-3-phenylbutanoic acid (6a)

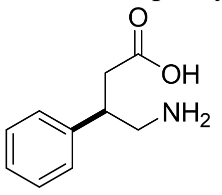

LRMS(ESI$^+$) for $C_9H_{12}ClNO_2$[M-HCl]$^+$ Calcd: 180.10, Found: 180.20.

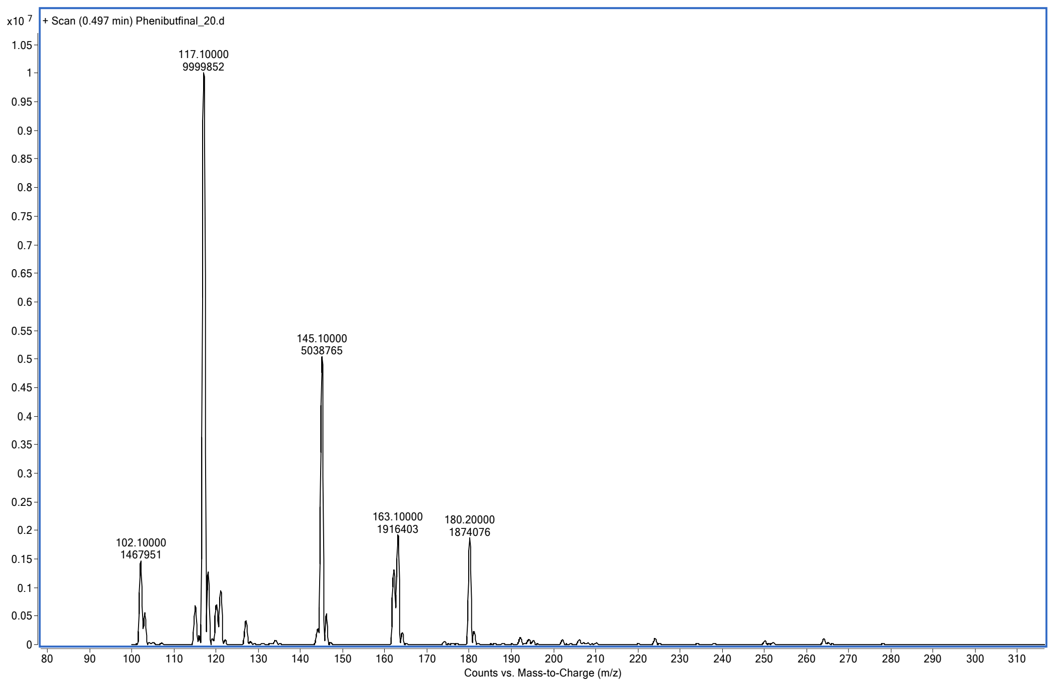

Referrence

a) D. Almaşi, D. Alonso, E. Gómez-Bengoa, C. Nájera, *J. Org. Chem.* 2009, **74**, 6163; Manzano, R.; Andrés, J. M.; Muruzábal, M.; Pedrosa, R. *Adv. Synth. Cat.* 2010, **352**, 3364; T. Okino, Y. Hoashi, T. Furukawa, X. Xu, Y. Takemoto *J. Am. Chem. Soc.* 2005, **127**, 119; b) X. Jiang, Y. Zhang, X. Liu, G. Zhang, L. Lai, L. Wu, J. Zhang, R. Wang, *J. Org. Chem.* 2009, **74**, 5562; c) K. Murai, S. Fukushima, S. Hayashi, Y. Takahara, H. Fujioka, *Org. Lett.* 2010, **12**, 964; d) Z. H. Zhang, X. Dong, D. Chen, C. J. Wang *Chem. Eur. J.* 2008, **14**, 8780;

Computational Results of DFT Calculations

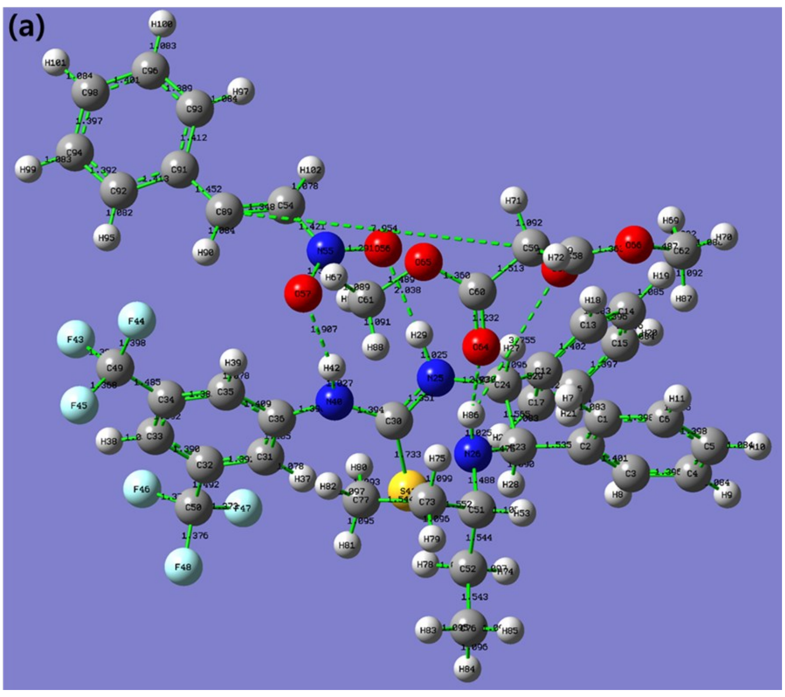

EE + Thermal Free Energy Correction = -3238.1132 Hartree

Symbolic Z-matrix:

Charge = 0 Multiplicity = 1

C	-5.33711	-0.08333	-0.70798
C	-4.72135	-1.02586	0.1279
C	-5.51868	-1.76266	1.0133
C	-6.89867	-1.55921	1.07397
C	-7.50509	-0.61787	0.23886
C	-6.71919	0.11811	-0.65341
H	-4.73363	0.48762	-1.40337
H	-5.05033	-2.48933	1.66779
H	-7.49909	-2.13674	1.76786
H	-8.57853	-0.46829	0.27377
H	-7.18386	0.8388	-1.31898
C	-2.9338	-0.26512	2.45144
C	-3.75437	0.77022	2.92218
C	-4.19836	0.77645	4.24606
C	-3.82396	-0.2487	5.11685
C	-2.9988	-1.27758	4.65522
C	-2.5541	-1.28801	3.33176

H	-4.03055	1.56771	2.24277
H	-4.83319	1.58249	4.598
H	-4.16747	-0.24451	6.14539
H	-2.69769	-2.07416	5.32663
H	-1.90844	-2.0797	2.97249
C	-3.20633	-1.2666	0.06213
C	-2.48133	-0.23616	0.99211
N	-0.99641	-0.29233	0.84397
N	-2.61133	-1.11652	-1.28117
H	-2.74189	0.75401	0.60178
H	-2.98563	-2.26917	0.42831
H	-0.56858	0.63787	0.79514
C	-0.18634	-1.35201	0.62177
C	2.66837	-2.759	0.53669
C	3.99338	-3.17489	0.43045
C	4.95985	-2.43177	-0.23688
C	4.55404	-1.24579	-0.84343
C	3.23731	-0.82429	-0.77574
C	2.2671	-1.55091	-0.0567
H	1.95611	-3.35578	1.08281
H	5.98924	-2.76103	-0.25801
H	2.95373	0.10153	-1.24978
N	1.01068	-0.94656	0.03609
S	-0.52506	-3.00617	1.01752
H	0.98595	-0.0188	-0.40362
F	6.65534	-0.13394	-0.79247
F	5.00054	0.84	-1.90119
F	6.00521	-0.95891	-2.7311
F	5.70532	-4.47832	1.40104
F	3.60848	-4.79195	2.1146
F	4.23635	-5.52715	0.12821
C	5.53917	-0.40499	-1.57066
C	4.37989	-4.4893	1.02004
C	-3.11363	-1.9956	-2.37435
C	-2.88937	-3.48069	-2.01725
H	-4.1966	-1.8546	-2.54344
C	2.61735	3.11021	0.76577
N	1.49806	2.39657	0.25834
O	0.42029	2.41708	0.96884
O	1.59968	1.73911	-0.87499
C	-4.38872	3.42936	-0.55237
C	-3.77799	3.89739	-1.86294
C	-2.78778	2.87011	-2.36843
C	-0.54584	2.58424	-3.25711
C	-6.39118	3.48894	0.79468
O	-3.80967	2.77871	0.31352
O	-3.00092	1.65936	-2.45407
O	-1.62662	3.47151	-2.74356
O	-5.67362	3.87113	-0.45166
H	0.00666	3.19416	-3.97031
H	0.08853	2.28759	-2.41958
H	-5.83479	3.82882	1.67051
H	-7.35234	3.9932	0.71635
H	-3.25803	4.84617	-1.71271
H	-4.58371	4.03977	-2.58695
C	-2.37621	-1.55399	-3.66621
H	-3.4772	-3.71403	-1.12144
H	-2.58398	-0.48508	-3.80814
C	-3.32068	-4.44163	-3.14522
C	-0.85035	-1.77574	-3.59636
H	-1.83848	-3.62522	-1.75102
H	-2.80053	-2.08522	-4.52566
H	-0.46858	-1.36285	-2.65898
H	-0.60649	-2.84291	-3.61953
H	-0.34553	-1.2957	-4.44374
H	-2.68855	-4.32704	-4.0323
H	-3.24556	-5.48219	-2.81033
H	-4.36109	-4.25306	-3.43823
H	-2.64201	-0.13566	-1.57936
H	-6.50983	2.40432	0.82703
H	-0.97914	1.70271	-3.73059
C	3.84618	2.92822	0.24133
H	3.95502	2.22227	-0.57371
C	5.06835	3.55494	0.71142
C	6.3022	2.97376	0.34353
C	5.06789	4.70822	1.5257
C	7.49728	3.52824	0.79349
H	6.31447	2.07374	-0.25671
C	6.26567	5.26153	1.95931
H	4.13008	5.17971	1.79474
C	7.48342	4.67152	1.59638
H	8.43877	3.06905	0.51862
H	6.25807	6.15236	2.57535
H	8.41572	5.10498	1.93821
H	2.36901	3.70152	1.63193

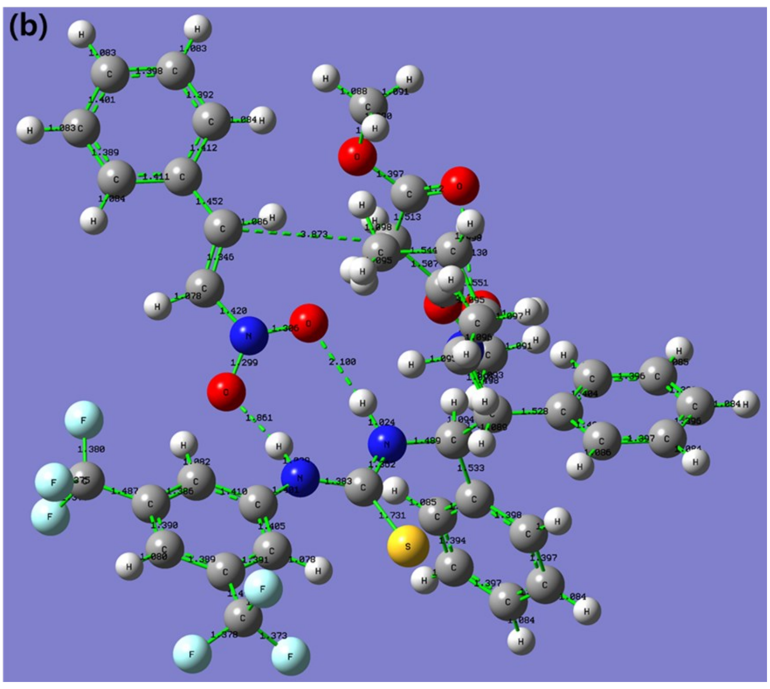

EE + Thermal Free Energy Correction = -3238.1079 Hartree

Symbolic Z-matrix:

Charge = 0 Multiplicity = 1

C	3.17585	-4.17105	0.50928
C	1.83049	-4.11001	0.90525
C	1.28201	-5.20695	1.58803
C	2.04592	-6.34614	1.8515
C	3.37875	-6.40203	1.44045
C	3.93872	-5.31001	0.7716
H	3.62423	-3.32844	-0.00149
H	0.24376	-5.16828	1.90291
H	1.6023	-7.18428	2.37733
H	3.97585	-7.28421	1.6417
H	4.97549	-5.34579	0.4547
C	0.57718	-3.3512	-1.94794
C	0.5293	-2.79668	-3.2385
C	0.38222	-3.60427	-4.36546
C	0.28625	-4.99065	-4.22156
C	0.33396	-5.5501	-2.94439
C	0.47937	-4.73909	-1.81594
H	0.57302	-1.71814	-3.34795
H	0.33449	-3.15385	-5.35087
H	0.16944	-5.62448	-5.09318
H	0.25162	-6.62379	-2.81923
H	0.51585	-5.19006	-0.83693
C	0.97139	-2.86661	0.682
C	0.83356	-2.37306	-0.79596
N	-0.14539	-1.2515	-0.82322
N	1.48801	-1.66491	1.41148
H	1.78864	-1.89202	-1.02505
H	-0.0369	-3.0771	1.03607
H	0.27167	-0.34135	-0.60995
C	-1.49052	-1.38278	-0.65423
C	-4.4922	-0.6238	-0.00914
C	-5.74751	-0.09607	0.27633
C	-6.02316	1.26085	0.17241
C	-4.98872	2.10192	-0.22227
C	-3.72276	1.60743	-0.49322
C	-3.44389	0.22842	-0.39461
H	-4.32763	-1.68792	0.04598
H	-7.0077	1.64882	0.38903
H	-2.93763	2.29732	-0.77138
N	-2.12776	-0.15562	-0.6814
S	-2.27633	-2.91292	-0.4563
H	-1.54069	0.6335	-0.9828
F	-6.24147	3.99749	0.42331
F	-5.66838	3.8474	-1.70307
F	-4.11453	4.29935	-0.20257
F	-8.06166	-0.53783	0.43832
F	-6.66648	-2.2769	0.25663
F	-6.79671	-1.13952	2.14527
C	-5.25325	3.55122	-0.42302
C	-6.81194	-1.01304	0.77309
C	1.48919	-1.72862	2.90165
C	0.04622	-1.87571	3.4345
H	2.07206	-2.59132	3.2707
C	0.7586	3.87125	-0.87354
C	1.93796	4.23178	-0.33346
C	2.48317	5.57694	-0.30614
C	1.72092	6.72902	-0.59358
C	3.85367	5.71947	0.00384
C	2.31614	7.98438	-0.57219
H	0.66312	6.6402	-0.81115
C	4.4453	6.97915	0.01211
H	4.42906	4.825	0.21344
C	3.67884	8.11274	-0.27298
H	1.72388	8.86618	-0.7839
H	5.4994	7.08049	0.2403
H	4.13733	9.09436	-0.25968
N	0.35279	2.51013	-0.89546
O	1.11362	1.57094	-0.40199
O	-0.79419	2.27118	-1.45676
H	2.56176	3.46065	0.10932

H	0.04123	4.51796	-1.35186
C	4.26544	-0.28576	-1.29
C	4.22572	1.14544	-0.82079
C	4.56821	1.29107	0.64568
C	4.7375	2.92229	2.43306
C	4.64749	-1.69949	-3.20703
O	3.88686	-1.26671	-0.65063
O	4.90328	0.43526	1.44652
O	4.43663	2.63541	1.00229
O	4.70377	-0.35151	-2.57827
H	4.72571	4.00802	2.50539
H	3.96979	2.47693	3.06589
H	3.61393	-2.0514	-3.25435
H	5.06027	-1.54879	-4.202
H	3.18379	1.48158	-0.92654
H	4.8667	1.77375	-1.43994
C	2.18367	-0.43689	3.40528
H	-0.37929	-2.81058	3.05399
H	3.18799	-0.39305	2.96417
C	-0.02905	-1.90058	4.97584
C	1.41562	0.85014	3.0339
H	-0.56352	-1.06241	3.02811
H	2.30961	-0.50005	4.4922
H	1.24645	0.88668	1.95434
H	0.44438	0.89146	3.53797
H	1.9832	1.73845	3.3396
H	0.2697	-0.93946	5.40756
H	-1.05152	-2.1154	5.30534
H	0.6295	-2.67833	5.38147
H	2.43895	-1.45817	1.07512
H	5.24493	-2.40825	-2.63093
H	5.71282	2.5118	2.69825
